# Ni-catalyzed mild hydrogenolysis and oxidations of C–O bonds via carbonate redox tags

**DOI:** 10.1038/s41467-023-38305-y

**Published:** 2023-05-05

**Authors:** Georgios Toupalas, Loélie Ribadeau-Dumas, Bill Morandi

**Affiliations:** Laboratory of Organic Chemistry, Department of Chemistry and Applied Biosciences, ETH Zurich, Zurich, Switzerland

**Keywords:** Synthetic chemistry methodology, Homogeneous catalysis

## Abstract

Oxygenated molecules are omnipresent in natural as well as artificial settings making the redox transformation of the present C–O bonds a central tool for their processing. However, the required (super)stoichiometric redox agents which traditionally include highly reactive and hazardous reagents pose multiple practical challenges including process safety hazards or special waste management requirements. Here, we report a mild Ni-catalyzed fragmentation strategy based on carbonate redox tags for redox transformations of oxygenated hydrocarbons in the absence of any external redox equivalents or other additives. The purely catalytic process enables the hydrogenolysis of strong C(sp^2^)–O bonds including that of enol carbonates as well as the catalytic oxidation of C–O bonds under mild conditions down to room temperature. Additionally, we investigated the underlying mechanism and showcased the benefits of carbonate redox tags in multiple applications. More broadly, the work herein demonstrates the potential of redox tags for organic synthesis.

## Abstract (web summary)

Catalytic redox transformations of C–O bonds are pertinent to the valorization of oxygenated hydrocarbons, but typically rely on hazardous redox agents which bring about numerous drawbacks ranging from safety hazards to waste management. Here, the authors demonstrate that such reagents can be rendered redundant by catalytically harnessing internal C–H bonds as redox equivalents.

## Introduction

The redox manipulation of C–O bonds is fundamental to the valorization of oxygenated hydrocarbons by providing an integral handle for precise oxidation state operations at the carbon atom. As such, it is a key pillar of organic synthesis which has, however, been traditionally achieved using stoichiometric methods. In this context, the development of catalytic strategies has become increasingly important. Among these, the catalytic hydrogenolysis of C–O bonds to form a reduced C–H bond is a widespread reaction of importance to timely topics such as biomass valorization^1^. Likewise, the catalytic oxidation of C–O bonds to C=O bonds is a mainstay in organic synthesis that is relevant to the production of chemicals and fuels^2,3^.

**Fig. 1:**
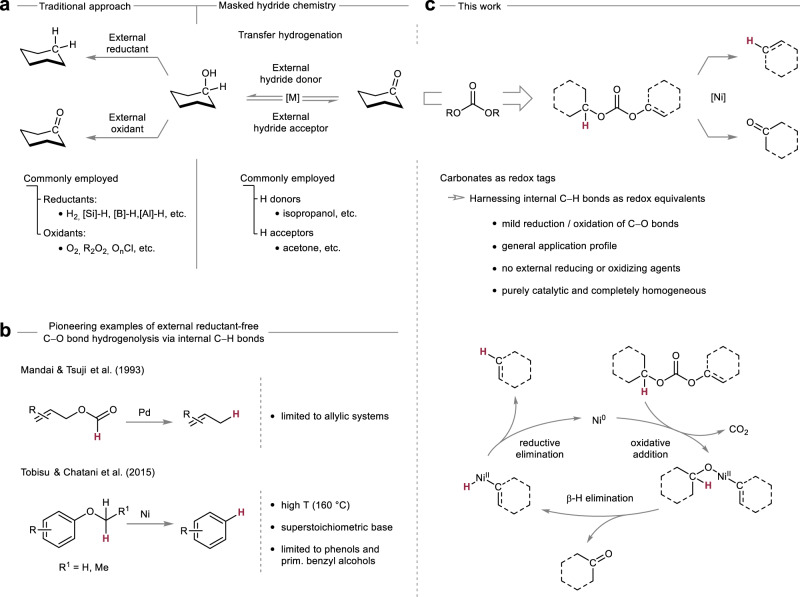
Context of this work. **a** Traditional and masked hydride redox chemistry of C–O bonds. **b** Pioneering examples of external reductant-free C–O bond reductions making use of internal C–H bonds as masked hydrides. **c** Present work harnessing internal C–H bonds for mild external reductant-free hydrogenolysis and external oxidant-free oxidations of C–O bonds on the basis of benign carbonates as redox tags.

The current paradigm for catalytic redox transformations of C–O bonds relies on the use of (super)stoichiometric terminal redox equivalents. Common examples of reagents include e.g. hydrogen^4,5^, silanes^6–9^, boranes^10,11^, oxygen^12,13^, peroxides^14^ or chlorinated oxyanions^15^ (Fig. 1a). These reactions have found widespread application, yet the use of these highly reactive reagents poses safety risks and often requires special precautions^16^. Additionally, the use of hydrogen or oxygen gas, two of the most employed reducing / oxidizing agents in catalytic strategies, involves pressurization commonly requiring specialized infrastructure such as high-pressure reactors, thus limiting the practicality of these reactions for laboratory-scale research^17,18^. Another challenge are arising functional group incompatibilities when using strong oxidants and reducing agents that can react with other important functional groups in complex molecules. Finally, the reactive and hazardous nature of the sacrificial redox agents results in waste streams that require special waste management.

In contrast to such traditional approaches, masked hydride based strategies enable the use of simple, benign organic molecules as redox equivalents and have thus had a major impact on catalytic redox transformations of C–O bonds^19,20^. The prototypical example of catalytic transfer hydrogenation circumvents the direct employment of hydrogen gas by making use of a donor such as isopropanol which carries a masked hydride in combination with a catalyst assisting in the hydride transfer from the donor molecule to the target molecule^21^. Conversely, the employment of a suitable hydride acceptor such as acetone can be used for the catalytic oxidation of alcohols to the corresponding carbonyl compounds^22,23^. Due to the benign and stable nature of the terminal redox agents, masked hydride strategies oftentimes bear advantages in terms of safety, practicality and waste disposal when compared to traditional approaches. In addition, depending on the employed redox equivalents, they offer the possibility for introducing recycling schemes as the spent redox agents can serve as suitable reagents in the reverse redox processes.

Due to the relevance of developing valorization schemes for lignin^1,24^, masked hydride strategies have been studied for the hydrogenolysis of C(sp^2^)–O bonds^25–27^. However, due to the stability of such bonds, this deoxygenation reaction still represents a considerable challenge and strategies suffer from drawbacks including, side reactions involving the employed (super)stoichiometric redox equivalents and resulting products, the regular need for (super)stoichiometric additives such as exogenous bases^28,29^ as well as the often high temperatures and suboptimal chemoselectivity, especially in combination with heterogeneous catalysts^30^.

Recently, molecular Ni-based catalysts pioneered by the groups of Martin^31^, Tobisu and Chatani^32^ as well as Hartwig^33^ have been found to enable selective hydrogenolysis of a variety of strong C(sp^2^)–O bonds. In further seminal studies, Tobisu, Chatani and co-workers additionally demonstrated that molecular Ni-based catalysts are compatible with internal hydride transfer processes enabling the hydrogenolysis of strong C(sp^2^)–O bonds in alkyl aryl ethers in the absence of external reductants albeit requiring temperatures of 160 °C (Fig. 1b)^34^. Interestingly, the group of Martin as well as the group of Agapie have shown in stoichiometric experiments that the internal hydride transfer from a methoxy ligand to an aryl ligand via a nickel center occurred at lower temperatures, depending on the starting complex even at room temperature and in short timeframes down to 2 h^35,36^.

Given the currently faced challenges in redox transformations of C–O bonds which ultimately limit advancements across numerous areas there is a need for the development of conceptual novel approaches. Inspired by pioneering studies of Mandai and Tsuji et al. demonstrating that allyl formates can be leveraged for the Pd-catalyzed external reductant-free hydrogenolysis of allylic C–O bonds^37^ (Fig. 1b), we envisaged the strategic use of redox tags^38,39^ which would unlock internal hydride transfer processes during transition metal-catalyzed fragmentations as a general platform for redox transformations of C–O bonds – a concept that would allow mild reductive as well as oxidative pathways alike as one part of the molecule would serve as a hydride acceptor while the other as a hydride donor respectively (Fig. 1c)^40^. Based on all these precedents, we reasoned that there was untapped potential for the development of a mild Ni-catalyzed redox tag strategy. We anticipated that the selection of a suitable precursor would be fundamental and identified four main requisites for it. First, it should lower the barrier for oxidative addition into the C–O bond. Second, it should enable an efficient hydride transposition. Third, it should be applicable to reduction as well as oxidation processes, as both reactions are synthetically valuable. Lastly, it should be readily accessible and result in minimal, non-hazardous waste generation. We identified alkyl aryl carbonates as ideal candidates for our envisioned purpose. Such organic carbonates represent benign, readily accessible entities that would prime the C–O bond for oxidative addition while allowing reductive as well as oxidative hydride transfer processes upon formation of benign carbon dioxide as a by-product.

Here, we show the strategic utilization of carbonate redox tags for external redox agent-free, purely catalytic hydrogenolysis as well as oxidations of C–O bonds. The developed Ni-catalyzed strategy enables mild redox transformations of a variety of redox-tagged C–O bonds while also being readily miniaturizable and compatible with fluorescence-based reactivity sensing. In addition to the synthetic investigations, this work is complemented by a mechanistic study of the underlying processes providing fundamental insights to this largely underexplored class of reactions.

## Results

### Design principle

**Fig. 2:**
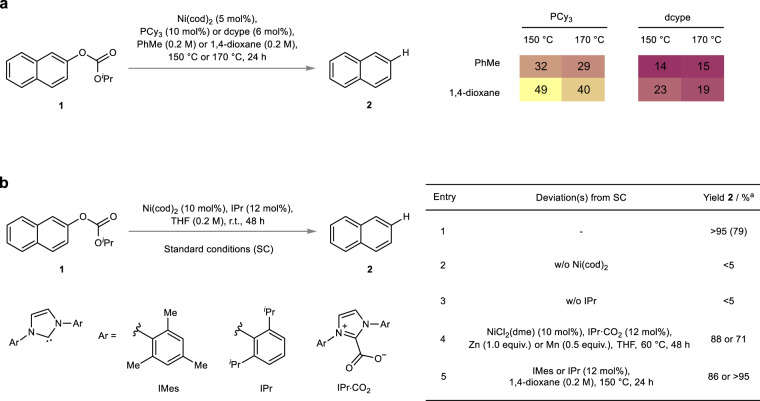
Reaction development. **a** Proof-of-principle study and the corresponding lead results. **b** Optimized conditions for the external reductant-free hydrogenolysis of isopropyl aryl carbonates and control reactions as well as Ni^II^ pre-catalyst employing conditions. ^a^GC yield (isolated yield in parentheses).

Based on the abovementioned design principle, we set out and performed a proof-of-principle study. We selected isopropyl naphthyl carbonate **1** as a suitable model substrate and studied the exogenous reductant-free hydrogenolysis to naphthalene **2** (Fig. 2a). We reasoned that a nickel phosphine based catalyst system would be an ideal starting point, as they have shown high activity in C–O bond oxidative addition^41,42^. For the reactivity assessment, Ni(cod)_2_ was selected as a pre-catalyst and combined with a trialkylphosphine ligand varying the ligand denticity, i.e. tricyclohexylphosphine (PCy_3_) or 1,2-bis(dicyclohexylphosphaneyl)ethane (dcype). Furthermore, two sets of solvents, i.e. toluene or 1,4-dioxane, and, as previous internal hydride transfer processes required forcing conditions^34^, elevated temperatures, i.e. 150 °C or 170 °C, were initially investigated.

Our reactivity assessment provided lead results as naphthalene **2** was detected under all tested conditions. The combination of Ni(cod)_2_ (5 mol%) and PCy_3_ (10 mol%) in 1,4-dioxane at 150 °C performed best affording the target molecule in promising initial yields of 49%. Thereafter, we set out for the optimization study and after investigating different parameters (see Supplementary Table 1) it was found that the usage of N-heterocyclic carbene ligands, which have proven advantageous to a range of other transformations involving transition-metal-catalyzed hydrogen transpositions^43–45^, had a significant effect on the reactivity (Fig. 2b, Entry 5). In particular, 1,3-bis(2,6-diisopropylphenyl)imidazol-2-ylidene (IPr) proved to be a privileged ligand and as a result the employment of a Ni / IPr based catalyst system ultimately allowed for the mild exogenous reductant-free hydrogenolysis of carbonate **1** to naphthalene **2** at room temperature validating the anticipated benefits of our design strategy (Fig. 2b, Entry 1). Furthermore, control reactions revealed that all reaction components are essential (Fig. 2b, Entries 2 & 3). Notably, the reaction could also be performed starting from a Ni^II^-precursor, i.e. NiCl_2_(dme), in combination with air-/moisture-stable^46^ imidazolium carboxylate IPr·CO_2_ and in the presence of a metal reductant which in the case of manganese could be reduced to substoichiometric levels (0.5 equiv.) (Fig. 2b, Entry 4). Having established the proof of principle, we showcased that the use of carbonate redox tags in combination with a Ni-catalyst indeed enabled the conversion of an internal C–H bond into a competent masked hydride resulting in the mild external reductant-free hydrogenolysis of a C(sp^2^)–O bond. Notably, the process does not require any additive and only generates catalytic metal waste alongside benign stoichiometric by-products, that is acetone and carbon dioxide.

### Mechanistic investigation

**Fig. 3:**
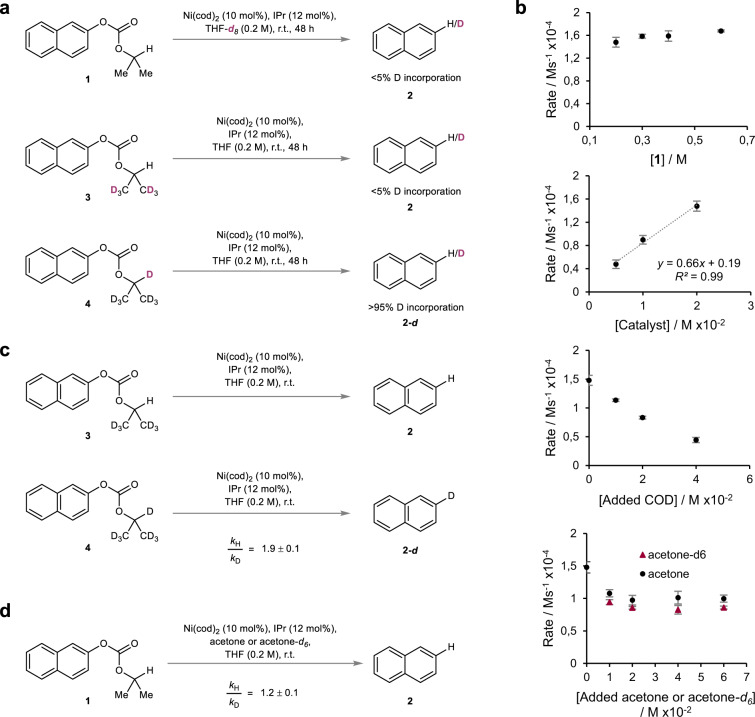
Mechanistic experiments. **a** Deuterium-labeling study. **b** Kinetic interrogation of the individual reaction parameters. Error bars represent the standard deviation of three independent experiments. **c** Primary KIE based on the difference of the initial rates of independent reactions employing either carbonate **3** or carbonate **4**. **d** β-Secondary KIE regarding added acetone or acetone-*d*_*6*_ based on the difference of the initial rates of individual reactions.

Intrigued by the mild nature of the hydrogenolysis through the use of carbonate redox tags, we next aimed to shed light on the underlying mechanism (Fig. 3). As such reactions are largely underexplored and consequently lack clear fundamental understanding, a mechanistic analysis would provide a basis for future progress. We began our mechanistic investigation by conducting a deuterium labeling study to identify the origin of the hydrogen atom in the newly formed C–H bond of the product (Fig. 3a). According to our working hypothesis (Fig. 1c), the hydrogen atom should arise from the 2-position of the isopropyl rest of the carbonate. In line with this hypothesis, performing the model reaction in perdeuterated THF (THF-*d*_*8*_) or with deuterated carbonate **3** did not result in any detectable deuterium incorporation, whereas employment of deuterated carbonate **4** gave rise to deuterated naphthalene **2-*d***. Having traced the origin of the hydrogen atom, we continued our investigation by interrogating the mechanism through kinetic analysis (Fig. 3b). The initial rates of the reaction were measured independently by varying the concentration of carbonate **1**, catalyst, added 1,5-cyclooctadiene (COD) or added acetone while keeping the corresponding remaining parameters constant. Variation of the concentration of carbonate **1** had a negligible effect on the initial rate of the reaction resulting in an approximately zeroth order in the starting material suggesting that reaction of carbonate **1** with the catalyst is not involved in the turnover-limiting step of the reaction. In contrast, the initial rate was directly proportional to the concentration of catalyst resulting in a rate increase with increasing catalyst loadings leading to approximately first order behaviour. Varying the concentration of COD in the reaction revealed approximately inverse first order dependence on COD indicating that the dissociation of one equivalent of COD from the resting state is necessary prior to the turnover-limiting step of the reaction. Lastly, it was found that adding acetone, an inherent by-product of the reaction, to the reaction mixture resulted in apparent saturation kinetics which would be in accordance with a change in resting state during the reaction as a function of the concentration of acetone^45^. At low acetone concentrations, an inverse order in acetone was observed which is consistent with a dissociation event prior to the turnover-limiting step of the reaction. At higher concentrations, acetone displays zeroth order pointing towards an altered resting state incorporating an acetone molecule. Having gathered information regarding the interplay of the individual components during the catalytic cycle, we pursued our investigation by studying possible isotope effects (Fig. 3c, d). In a first set of experiments we focused on the determination of any primary kinetic isotope effect (KIE) by comparing the initial rates of two separate reactions employing either carbonate **3** or carbonate **4** under otherwise standard reaction conditions (Fig. 3c). As a result, a normal primary KIE of 1.9 ± 0.1 was found. In combination with the approx. zeroth order in starting material, this suggested that either β-H elimination or the subsequent release of the organic products, i.e. reductive elimination to form naphthalene **2** or prior acetone dissociation, could be turnover-limiting in the catalytic cycle^47–49^.

**Fig. 4:**
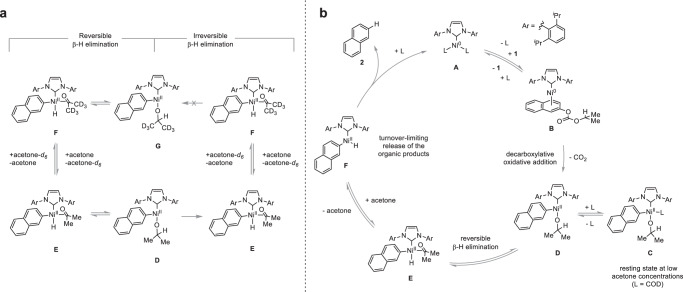
Mechanistic rationale. **a** Ir-/reversible β-H elimination scenarios and implications towards a β-secondary KIE. In the presence of acetone-*d*_*6*_, a reversible pathway would involve a C–H bond cleavage from alkoxo complex **D** and deuterated alkoxo complex **G** which would be consistent with the observation of a KIE, whereas an irreversible pathway would lack C–H bond cleavage from deuterated alkoxo complex **G** which would be consistent with the lack of any significant KIE. **b** Plausible mechanism based on the observations made during the mechanistic investigation of the reaction. Starting from intermediate **A**, association of **1** affords pre-oxidative addition complex **B** which undergoes decarboxylative oxidative addition to COD bound alkoxo intermediate **C**, the resting state of the reaction at low acetone concentrations. Dissociation of COD affords alkoxo complex **D**. Thereafter, a reversible β-H elimination results in the formation of hydrido complex **E** with coordinated acetone. Finally, turnover-limiting release of the organic products yields product **2** and acetone regenerating Ni^0^ complex **A** thereby closing the catalytic cycle.

In order to differentiate between these two scenarios and to probe the nature of the β-H elimination, that is whether it is reversible or not, we decided to evaluate any potentially occurring β-secondary KIE by comparing the initial rates of individual reactions in the presence of added acetone or acetone-*d*_*6*_ (Fig. 3d). In a scenario where the β-H elimination pathway is reversible, alkoxo complex **D** would exist in an equilibrium with deuterated alkoxo complex **G** in the presence of acetone-*d*_*6*_, leading to C–H bond cleavages from both species. This would be consistent with the observation of a β-secondary KIE due to better hyperconjugative stabilization of the transition state by the β-C–H bonds compared to the β-C–D bonds during rehybridization from C(sp^3^) to C(sp^2^) (Fig. 4a)^50,51^. In contrast, an irreversible β-H elimination pathway would not lead to alkoxo complex **G** and could explain the lack of any significant β-secondary KIE (Fig. 4a). Accordingly, studies were conducted over a range of acetone-*d*_*6*_ concentrations, all of which displayed a normal β-secondary KIE resulting in an overall value of 1.2 ± 0.1 indicating a reversible β-H elimination pathway^52^.

Based on all the collected mechanistic information, a plausible reaction mechanism can be proposed (Fig. 4b). The catalytic cycle is likely to begin with tricoordinated Ni^0^ complex **A**. Association of carbonate **1** affords pre-oxidative addition complex **B** which undergoes decarboxylative oxidative addition forming alkoxo intermediate **D** which interconverts to species **C**^53^. Based on the kinetic data (zeroth order in substrate and approx. inverse first order in COD), COD bound alkoxo Ni^II^ complex **C**, which lacks a readily accessible open coordination site for β-H elimination, is likely to be the resting state of the reaction at low acetone concentrations. From there dissociation of COD liberates a coordination site and affords tricoordinate alkoxo complex **D**. Thereafter, a reversible β-H elimination^19,20^ results in the formation of hydrido complex **E** with coordinated acetone. Finally, turnover-limiting release of the organic products yields naphthalene **2** as well as acetone and regenerates starting complex **A** thereby closing the catalytic cycle. Due to the observed inverse first order in acetone at low acetone concentrations and the considerable primary KIE, this step is likely to be composed of acetone decoordination prior to turnover-limiting reductive elimination at the early stages of the reaction^54–56^.

### Scope study

**Fig. 5:**
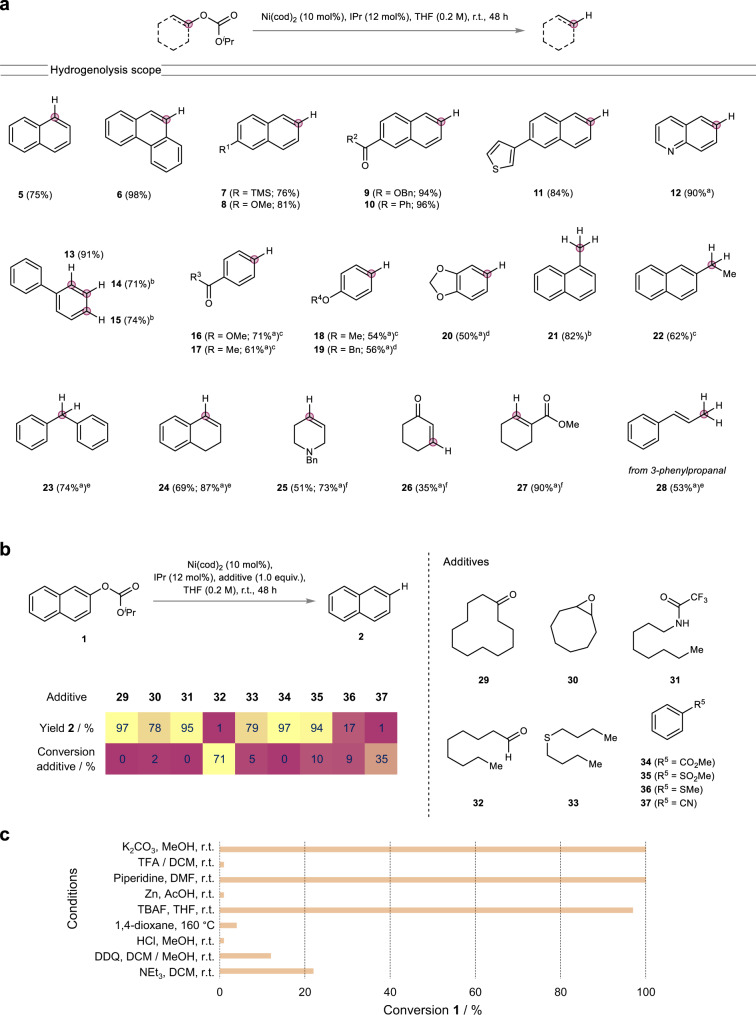
Scope of the hydrogenolysis. **a** External reductant-free hydrogenolysis of carbonates containing a variety of C–O bonds. Isolated yields in parentheses. ^a^GC yield; ^b^60 °C; ^c^1,4-dioxane, 120 °C; ^d^Ni(cod)_2_ (20 mol%), IPr (24 mol%), 1,4-dioxane, 160 °C; ^e^100 °C; ^f^80 °C. **b** Additive study with redox- and/or base-sensitive functionalities. **c** Stability studies of carbonate **1** under various conditions.

Having expanded the understanding of the fundamental reactivity, we proceeded to investigate the synthetic scope of the strategy. Therefore, we first focused on the catalytic external reductant-free hydrogenolysis and tested a variety of different C–O bond classes (Fig. 5a). Among these, multiple aryl C(sp^2^)–O bonds were readily cleaved at room temperature (**5**–**13**). Less reactive non-π-extended aromatic systems could also be employed albeit requiring heating (**16**–**20**). Moreover, several functional groups, e.g. esters (**9,** **16,** **27**), ketones (**10,** **17,** **26**), thiophenes (**11**) or benzylesters/-ethers (**9,** **19**), which are usually sensitive to reducing conditions, were compatible with the reaction conditions. Importantly, the strategy proved to be highly chemoselective for carbonate tagged C(sp^2^)–O bonds and orthogonal to other aryl C(sp^2^)–O bonds such as in methyl aryl ethers (**18**) or benzyl aryl ethers (**19**) which have been shown to be reactive in the presence of a Ni-catalyst^6,31–33^ even in the absence of external reductants^34^. In addition, no overreduction of the aromatic rings was observed. Primary as well as secondary benzyl alcohol derived carbonates containing a C(sp^3^)–O bond could be hydrogenated to the corresponding hydrocarbons (**21**–**23**) in good yields requiring temperatures between 60 °C to 120 °C. Finally, the strategy was applied to enol carbonates in order to deoxygenate a variety of substrates affording the corresponding alkenes in good yields (**24**–**27**) requiring temperatures of 80 °C or 100 °C. Moreover, 3-phenylpropanal derived enol carbonate could be employed and afforded *trans*-β-methylstyrene (**28**), in which the double bond had migrated. Importantly, to this date similar enol reductions have relied entirely on the use of external reducing agents and most often have required the installment of highly reactive groups such as triflates^57,58^. In contrast, in the present case enol derived isopropyl carbonates serve as a basis for the straightforward synthesis of alkenes without the use of an external reducing agent highlighting the benefits of our strategy. Having demonstrated that the approach is suitable for a variety of C–O bonds, we went on and performed an additive study to further probe the functional group compatibility (Fig. 5b). Therefore, we chose the model hydrogenolysis of carbonate **1** under standard conditions and examined the performance in the presence of various additives containing redox- and/or base-sensitive functional groups monitoring the conversion of the corresponding additive. It was found that the reaction is compatible with multiple redox- and/or base-sensitive functional groups such as enolizable ketones (**29**), epoxides (**30**), trifluoroacetamides (**31**), dialkylthioethers (**33**), esters (**34**) and sulfones (**35**), whereas aldehydes (**32**), thioanisoles (**36**) and nitriles (**37**) are largely incompatible.

**Fig. 6:**
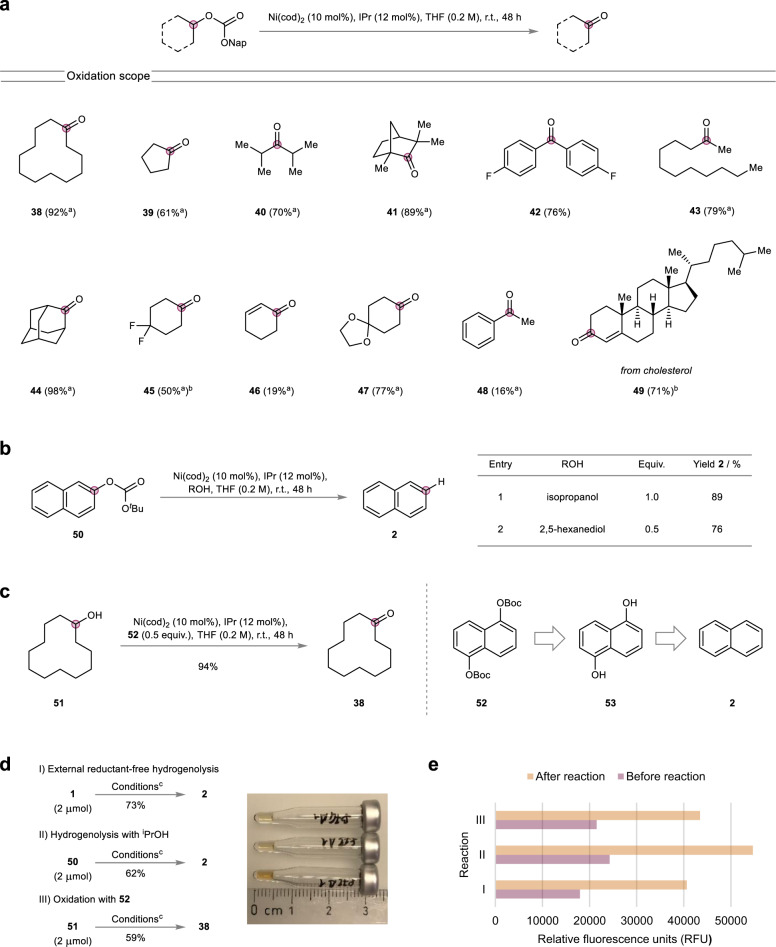
Scope of the oxidation and applications. **a** External oxidant-free oxidation of alcohols. Isolated yield in parentheses. ^a^GC yield; ^b^60 °C. **b** Hydrogenolysis of non-innocent *tert*-butyl carbonates. **c** Non-innocent biscarbonates as oxidants. **d** Miniaturization experiments. ^c^see Supplementary Tables 10–12. **e** Fluorescence-based reactivity assay comparing the fluorescence before and after the reactions.

Having investigated the scope of the external reductant-free hydrogenolysis of isopropyl carbonate tagged C–O bonds, we continued by studying the oxidation of C(sp^3^)–O bonds making use of naphthyl carbonates as a redox tag for C–O bond oxidation (Fig. 6a). The corresponding carbonates were again readily prepared and allowed for the mild catalytic oxidation of a variety of substrates in the absence of an external oxidant. Cyclic as well as acyclic secondary alcohol derived carbonates proved reactive and afforded the corresponding ketones in good yields. Moreover, sterically demanding α,α-disubstitution proved compatible with the reaction (**40,** **41**). Secondary diarylsubstituted benzylic positions (**42**) performed well, whereas alkyl aryl substituted (**48**) as well as allylic (**46**) C–O bonds performed sluggishly. Finally, performing the reaction on cholesterol derived naphthyl carbonate gave straightforward access to cholestenone **49**, in which the double bond had migrated.

Next, we envisaged to merge the presented masked hydride chemistry with our previously introduced concept of non-innocent electrophiles (NIE)^42,59^. Having showcased earlier that *tert*-butyl aryl carbonates can serve as suitable NIEs for a variety of catalytic transformations, we anticipated that the combination with isopropanol as a benign reductant could be used for the base-free hydrogenolytic cleavage of C–O bonds under mild conditions (Fig. 6b). Accordingly, carbonate **50** was reacted with isopropanol at room temperature and cleanly afforded naphthalene **2** in excellent yield (Fig. 6b, Entry 1). Notably, no excess of reductant was required as equimolar amounts of isopropanol were sufficient for the hydrogenolysis. On the basis of this, we envisaged the use of biomass-derived 2,5-hexanediol^60^ as a suitable external hydride donor. This diol would, in principle, permit the usage of substoichiometric amounts of it. Indeed, the amount of added reducing agent could be halved (Fig. 6b, Entry 2). Notably, apart from benign carbon dioxide and *tert*-butanol this approach does not generate any other stoichiometric waste, creating 2,5-hexanedione, a key platform chemical in potential cellulose based value chains^60^, as a substoichiometric by-product. After demonstrating that *tert*-butyl aryl carbonates could be used as competent NIEs in mild reduction strategies, we envisioned to harness this ability for the opposite redox process and set out to employ *tert*-butyl aryl carbonates as benign redox equivalents for the direct catalytic oxidation of alcohols (Fig. 6c). Building on the diol strategy in the hydrogenolysis, we anticipated that the utilization of dihydroxynaphthalene derived carbonates would enable the mild oxidation of alcohols with substoichiometric amounts of added oxidant. Consequently, we studied a variety of dihydroxynaphthalene derived carbonates (see Supplementary Table 8) for the oxidation of model alcohol **51** and found that 1,5-dihydroxynaphthalene derived bis(carbonate) **52** could be used in substoichiometric amounts (0.5 equiv.) yielding ketone **38** in excellent yield and under mild conditions. Importantly, the substoichiometrically generated naphthalene during the reaction represents the industrial precursor for the synthesis of 1,5-dihydroxynaphthalene^61^ and thus this oxidation approach offers the potential for the introduction of recycling schemes of the spent oxidant.

### Applications

Due to their more resistant nature towards hydrolysis compared to esters, the fact that they can be used for highly selective monoprotections of polyols as well as the fact that enol carbonates can mask the reactivity of the enol double bond, carbonates have found widespread use as protecting groups^62^. Within this context, the present alkyl aryl carbonates offer additional reactive opportunities as redox active protecting groups. In order to elucidate the stability profile of this moiety, we decided to monitor the decomposition of carbonate **1** under different conditions additionally aiming at identifying potential orthogonal deprotection schemes (Fig. 5c). Consequently, it was found that carbonate **1** is completely stable under acidic conditions and that it is thermally stable up to 160 °C which is in stark contrast to thermolabile *tert*-butyl carbonates. Moreover, carbonate **1** is completely stable under reducing conditions traditionally employed for Troc-deprotection and is also not significantly affected by oxidizing conditions traditionally used for PMB-deprotection. In line with typical carbonate reactivity, complete conversion to 2-naphthol was observed under basic reaction conditions as well as in the presence of TBAF. However, tertiary amines were largely compatible. Taken together, the study demonstrated that isopropyl aryl carbonates are compatible with a variety of conditions and allow for orthogonal deprotection schemes, ultimately underlining the potential to serve as redox active protecting groups in synthetic strategies.

Having showcased that the developed C–H harnessing strategy is applicable to a variety of redox transformations of different C–O bonds and also offers prospects in the use as a redox active protecting group concept, we set out to study the miniaturizability of the developed reactions (Fig. 6d). High-throughput experimentation (HTE) has become an enabling tool in modern chemical space exploration and the miniaturizability of reactions is an ever more important parameter for the optimal implementation into HTE setups^63,64^. As a result, the developed transformations were readily downsized to the micromole-scale (2 μmol, 10 μL total reaction volume) without any adjustments to the conditions and, due to their complete homogeneity, were compatible with liquid handling techniques, another key parameter for the optimal utilization in HTE platforms. Likewise, the fast analysis of reaction outcomes is essential to HTE and still represents a substantial limiting factor^65^. In that regard, we previously introduced a fluorescence-based reactivity assay based on substoichiometric amounts of 4-methylumbelliferone (10 mol%) as an exogenous fluorescent sensor which enabled the fast, convenient and reliable analysis of reactions and was suitable for reaction discovery. This assay proved equally applicable to the present redox tag strategy and enabled the straightforward detection of reactivity via visible inspection after irradiation of the samples at 366 nm with a common benchtop UV-lamp (see Supplementary Fig. 2) as well as via the use of a fluorescence plate reader (Fig. 6e).

In summary, we have developed a Ni-catalyzed fragmentation strategy that allows for mild redox transformations of C–O bonds. We have showcased that benign carbonates can be used as redox tags for the conversion of internal C–H bonds of a molecule into competent redox equivalents. Importantly, we have provided key mechanistic insights that pave the way for future advancements of this novel strategy. We applied the concept to the external reductant-free hydrogenolysis and to the external oxidant-free oxidation of a variety of C–O bonds. Notably, the hydrogenolysis of enol carbonates proved feasible providing straightforward access to alkenes from ketones and aldehydes. In addition, we combined the strategy with the concept of NIEs and demonstrated that non-innocent *tert*-butyl aryl carbonates offer additional reactive opportunities. Finally, we showed that the studied alkyl aryl carbonates offer potential benefits as redox active protecting groups and that the developed reactions can be readily downsized without any adjustment to the conditions in addition to the compatibility with a previously developed fluorescence-based reactivity assay allowing for fast, convenient and reliable analysis. In a broader context, we believe that this work presents a conceptual blueprint for the utilization of innate molecular moieties as redox tags and their diverse application profile in organic synthesis.

## Methods

### General procedure for redox transformations of C–O bonds via carbonate redox tags

Inside an Ar-filled glovebox, an oven-dried 16 mL vial was charged with the corresponding carbonate (0.5 mmol, 1.0 equiv.), Ni(cod)_2_ (10 mol%) and IPr (12 mol%). Thereafter THF (2.5 mL) was added and the vial was sealed. The reaction mixture was stirred at room temperature for 48 h. After that, the vial was decapped and the volatiles were removed under reduced pressure. Purification via flash column chromatography afforded the title compounds.

## Supplementary information

Supplementary Information

Peer Review File

## Data Availability

The data generated in this study and that support the findings of it are provided within the article and in the Supplementary Information. All data are also available from the authors upon request. Crystallographic data for compound **49** was deposited on the Cambridge Structural Database and is freely available via the Cambridge Crystallographic Data Centre under CCDC number 2220801. The data can be obtained free of charge via www.ccdc.cam.ac.uk/structures, or by emailing data_request@ccdc.cam.ac.uk, or by contacting The Cambridge Crystallographic Data Centre, 12 Union Road, Cambridge CB2 1EZ, UK; fax: +44 1223 336033.
