# Supplementary material for: Ni-catalyzed mild hydrogenolysis and oxidations of C–O bonds via carbonate redox tags
Source: Nat Commun. 2023 May 5;14:2604. doi: 10.1038/s41467-023-38305-y (PMC10163265; doi:10.1038/s41467-023-38305-y)
Supplement: Supplementary file 1 — Supplementary Information [file 41467_2023_38305_MOESM1_ESM.pdf]

Supplementary Information

Ni-catalyzed mild hydrogenolysis and oxidations of C–O bonds via carbonate redox tags

Georgios Toupalas[1], Loélie Ribadeau-Dumas[1], Bill Morandi[1]*

[1]Laboratory of Organic Chemistry, Department of Chemistry and Applied Biosciences, ETH Zürich, Zürich, Switzerland

*Correspondence to: bill.morandi@org.chem.ethz.ch

Supplementary Methods

General Considerations

All reactions were carried out in dried glassware (oven- or flame-dried) with magnetic stirring using PTFE coated stir bars under N_2 or Ar atmosphere. Syringes were used to transfer reagents and solvents and were purged if used outside the glovebox with N_2 (3 times) prior to use.

Solvents

All reactions were carried out in dry solvents. The dry solvents were either purchased from commercial suppliers (Acros Organics, Extra dry over molecular sieves, AcroSeal®) or collected from a LC Technology Solutions solvent purification system and stored over molecular sieves inside an Ar-filled glovebox.

Reagents

All reagents were obtained from commercial sources and used as received unless otherwise stated. Air- and moisture sensitive compounds were stored in an Ar-filled glovebox and used therein.

$Ni(cod)_2$ was purchased from Strem Chemicals, Inc. and stored inside an Ar-filled glovebox at -30 °C. 1,3-Bis(2,6-diisopropylphenyl)-1,3-dihydro-2*H*-imidazol-2-ylidene (IPr) was purchased from Sigma-Aldrich, Co. or Tokyo Chemical Industry Co, Ltd. and stored inside an Ar-filled glovebox at -30 °C. Manganese (325 mesh) was purchased from Strem Chemicals, Inc. Zinc (325 mesh) was purchased from abcr GmbH.

Following reagents were prepared according to literature procedures:

- Benzyl 6-hydroxy-2-naphthoate[1]
- 6-(Trimethylsilyl)naphthalen-2-ol[2]
- 6-(Thiophen-3-yl)naphthalen-2-ol[3]
- *tert*-Butyl naphthalen-2-yl carbonate (**50**)[4]
- 2,2,2-Trifluoro-*N*-octylacetamide (**31**)[5]

Chromatography

Flash column chromatography was performed with silica gel 60 (40-63 µm, 230-400 mesh) from SiliCycle using technical grade solvents. Analytical thin layer chromatography (TLC) was performed on silica gel 60 F_{254} glass plates from Merck. The chromatograms were visualized under UV light at 254 nm and / or by staining with an appropriate TLC stain followed by heating if necessary.

NMR

NMR spectra were recorded either on a Bruker Neo 400 MHz spectrometer equipped with a BBFO smart probe, a Bruker Avance III 400 MHz spectrometer equipped with a BBFO smart probe or a Bruker Neo 500 MHz spectrometer equipped with a BBFO smart probe at 25 °C. Chemical shifts are reported as δ-values in ppm relative to the residual proton signal of the deuterated solvent downfield to TMS. ^{1}H NMR data is reported the following way: chemical shift (multiplicity, coupling constants, number of protons). ^{13}C NMR and ^{19}F NMR spectra were recorded with ^{1}H decoupling and corresponding data is reported the following way: chemical shift (multiplicity and coupling constants stated only if other than singlet). Multiplicities are denoted as follows: s (singlet), d (doublet), t (triplet), q (quartet), sept (septet), m (multiplet), br (broad) and/or combinations thereof. Coupling constants *J* are reported in Hz.

GC

Qualitative analysis of samples was performed using GC-MS on a Shimadzu GC-2010 Plus / GCMS-QP2020 system (column: Macherey-Nagel OPTIMA 5 MS Accent - 0.25 µm, 30.0 m x 0.25 mm; carrier gas: helium). Unless stated otherwise, quantitative analysis of samples was performed using GC-FID on a Shimadzu GC-2025 instrument (column: Macherey-Nagel OPTIMA 5 MS Accent - 0.25 µm, 30.0 m x 0.25 mm; carrier gas: hydrogen) with *n*-decane as an internal standard. Samples were filtered over a short plug of silica or celite and diluted with EtOAc prior to submission.

HRMS

HRMS data was acquired by the mass spectrometry service (MoBiAS, Molecular and Biomolecular Analysis Service) in the Laboratory for Organic Chemistry at ETH Zurich. For EI measurements a Thermo scientific Q Exactive GC Orbitrap instrument with direct probe was used. For ESI measurements a Bruker Daltonics maXis ESI-QTOF or solariX ESI-FTICR-MS was used. Data is reported in m/z.

X-Ray

X-ray single crystal structure analysis was performed by the Small Molecule Crystallography Center (SMoCC) of the Department of Chemistry and Applied Biosciences at ETH Zurich. Single crystalline samples were measured on the following instrument: Rigaku Oxford Diffraction XtaLAB Synergy-S Dualflex kappa diffractometer equipped with a Dectris Pilatus 300 HPAD detector and using microfocus sealed tube Cu-Kα radiation with mirror optics (λ = 1.54178 Å).

Fluorescence spectroscopy

Fluorescence measurements were conducted on a Tecan Infinite F200 PRO microplate reader (excitation wavelength: 360 nm; emission wavelength: 465 nm).

Synthesis of starting materials

General Procedure A (GPA)

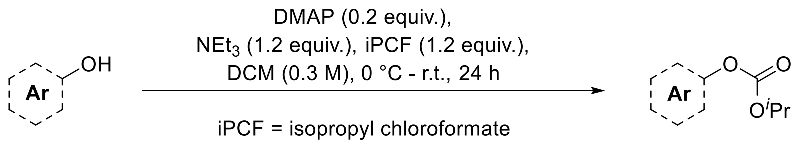

Isopropyl chloroformate (1.0 M in PhMe, 6 mmol, 1.2. equiv.) was added dropwise to a mixture of the corresponding alcohol (5.0 mmol, 1.0 equiv.), DMAP (1 mmol, 0.2 equiv.) and NEt_3 (6 mmol, 1.2 equiv.) in DCM (15 mL) at 0 °C. The reaction was stirred for 10 min at 0 °C before the cooling was removed and stirring continued for 24 h at room temperature. Thereafter, water was added and the layers separated. The org. layer was dried over $MgSO_4$, filtered and the solvent removed under reduced pressure. Purification via flash column chromatography afforded the title compounds.

General procedure B (GPB)

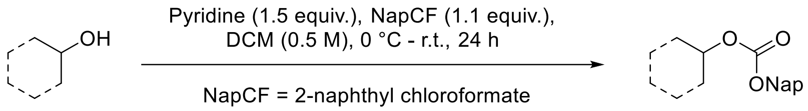

Pyridine (3.75 mmol, 1.5 equiv.) was added dropwise to a mixture of the corresponding alcohol (2.5 mmol, 1.0 equiv.) and 2-naphthyl chloroformate (2.75 mmol, 1.1 equiv.) in DCM (5 mL) at 0 °C. The reaction was stirred for 10 min at 0 °C before the cooling was removed and stirring continued for 24 h at room temperature. Thereafter, water was added and the layers separated. The org. layer was dried over $MgSO_4$, filtered and the solvent removed under reduced pressure. Purification via flash column chromatography afforded the title compounds.

2-Naphthyl chloroformate (S1)

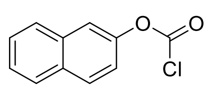

$C_{11}H_7ClO_2$
MW: 206,63
S1

A solution of 2-naphthol (2.9 g, 20 mmol, 1.0 equiv.) and DIPEA (3.5 mL, 36 mmol, 1.8 equiv.) in DCM (20 mL) was added dropwise to a solution of triphosgene (3.1 g, 10.5 mmol, 0.5 equiv.) in DCM (20 mL) at 0 °C. The resulting mixture was stirred for 20 min at 0 °C before the cooling bath was removed and stirring continued for 18 h at room temperature. Thereafter the reaction was quenched by the addition of water and the layers were separated. The org. layer was washed with water and the combined aq. layers extracted with DCM. After that, the org. layers were combined and dried over $MgSO_4$. Filtration, removal of the solvent under reduced pressure and drying under high vaccum afforded the title compound as an off-white solid (3.9 g, 18.9 mmol, 94%). **^{1}H NMR** (500 MHz, $CDCl_3$) δ 7.95 – 7.84 (m, 3H), 7.76 – 7.72 (m, 1H), 7.61 – 7.53 (m, 2H), 7.36 (dd, J = 8.9, 2.4 Hz, 1H). **^{13}C NMR** (125 MHz, $CDCl_3$) δ 149.8, 149.3, 133.6, 132.0, 130.2, 128.0, 128.0, 127.3, 126.7, 119.4, 118.0. **HRMS (ESI):** calc. for $C_{11}H_7ClO_2^+$ $[M]^+$: 206.0129; found: 206.0129.

Isopropyl naphthalen-2-yl carbonate (1)

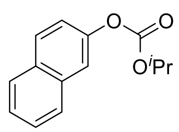

$C_{14}H_{14}O_3$
MW: 230,26
1

According to **GPA** using 2-naphthol (20 mmol). Purification via flash column chromatography (SiO_2, ^{n}Hex / EtOAc, 20 / 1) afforded the title compound as a white solid (3.97 g, 17.3 mmol, 86%). **^{1}H NMR** (500 MHz, $CDCl_3$) δ 7.89 – 7.80 (m, 3H), 7.69 – 7.65 (m, 1H), 7.55 – 7.44 (m, 2H), 7.34 (dd, *J* = 8.9, 2.4 Hz, 1H), 5.04 (sept, *J* = 6.3 Hz, 1H), 1.42 (d, *J* = 6.3 Hz, 6H). **^{13}C NMR** (125 MHz, $CDCl_3$) δ 153.4, 148.9, 133.8, 131.6, 129.6, 127.9, 127.8, 126.7, 125.9, 120.8, 118.2, 73.3, 21.9. **HRMS (ESI):** calc. for $C_{14}H_{14}NaO_3^+$ $[M+Na]^+$: 253.0835; found: 253.0838.

Isopropyl naphthalen-1-yl carbonate (S2)

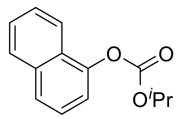

$C_{14}H_{14}O_3$
MW: 230,26
S2

According to **GPA** using 1-naphthol. Purification via flash column chromatography (SiO_2, ^{n}Hex / EtOAc, 20 / 1) afforded the title compound as a white solid (1.02 g, 4.44 mmol, 89%). **^{1}H NMR** (400 MHz, $CDCl_3$) δ 8.11 – 8.01 (m, 1H), 7.94 – 7.88 (m, 1H), 7.84 – 7.74 (m, 1H), 7.63 – 7.45 (m, 3H), 7.45 – 7.37 (m, 1H), 5.08 (sept, *J* = 6.3 Hz, 1H), 1.46 (d, *J* = 6.3 Hz, 6H). **^{13}C NMR** (100 MHz, $CDCl_3$) δ 153.4, 147.0, 134.7, 128.1, 126.9, 126.7, 126.6, 126.2, 125.5, 121.1, 117.8, 73.4, 21.9. **HRMS (ESI):** calc. for $C_{14}H_{14}NaO_3^+$ $[M+Na]^+$: 253.0835; found: 253.0835.

Isopropyl (6-(trimethylsilyl)naphthalen-2-yl) carbonate (S3)

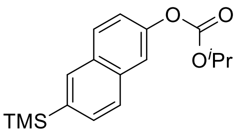

$C_{17}H_{22}O_3Si$
MW: 302,45
S3

According to **GPA** using 6-(trimethylsilyl)naphthalen-2-ol (9 mmol). Purification via flash column chromatography (SiO_2, ^{n}Hex / EtOAc, 50 / 1) afforded the title compound as a white solid (1.64 g, 5.44 mmol, 61%). **^{1}H NMR** (400 MHz, $CDCl_3$) δ 8.01 – 7.98 (m, 1H), 7.90 – 7.84 (m, 1H), 7.84 – 7.76 (m, 1H), 7.66 – 7.58 (m, 2H), 7.32 (dd, *J* = 8.9, 2.4 Hz, 1H), 5.02 (sept, *J* = 6.3 Hz, 1H), 1.41 (d, *J* = 6.3 Hz, 6H), 0.34 (s, 9H). **^{13}C NMR** (100 MHz, $CDCl_3$) δ 153.3, 149.1, 138.1, 134.0, 133.7, 131.1, 130.8, 129.7, 126.9, 120.8, 118.0, 73.3, 21.9, -1.0. **HRMS (ESI):** calc. for $C_{17}H_{22}NaO_3Si^+$ $[M+Na]^+$: 325.123; found: 325.1229.

Isopropyl (6-methoxynaphthalen-2-yl) carbonate (S4)

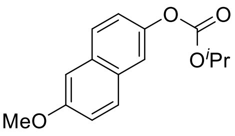

$C_{15}H_{16}O_4$
MW: 260,29
S4

According to **GPA** using 6-methoxynaphthalen-2-ol. Purification via flash column chromatography (SiO_2, ^{n}Hex / EtOAc, 10 / 1) afforded the title compound as a white solid (1.11 g, 4.25 mmol, 85%). **^{1}H NMR** (400 MHz $CDCl_3$) δ 7.79 – 7.67 (m, 2H), 7.61 – 7.56 (m, 1H), 7.28 (dd, *J* = 8.9, 2.4 Hz, 1H), 7.20 – 7.13 (m, 2H), 5.01 (sept, *J* = 6.3 Hz, 1H), 3.92 (s, 3H), 1.41 (d, *J* = 6.3 Hz, 6H). **^{13}C NMR** (100 MHz, $CDCl_3$) δ 157.8, 153.6, 147.3, 132.7, 129.2, 129.1, 128.2, 121.2, 119.7, 118.2, 105.9, 73.2, 55.5, 21.9. **HRMS (ESI):** calc. for $C_{15}H_{17}O_4^+$ $[M+H]^+$: 261.1121; found: 261.1116.

6-Benzoylnaphthalen-2-yl isopropyl carbonate (S5)

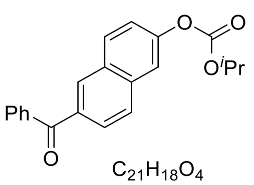

$C_{21}H_{18}O_4$
MW: 334,37
S5

According to **GPA** using (6-hydroxynaphthalen-2-yl)(phenyl)methanone (2 mmol). Purification via flash column chromatography (SiO_2, ^{n}Hex / EtOAc, 5 / 1) afforded the title compound as a white solid (621 mg, 1.86 mmol, 93%). **^{1}H NMR** (400 MHz, $CDCl_3$) δ 8.30 – 8.24 (m, 1H), 8.00 – 7.90 (m, 3H), 7.88 – 7.82 (m, 2H), 7.77 – 7.73 (m, 1H), 7.66 – 7.59 (m, 1H), 7.56 – 7.49 (m, 2H), 7.41 (dd, *J* = 8.9, 2.4 Hz, 1H), 5.04 (sept, *J* = 6.3 Hz, 1H), 1.42 (d, *J* = 6.2 Hz, 6H). **^{13}C NMR** (100 MHz, $CDCl_3$) δ 196.6, 153.1, 150.7, 137.9, 135.9, 135.0, 132.6, 131.7, 131.2, 130.4, 130.2, 128.5, 128.2, 126.8, 121.9, 118.2, 73.6, 21.9. **HRMS (ESI):** calc. for $C_{21}H_{18}NaO_4^+$ $[M+Na]^+$: 357.1097; found: 357.1089.

Benzyl 6-((isopropoxycarbonyl)oxy)-2-naphthoate (S6)

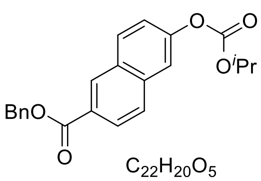

$C_{22}H_{20}O_5$
MW: 364,40
S6

According to **GPA** using benzyl 6-hydroxy-2-naphthoate. Purification via flash column chromatography (SiO_2, ^{n}Hex / EtOAc, 5 / 1) afforded the title compound as a white solid (1.35 g, 3.69 mmol, 74%). **^{1}H NMR** (500 MHz, Chloroform-*d*) δ 8.66 – 8.62 (m, 1H), 8.12 (dd, *J* = 8.6, 1.7 Hz, 1H), 8.01 – 7.94 (m, 1H), 7.90 – 7.82 (m, 1H), 7.76 – 7.68 (m, 1H), 7.54 – 7.47 (m, 2H), 7.47 – 7.34 (m, 4H), 5.43 (s, 2H), 5.03 (sept, *J* = 6.2 Hz, 1H), 1.42 (d, *J* = 6.2 Hz, 6H). **^{13}C NMR** (125 MHz, $CDCl_3$) δ 166.5, 153.1, 150.7, 136.2, 136.1, 131.1, 131.1, 130.6, 128.8, 128.5, 128.4, 128.1, 127.5, 126.3, 121.7, 118.1, 73.6, 67.1, 21.9. **HRMS (ESI):** calc. for $C_{22}H_{20}NaO_5^+$ $[M+Na]^+$: 387.1203; found: 387.1201.

Isopropyl (6-(thiophen-3-yl)naphthalen-2-yl) carbonate (S7)

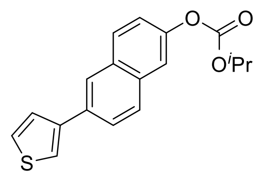

$C_{18}H_{16}O_3S$
MW: 312,38
S7

According to **GPA** using 6-(thiophen-3-yl)naphthalen-2-ol. Purification via flash column chromatography (SiO_2, ^{n}Hex / EtOAc, 20 / 1) afforded the title compound as a white solid (1.20 g, 3.85 mmol, 77%). **^{1}H NMR** (500 MHz, $CDCl_3$) δ 8.07 – 8.02 (m, 1H), 7.90 – 7.82 (m, 2H), 7.77 (dd, *J* = 8.5, 1.8 Hz, 1H), 7.65 (d, *J* = 2.4 Hz, 1H), 7.58 (dd, *J* = 2.9, 1.3 Hz, 1H), 7.52 (dd, *J* = 5.0, 1.3 Hz, 1H), 7.44 (dd, *J* = 5.0, 2.9 Hz, 1H), 7.33 (dd, *J* = 8.9, 2.4 Hz, 1H), 5.03 (sept, *J* = 6.3 Hz, 1H), 1.42 (d, *J* = 6.3 Hz, 6H). **^{13}C NMR** (125 MHz, $CDCl_3$) δ 153.4, 148.9, 142.1, 133.4, 132.9, 131.9, 129.7, 128.4, 126.6, 126.5, 126.0, 124.7, 121.3, 120.9, 118.1, 73.4, 21.9. **HRMS (ESI):** calc. for $C_{18}H_{17}O_3S^+$ $[M+H]^+$: 313.0893; found: 313.089.

Isopropyl quinolin-6-yl carbonate (S8)

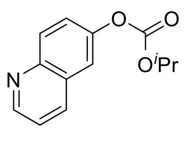

$C_{13}H_{13}NO_3$
MW: 231,25
S8

According to **GPA** using quinolin-6-ol. Purification via flash column chromatography (SiO_2, ^{n}Hex / EtOAc, 2 / 1) afforded the title compound as a white solid (1.08 g, 4.65 mmol, 93%). **^{1}H NMR** (500 MHz, $CDCl_3$) δ 8.91 (dd, *J* = 4.2, 1.7 Hz, 1H), 8.17 – 8.09 (m, 2H), 7.69 – 7.63 (m, 1H), 7.55 (dd, *J* = 9.1, 2.6 Hz, 1H), 7.41 (dd, *J* = 8.3, 4.2 Hz, 1H), 5.02 (sept, *J* = 6.3 Hz, 1H), 1.41 (d, *J* = 6.3 Hz, 6H). **^{13}C NMR** (125 MHz, $CDCl_3$) δ 153.1, 150.5, 149.0, 146.4, 136.0, 131.3, 128.6, 124.4, 121.8, 118.0, 73.6, 21.9. **HRMS (ESI):** calc. for $C_{13}H_{14}NO_3^+$ $[M+H]^+$: 232.0968; found: 232.0962.

Isopropyl phenanthren-9-yl carbonate (S9)

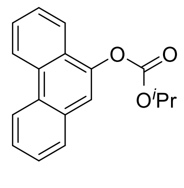

$C_{18}H_{18}O_3$
MW: 282,34
S9

According to **GPA** using phenanthren-9-ol. Purification via flash column chromatography (SiO_2, ^{n}Hex / EtOAc, 20 / 1) afforded the title compound as a white solid (861 mg, 3.05 mmol, 61%). **^{1}H NMR** (400 MHz, $CDCl_3$) δ 8.75 – 8.64 (m, 2H), 8.17 – 8.10 (m, 1H), 7.96 – 7.86 (m, 1H), 7.77 – 7.58 (m, 5H), 5.13 (sept, *J* = 6.3 Hz, 1H), 1.49 (d, *J* = 6.3 Hz, 6H). **^{13}C NMR** (100 MHz, $CDCl_3$) δ 153.4, 145.4, 131.7, 131.6, 129.1, 128.7, 127.4, 127.2, 127.1, 126.7, 126.6, 123.1, 122.8, 121.9, 117.4, 73.5, 21.9. **HRMS (ESI):** calc. for $C_{18}H_{18}NaO_3^+$ $[M+Na]^+$: 303.0992; found: 303.0987.

[1,1'-Biphenyl]-2-yl isopropyl carbonate (S10)

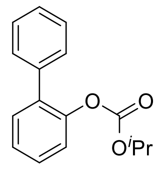

$C_{16}H_{16}O_3$
MW: 256,30
S10

According to **GPA** using [1,1'-biphenyl]-2-ol. Purification via flash column chromatography (SiO_2, ^{n}Hex / EtOAc, 20 / 1) afforded the title compound as a colorless liquid (1.27 g, 4.94 mmol, 99%). **^{1}H NMR** (400 MHz, $CDCl_3$) δ 7.52 – 7.32 (m, 8H), 7.26 – 7.22 (m, 1H), 4.84 – 4.70 (m, 1H), 1.21 – 1.14 (m, 6H). **^{13}C NMR** (100 MHz, $CDCl_3$) δ 152.8, 148.3, 137.4, 135.0, 131.0, 129.0, 128.7, 128.4, 127.6, 126.7, 122.4, 72.9, 21.5. **HRMS (ESI):** calc. for $C_{16}H_{16}NaO_3^+$ $[M+Na]^+$: 279.0992; found: 279.099.

[1,1'-Biphenyl]-3-yl isopropyl carbonate (S11)

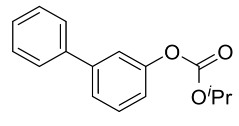

$C_{16}H_{16}O_3$
MW: 256,30
S11

According to **GPA** using [1,1'-biphenyl]-3-ol. Purification via flash column chromatography (SiO_2, ^{n}Hex / EtOAc, 20 / 1) afforded the title compound as a white solid (882 mg, 3.44 mmol, 69%). **^{1}H NMR** (400 MHz, $CDCl_3$) δ 7.68 – 7.56 (m, 2H), 7.54 – 7.26 (m, 6H), 7.27 – 7.19 (m, 1H), 5.05 (sept, *J* = 6.2 Hz, 1H), 1.44 (d, *J* = 6.3 Hz, 6H). **^{13}C NMR** (100 MHz, $CDCl_3$) δ 153.3, 151.6, 143.0, 140.2, 129.9, 129.0, 127.8, 127.3, 124.8, 120.0, 120.0, 73.3, 21.9. **HRMS (ESI):** calc. for $C_{16}H_{16}NaO_3^+$ $[M+Na]^+$: 279.0992; found: 279.0988.

[1,1'-Biphenyl]-4-yl isopropyl carbonate (S12)

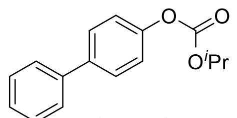

$C_{16}H_{16}O_3$
MW: 256,30
S12

According to **GPA** using [1,1'-biphenyl]-4-ol. Purification via flash column chromatography (SiO_2, ^{n}Hex / EtOAc, 20 / 1) afforded the title compound as a white solid (1.08 g, 4.22 mmol, 84%). **^{1}H NMR** (400 MHz, $CDCl_3$) δ 7.63 – 7.54 (m, 4H), 7.50 – 7.41 (m, 2H), 7.41 – 7.32 (m, 1H), 7.30 – 7.23 (m, 2H), 5.01 (sept, *J* = 6.3 Hz, 1H), 1.41 (d, *J* = 6.3 Hz, 6H). **^{13}C NMR** (100 MHz, $CDCl_3$) δ 153.3, 150.7, 140.4, 139.2, 128.9, 128.3, 127.5, 127.3, 121.5, 73.3, 21.9. **HRMS (ESI):** calc. for $C_{16}H_{16}NaO_3^+$ $[M+Na]^+$: 279.0992; found: 279.0984.

Isopropyl (naphthalen-1-ylmethyl) carbonate (S13)

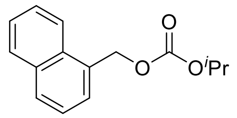

$C_{15}H_{16}O_3$
MW: 244,29
S13

According to **GPA** using naphthalen-1-ylmethanol (3.3 mmol). Purification via flash column chromatography (SiO_2, ^{n}Hex / EtOAc, 20 / 1) afforded the title compound as a colorless liquid (709 mg, 2.90 mmol, 87%). **^{1}H NMR** (500 MHz, $CDCl_3$) δ 8.13 – 8.07 (m, 1H), 7.93 – 7.85 (m, 2H), 7.63 – 7.52 (m, 3H), 7.50 – 7.45 (m, 1H), 5.66 (s, 2H), 4.96 (sept, *J* = 6.3 Hz, 1H), 1.33 (d, *J* = 6.3 Hz, 6H). **^{13}C NMR** (125 MHz, $CDCl_3$) δ 154.8, 133.8, 131.6, 131.0, 129.5, 128.7, 127.6, 126.7, 126.0, 125.3, 123.6, 72.2, 67.5, 21.8. **HRMS (ESI):** calc. for $C_{15}H_{16}NaO_3^+$ $[M+Na]^+$: 267.0992; found: 267.0987.

Isopropyl (1-(naphthalen-2-yl)ethyl) carbonate (S14)

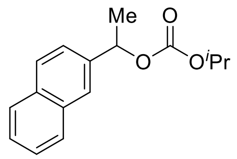

$C_{16}H_{18}O_3$
MW: 258,32
S14

According to **GPA** using 1-(naphthalen-2-yl)ethan-1-ol. Purification via flash column chromatography (SiO_2, ^{n}Hex / EtOAc, 20 / 1) afforded the title compound as a white solid (1.23 g, 4.76 mmol, 95%). **^{1}H NMR** (500 MHz, $CDCl_3$) δ 7.88 – 7.80 (m, 4H), 7.53 – 7.44 (m, 3H), 5.91 – 5.85 (m, 1H), 4.86 (sept, *J* = 6.2 Hz, 1H), 1.68 (d, *J* = 6.6 Hz, 3H), 1.32 (d, *J* = 6.2 Hz, 3H), 1.25 (d, *J* = 6.2 Hz, 3H). **^{13}C NMR** (125 MHz, $CDCl_3$) δ 154.2, 138.8, 133.3, 133.3, 128.6, 128.2, 127.8, 126.4, 126.3, 125.2, 124.1, 76.3, 72.1, 22.6, 21.9. **HRMS (ESI):** calc. for $C_{16}H_{18}NaO_3^+$ $[M+Na]^+$: 281.1148; found: 281.115.

Benzhydryl isopropyl carbonate (S15)

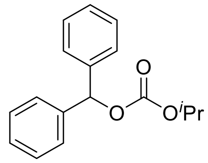

$C_{17}H_{18}O_3$
MW: 270,33
S15

According to **GPA** using diphenylmethanol. Purification via flash column chromatography (SiO_2, ^{n}Hex / EtOAc, 100 / 1 *to* 50 / 1) afforded the title compound as a white solid (457 mg, 1.69 mmol, 34%). **^{1}H NMR** (500 MHz, $CDCl_3$) δ 7.44 – 7.30 (m, 10H), 6.74 (s, 1H), 4.92 (sept, *J* = 6.2 Hz, 1H), 1.33 (d, *J* = 6.3 Hz, 6H). **^{13}C NMR** (125 MHz, $CDCl_3$) δ 154.2, 140.0, 128.6, 128.1, 127.1, 80.5, 72.4, 21.9. **HRMS (ESI):** calc. for. $C_{17}H_{18}NaO_3^+$ $[M+Na]^+$: 293.1148; found: 293.1141.

Isopropyl (4-methoxyphenyl) carbonate (S16)

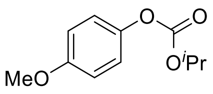

$C_{11}H_{14}O_4$
MW: 210,23
S16

According to **GPA** using 4-methoxyphenol. Purification via flash column chromatography (SiO_2, ^{n}Hex / EtOAc, 20 / 1) afforded the title compound as a white solid (909 mg, 4.32 mmol, 86%). **^{1}H NMR** (400 MHz, $CDCl_3$) δ 7.13 – 7.06 (m, 2H), 6.92 – 6.85 (m, 2H), 4.96 (sept, *J* = 6.2 Hz, 1H), 3.79 (s, 3H), 1.37 (d, *J* = 6.3 Hz, 6H). **^{13}C NMR** (100 MHz, $CDCl_3$) δ 157.4, 153.7, 144.8, 122.1, 114.5, 73.0, 55.7, 21.8. **HRMS (ESI):** calc. for $C_{11}H_{15}O_4^+$ $[M+H]^+$: 211.0965; found: 211.0963.

4-Acetylphenyl isopropyl carbonate (S17)

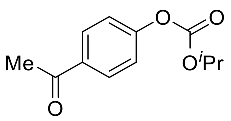

$C_{12}H_{14}O_4$
MW: 222,24
S17

According to **GPA** using 1-(4-hydroxyphenyl)ethan-1-one. Purification via flash column chromatography (SiO_2, ^{n}Hex / EtOAc, 10 / 1) afforded the title compound as a white solid (698 mg, 3.13 mmol, 63%). **^{1}H NMR** (400 MHz, $CDCl_3$) δ 8.05 – 7.91 (m, 2H), 7.32 – 7.27 (m, 2H), 5.00 (sept, *J* = 6.3 Hz, 1H), 2.60 (s, 3H), 1.39 (d, *J* = 6.3 Hz, 6H). **^{13}C NMR** (100 MHz, $CDCl_3$) δ 196.9, 154.8, 152.5, 134.8, 130.1, 121.3, 73.7, 26.7, 21.8. **HRMS (ESI):** calc. for $C_{12}H_{14}NaO_4^+$ $[M+Na]^+$: 245.0784; found: 245.0789.

Methyl 4-((isopropoxycarbonyl)oxy)benzoate (S18)

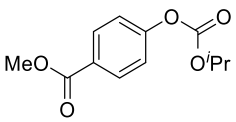

$C_{12}H_{14}O_5$
MW: 238,24
S18

According to **GPA** using methyl 4-hydroxybenzoate. Purification via flash column chromatography (SiO_2, ^{n}Hex / EtOAc, 10 / 1) afforded the title compound as a white solid (1.02 g, 4.28 mmol, 86%). **^{1}H NMR** (400 MHz, $CDCl_3$) δ 8.10 – 8.04 (m, 2H), 7.30 – 7.24 (m, 2H), 4.99 (sept, *J* = 6.3 Hz, 1H), 3.92 (s, 3H), 1.39 (d, *J* = 6.3 Hz, 6H). **^{13}C NMR** (125 MHz, $CDCl_3$) δ 166.4, 154.8, 152.6, 131.3, 127.9, 121.2, 73.6, 52.3, 21.8. **HRMS (ESI):** calc. for $C_{12}H_{14}NaO_5^+$ $[M+Na]^+$: 261.0733; found: 261.0728.

4-(Benzyloxy)phenyl isopropyl carbonate (S19)

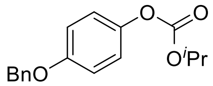

$C_{17}H_{18}O_4$
MW: 286,33
S19

According to **GPA** using 4-(phenoxymethyl)phenol. Purification via flash column chromatography (SiO_2, ^{n}Hex / EtOAc, 10 / 1) afforded the title compound as a white solid (1.11 g, 3.88 mmol, 78%). **^{1}H NMR** (500 MHz, $CDCl_3$) δ 7.46 – 7.30 (m, 5H), 7.13 – 7.06 (m, 2H), 7.00 – 6.91 (m, 2H), 5.05 (s, 2H), 5.01 – 4.92 (m, 1H), 1.38 (d, *J* = 6.3 Hz, 6H). **^{13}C NMR** (125 MHz, $CDCl_3$) δ 156.6, 153.7, 145.1, 136.9, 128.8, 128.2, 127.6, 122.1, 115.6, 73.1, 70.6, 21.9. **HRMS (ESI):** calc. for $C_{17}H_{18}NaO_4^+$ $[M+Na]^+$: 309.1097; found: 309.1094.

Benzo[*d*][1,3]dioxol-5-yl isopropyl carbonate (S20)

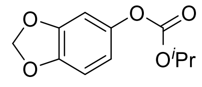

$C_{11}H_{12}O_5$
MW: 224,21
S20

According to **GPA** using benzo[*d*][1,3]dioxol-5-ol. Purification via flash column chromatography (SiO_2, ^{n}Hex / EtOAc, 10 / 1) afforded the title compound as a white solid (997 mg, 4.45 mmol, 89%). **^{1}H NMR** (500 MHz, $CDCl_3$) δ 6.76 (dd, *J* = 8.4, 0.3 Hz, 1H), 6.70 (dd, *J* = 2.4, 0.3 Hz, 1H), 6.62 (dd, *J* = 8.4, 2.4 Hz, 1H), 5.98 (s, 2H), 4.96 (sept, *J* = 6.3 Hz, 1H), 1.37 (d, *J* = 6.2 Hz, 6H). **^{13}C NMR** (125 MHz, $CDCl_3$) δ 153.5, 148.1, 145.7, 145.5, 113.8, 108.0, 103.6, 101.9, 73.2, 21.8. **HRMS (ESI):** calc. for $C_{11}H_{12}NaO_5^+$ $[M+Na]^+$: 247.0577; found: 247.0578.

3,4-Dihydronaphthalen-1-yl isopropyl carbonate (S21)

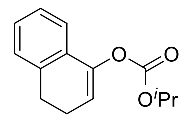

$C_{14}H_{16}O_3$
MW: 232,28
S21

Adapted from a reported procedure.[6] A solution of 3,4-dihydronaphthalen-1(2*H*)-one (0.67 mL, 5.0 mmol, 1.0 equiv.) and TMEDA (0.8 mL, 5.5 mmol, 1.1 equiv.) in THF (5 mL) was added dropwise to a solution of KHMDS (1 M in THF, 7.5 mL, 7.5 mmol, 1.5 equiv.) in THF (10 mL) at -78 °C. The resulting mixture was stirred for 1 h at -78 °C. Thereafter, isopropyl chloroformate (1 M in PhMe, 5.5 mL, 5.5 mmol, 1.1 equiv.) was added dropwise. Upon complete addition the reaction was stirred for further 30 min at -78 °C before being quenched by the addition of sat. aq. NH_4Cl and diluted with Et_2O. The layers were seperated and the aq. layer extracted twice with Et_2O. The combined org. layers were dried over Na_2SO_4, filtered and the solvent was removed under reduced pressure. Purification via flash column chromatography (SiO_2, ^{n}Hex / EtOAc, 20 / 1) afforded the title compound as a yellow liquid (783 mg, 3.37 mmol, 67%). **^{1}H NMR** (400 MHz, $CDCl_3$) δ 7.23 – 7.14 (m, 4H), 5.82 (t, *J* = 4.7 Hz, 1H), 4.96 (sept, *J* = 6.3 Hz, 1H), 2.92 – 2.82 (m, 2H), 2.46 (ddd, *J* = 9.0, 7.6, 4.7 Hz, 2H), 1.38 (d, *J* = 6.3 Hz, 6H). **^{13}C NMR** (100 MHz, $CDCl_3$) δ 153.1, 146.1, 136.4, 130.4, 128.1, 127.6, 126.5, 120.6, 115.2, 72.9, 27.4, 22.0, 21.8. **HRMS (ESI):** calc. for $C_{14}H_{16}NaO_3^+$ $[M+Na]^+$: 255.0992; found: 255.0997.

1-Benzyl-1,2,3,6-tetrahydropyridin-4-yl isopropyl carbonate (S22)

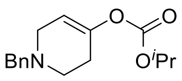

$C_{16}H_{21}NO_3$
MW: 275,35
S22

Adapted from a reported procedure.[6] A solution of 1-benzylpiperidin-4-one (0.93 mL, 5.0 mmol, 1.0 equiv.) and TMEDA (0.8 mL, 5.5 mmol, 1.1 equiv.) in THF (5 mL) was added dropwise to a solution of KHMDS (1 M in THF, 7.5 mL, 7.5 mmol, 1.5 equiv.) in THF (10 mL) at -78 °C. The resulting mixture was stirred for 1 h at -78 °C. Thereafter, isopropyl chloroformate (1 M in PhMe, 5.5 mL, 5.5 mmol, 1.1 equiv.) was added dropwise. Upon complete addition the reaction was stirred for further 30 min at -78 °C before being quenched by the addition of sat. aq. NH_4Cl and diluted with Et_2O. The layers were seperated and the aq. layer extracted twice with Et_2O. The combined org. layers were dried over Na_2SO_4, filtered and the solvent was removed under reduced pressure. Purification via flash column chromatography (SiO_2, ^{n}Hex / EtOAc, 5 / 1) afforded the title compound as a yellow liquid (719 mg, 2.61 mmol, 52%). **^{1}H NMR** (400 MHz, $CDCl_3$) δ 7.38 – 7.28 (m, 4H), 7.29 – 7.22 (m, 1H), 5.56 – 5.40 (m, 1H), 4.89 (sept, *J* = 6.3 Hz, 1H), 3.62 (s, 2H), 3.12 – 3.06 (m, 2H), 2.70 (t, *J* = 5.8 Hz, 2H), 2.39 – 2.30 (m, 2H), 1.32 (d, *J* = 6.3 Hz, 6H). **^{13}C NMR** (100 MHz, $CDCl_3$) δ 152.7, 147.0, 138.3, 129.1, 128.3, 127.2, 111.7, 72.5, 61.8, 50.8, 49.3, 27.3, 21.8. **HRMS (ESI):** calc. for $C_{16}H_{22}NO_3^+$ $[M+H]^+$: 276.1594; found: 276.1595.

Isopropyl (3-oxocyclohex-1-en-1-yl) carbonate (S23)

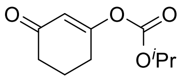

$C_{10}H_{14}O_4$
MW: 198,22
S23

Adapted from a reported procedure.[7] Isopropyl chloroformate (1 M in PhMe, 5.5 mL, 5.5 mmol, 1.1 equiv.) was added dropwise to a suspension of cyclohexane-1,3-dione (561 mg, 5.0 mmol, 1.0 equiv.) and K_2CO_3 (1.38 g, 10 mmol, 2.0 equiv.) in acetone (10 mL) at 0 °C. Upon complete addition the cooling was removed and the reaction was stirred for 18 h at room temperature before being by the addition of aq. HCl (1 M) and diluted with EtOAc. The layers were seperated and the aq. layer extracted with EtOAc. The combined org. layers were washed with sat. aq. $NaHCO_3$ and brine. The org. layer was dried over Na_2SO_4, filtered and the solvent was removed under reduced pressure. Purification via flash column chromatography (SiO_2, ^{n}Hex / EtOAc, 2 / 1) afforded the title compound as a colorless liquid (719 mg, 3.63 mmol, 73%). **^{1}H NMR** (500 MHz, $CDCl_3$) δ 5.99 (t, *J* = 1.3 Hz, 1H), 4.90 (sept, *J* = 6.3 Hz, 1H), 2.53 (td, *J* = 6.3, 1.3 Hz, 2H), 2.40 – 2.31 (m, 2H), 2.07 – 1.97 (m, 2H), 1.31 (d, *J* = 6.3 Hz, 6H). **^{13}C NMR** (125 MHz, $CDCl_3$) δ 199.4, 169.4, 150.5, 116.3, 73.9, 36.7, 28.0, 21.6, 21.2. **HRMS (ESI):** calc. for $C_{10}H_{14}NaO_4^+$ $[M+Na]^+$: 221.0784; found: 221.0786.

Methyl 2-((isopropoxycarbonyl)oxy)cyclohex-1-ene-1-carboxylate (S24)

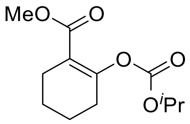

$C_{12}H_{18}O_5$
MW: 242,27
S24

Adapted from a reported procedure.[7] Methyl 2-oxocyclohexane-1-carboxylate (1.0 g, 6.4 mmol, 1.0 equiv.) was dissolved in THF (10 mL) and added dropwise to a suspension of NaH (60 wt% in mineral oil, 384 mg, 9.6 mmol, 1.5 equiv.) in THF (10 mL) at 0 °C. The resulting mixture was stirred for 20 min before isopropyl chloroformate (1 M in PhMe, 7.1 mL, 7.1 mmol, 1.1 equiv.) was added dropwise at 0 °C. Upon complete addition the cooling was removed and the reaction was stirred for 3 h at room temperature before being by the addition of aq. HCl (1 M) and diluted with EtOAc. The layers were seperated and the aq. layer extracted with EtOAc. The combined org. layers were washed with sat. aq. $NaHCO_3$ and brine. The org. layer was dried over Na_2SO_4, filtered and the solvent was removed under reduced pressure. Purification via flash column chromatography (SiO_2, ^{n}Hex / EtOAc, 10 / 1) afforded the title compound as a colorless liquid (1.3 g, 5.34 mmol, 84%). **^{1}H NMR** (400 MHz, $CDCl_3$) δ 4.87 (sept, *J* = 6.3 Hz, 1H), 3.66 (s, 3H), 2.41 – 2.30 (m, 2H), 2.30 – 2.20 (m, 2H), 1.76 – 1.66 (m, 2H), 1.66 – 1.55 (m, 2H), 1.31 (d, *J* = 6.3 Hz, 6H). **^{13}C NMR** (100 MHz, $CDCl_3$) δ 166.0, 155.3, 151.9, 117.8, 72.8, 51.6, 28.8, 25.2, 22.0, 21.7, 21.6. **HRMS (ESI):** calc. for $C_{12}H_{18}NaO_5^+$ $[M+Na]^+$: 265.1046; found: 265.1046.

Isopropyl (3-phenylprop-1-en-1-yl) carbonate (S25)

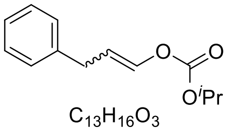

$C_{13}H_{16}O_3$
MW: 220,27
S25

Adapted from a reported procedure.[8] Isopropyl chloroformate (1 M in PhMe, 5.5 mL, 5.5 mmol, 1.1 equiv.) was added to a mixture of 3-phenylpropanal (0.67 mL, 5.0 mmol, 1.0 equiv.), KF (1.5 g, 25 mmol, 5.0 equiv.) and 18-Crown-6 (265 mg, 1.0 mmol, 20 mol%) in MeCN (20 mL). The reaction was stirred at 70 °C for 24 h. Thereafter water was added and the mixture was extracted with DCM, dried over $MgSO_4$, filtered and the solvent was removed under reduced pressure. Purification via flash column chromatography (SiO_2, ^{n}Hex / EtOAc, 20 / 1) afforded the title compound as a colorless liquid and as an inconsequential 1 : 2 mixture of *E* : *Z* isomers (612 mg, 2.78 mmol, 56%). *Z-isomer:* **^{1}H NMR** (400 MHz, $CDCl_3$) δ 7.35 – 7.28 (m, 2H), 7.26 – 7.19 (m, 3H), 7.05 – 6.99 (m, 1H), 5.08 (dt, *J* = 7.6, 6.3 Hz, 1H), 5.03 – 4.93 (m, 1H), 3.58 – 3.51 (m, 2H), 1.38 (d, *J* = 6.3 Hz, 6H). **^{13}C NMR** (101 MHz, $CDCl_3$) δ 152.5, 140.1, 136.1, 128.6, 128.4, 126.2, 112.3, 72.9, 30.6, 21.8. *E-isomer:* **^{1}H NMR** (400 MHz, $CDCl_3$) δ 7.35 – 7.28 (m, 2H), 7.26 – 7.19 (m, 3H), 7.05 – 6.99 (m, 1H), 5.62 (dt, *J* = 12.3, 7.6 Hz, 1H), 5.03 – 4.93 (m, 1H), 3.40 – 3.32 (m, 2H), 1.35 (d, *J* = 6.3 Hz, 6H). **^{13}C NMR** (100 MHz, $CDCl_3$) δ 152.4, 139.7, 137.8, 128.6, 128.4, 126.4, 113.5, 72.8, 33.4, 21.8. **HRMS (ESI):** calc. for $C_{13}H_{16}NaO_3^+$ $[M+Na]^+$: 243.0992; found: 243.0991.

Cyclododecyl naphthalen-2-yl carbonate (S26)

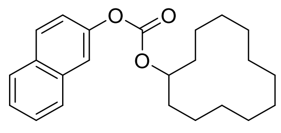

$C_{23}H_{30}O_3$
MW: 354,49
S26

According to **GPB** using cyclododecanol (5 mmol). Purification via flash column chromatography (SiO_2, ^{n}Hex / EtOAc, 50 / 1) afforded the title compound as a white solid (1.68 g, 4.73 mmol, 95%). **^{1}H NMR** (400 MHz, $CDCl_3$) δ 7.90 – 7.78 (m, 3H), 7.67 (d, *J* = 2.4 Hz, 1H), 7.56 – 7.43 (m, 2H), 7.33 (dd, *J* = 8.9, 2.4 Hz, 1H), 5.05 – 4.93 (m, 1H), 1.92 – 1.79 (m, 2H), 1.79 – 1.65 (m, 2H), 1.60 – 1.24 (m, 18H). **^{13}C NMR** (100 MHz, $CDCl_3$) δ 153.8, 149.0, 133.8, 131.6, 129.6, 127.9, 127.9, 126.7, 125.9, 120.8, 118.2, 78.1, 29.3, 24.1, 23.9, 23.5, 23.3, 21.1. **HRMS (ESI):** calc. for $C_{23}H_{30}NaO_3^+$ $[M+Na]^+$: 377.2087; found: 377.2091.

Cyclopentyl naphthalen-2-yl carbonate (S27)

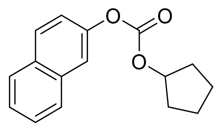

$C_{16}H_{16}O_3$
MW: 256,30
S27

According to **GPB** using cyclopentanol. Purification via flash column chromatography (SiO_2, ^{n}Hex / EtOAc, 75 / 1) afforded the title compound as a white solid (411 mg, 1.60 mmol, 64%). **^{1}H NMR** (400 MHz, $CDCl_3$) δ 7.90 – 7.79 (m, 3H), 7.68 – 7.65 (m, 1H), 7.55 – 7.43 (m, 2H), 7.33 (dd, *J* = 8.9, 2.4 Hz, 1H), 5.27 – 5.22 (m, 1H), 2.00 – 1.91 (m, 4H), 1.89 – 1.77 (m, 2H), 1.72 – 1.61 (m, 2H). **^{13}C NMR** (100 MHz, $CDCl_3$) δ 153.6, 148.9, 133.8, 131.6, 129.6, 127.9, 127.8, 126.7, 125.9, 120.8, 118.2, 82.3, 32.8, 23.7. **HRMS (ESI):** calc. for $C_{16}H_{16}NaO_3^+$ $[M+Na]^+$: 279.0992; found: 279.0993.

2,4-Dimethylpentan-3-yl naphthalen-2-yl carbonate (S28)

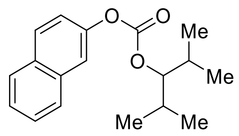

$C_{18}H_{22}O_3$
MW: 286,37
S28

According to **GPB** using 2,4-dimethylpentan-3-ol. Purification via flash column chromatography (SiO_2, ^{n}Hex / EtOAc, 50 / 1) afforded the title compound as a colorless liquid (695 mg, 2.43 mmol, 97%). **^{1}H NMR** (500 MHz, $CDCl_3$) δ 7.90 – 7.80 (m, 3H), 7.68 – 7.64 (m, 1H), 7.54 – 7.44 (m, 2H), 7.32 (dd, *J* = 8.9, 2.4 Hz, 1H), 4.53 (t, *J* = 6.2 Hz, 1H), 2.11 – 1.96 (m, 2H), 1.02 (dd, *J* = 6.8, 1.1 Hz, 12H). **^{13}C NMR** (125 MHz, $CDCl_3$) δ 154.6, 149.1, 133.8, 131.6, 129.6, 127.9, 127.8, 126.7, 125.9, 120.9, 118.1, 89.0, 29.7, 19.6, 17.3. **HRMS (ESI):** calc. for $C_{18}H_{22}NaO_3^+$ $[M+Na]^+$: 309.1461; found: 309.1462.

Naphthalen-2-yl ((1*R*,2*R*,4*S*)-1,3,3-trimethylbicyclo[2.2.1]heptan-2-yl) carbonate (S29)

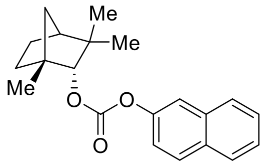

$C_{21}H_{24}O_3$
MW: 324,42
S29

According to **GPB** using (1*R*,2*R*,4*S*)-1,3,3-trimethylbicyclo[2.2.1]heptan-2-ol. Purification via flash column chromatography (SiO_2, ^{n}Hex / EtOAc, 75 / 1) afforded the title compound as an off-white solid (802 mg, 2.47 mmol, 99%). **^{1}H NMR** (500 MHz, $CDCl_3$) δ 7.90 – 7.78 (m, 3H), 7.68 – 7.64 (m, 1H), 7.54 – 7.44 (m, 2H), 7.33 (dd, *J* = 8.9, 2.4 Hz, 1H), 4.37 (d, *J* = 1.9 Hz, 1H), 1.85 – 1.72 (m, 3H), 1.65 – 1.59 (m, 1H), 1.54 – 1.46 (m, 1H), 1.26 – 1.22 (m, 1H), 1.18 (d, *J* = 11.2 Hz, 6H), 1.16 – 1.09 (m, 1H), 0.98 (s, 3H). **^{13}C NMR** (125 MHz, $CDCl_3$) δ 154.6, 149.0, 133.8, 131.6, 129.6, 127.9, 127.8, 126.8, 125.9, 120.8, 118.2, 91.2, 48.6, 48.5, 41.4, 39.9, 29.9, 26.6, 25.9, 20.1, 19.6. **HRMS (ESI):** calc. for $C_{21}H_{24}NaO_3^+$ $[M+Na]^+$: 347.1618; found: 347.1617.

Bis(4-fluorophenyl)methyl naphthalen-2-yl carbonate (S30)

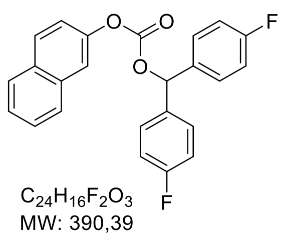

$C_{24}H_{16}F_2O_3$
MW: 390,39
S30

According to **GPB** using bis(4-fluorophenyl)methanol. Purification via flash column chromatography (SiO_2, ^{n}Hex / EtOAc, 50 / 1) afforded the title compound as a white solid (379 mg, 0.97 mmol, 39%). **^{1}H NMR** (400 MHz, $CDCl_3$) δ 7.89 – 7.76 (m, 3H), 7.67 – 7.59 (m, 1H), 7.54 – 7.44 (m, 2H), 7.44 – 7.36 (m, 4H), 7.29 (dd, *J* = 8.9, 2.4 Hz, 1H), 7.13 – 7.04 (m, 4H), 6.79 (s, 1H). **^{13}C NMR** (125 MHz, $CDCl_3$) δ 163.8, 161.8, 153.2, 148.7, 135.1 (d, *J* = 3.2 Hz), 133.7, 131.7, 129.7, 129.1 (d, *J* = 8.3 Hz), 127.9 (d, *J* = 5.8 Hz), 126.9, 126.1, 120.5, 118.1, 115.9 (d, *J* = 21.7 Hz), 80.4. **^{19}F NMR** (471 MHz, $CDCl_3$) δ -113.2. **HRMS (ESI):** calc. for $C_{24}H_{16}F_2NaO_3^+$ $[M+Na]^+$: 413.096; found: 413.0957.

Dodecan-2-yl naphthalen-2-yl carbonate (S31)

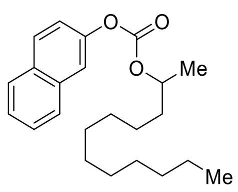

$C_{23}H_{32}O_3$
MW: 356,51
S31

According to **GPB** using dodecan-2-ol. Purification via flash column chromatography (SiO_2, ^{n}Hex / EtOAc, 100 / 1) afforded the title compound as a colorless liquid (859 mg, 2.41 mmol, 96%). **^{1}H NMR** (400 MHz, $CDCl_3$) δ 7.90 – 7.80 (m, 3H), 7.69 (d, *J* = 2.4 Hz, 1H), 7.55 – 7.44 (m, 2H), 7.36 (dd, *J* = 8.9, 2.4 Hz, 1H), 4.98 – 4.88 (m, 1H), 1.86 – 1.73 (m, 1H), 1.70 – 1.57 (m, 1H), 1.52 – 1.25 (m, 19H), 0.98 – 0.87 (m, 3H). **^{13}C NMR** (100 MHz, $CDCl_3$) δ 153.6, 148.9, 133.8, 131.5, 129.5, 127.8, 127.8, 126.7, 125.8, 120.8, 118.1, 76.8, 36.0, 32.0, 29.7, 29.7, 29.6, 29.5, 29.5, 25.4, 22.8, 20.0, 14.2. **HRMS (ESI):** calc. for $C_{23}H_{36}NO_3^+$ $[M+NH_4]^+$: 374.269; found: 374.2688.

Adamantan-2-yl naphthalen-2-yl carbonate (S32)

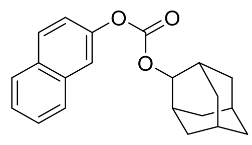

$C_{21}H_{22}O_3$
MW: 322,40
S32

According to **GPB** using adamantan-2-ol. Purification via flash column chromatography (SiO_2, ^{n}Hex / EtOAc, 100 / 1) afforded the title compound as a white solid (315 mg, 0.98 mmol, 39%). **^{1}H NMR** (500 MHz, $CDCl_3$) δ 7.90 – 7.78 (m, 3H), 7.73 – 7.64 (m, 1H), 7.55 – 7.44 (m, 2H), 7.35 (dd, *J* = 8.9, 2.4 Hz, 1H), 4.97 – 4.93 (m, 1H), 2.26 – 2.19 (m, 2H), 2.17 – 2.10 (m, 2H), 1.97 – 1.86 (m, 4H), 1.84 – 1.76 (m, 4H), 1.68 – 1.59 (m, 2H). **^{13}C NMR** (125 MHz, $CDCl_3$) δ 153.4, 149.0, 133.8, 131.6, 129.5, 127.9, 127.8, 126.7, 125.9, 120.9, 118.2, 82.4, 37.4, 36.4, 32.0, 31.7, 27.2, 27.0. **HRMS (ESI):** calc. for $C_{21}H_{22}NaO_3^+$ $[M+Na]^+$: 345.1461; found: 345.146.

4,4-Difluorocyclohexyl naphthalen-2-yl carbonate (S33)

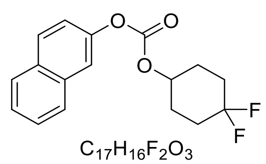

$C_{17}H_{16}F_2O_3$
MW: 306,31
S33

According to **GPB** using 4,4-difluorocyclohexan-1-ol. Purification via flash column chromatography (SiO_2, ^{n}Hex / EtOAc, 100 / 1) afforded the title compound as a white solid (737 mg, 2.41 mmol, 96%). **^{1}H NMR** (500 MHz, $CDCl_3$) δ 7.92 – 7.79 (m, 3H), 7.71 – 7.64 (m, 1H), 7.57 – 7.45 (m, 2H), 7.32 (dd, *J* = 8.9, 2.4 Hz, 1H), 5.06 – 4.88 (m, 1H), 2.27 – 1.86 (m, 8H). **^{13}C NMR** (125 MHz, $CDCl_3$) δ 153.2, 148.7, 133.8, 131.7, 129.7, 127.9, 127.9, 126.9, 126.1, 123.5 (t, *J* = 241.2 Hz), 120.6, 118.2, 73.6, 30.0 (t, *J* = 25.0 Hz), 27.4 – 27.1 (m). **^{19}F NMR** (471 MHz, $CDCl_3$) δ -94.5 (d, *J* = 241.2 Hz), -101.0 (d, *J* = 240.5 Hz). **HRMS (ESI):** calc. for $C_{17}H_{16}F_2NaO_3^+$ $[M+Na]^+$: 329.096; found: 329.0965.

Cyclohex-2-en-1-yl naphthalen-2-yl carbonate (S34)

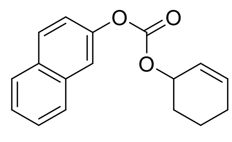

$C_{17}H_{16}O_3$
MW: 268,31
S34

According to **GPB** using cyclohex-2-en-1-ol. Purification via flash column chromatography (SiO_2, ^{n}Hex / EtOAc, 20 / 1) afforded the title compound as a white solid (407 mg, 1.52 mmol, 61%). **^{1}H NMR** (500 MHz, $CDCl_3$) δ 7.89 – 7.79 (m, 3H), 7.69 – 7.64 (m, 1H), 7.55 – 7.45 (m, 2H), 7.34 (dd, *J* = 8.9, 2.4 Hz, 1H), 6.10 – 6.03 (m, 1H), 5.94 – 5.86 (m, 1H), 5.31 – 5.23 (m, 1H), 2.20 – 2.11 (m, 1H), 2.09 – 2.01 (m, 1H), 2.01 – 1.95 (m, 2H), 1.90 – 1.79 (m, 1H), 1.75 – 1.65 (m, 1H). **^{13}C NMR** (125 MHz, $CDCl_3$) δ 153.6, 148.9, 134.1, 133.8, 131.6, 129.6, 127.9, 127.8, 126.7, 125.9, 124.6, 120.8, 118.2, 73.0, 28.3, 25.0, 18.6. **HRMS (ESI):** calc. for $C_{17}H_{16}NaO_3^+$ $[M+Na]^+$: 291.0992; found: 291.099.

Naphthalen-2-yl (1,4-dioxaspiro[4.5]decan-8-yl) carbonate (S35)

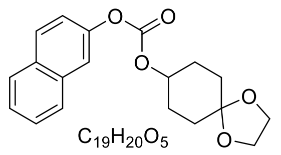

$C_{19}H_{20}O_5$
MW: 328,36
S35

According to **GPB** using 1,4-dioxaspiro[4.5]decan-8-ol. Purification via flash column chromatography (SiO_2, ^{n}Hex / EtOAc, 50 / 1) afforded the title compound as a white solid (554 mg, 1.69 mmol, 67%). **^{1}H NMR** (400 MHz, $CDCl_3$) δ 7.89 – 7.79 (m, 3H), 7.71 – 7.62 (m, 1H), 7.54 – 7.43 (m, 2H), 7.32 (dd, *J* = 8.9, 2.4 Hz, 1H), 4.89 (tt, *J* = 6.6, 4.2 Hz, 1H), 4.02 – 3.92 (m, 4H), 2.08 – 1.94 (m, 4H), 1.94 – 1.84 (m, 2H), 1.74 – 1.63 (m, 2H). **^{13}C NMR** (100 MHz, $CDCl_3$) δ 153.4, 148.9, 133.8, 131.6, 129.6, 127.9, 127.9, 126.8, 125.9, 120.7, 118.2, 107.9, 75.5, 64.5, 31.3, 28.4. **HRMS (ESI):** calc. for $C_{19}H_{20}NaO_5^+$ $[M+Na]^+$: 351.1203; found: 351.1209.

Naphthalen-2-yl (1-phenylethyl) carbonate (S36)

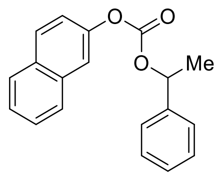

$C_{19}H_{16}O_3$
MW: 292,33
S36

According to **GPB** using 1-phenylethan-1-ol. Purification via flash column chromatography (SiO_2, ^{n}Hex / EtOAc, 75 / 1) afforded the title compound as a white solid (731 mg, 2.50 mmol, quant.). **^{1}H NMR** (500 MHz, $CDCl_3$) δ 7.87 – 7.78 (m, 3H), 7.66 – 7.64 (m, 1H), 7.50 – 7.44 (m, 4H), 7.44 – 7.39 (m, 2H), 7.39 – 7.34 (m, 1H), 7.30 (dd, *J* = 8.9, 2.4 Hz, 1H), 5.88 (q, *J* = 6.6 Hz, 1H), 1.72 (d, *J* = 6.6 Hz, 3H). **^{13}C NMR** (125 MHz, $CDCl_3$) δ 153.3, 148.8, 140.7, 133.8, 131.6, 129.6, 128.8, 128.5, 127.9, 127.8, 126.8, 126.3, 125.9, 120.7, 118.2, 77.7, 22.4. **HRMS (ESI):** calc. for $C_{19}H_{20}NO_3^+$ $[M+NH_4]^+$: 310.1438; found: 310.1439.

(3*S*,8*S*,9*S*,10*R*,13*R*,14*S*,17*R*)-10,13-dimethyl-17-((*R*)-6-methylheptan-2-yl)-2,3,4,7,8,9,10,11,12,13,14,15,16,17-tetradecahydro-1*H*-cyclopenta[*a*]phenanthren-3-yl naphthalen-2-yl carbonate (S37)

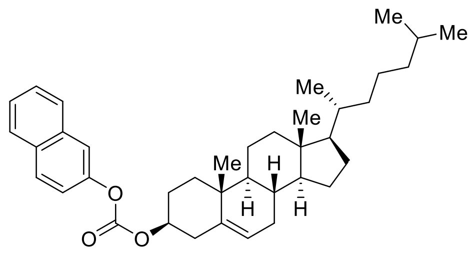

$C_{38}H_{52}O_3$
MW: 556,83
S37

According to **GPB** using cholesterol. Purification via flash column chromatography (SiO_2, ^{n}Hex / EtOAc, 50 / 1) afforded the title compound as a white solid (901 mg, 1.62 mmol, 65%). **^{1}H NMR** (500 MHz, $CDCl_3$) δ 7.88 – 7.79 (m, 3H), 7.68 – 7.65 (m, 1H), 7.54 – 7.44 (m, 2H), 7.33 (dd, *J* = 8.9, 2.4 Hz, 1H), 5.47 – 5.40 (m, 1H), 4.68 – 4.57 (m, 1H), 2.59 – 2.44 (m, 2H), 2.11 – 1.97 (m, 3H), 1.93 (dt, *J* = 13.4, 3.6 Hz, 1H), 1.90 – 1.79 (m, 1H), 1.79 – 1.72 (m, 1H), 1.64 – 1.43 (m, 6H), 1.43 – 1.30 (m, 3H), 1.30 – 1.22 (m, 1H), 1.22 – 1.06 (m, 7H), 1.06 (s, 3H), 1.05 – 0.94 (m, 3H), 0.92 (d, *J* = 6.5 Hz, 3H), 0.88 (d, *J* = 2.2 Hz, 3H), 0.86 (d, *J* = 2.3 Hz, 3H), 0.69 (s, 3H). **^{13}C NMR** (125 MHz, $CDCl_3$) δ 153.3, 148.9, 139.3, 133.8, 131.6, 129.6, 127.9, 127.9, 126.8, 125.9, 123.4, 120.8, 118.2, 79.1, 56.8, 56.3, 50.1, 42.5, 39.9, 39.7, 38.1, 37.0, 36.7, 36.3, 35.9, 32.1, 32.0, 28.4, 28.2, 27.8, 24.4, 24.0, 23.0, 22.7, 21.2, 19.4, 18.9, 12.0. **HRMS (ESI):** calc. for $C_{38}H_{52}KO_3^+$ $[M+K]^+$: 595.3548; found: 595.354.

Di-*tert*-butyl naphthalene-2,6-diyl bis(carbonate) (S38)

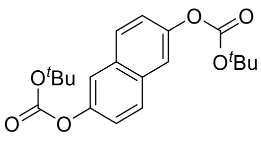

$C_{20}H_{24}O_6$
MW: 360,41
S38

NEt_3 (3.1 mL, 22 mmol, 2.2 equiv.) was added to a mixture of naphthalene-2,6-diol (1.60 g, 10 mmol, 1.0 equiv.) and DMAP (244 mg, 2.0 mmol, 0.2 equiv.) in DCM / THF (1 / 1; 40 mL). Thereafter, the mixture was cooled to 0 °C and Boc_2O (5.1 mL, 22 mmol, 2.2 equiv.) was added. The cooling was removed and the resulting mixture stirred for 48 h. After that, aq. sat. NH_4Cl was added and the layers were separated. The aq. layer was extracted with DCM. The combined org. layers were dried over $MgSO_4$, filtered and the solvent was removed under reduced pressure. Purification by flash column chromatography (SiO_2, ^{n}Hex / EtOAc, 2 / 1) afforded the title compound as a white solid (2.46 g, 6.82 mmol, 68%.). **^{1}H NMR** (500 MHz, $CDCl_3$) δ 7.81 (d, *J* = 8.8 Hz, 2H), 7.64 (d, *J* = 2.4 Hz, 2H), 7.33 (dd, *J* = 8.7, 2.6 Hz, 2H), 1.58 (s, 18H). **^{13}C NMR** (125 MHz, $CDCl_3$) δ 152.0, 148.8, 131.7, 129.2, 121.8, 118.2, 83.9, 27.9. **HRMS (ESI):** calc. for $C_{20}H_{28}NO_6^+$ $[M+NH_4]^+$: 378.1911; found: 378.1908.

Di-*tert*-butyl naphthalene-2,7-diyl bis(carbonate) (S39)

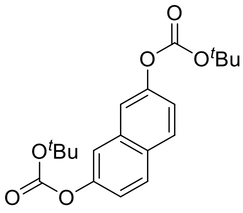

$C_{20}H_{24}O_6$
MW: 360,41
S39

NEt_3 (3.1 mL, 22 mmol, 2.2 equiv.) was added to a mixture of naphthalene-2,7-diol (1.60 g, 10 mmol, 1.0 equiv.) and DMAP (244 mg, 2.0 mmol, 0.2 equiv.) in DCM / THF (1 / 1; 40 mL). Thereafter, the mixture was cooled to 0 °C and Boc_2O (5.1 mL, 22 mmol, 2.2 equiv.) was added. The cooling was removed and the resulting mixture stirred for 48 h. After that, aq. sat. NH_4Cl was added and the layers were separated. The aq. layer was extracted with DCM. The combined org. layers were dried over $MgSO_4$, filtered and the solvent was removed under reduced pressure. Purification by flash column chromatography (SiO_2, ^{n}Hex / EtOAc, 5 / 1) afforded the title compound as a white solid (3.50 g, 9.71 mmol, 97%.). **^{1}H NMR** (400 MHz, $CDCl_3$) δ 7.86 – 7.80 (m, 2H), 7.63 – 7.58 (m, 2H), 7.30 (dd, *J* = 8.9, 2.3 Hz, 2H), 1.58 (s, 18H). **^{13}C NMR** (100 MHz, $CDCl_3$) δ 151.9, 149.5, 134.3, 129.4, 129.4, 120.9, 118.2, 83.9, 27.9. **HRMS (ESI):** calc. for $C_{20}H_{28}NO_6^+$ $[M+NH_4]^+$: 378.1911; found: 378.1912.

Di-*tert*-butyl naphthalene-1,6-diyl bis(carbonate) (S40)

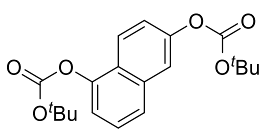

$C_{20}H_{24}O_6$
MW: 360,41
S40

NEt_3 (3.1 mL, 22 mmol, 2.2 equiv.) was added to a mixture of naphthalene-1,6-diol (1.60 g, 10 mmol, 1.0 equiv.) and DMAP (244 mg, 2.0 mmol, 0.2 equiv.) in DCM / THF (1 / 1; 40 mL). Thereafter, the mixture was cooled to 0 °C and Boc_2O (5.1 mL, 22 mmol, 2.2 equiv.) was added. The cooling was removed and the resulting mixture stirred for 48 h. After that, aq. sat. NH_4Cl was added and the layers were separated. The aq. layer was extracted with DCM. The combined org. layers were dried over $MgSO_4$, filtered and the solvent was removed under reduced pressure. Purification by flash column chromatography (SiO_2, ^{n}Hex / EtOAc, 10 / 1) afforded the title compound as a white solid (3.33 g, 9.23 mmol, 92%.). **^{1}H NMR** (500 MHz, $CDCl_3$) δ 8.02 – 7.97 (m, 1H), 7.73 – 7.66 (m, 2H), 7.47 (dd, *J* = 8.3, 7.6 Hz, 1H), 7.36 (dd, *J* = 9.1, 2.3 Hz, 1H), 7.30 (dd, *J* = 7.6, 1.0 Hz, 1H), 1.59 (m, 18H). **^{13}C NMR** (125 MHz, $CDCl_3$) δ 152.0, 151.9, 149.4, 147.1, 135.2, 126.5, 125.9, 125.0, 123.1, 121.6, 118.4, 117.8, 84.0, 83.9, 27.9, 27.8. **HRMS (ESI):** calc. for $C_{20}H_{28}NO_6^+$ $[M+NH_4]^+$: 378.1911; found: 378.1915.

Di-*tert*-butyl naphthalene-1,5-diyl bis(carbonate) (52)

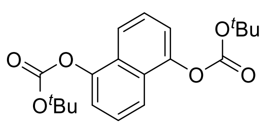

$C_{20}H_{24}O_6$
MW: 360,41
52

NEt_3 (3.1 mL, 22 mmol, 2.2 equiv.) was added to a mixture of naphthalene-1,5-diol (1.60 g, 10 mmol, 1.0 equiv.) and DMAP (244 mg, 2.0 mmol, 0.2 equiv.) in DCM / THF (1 / 1; 40 mL). Thereafter, the mixture was cooled to 0 °C and Boc_2O (5.1 mL, 22 mmol, 2.2 equiv.) was added. The cooling was removed and the resulting mixture stirred for 48 h. After that, aq. sat. NH_4Cl was added and the layers were separated. The aq. layer was extracted with DCM. The combined org. layers were dried over $MgSO_4$, filtered and the solvent was removed under reduced pressure. Purification by flash column chromatography (SiO_2, ^{n}Hex / EtOAc, 20 / 1) afforded the title compound as a white solid (2.40 g, 6.66 mmol, 67%.). **^{1}H NMR** (400 MHz, $CDCl_3$) δ 7.91 – 7.86 (m, 2H), 7.56 – 7.47 (m, 2H), 7.39 – 7.33 (m, 2H), 1.59 (s, 18H). **^{13}C NMR** (100 MHz, $CDCl_3$) δ 152.0, 147.1, 128.4, 126.2, 119.4, 118.6, 84.0, 27.9. **HRMS (ESI):** calc. for $C_{20}H_{28}NO_6^+$ $[M+NH_4]^+$: 378.1911; found: 378.1912.

Naphthalen-2-yl (propan-2-yl-1,1,1,3,3,3-d_6) carbonate (3)

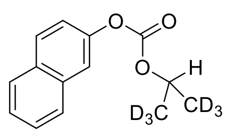

$C_{14}H_8D_6O_3$
MW: 236,30
3

Propan-1,1,1,3,3,3-d_6-2-ol (99 atom% D; 0.5 mL, 6.5 mmol, 1.3 equiv.) was added to a solution of 2-naphthyl chloroformate **S1** (1.0 g, 5.0 mmol, 1.0 equiv.) in DCM (10 mL) at 0 °C. Thereafter, pyridine (0.6 mL, 7.5 mol, 1.5 equiv.) was added dropwise. The reaction was stirred for 10 min at 0 °C before the cooling was removed and stirring continued for 24 h at room temperature. After that, water was added and the layers separated. The org. layer was dried over $MgSO_4$, filtered and the solvent removed under reduced pressure. Purification via flash column chromatography (SiO_2, ^{n}Hex / EtOAc, 50 / 1) afforded the title compound as a white solid (>95 atom% D according to ^{1}H NMR; 626 mg, 2.65 mmol, 53%). **^{1}H NMR** (400 MHz, $CDCl_3$) δ 7.89 – 7.79 (m, 3H), 7.69 – 7.65 (m, 1H), 7.53 – 7.44 (m, 2H), 7.33 (dd, *J* = 8.9, 2.4 Hz, 1H), 5.01 (s, 1H). **^{13}C NMR** (100 MHz, $CDCl_3$) δ 153.4, 148.9, 133.8, 131.6, 129.6, 127.9, 127.8, 126.7, 125.9, 120.8, 118.2, 73.0, 21.57 – 20.33 (m). **^{2}H NMR** (77 MHz, $CHCl_3$) δ 1.32 (d, *J* = 0.7 Hz, 6H). **HRMS (ESI):** calc. for $C_{14}H_8NaO_3D_6^+$ $[M+Na]^+$: 259.1212; found: 259.1217.

Naphthalen-2-yl (propan-2-yl-d_7) carbonate (4)

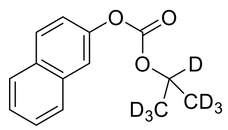

$C_{14}H_7D_7O_3$
MW: 237,31
4

Propan-2-ol-d_8 (99.5 atom% D; 0.5 mL, 6.5 mmol, 1.3 equiv.) was added to a solution of 2-naphthyl chloroformate **S1** (1.0 g, 5.0 mmol, 1.0 equiv.) in DCM (10 mL) at 0 °C. Thereafter, pyridine (0.6 mL, 7.5 mol, 1.5 equiv.) was added dropwise. The reaction was stirred for 10 min at 0 °C before the cooling was removed and stirring continued for 24 h at room temperature. After that, water was added and the layers separated. The org. layer was dried over $MgSO_4$, filtered and the solvent removed under reduced pressure. Purification via flash column chromatography (SiO_2, ^{n}Hex / EtOAc, 50 / 1) afforded the title compound as a white solid (>95 atom% D according to ^{1}H NMR; 725 mg, 3.05 mmol, 61%). **^{1}H NMR** (400 MHz, $CDCl_3$) δ 7.90 – 7.79 (m, 3H), 7.70 – 7.64 (m, 1H), 7.56 – 7.43 (m, 2H), 7.36 – 7.31 (m, 1H). **^{13}C NMR** (100 MHz, $CDCl_3$) δ 153.4, 148.9, 133.8, 131.6, 129.6, 127.9, 127.9, 126.8, 125.9, 120.8, 118.2, 73.04 – 72.37 (m), 21.20 – 20.39 (m). **^{2}H NMR** (77 MHz, $CHCl_3$) δ 4.94 (s, 1H), 1.32 (s, 6H). **HRMS (ESI):** calc. for $C_{14}H_7NaO_3D_7^+$ $[M+Na]^+$: 260.1275; found: 260.1275.

Reactivity assessment

General Information

Reactions were conducted on a 0.1 mmol scale and run in oven-dried 8 mL vials equipped with PTFE coated stir bars. The reactions were set-up and sealed with a rubber lined cap inside an Ar-filled glovebox (H_2O < 0.1 ppm; O_2 < 0.1 ppm). To ensure tight sealing PTFE tape can be wrapped around the cap and the upper part of the vial. The reactions were stirred outside the glovebox in preheated aluminium heating blocks. Therafter, the reactions were let cool to room temperature if necessary, decapped and *n*-decane was added as an internal standard. The mixtures were diluted with EtOAc, an aliquot was taken, filtered over a short plug of silica and submitted for GC analysis.

General procedure C (GPC)

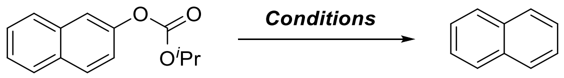

1 (0.1 mmol) **2**

Inside an Ar-filled glovebox, an oven-dried 8 mL vial was charged with carbonate **1** (0.1 mmol, 1.0 equiv.), the Ni(cod)$_2$ and the corresponding ligand. Thereafter, the corresponding solvent (0.5 mL) was added and the vial was sealed. The reaction mixture was stirred at the corresponding temperature for the indicated time. After that, the mixture was let come to room temperature if necessary and the vial was decapped. *n*-Decane was added as an internal standard and the mixture was diluted with EtOAc and an aliquot was taken for GC analysis.

Supplementary Table 1 Assessed conditions and corresponding yields.

Entry	Conditions	Yield 2 / %
**1**	Ni(cod)$_2$ (5 mol%), PCy$_3$ (10 mol%), PhMe (0.2 M), 150 °C, 24 h	32
**2**	Ni(cod)$_2$ (5 mol%), PCy$_3$ (10 mol%), 1,4-dioxane (0.2 M), 150 °C, 24 h	49
**3**	Ni(cod)$_2$ (5 mol%), PCy$_3$ (10 mol%), PhMe (0.2 M), 170 °C, 24 h	29
**4**	Ni(cod)$_2$ (5 mol%), PCy$_3$ (10 mol%), 1,4-dioxane (0.2 M), 170 °C, 24 h	40
**5**	Ni(cod)$_2$ (5 mol%), dcype (6 mol%), PhMe (0.2 M), 150 °C, 24 h	14
**6**	Ni(cod)$_2$ (5 mol%), dcype (6 mol%), 1,4-dioxane (0.2 M), 150 °C, 24 h	23
**7**	Ni(cod)$_2$ (5 mol%), dcype (6 mol%), PhMe (0.2 M), 170 °C, 24 h	15
**8**	Ni(cod)$_2$ (5 mol%), dcype (6 mol%), 1,4-dioxane (0.2 M), 170 °C, 24 h	19
**9**	Ni(cod)$_2$ (5 mol%), PCy$_3$ (10 mol%), DMF (0.2 M), 150 °C, 24 h	9
**10**	Ni(cod)$_2$ (5 mol%), PCy$_3$ (10 mol%), 1,4-dioxane (0.2 M), 100 °C, 24 h	35

**11**	$Ni(cod)_2$ (5 mol%), PCy_3 (10 mol%), 1,4-dioxane (0.2 M), 80 °C, 24 h	16
**12**	$Ni(cod)_2$ (10 mol%), PCy_3 (20 mol%), 1,4-dioxane (0.2 M), 150 °C, 24 h	72
**13**	$Ni(cod)_2$ (20 mol%), PCy_3 (40 mol%), 1,4-dioxane (0.2 M), 150 °C, 24 h	65
**14**	$Ni(cod)_2$ (5 mol%), IMes (6 mol%), 1,4-dioxane (0.2 M), 150 °C, 24 h	86
**15**	$Ni(cod)_2$ (5 mol%), IPr (6 mol%), 1,4-dioxane (0.2 M), 150 °C, 24 h	>95
**16**	$Ni(cod)_2$ (5 mol%), SIMes (6 mol%), 1,4-dioxane (0.2 M), 150 °C, 24 h	22
**17**	$Ni(cod)_2$ (5 mol%), SIPr (6 mol%), 1,4-dioxane (0.2 M), 150 °C, 24 h	93
**18**	$Ni(cod)_2$ (5 mol%), IPr (6 mol%), 1,4-dioxane (0.2 M), 100 °C, 24 h	>95
**19**	$Ni(cod)_2$ (5 mol%), IPr (6 mol%), 1,4-dioxane (0.2 M), 80 °C, 24 h	>95
**20**	$Ni(cod)_2$ (5 mol%), IPr (6 mol%), 1,4-dioxane (0.2 M), 60 °C, 24 h	95
**21**	$Ni(cod)_2$ (5 mol%), IPr (6 mol%), 1,4-dioxane (0.2 M), r.t., 24 h	52
**22**	$Ni(cod)_2$ (10 mol%), IPr (12 mol%), 1,4-dioxane (0.2 M), r.t., 24 h	85
**23**	$Ni(cod)_2$ (20 mol%), IPr (24 mol%), 1,4-dioxane (0.2 M), r.t., 24 h	91
**24**	$Ni(cod)_2$ (10 mol%), IPr (12 mol%), THF (0.2 M), r.t., 48 h	>95

Control reactions

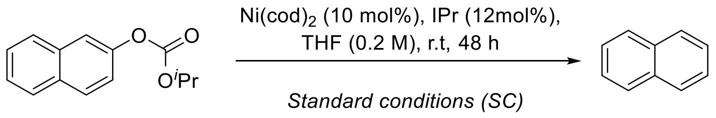

1 (0.1 mmol) **2**

Inside an Ar-filled glovebox, an oven-dried 8 mL vial was charged with carbonate **1** (0.1 mmol, 1.0 equiv.) and either $Ni(cod)_2$ (10 mol%) or IPr (12 mol%). Thereafter THF (0.5 mL) was added and the vial was sealed. The reaction mixture was stirred at room temperature for 48 h. After that, the vial was decapped, *n*-decane was added as an internal standard, the mixture was diluted with EtOAc and an aliquot was taken for GC analysis.

Supplementary Table 2 Control reactions.

Entry	Deviation from SC	Yield 2 / %
**1**	w/o $Ni(cod)_2$	<5
**2**	w/o IPr	<5

Reaction starting from $NiCl_2$(dme) and IPr·CO_2

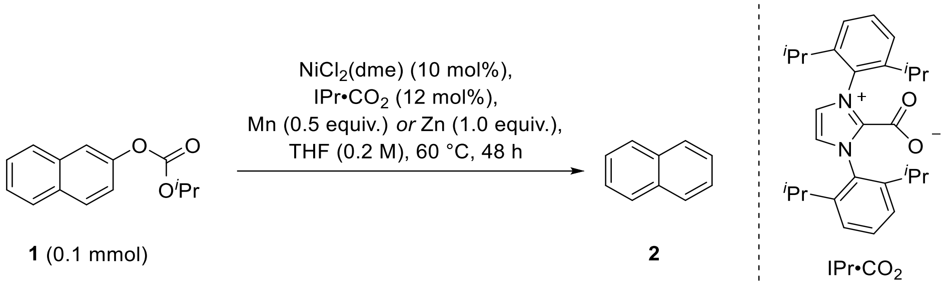

Inside an Ar-filled glovebox, an oven-dried 8 mL vial was charged with carbonate **1** (0.1 mmol, 1.0 equiv.), $NiCl_2$(dme) (10 mol%), IPr·CO_2 (12 mol%) and either Mn (0.5 equiv.) or Zn (1.0 equiv.). Thereafter THF (0.5 mL) was added and the vial was sealed. The reaction mixture was stirred at 60 °C for 48 h. After that, the vial was decapped, *n*-decane was added as an internal standard, the mixture was diluted with EtOAc and an aliquot was taken for GC analysis.

Supplementary Table 3 Results of the reaction employing $NiCl_2$(dme) and IPr·CO_2.

Entry	Reductant	Yield 2 / %
**1**	Mn	71
**2**	Zn	88

Scope Study

Hydrogenolysis

General procedure D (GPD)

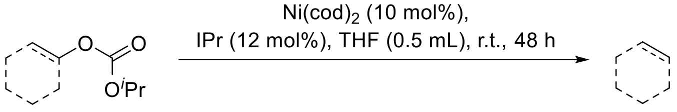

Inside an Ar-filled glovebox, an oven-dried 16 mL vial was charged with the corresponding carbonate (0.5 mmol, 1.0 equiv.), $Ni(cod)_2$ (10 mol%) and IPr (12 mol%). Thereafter THF (2.5 mL) was added and the vial was sealed. The reaction mixture was stirred at room temperature for 48 h. After that, the vial was decapped and the volatiles were removed under reduced pressure. Purification via flash column chromatography afforded the title compounds.

Naphthalene (2)

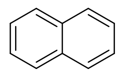

$C_{10}H_8$
MW: 128,17
2

According to **GPD** using carbonate **1**. Purification via flash column chromatography (SiO_2, ^{n}Hex) afforded the title compound as a white solid (51 mg, 0.40 mmol, 79%). **^{1}H NMR** (400 MHz, $CDCl_3$) δ 7.92 – 7.83 (m, 4H), 7.57 – 7.46 (m, 4H). **^{13}C NMR** (100 MHz, $CDCl_3$) δ 133.6, 128.0, 126.0. **HRMS (ESI):** calc. for $C_{10}H_8^+$ $[M]^+$: 128.0621; found: 128.0618.

Naphthalene (5)

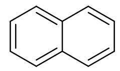

$C_{10}H_8$
MW: 128,17
5

According to **GPD** using carbonate **S2**. Purification via flash column chromatography (SiO_2, ^{n}Hex) afforded the title compound as a white solid (48 mg, 0.38 mmol, 75%). **^{1}H NMR** (500 MHz, $CDCl_3$) δ 7.93 – 7.78 (m, 4H), 7.57 – 7.43 (m, 4H). **^{13}C NMR** (125 MHz, $CDCl_3$) δ 133.6, 128.0, 126.0. **HRMS (ESI):** calc. for $C_{10}H_8^+$ $[M]^+$: 128.0621; found: 128.0617.

Phenanthrene (6)

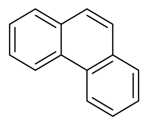

$C_{14}H_{10}$
MW: 178,23
6

According to **GPD** using carbonate **S9**. Purification via flash column chromatography (SiO_2, ^{n}Hex) afforded the title compound as a white solid (87 mg, 0.49 mmol, 98%). **^{1}H NMR** (500 MHz, $CDCl_3$) δ 8.76 – 8.66 (m, 2H), 7.97 – 7.85 (m, 2H), 7.76 (s, 2H), 7.72 – 7.65 (m, 2H), 7.65 – 7.60 (m, 2H). **^{13}C NMR** (125 MHz, $CDCl_3$) δ 132.2, 130.4, 128.7, 127.1, 126.7 (*overlap of 2 signals*), 122.8. **HRMS (ESI):** calc. for $C_{14}H_{10}^+$ $[M]^+$: 178.0777; found: 178.0772.

Trimethyl(naphthalen-2-yl)silane (7)

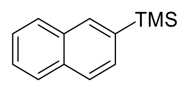

$C_{13}H_{16}Si$
MW: 200,36
7

According to **GPD** using carbonate **S3**. Purification via flash column chromatography (SiO_2, ^{n}Hex) afforded the title compound as a colorless liquid (77 mg, 0.38 mmol, 76%). **^{1}H NMR** (400 MHz, $CDCl_3$) δ 8.09 – 8.06 (m, 1H), 7.95 – 7.84 (m, 3H), 7.67 (dd, *J* = 8.2, 1.2 Hz, 1H), 7.57 – 7.49 (m, 2H), 0.42 (s, 9H). **^{13}C NMR** (100 MHz, $CDCl_3$) δ 138.0, 133.9, 133.8, 133.1, 129.9, 128.2, 127.8, 127.1, 126.3, 126.0, -0.9. **HRMS (ESI):** calc. for $C_{13}H_{16}Si^{+}$ $[M]^{+}$: 200.1016; found: 200.1015.

2-Methoxynaphthalene (8)

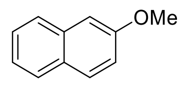

$C_{11}H_{10}O$
MW: 158,20
8

According to **GPD** using carbonate **S4**. Purification via flash column chromatography (SiO_2, ^{n}Hex / EtOAc, 100 / 1) afforded the title compound as a white solid (64 mg, 0.41 mmol, 81%). **^{1}H NMR** (400 MHz, $CDCl_3$) δ 7.81 – 7.71 (m, 3H), 7.45 (ddd, *J* = 8.2, 6.7, 1.3 Hz, 1H), 7.35 (ddd, *J* = 8.1, 6.9, 1.3 Hz, 1H), 7.20 – 7.13 (m, 2H), 3.93 (s, 3H). **^{13}C NMR** (100 MHz, $CDCl_3$) δ 157.7, 134.7, 129.5, 129.1, 127.8, 126.9, 126.5, 123.7, 118.9, 105.9, 55.4. **HRMS (ESI):** calc. for $C_{11}H_{11}O^{+}$ $[M+H]^{+}$: 159.0804; found: 159.0805.

Benzyl 2-naphthoate (9)

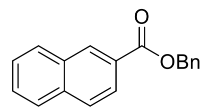

$C_{18}H_{14}O_2$
MW: 262,31
9

According to **GPD** using carbonate **S6**. Purification via flash column chromatography (SiO_2, ^{n}Hex / EtOAc, 50 / 1) afforded the title compound as a white solid (123 mg, 0.47 mmol, 94%). **^{1}H NMR** (400 MHz, $CDCl_3$) δ 8.67 – 8.63 (m, 1H), 8.11 (dd, *J* = 8.6, 1.7 Hz, 1H), 7.98 – 7.82 (m, 3H), 7.63 – 7.48 (m, 4H), 7.46 – 7.34 (m, 3H), 5.44 (s, 2H). **^{13}C NMR** (100 MHz, $CDCl_3$) δ 166.8, 136.3, 135.7, 132.6, 131.4, 129.5, 128.8, 128.4, 128.4, 128.4, 128.3, 127.9, 127.5, 126.8, 125.5, 67.0. **HRMS (ESI):** calc. for $C_{18}H_{14}NaO_2^{+}$ $[M+Na]^{+}$: 285.0886; found: 285.0883.

Naphthalen-2-yl(phenyl)methanone (10)

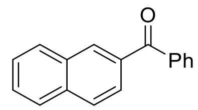

$C_{17}H_{12}O$
MW: 232,28
10

According to **GPD** using carbonate **S5**. Purification via flash column chromatography (SiO_2, ^{n}Hex / EtOAc, 20 / 1) afforded the title compound as a white solid (112 mg, 0.48 mmol, 96%). **^{1}H NMR** (400 MHz, $CDCl_3$) δ 8.31 – 8.24 (m, 1H), 7.99 – 7.89 (m, 4H), 7.89 – 7.84 (m, 2H), 7.67 – 7.59 (m, 2H), 7.59 – 7.49 (m, 3H). **^{13}C NMR** (100 MHz, $CDCl_3$) δ 196.9, 138.1, 135.4, 135.0, 132.5, 132.4, 132.0, 130.2, 129.6, 128.5, 128.5, 128.4, 128.0, 126.9, 125.9. **HRMS (ESI):** calc. for $C_{17}H_{13}O^{+}$ $[M+H]^{+}$: 233.0961; found: 233.0964.

3-(Naphthalen-2-yl)thiophene (11)

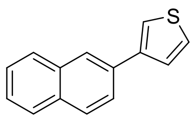

$C_{14}H_{10}S$
MW: 210,29
11

According to **GPD** using carbonate **S7**. Purification via flash column chromatography (SiO_2, ^{n}Hex) afforded the title compound as a white solid (88 mg, 0.42 mmol, 84%). **^{1}H NMR** (400 MHz, $CDCl_3$) δ 8.06 (dd, *J* = 2.1, 0.9 Hz, 1H), 7.91 – 7.83 (m, 3H), 7.76 (dd, *J* = 8.5, 1.8 Hz, 1H), 7.59 (dd, *J* = 2.9, 1.4 Hz, 1H), 7.55 (dd, *J* = 5.0, 1.4 Hz, 1H), 7.53 – 7.47 (m, 2H), 7.47 – 7.42 (m, 1H). **^{13}C NMR** (100 MHz, $CDCl_3$) δ 142.4, 133.9, 133.3, 132.7, 128.6, 128.2, 127.8, 126.6, 126.5 (*overlap of 2 signals*), 125.9, 125.2, 124.9, 120.8. **HRMS (ESI):** calc. for $C_{14}H_{11}S^+$ [M+H]$^+$: 211.0576; found: 211.0574.

1,1'-Biphenyl (13)

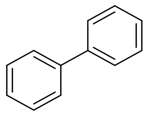

$C_{12}H_{10}$
MW: 154,21
13

According to **GPD** using carbonate **S10**. Purification via flash column chromatography (SiO_2, ^{n}Hex) afforded the title compound as a white solid (70 mg, 0.46 mmol, 91%). **^{1}H NMR** (400 MHz, $CDCl_3$) δ 7.65 – 7.58 (m, 4H), 7.50 – 7.41 (m, 4H), 7.40 – 7.33 (m, 2H). **^{13}C NMR** (100 MHz, $CDCl_3$) δ 141.4, 128.9, 127.4, 127.3. **HRMS (ESI):** calc. for $C_{12}H_{10}^+$ [M]$^+$: 154.0777; found: 154.0774.

1,1'-Biphenyl (14)

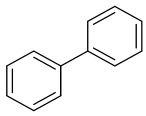

$C_{12}H_{10}$
MW: 154,21
14

According to **GPD** using carbonate **S11** at 60 °C. Purification via flash column chromatography (SiO_2, ^{n}Hex) afforded the title compound as a white solid (55 mg, 0.35 mmol, 71%). **^{1}H NMR** (500 MHz, $CDCl_3$) δ 7.67 – 7.58 (m, 4H), 7.51 – 7.43 (m, 4H), 7.41 – 7.33 (m, 2H). **^{13}C NMR** (125 MHz, $CDCl_3$) δ 141.4, 128.9, 127.4, 127.3.**HRMS (ESI):** calc. for $C_{12}H_{10}^+$ [M]$^+$: 154.0777; found: 154.0774.

1,1'-Biphenyl (15)

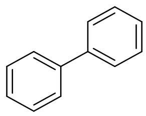

$C_{12}H_{10}$
MW: 154,21
15

According to **GPD** using carbonate **S12** at 60 °C. Purification via flash column chromatography (SiO_2, ^{n}Hex) afforded the title compound as a white solid (57 mg, 0.37 mmol, 74%). **^{1}H NMR** (500 MHz, $CDCl_3$) δ 7.65 – 7.59 (m, 4H), 7.49 – 7.42 (m, 4H), 7.41 – 7.34 (m, 2H). **^{13}C NMR** (125 MHz, $CDCl_3$) δ 141.4, 128.9, 127.4, 127.3.**HRMS (ESI):** calc. for $C_{12}H_{10}^+$ [M]$^+$: 154.0777; found: 154.0775.

1-Methylnaphthalene (21)

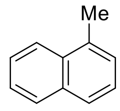

$C_{11}H_{10}$
MW: 142,20
21

According to **GPD** using carbonate **S13** at 60 °C. Purification via flash column chromatography (SiO_2, ^{n}Hex) afforded the title compound as a colorless liquid (59 mg, 0.41 mmol, 82%). **^{1}H NMR** (400 MHz, $CDCl_3$) δ 8.12 – 8.05 (m, 1H), 7.96 – 7.92 (m, 1H), 7.82 – 7.78 (m, 1H), 7.65 – 7.54 (m, 2H), 7.50 – 7.43 (m, 1H), 7.43 – 7.39 (m, 1H), 2.79 (s, 3H). **^{13}C NMR** (100 MHz, $CDCl_3$) δ 134.4, 133.7, 132.8, 128.7, 126.7, 126.5, 125.8, 125.7, 125.7, 124.2, 19.5. **HRMS (ESI):** calc. for $C_{11}H_{10}^{+}$ $[M]^{+}$: 142.0777; found: 142.0774.

2-Ethylnaphthalene (22)

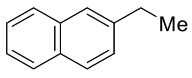

$C_{12}H_{12}$
MW: 156,23
22

According to **GPD** using carbonate **S14** in 1,4-dioxane at 120 °C. Purification via flash column chromatography (SiO_2, ^{n}Hex) afforded the title compound as a colorless liquid (48 mg, 0.31 mmol, 62%). **^{1}H NMR** (400 MHz, $CDCl_3$) δ 7.89 – 7.75 (m, 3H), 7.68 – 7.65 (m, 1H), 7.51 – 7.42 (m, 2H), 7.39 (dd, *J* = 8.4, 1.8 Hz, 1H), 2.86 (q, *J* = 7.6 Hz, 3H), 1.37 (t, *J* = 7.6 Hz, 2H). **^{13}C NMR** (100 MHz, $CDCl_3$) δ 141.9, 133.9, 132.1, 127.9, 127.7, 127.6, 127.2, 126.0, 125.7, 125.1, 29.2, 15.7. **HRMS (ESI):** calc. for $C_{12}H_{12}^{+}$ $[M]^{+}$: 156.0934; found: 156.0932.

1,2-Dihydronaphthalene (24)

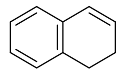

$C_{10}H_{10}$
MW: 130,19
24

According to **GPD** using carbonate **S21** at 100 °C. Purification via flash column chromatography (SiO_2, ^{n}Hex) afforded the title compound as a colorless liquid (45 mg, 0.35 mmol, 69%). **^{1}H NMR** (400 MHz, $CDCl_3$) δ 7.22 – 7.09 (m, 3H), 7.09 – 6.99 (m, 1H), 6.48 (dt, *J* = 9.6, 1.9 Hz, 1H), 6.05 (dt, *J* = 9.6, 4.4 Hz, 1H), 2.82 (t, *J* = 8.2 Hz, 2H), 2.34 (dddd, *J* = 9.6, 8.2, 4.4, 1.9 Hz, 2H). **^{13}C NMR** (100 MHz, $CDCl_3$) δ 135.6, 134.3, 128.8, 127.9, 127.6, 127.0, 126.6, 126.0, 27.6, 23.3. **HRMS (ESI):** calc. for $C_{10}H_{10}^{+}$ $[M]^{+}$: 130.0777; found: 130.0773.

1-Benzyl-1,2,3,6-tetrahydropyridine (25)

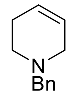

$C_{12}H_{15}N$
MW: 173,26
25

According to **GPD** using carbonate **S22** at 80 °C. Purification via flash column chromatography (basic Al_2O_3, ^{n}Hex/EtOAc 100/1 *to* 50/1) afforded the title compound as a yellow liquid (44 mg, 0.26 mmol, 51%). **^{1}H NMR** (500 MHz, $CDCl_3$) δ 7.41 – 7.26 (m, 5H), 5.84 – 5.75 (m, 1H), 5.74 – 5.66 (m, 1H), 3.62 (s, 2H), 3.09 – 2.96 (m, 2H), 2.59 (t, *J* = 5.7 Hz, 2H), 2.26 – 2.15 (m, 2H). **^{13}C NMR** (125 MHz, $CDCl_3$) δ 138.5, 129.3, 128.3, 127.1, 125.5, 125.4, 63.1, 52.9, 49.8, 26.3. **HRMS (ESI):** calc. for $C_{12}H_{16}N^{+}$ $[M+H]^{+}$: 174.1277; found: 174.1279.

Oxidation

General procedure E (GPE)

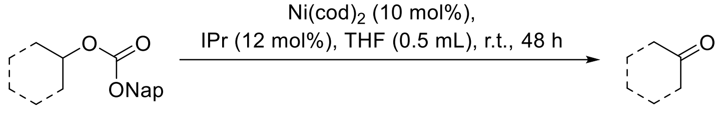

Inside an Ar-filled glovebox, an oven-dried 16 mL vial was charged with the corresponding carbonate (0.5 mmol, 1.0 equiv.), $Ni(cod)_2$ (10 mol%) and IPr (12 mol%). Thereafter THF (2.5 mL) was added and the vial was sealed. The reaction mixture was stirred at room temperature for 48 h. After that, the vial was decapped and the volatiles were removed under reduced pressure. Purification via flash column chromatography afforded the title compounds.

Bis(4-fluorophenyl)methanone (42)

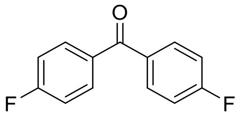

$C_{13}H_8F_2O$
MW: 218,20
42

According to **GPE** using carbonate **S30**. Purification via flash column chromatography (SiO_2, ^{n}Hex / EtOAc, 100 / 1) afforded the title compound as a white solid (83 mg, 0.38 mmol, 76%). **^{1}H NMR** (500 MHz, $CDCl_3$) δ 7.84 – 7.79 (m, 4H), 7.21 – 7.13 (m, 4H). **^{13}C NMR** (125 MHz, $CDCl_3$) δ 193.9, 165.5 (d, *J* = 254.3 Hz), 133.8 (d, *J* = 3.1 Hz), 132.6 (d, *J* = 9.2 Hz), 115.7 (d, *J* = 21.9 Hz). **^{19}F NMR** (471 MHz, $CDCl_3$) δ -105.8. **HRMS (ESI):** calc. for $C_{13}H_8F_2O^+$ [M]$^+$: 218.0538; found: 218.0533.

(8*S*,9*S*,10*R*,13*R*,14*S*,17*R*)-10,13-dimethyl-17-((*R*)-6-methylheptan-2-yl)-1,2,6,7,8,9,10,11,12,13,14,15,16,17-tetradecahydro-3*H*-cyclopenta[*a*]phenanthren-3-one (49)

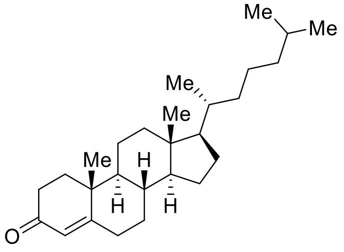

$C_{27}H_{44}O$
MW: 384,65
49

According to **GPE** using carbonate **S37** at 60 °C. Purification via flash column chromatography (SiO_2, ^{n}Hex / EtOAc, 10 / 1) afforded the title compound as a white solid (137 mg, 0.36 mmol, 71%). **^{1}H NMR** (500 MHz, $CDCl_3$) δ 5.74 – 5.69 (m, 1H), 2.49 – 2.20 (m, 4H), 2.08 – 1.97 (m, 2H), 1.91 – 1.79 (m, 2H), 1.69 (td, *J* = 13.9, 4.6 Hz, 1H), 1.64 – 0.96 (m, 21H), 0.95 – 0.88 (m, 4H), 0.86 (dd, *J* = 6.6, 2.3 Hz, 6H), 0.70 (s, 3H).**^{13}C NMR** (125 MHz, $CDCl_3$) δ 199.8, 171.8, 123.9, 56.2, 56.0, 54.0, 42.5, 39.8, 39.6, 38.7, 36.3, 35.9, 35.8, 35.8, 34.1, 33.1, 32.2, 28.3, 28.2, 24.3, 24.0, 23.0, 22.7, 21.2, 18.8, 17.5, 12.1. **HRMS (ESI):** calc. for $C_{27}H_{45}O^+$ [M+H]$^+$: 385.3465; found: 385.346. Structure assignment was corroborated via single crystal X-ray structure analysis (see appendix X-Ray data section).

General procedure F (GPF)

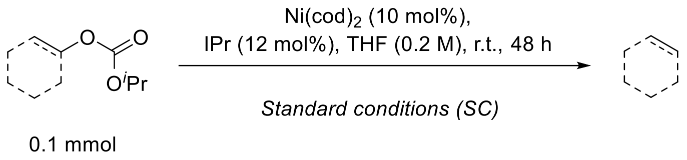

Inside an Ar-filled glovebox, an oven-dried 8 mL vial was charged with the corresponding carbonate (0.1 mmol, 1.0 equiv.), $Ni(cod)_2$ (10 mol%) and IPr (12 mol%). Thereafter THF (0.5 mL) was added and the vial was sealed. The reaction mixture was stirred at room temperature for 48 h. After that, the vial was decapped, *n*-decane was added as an internal standard, the mixture was diluted with EtOAc and an aliquot was taken for GC analysis.

Supplementary Table 4 Results of the substrates obtained via **GPF**.

Entry	Product	Deviation(s) from SC	Yield / %
**1**	**12**	-	90
**2**	**25**	80 °C	73
**3**	**26**		35
**4**	**27**		90
**5**	**23**	100 °C	74
**6**	**24**		87
**7**	**28**		53
**8**	**16**	1,4-dioxane (0.2 M), 120 °C	71
**9**	**17**		61
**10**	**18**		54
**11**	**19**	$Ni(cod)_2$ (20 mol%), IPr (24 mol%), 1,4-dioxane (0.2 M), 160 °C	56
**12**	**20**		50

General procedure G (GPG)

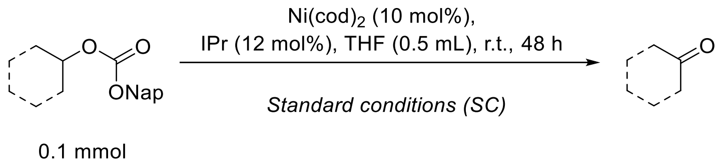

Inside an Ar-filled glovebox, an oven-dried 8 mL vial was charged with the corresponding carbonate (0.1 mmol, 1.0 equiv.), $Ni(cod)_2$ (10 mol%) and IPr (12 mol%). Thereafter THF (0.5 mL) was added and the vial was sealed. The reaction mixture was stirred at room temperature for 48 h. After that, the vial was decapped, *n*-decane was added as an internal standard, the mixture was diluted with EtOAc and an aliquot was taken for GC analysis.

Supplementary Table 5 Results of the substrates obtained via **GPG**.

Entry	Product	Deviation from SC	Yield / %
**1**	**38**		92
**2**	**39**		61
**3**	**40**		70
**4**	**41**		89
**5**	**43**	-	79
**6**	**44**		98
**7**	**46**		19
**8**	**47**		77
**9**	**48**		16
**11**	**45**	60 °C	50

Additive Study

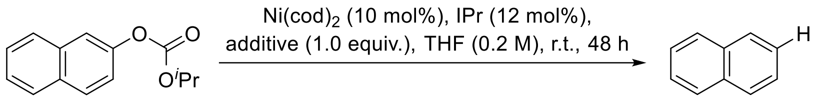

1 (0.1 mmol) **2**

Additives:

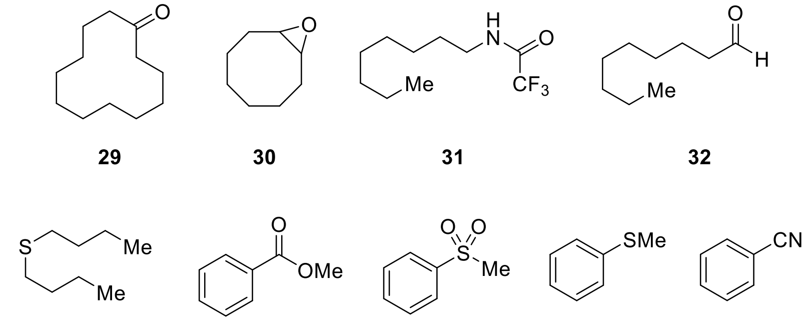

29 **30** **31** **32**

33 **34** **35** **36** **37**

Inside an Ar-filled glovebox, an oven-dried 8 mL vial was charged with carbonate **1** (0.1 mmol), and the corresponding additive (1.0 equiv.). Thereafter, 0.5 mL of a stock solution containing Ni(cod)$_2$ (0.02 M) and IPr (0.024 M) in THF was added and the vial was sealed. The reaction mixture was stirred at room temperature for 48 h. After that, the vial was decapped, *n*-decane was added as an internal standard, the mixture was diluted with EtOAc and an aliquot was taken for GC analysis.

Supplementary Table 6 Results of the additive study.

Entry	Additive	Yield 2 / %	Conversion Additive / %
**1**	**29**	97	<5
**2**	**30**	78	<5
**3**	**31**	95	<5
**4**	**32**	<5	71
**5**	**33**	79	5
**6**	**34**	97	<5
**7**	**35**	94	10
**8**	**36**	17	9
**9**	**37**	<5	35

Stability study

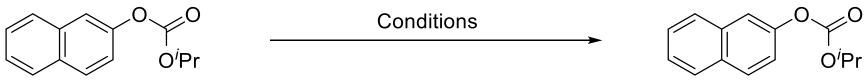

1 1

A 8 mL vial was charged with carbonate **1** (0.1 mmol) and reacted under the corresponding condition for 18 h. After that, *n*-decane was added as an internal standard, the mixture was diluted with EtOAc and an aliquot was taken for GC analysis.

Supplementary Table 7 Results of the stability study.

Entry	Conditions	Conversion 1 / %
**1**	K_2CO_3 (1.0 equiv.), MeOH (0.2 M), r.t.	>95
**2**	TFA / DCM (1 / 1; 0.2 M), r.t.	<5
**3**	Piperidine (0.2 equiv.), DMF (0.2 M), r.t.	>95
**4**	Zn (2.0 equiv.), AcOH (0.2 M), r.t.	<5
**5**	TBAF (0.5 mL, 1 M in THF), r.t.	>95
**6**	1,4-dioxane (0.2 M), 160 °C	<5
**7**	HCl (0.5 mL, 1 M in MeOH), r.t.	<5
**8**	DDQ (2.0 equiv.), DCM / MeOH (8 / 1; 0.2 M), r.t.	12
**9**	NEt_3 (2.0 equiv.), DCM (0.2 M), r.t.	22

tert-Butyl aryl carbonates as non-innocent electrophiles (NIE) for the reduction/oxidation of C–O bonds

Oxidation of alcohols

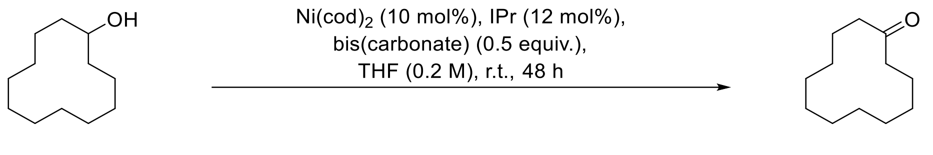

51 (0.1 mmol) **38**

Bis(carbonate)s:

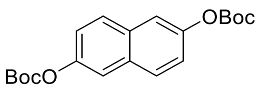

S38

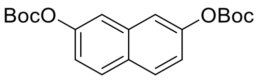

S39

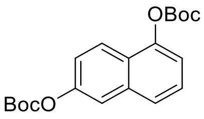

S40

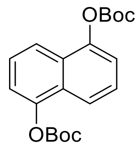

52

Inside an Ar-filled glovebox, an oven-dried 8 mL vial was charged with cyclododecanol **51** (0.1 mmol, 1.0 equiv.), Ni(cod)$_2$ (10 mol%), IPr (12 mol%) and the corresponding bis(carbonate) (0.5 equiv.). Thereafter THF (0.5 mL) was added and the vial was sealed. The reaction mixture was stirred at room temperature for 48 h. After that, the vial was decapped, *n*-decane was added as an internal standard, the mixture was diluted with EtOAc and an aliquot was taken for GC analysis.

Supplementary Table 8 Results for the oxidation of alcohols.

Entry	Bis(carbonate)	Yield / %
**1**	**S38**	64
**2**	**S39**	53
**3**	**S40**	69
**4**	**52**	94

Reduction of *tert*-butyl aryl carbonates

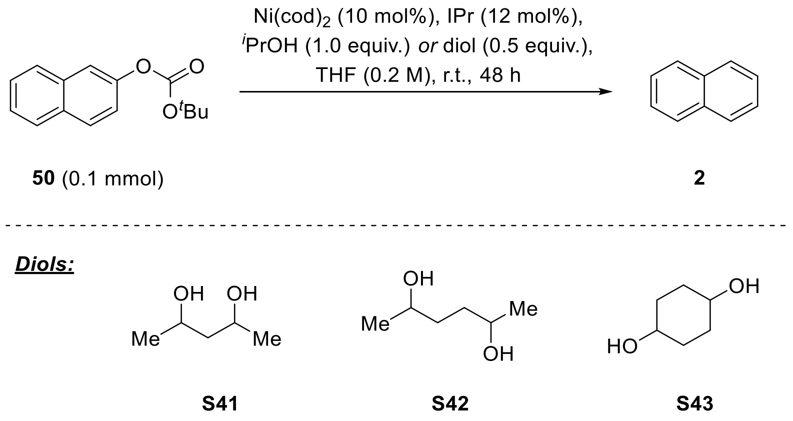

Inside an Ar-filled glovebox, an oven-dried 8 mL vial was charged with carbonate **50** (0.1 mmol, 1.0 equiv.), Ni(cod)$_2$ (10 mol%) and IPr (12 mol%). Thereafter THF (0.5 mL) and isopropanol (1.0 equiv.) or the corresponding diol (0.5 equiv.) were added and the vial was sealed. The reaction mixture was stirred at room temperature for 48 h. After that, the vial was decapped, *n*-decane was added as an internal standard, the mixture was diluted with EtOAc and an aliquot was taken for GC analysis.

Supplementary Table 9 Results for the reduction of tert-butyl aryl carbonates.

Entry	Reductant	Yield / %
**1**	^{i}PrOH	89
**2**	**S41**	34
**3**	**S42**	76
**4**	**S43**	44

Miniaturization experiments

General information

Miniaturization experiments were performed in oven-dried 0.2 mL crimp neck microvials (VWR™, 0.2 crimp neck vial, ND8, clear glass, conical, cat. no.: 548-0078) capped with aluminium crimp caps equipped with a PTFE/silicone/PTFE septum (VWR™, aluminium crimp caps, ND8 with central hole, 4 mm and septum, 45° shore A, PTFE red/silicone white/PTFE red, cat. no.: 548-0038). Experiments were perfomed on a micromole scale (2 µmol, 10 µL total reaction volume) and yields are reported as the average of three independent runs.

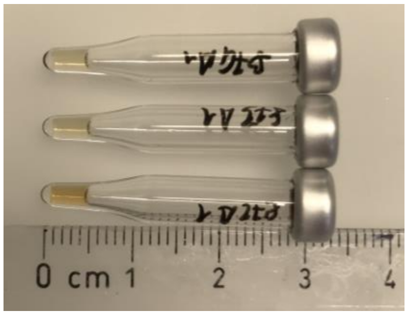

Supplementary Figure 1 Sealed microvials containing the micromole-scale reactions.

Micromole-scale reduction of isopropyl aryl carbonates

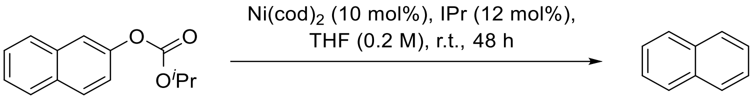

1 (2 µmol) **2**

Inside an Ar-filled glovebox, an oven-dried 8 mL vial was charged with carbonate **1** (0.1 mmol, 1.0 equiv.), Ni(cod)$_2$ (10 mol%) and IPr (12 mol%). Thereafter THF (0.5 mL) and *n*-decane (1.0 equiv.) as an internal standard were added. 10 µL of the resulting solution (equivalent to 2 µmol of carbonate **1**) were added to a 0.2 mL crimp neck microvial. The vial was capped with an aluminium crimp cap and the reaction was left standing at room temperature for 48 h outside the glovebox. After that, the microvial was decapped, the reaction mixture was diluted with a small amount of EtOAc and the complete content of the microvial was used for GC-FID analysis.

Supplementary Table 10 Result of the micromole-scale reduction of isopropyl aryl carbonates.

Entry	Yield 2 / %
1	73

Micromole-scale reduction of *tert*-butyl aryl carbonates

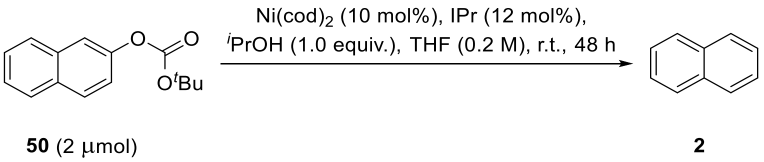

Inside an Ar-filled glovebox, an oven-dried 8 mL vial was charged with carbonate **50** (0.1 mmol, 1.0 equiv.), $Ni(cod)_2$ (10 mol%) and IPr (12 mol%). Thereafter THF (0.5 mL), ^{i}PrOH (1.0 equiv.) and *n*-decane (1.0 equiv.) as an internal standard were added. 10 µL of the resulting solution (equivalent to 2 µmol of carbonate **50**) were added to a 0.2 mL crimp neck microvial. The vial was capped with an aluminium crimp cap and the reaction was left standing at room temperature for 48 h outside the glovebox. After that, the microvial was decapped, the reaction mixture was diluted with a small amount of EtOAc and the complete content of the microvial was used for GC-FID analysis.

Supplementary Table 11 Result of the micromole-scale reduction of tert-butyl aryl carbonates.

Entry	Yield 2 / %
1	62

Micromole-scale oxidation of alcohols

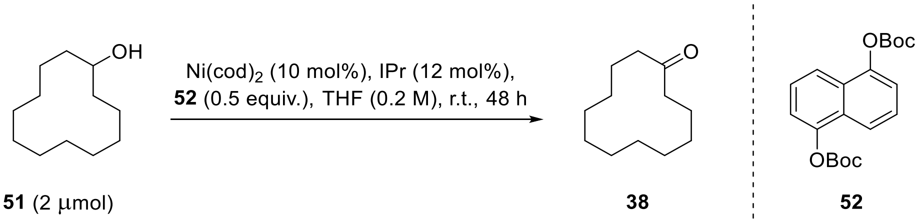

Inside an Ar-filled glovebox, an oven-dried 8 mL vial was charged with cyclododecanol **51** (0.1 mmol, 1.0 equiv.), $Ni(cod)_2$ (10 mol%), IPr (12 mol%) and carbonate **52** (0.5 equiv.). Thereafter THF (0.5 mL) and *n*-decane (1.0 equiv.) as an internal standard were added. 10 µL of the resulting solution (equivalent to 2 µmol of alcohol **51**) were added to a 0.2 mL crimp neck microvial. The vial was capped with an aluminium crimp cap and the reaction was left standing at room temperature for 48 h outside the glovebox. After that, the microvial was decapped, the reaction mixture was diluted with a small amount of EtOAc and the complete content of the microvial was used for GC-FID analysis.

Supplementary Table 12 Result of the micromole-scale oxidation of secondary alcohols.

Entry	Yield 2 / %
1	59

Fluorescence-based reactivity assay

Reactions were analyzed using a microplate reader by measuring (excitation wavelength 360 nm; emission wavelength 465 nm) the relative fluorescence intensity of the samples at the beginning and at the end of each reaction. Therefore, the reactions were run as described below. Thereafter, they were entered inside an Ar-filled glovebox and opened therein. 100 µL of each reaction mixture was added to individual wells on a 96-well microplate. *NOTE The following part has to be conducted fast to ensure low conversions of the freshly prepared reactions:* In addition, 100 µL of the corresponding freshly prepared and precooled to -30 °C reaction mixture was added for comparison, a lid was placed on the microplate and it was exited from the glovebox and measured quickly (ca. 1-2 min from the addition). Measurements were conducted as triplicates and the average values are reported in relative fluorescence units (RFU).

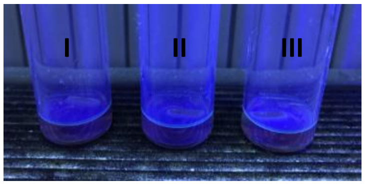

Supplementary Figure 2 Samples after the reaction under 366 nm irradiation.

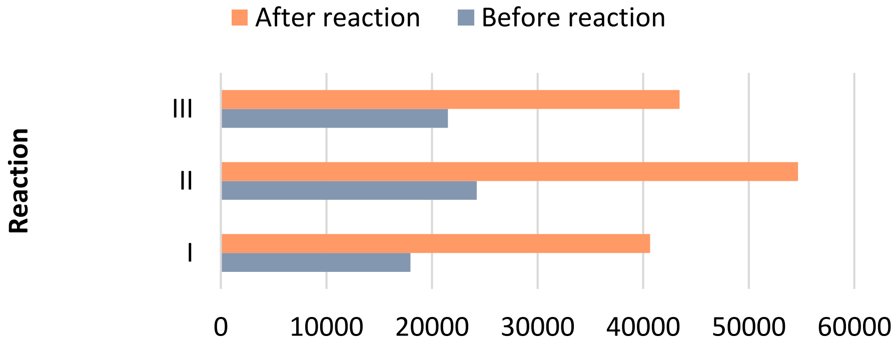

	I	II	III
After reaction	40658	54667	43447
Before reaction	17958	24236	21504

Relative fluorescence units (RFU)

Supplementary Figure 3 Relative fluorescence before and after the reactions.

Reaction I: ***Reduction of isopropyl aryl carbonates***

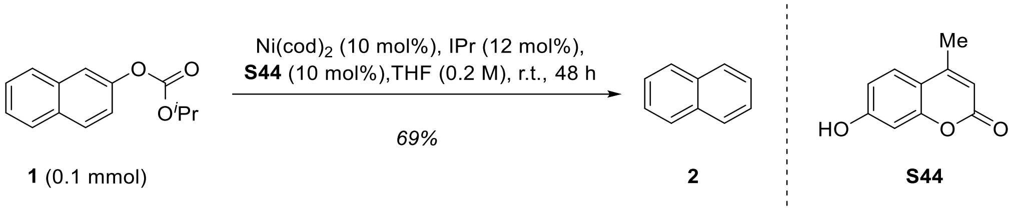

Inside an Ar-filled glovebox, an oven-dried 8 mL vial was charged with carbonate **1** (0.1 mmol, 1.0 equiv.), Ni(cod)$_2$ (10 mol%), IPr (12 mol%) and hydroxycoumarin **S44** (10 mol%). Thereafter THF (0.5 mL) was added and the vial was sealed. The reaction mixture was stirred at room temperature for 48 h outside the glovebox.

Reaction II: ***Reduction of tert-butyl aryl carbonates***

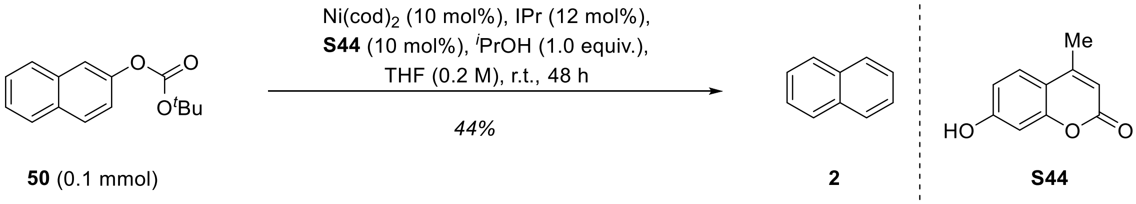

Inside an Ar-filled glovebox, an oven-dried 8 mL vial was charged with carbonate **50** (0.1 mmol, 1.0 equiv.), Ni(cod)$_2$ (10 mol%), IPr (12 mol) and hydroxycoumarin **S44** (10 mol%). Thereafter THF (0.5 mL) and isopropanol (1.0 equiv.) was added and the vial was sealed. The reaction mixture was stirred at room temperature for 48 h outside the glovebox.

Reaction III: ***Oxidation of alcohols***

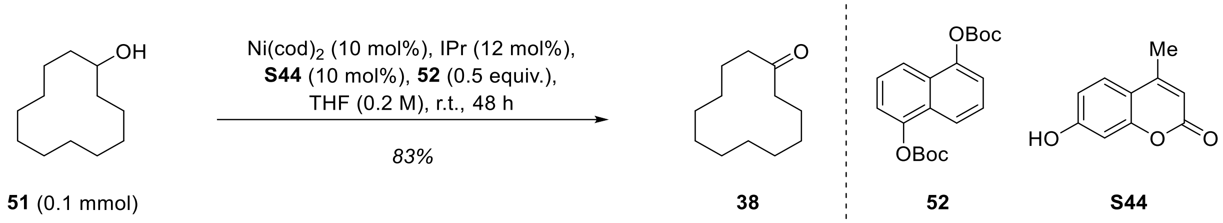

Inside an Ar-filled glovebox, an oven-dried 8 mL vial was charged with cyclododecanol **51** (0.1 mmol, 1.0 equiv.), Ni(cod)$_2$ (10 mol%), IPr (12 mol%), hydroxycoumarin **S44** (10 mol%) and carbonate **52** (0.5 equiv.). Thereafter THF (0.5 mL) was added and the vial was sealed. The reaction mixture was stirred at room temperature for 48 h outside the glovebox.

Mechanistic experiments

Deuterium labeling experiments

Starting from carbonate 1 and THF-d_8

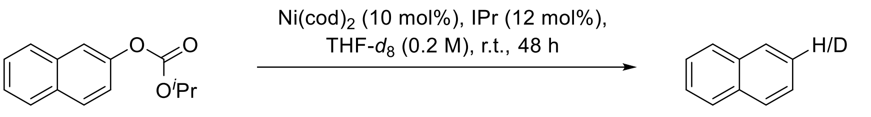

1 (0.5 mmol) **2** (69%; <5 atom% D)

Inside an Ar-filled glovebox, an oven-dried 16 mL vial was charged with carbonate **1** (0.5 mmol, 1.0 equiv.), Ni(cod)$_2$ (10 mol%) and IPr (12 mol%). Thereafter THF-d_8 (99.5 atom% D; 2.5 mL) was added and the vial was sealed. The reaction mixture was stirred at room temperature for 48 h. After that, the vial was decapped and the volatiles were removed under reduced pressure. Purification via flash column chromatography (SiO$_2$, ^{n}Hex) afforded napthalene **2** as a white solid (<5 atom% D according to ^{1}H NMR; 44 mg, 0.34 mmol, 69%). **^{1}H NMR** (500 MHz, CDCl$_3$) δ 7.89 – 7.84 (m, 4H), 7.52 – 7.47 (m, 4H). **^{13}C NMR** (125 MHz, CDCl$_3$) δ 133.6, 128.0, 126.0. **HRMS (ESI):** calc. for $C_{10}H_8^+$ [M]$^+$: 128.0621; found: 128.0618.

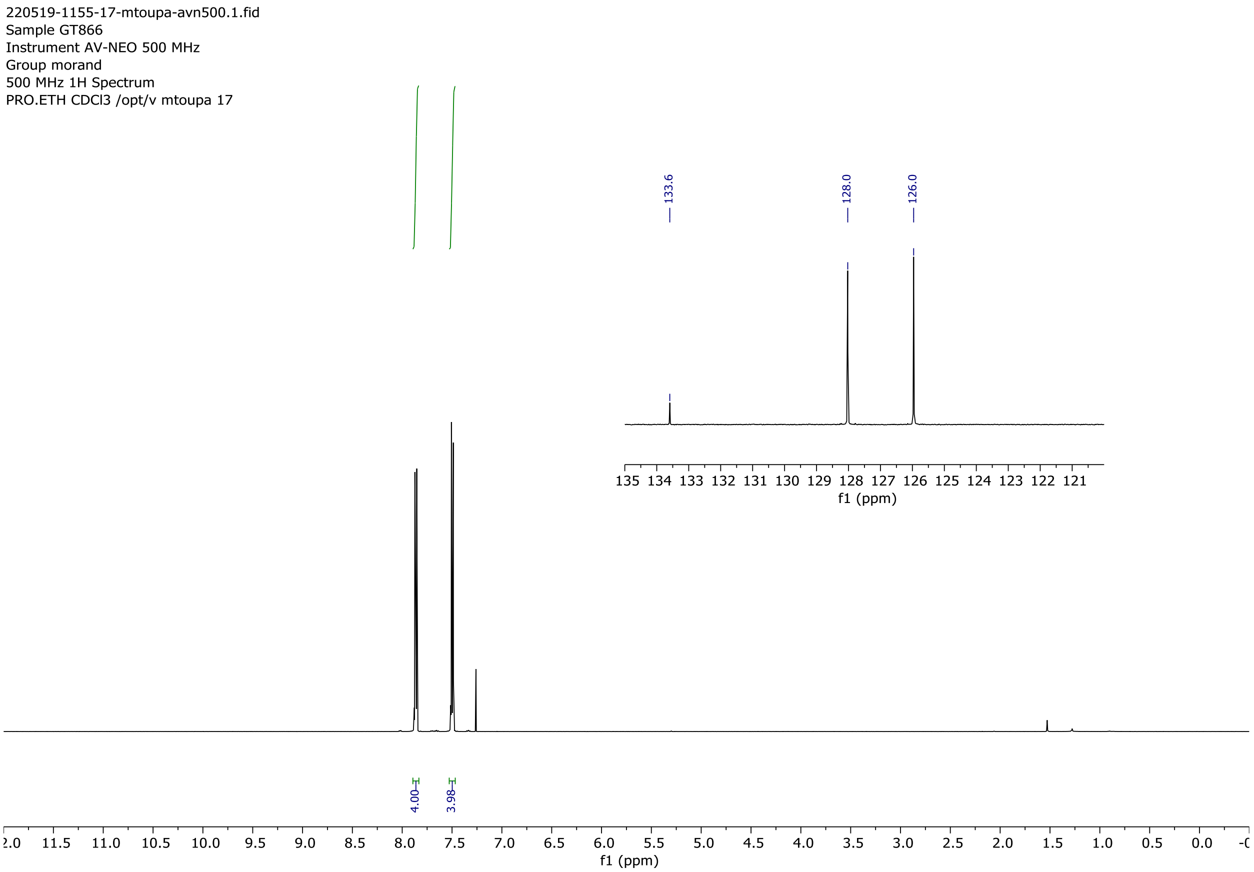

Supplementary Figure 4 ^{1}H NMR (500 MHz, CDCl$_3$, 25 °C) and ^{13}C NMR (125 MHz, CDCl$_3$, 25 °C) of compound **2** after the reaction with THF-d_8.

Starting from carbonate 3

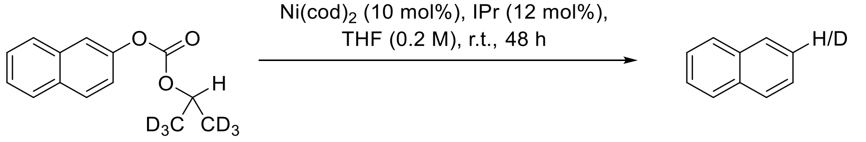

3 (0.5 mmol) **2** (73%; <5 atom% D)

Inside an Ar-filled glovebox, an oven-dried 16 mL vial was charged with carbonate **3** (0.5 mmol, 1.0 equiv.), $Ni(cod)_2$ (10 mol%) and IPr (12 mol%). Thereafter THF (2.5 mL) was added and the vial was sealed. The reaction mixture was stirred at room temperature for 48 h. After that, the vial was decapped and the volatiles were removed under reduced pressure. Purification via flash column chromatography (SiO_2, ^{n}Hex) afforded napthalene **2** as a white solid (<5 atom% D according to ^{1}H NMR; 47 mg, 0.37 mmol, 73%). **^{1}H NMR** (500 MHz, $CDCl_3$) δ 7.91 – 7.83 (m, 4H), 7.53 – 7.47 (m, 4H). **^{13}C NMR** (125 MHz, $CDCl_3$) δ 133.6, 128.0, 126.0. **HRMS (ESI):** calc. for $C_{10}H_8^+$ $[M]^+$: 128.0621; found: 128.0619.

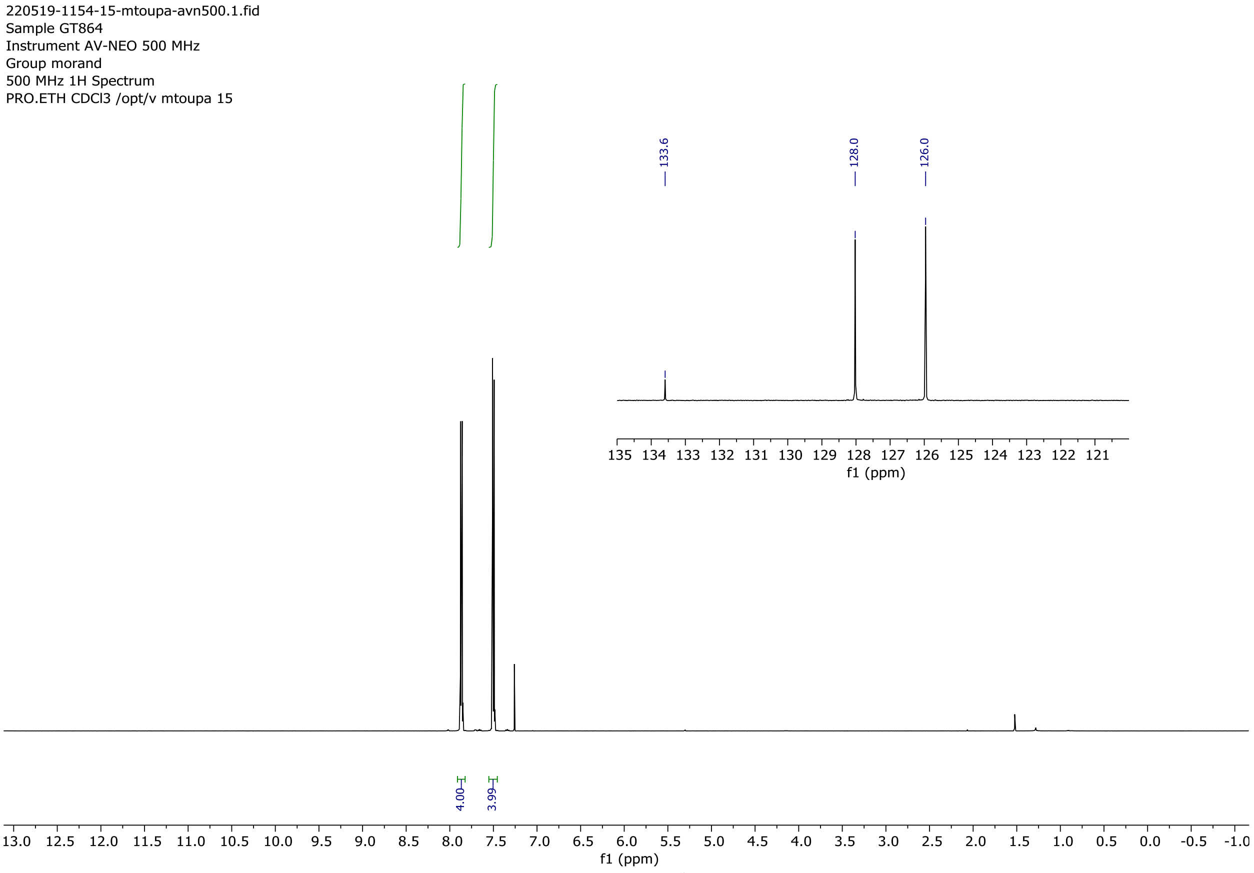

Supplementary Figure 5 ^{1}H NMR (500 MHz, $CDCl_3$, 25 °C) and ^{13}C NMR (125 MHz, $CDCl_3$, 25 °C) of compound **2** after the reaction with carbonate **3**.

Starting from carbonate 4

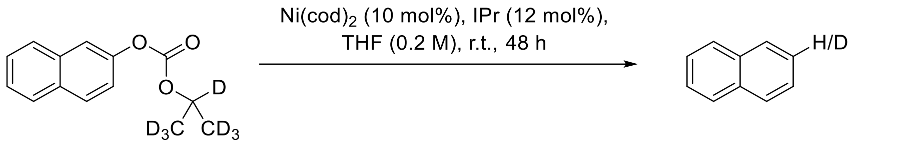

Inside an Ar-filled glovebox, an oven-dried 16 mL vial was charged with carbonate **4** (0.5 mmol, 1.0 equiv.), $Ni(cod)_2$ (10 mol%) and IPr (12 mol%). Thereafter THF (2.5 mL) was added and the vial was sealed. The reaction mixture was stirred at room temperature for 48 h. After that, the vial was decapped and the volatiles were removed under reduced pressure. Purification via flash column chromatography (SiO_2, ^{n}Hex) afforded napthalene **2-*d*** as a white solid (>95 atom% D according to ^{1}H NMR; 49 mg, 0.38 mmol, 76%). **^{1}H NMR** (500 MHz, $CDCl_3$) δ 7.90 – 7.83 (m, 4H), 7.54 – 7.46 (m, 3H). **^{13}C NMR** (125 MHz, $CDCl_3$) δ 133.6, 128.0, 127.9, 126.0, 125.9, 125.7 (t, *J* = 24.4 Hz). **^{2}H NMR** (77 MHz, $CHCl_3$) δ 7.8. Spectral data matches literature.[9] **HRMS (ESI):** calc. for $C_{10}H_7D^+$ $[M]^+$: 129.0683; found: 129.068.

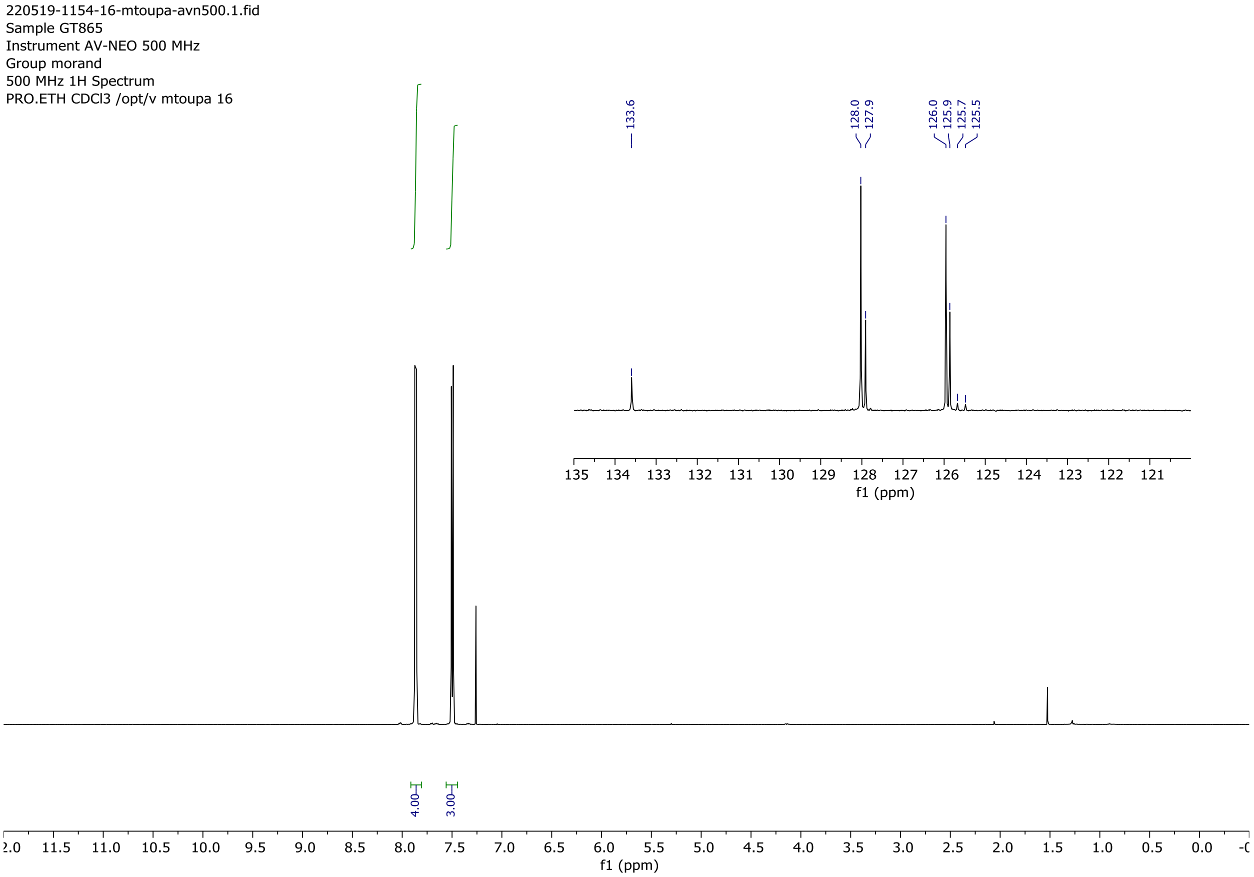

Supplementary Figure 6 ^{1}H NMR (500 MHz, $CDCl_3$, 25 °C) and ^{13}C NMR (125 MHz, $CDCl_3$, 25 °C) of compound **2-d** after the reaction with carbonate **4.**

Kinetic investigations

General Information

Kinetic investigations were performed using the initial rates method at early conversions (<30%). Data points were collected every 60 seconds during the first 5 minutes of the reactions. Reaction progress was determined via product formation and monitored via GC-analysis. All experiments were conducted as triplicates and initial rates are reported as the average of three independent runs.

Variation of parameters

Substrate 1

Inside an Ar-filled glovebox, an oven-dried 16 mL vial was charged with the corresponding amount of carbonate **1**. *n*-Decane (1.0 equiv. regarding the amount of **1**) was added as an internal standard followed by THF (1.5 mL). Thereafter, 1 mL of a stock solution containing $Ni(cod)_2$ (0.05 M) and IPr (0.06 M) in THF was added. After the addition was completed, the reactions were stirred at room temperature and 0.25 mL aliquots were taken every minute for the first 5 minutes, filtered over a plug of silica and analysed via GC.

Supplementary Table 13 Kinetic data for the variation of the amount of substrate **1**.

Entry	[1] / M	t / s	[2] / M			Average rate / Ms^{-1} x10^{-4}	Standard deviation / Ms^{-1} x10^{-4}
			Reaction 1	*Reaction 2*	*Reaction 3*		
1	0.2	60	0.0302	0.0303	0.0300	1.478	0.087
2		120	0.0399	0.0414	0.0394		
3		180	0.0502	0.0511	0.0501		
4		240	0.0583	0.0599	0.0551		
5		300	0.0666	0.0677	0.0629		
6	0.3	60	0.0289	0.0337	0.0310	1.584	0.085
7		120	0.0400	0.0445	0.0429		
8		180	0.0499	0.0544	0.0533		
9		240	0.0587	0.0639	0.0595		
10		300	0.0684	0.0713	0.0691		
11	0.4	60	0.0322	0.0310	0.0339	1.588	0.091
12		120	0.0422	0.0434	0.0449		
13		180	0.0531	0.0540	0.0558		
14		240	0.0612	0.0637	0.0601		
15		300	0.0708	0.0717	0.0705		
16	0.6	60	0.0325	0.0354	0.0325	1.677	0.018
17		120	0.0442	0.0463	0.0436		

18	180	0.0531	0.0570	0.0562
19	240	0.0642	0.0662	0.0624
20	300	0.0732	0.0761	0.0727

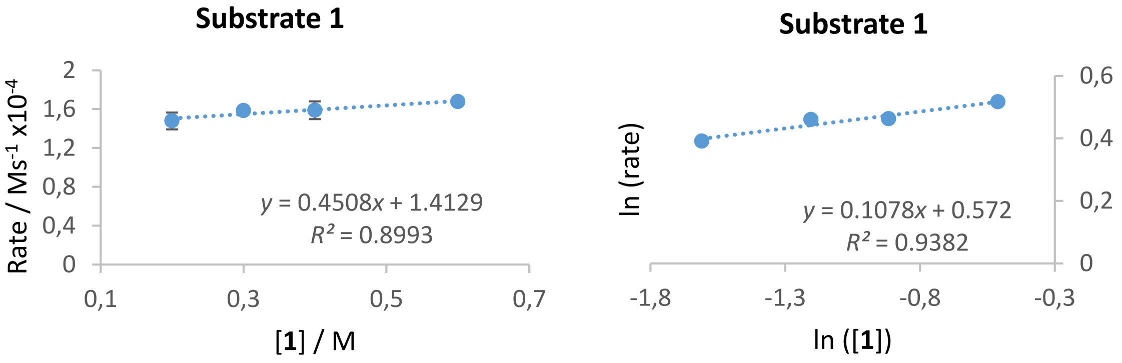

Supplementary Figure 7 Plot of the measured initial rate vs concentration of substrate **1** (left) and corresponding logarithmic representation (right). Error bars represent the standard deviation of three independent experiments.

Catalyst

Inside an Ar-filled glovebox, an oven-dried 16 mL vial was charged with the corresponding amount of Ni(cod)$_2$ and IPr (ratio 1 / 1.2) and THF (1.5 mL) was added. Thereafter, 1 mL of a stock solution containing carbonate **1** (0.5 M) and *n*-decane (0.5 M) in THF was added. After the addition was completed, the reactions were stirred at room temperature and 0.25 mL aliquots were taken every minute for the first 5 minutes, filtered over a plug of silica and analysed via GC.

Supplementary Table 14 Kinetic data for the variation of the amount of catalyst.

Entry	[cat] / M x10^{-2}	t / s	[2] / M *Reaction 1*	[2] / M *Reaction 2*	[2] / M *Reaction 3*	Average rate / Ms^{-1} x10^{-4}	Standard deviation / Ms^{-1} x10^{-4}
1	0.5	60	0.0065	0.0071	0.0057	0.477	0.073
2		120	0.0095	0.0101	0.0095		
3		180	0.0123	0.0126	0.0130		
4		240	0.0147	0.0146	0.0164		
5		300	0.0169	0.0174	0.0197		
6	1.0	60	0.0159	0.0156	0.0158	0.898	0.074
7		120	0.0216	0.0215	0.0219		
8		180	0.0269	0.0266	0.0284		
9		240	0.0314	0.0321	0.0342		
10		300	0.0363	0.0358	0.0397		
11	2.0	60	0.0302	0.0303	0.0300	1.478	0.087
12		120	0.0399	0.0414	0.0394		
13		180	0.0502	0.0511	0.0501		

14		240	0.0583	0.0599	0.0551		
15		300	0.0666	0.0677	0.0629		

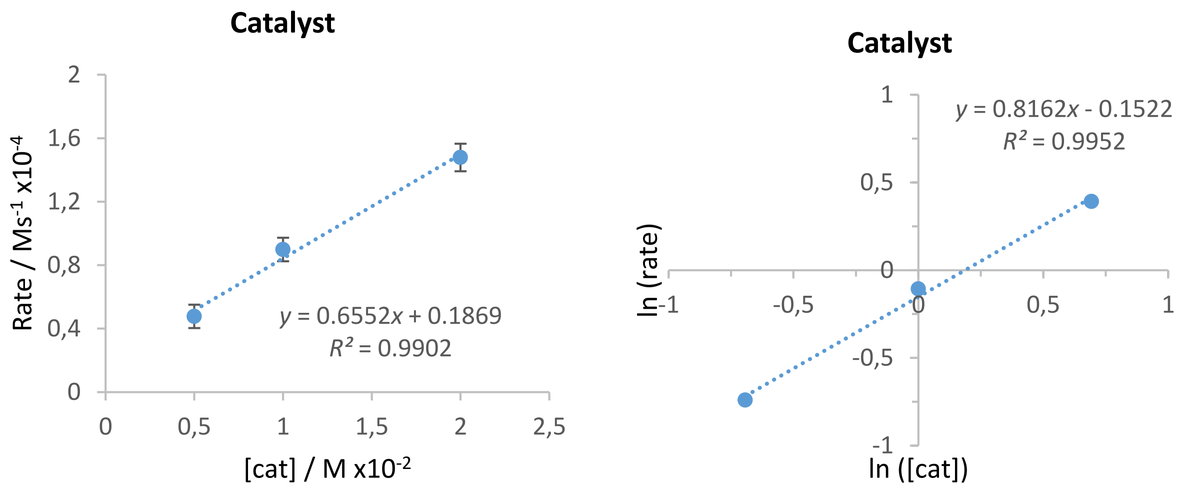

Supplementary Figure 8 Plot of the measured initial rate vs concentration of catalyst (left) and corresponding logarithmic representation (right). Error bars represent the standard deviation of three independent experiments.

1,5-Cyclooctadiene (COD)

Inside an Ar-filled glovebox, an oven-dried 16 mL vial was charged with the corresponding amount of COD and THF (0.5 mL) was added. Thereafter, 1 mL of a stock solution containing $Ni(cod)_2$ (0.05 M) and IPr (0.06 M) in THF was added, followed by 1 mL of a stock solution containing carbonate **1** (0.5 M) and *n*-decane (0.5 M) in THF. After the addition was completed, the reactions were stirred at room temperature and 0.25 mL aliquots were taken every minute for the first 5 minutes, filtered over a plug of silica and analysed via GC.

Supplementary Table 15 Kinetic data for the variation of the amount of added COD.

Entry	[Added COD] / M x10^{-2}	t / s	[2] / M			Average rate / Ms^{-1} x10^{-4}	Standard deviation / Ms^{-1} x10^{-4}
			Reaction 1	*Reaction 2*	*Reaction 3*		
1		60	0.0302	0.0303	0.0300		
2		120	0.0399	0.0414	0.0394		
3	0	180	0.0502	0.0511	0.0501	1.478	0.087
4		240	0.0583	0.0599	0.0551		
5		300	0.0666	0.0677	0.0629		
6		60	0.0268	0.0261	0.0232		
7	1.0	120	0.0350	0.0339	0.0304	1.133	0.022
8		180	0.0414	0.0412	0.0367		
9		240	0.0475	0.0475	0.0430		

10		300	0.0545	0.0541	0.0501		
11		60	0.0259	0.0214	0.0212		
12		120	0.0325	0.0284	0.0273		
13	2.0	180	0.0374	0.0336	0.0321	0.830	0.025
14		240	0.0420	0.0376	0.0363		
15		300	0.0462	0.0426	0.0406		
16		60	0.0207	0.0205	0.0199		
17		120	0.0250	0.0240	0.0240		
18	4.0	180	0.0282	0.0268	0.0263	0.442	0.046
19		240	0.0305	0.0295	0.0289		
20		300	0.0326	0.0315	0.0288		

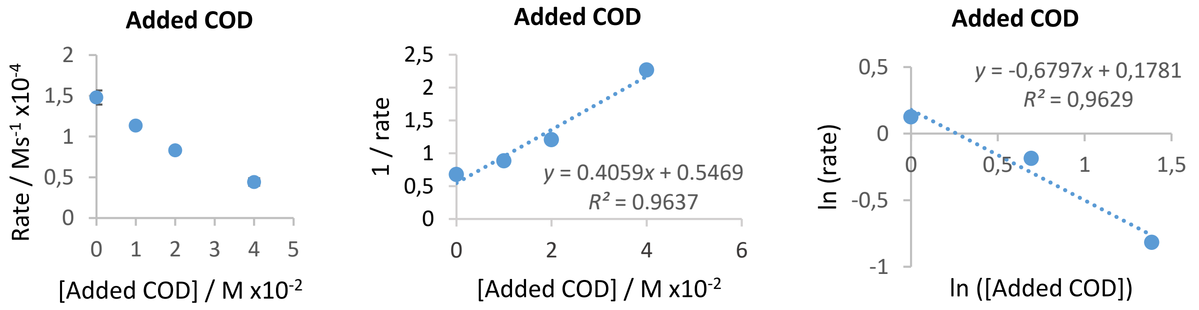

Supplementary Figure 9 Plot of the measured initial rate vs concentration of added COD (left), plot of the inverse of the measured initial rate vs concentration of added COD (middle) and plot of the ln of the measured initial rate vs ln of the concentration of added COD (right). Error bars represent the standard deviation of three independent experiments.

Acetone

Inside an Ar-filled glovebox, an oven-dried 16 mL vial was charged with the corresponding amount of acetone and THF (0.5 mL) was added. Thereafter, 1 mL of a stock solution containing Ni(cod)$_2$ (0.05 M) and IPr (0.06 M) in THF was added, followed by 1 mL of a stock solution containing carbonate **1** (0.5 M) and *n*-decane (0.5 M) in THF. After the addition was completed, the reactions were stirred at room temperature and 0.25 mL aliquots were taken every minute for the first 5 minutes, filtered over a plug of silica and analysed via GC.

Supplementary Table 16 Kinetic data for the variation of the amount of added acetone.

**Entry**	**[Added acetone] / M x10^{-2}**	**t / s**	**[2] / M**			**Average rate / Ms^{-1} x10^{-4}**	**Standard deviation / Ms^{-1} x10^{-4}**
			Reaction 1	*Reaction 2*	*Reaction 3*		
1		60	0.0302	0.0303	0.0300		
2	0	120	0.0399	0.0414	0.0394	1.478	0.087
3		180	0.0502	0.0511	0.0501		
4		240	0.0583	0.0599	0.0551		

5		300	0.0666	0.0677	0.0629		
6		60	0.0225	0.0221	0.0282		
7		120	0.0303	0.0307	0.0352		
8	1.0	180	0.0366	0.0372	0.0409	1.078	0.055
9		240	0.0424	0.0437	0.0471		
10		300	0.0486	0.0501	0.0528		
11		60	0.0221	0.0217	0.0262		
12		120	0.0297	0.0292	0.0339		
13	2.0	180	0.0355	0.0354	0.0386	0.973	0.074
14		240	0.0410	0.0412	0.0436		
15		300	0.0463	0.0472	0.0476		
16		60	0.0216	0.0215	0.0277		
17		120	0.0295	0.0298	0.0338		
18	4.0	180	0.0352	0.0359	0.0399	1.012	0.097
19		240	0.0405	0.0423	0.0451		
20		300	0.0472	0.0488	0.0486		
21		60	0.0138	0.0139	0.0168		
22		120	0.0221	0.0218	0.0227		
23	6.0	180	0.0281	0.0276	0.0292	0.997	0.054
24		240	0.0344	0.0322	0.0341		
25		300	0.0398	0.0372	0.0401		

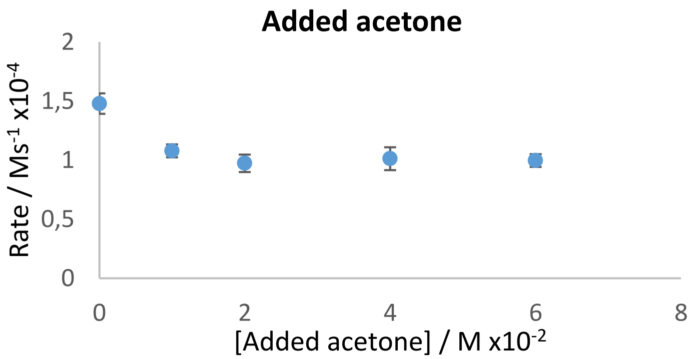

Supplementary Figure 10 Plot of the measured initial rate vs concentration of added acetone. Error bars represent the standard deviation of three independent experiments.

Acetone-d_6

Inside an Ar-filled glovebox, an oven-dried 16 mL vial was charged with the corresponding amount of acetone-d_6 and THF (0.5 mL) was added. Thereafter, 1 mL of a stock solution containing Ni(cod)$_2$ (0.05 M) and IPr (0.06 M) in THF was added, followed by 1 mL of a stock solution containing carbonate **1** (0.5 M) and *n*-decane (0.5 M) in THF. After the addition was completed, the reactions were stirred at room temperature and 0.25 mL aliquots were taken every minute for the first 5 minutes, filtered over a plug of silica and analysed via GC.

Supplementary Table 17 Kinetic data for the variation of the amount of added acetone-d_6.

Entry	[Added acetone-d_6] / M x10^{-2}	t / s	[2] / M			Average rate / Ms^{-1} x10^{-4}	Standard deviation / Ms^{-1} x10^{-4}
			Reaction 1	*Reaction 2*	*Reaction 3*		
1	0	60	0.0302	0.0303	0.0300	1.478	0.087
2		120	0.0399	0.0414	0.0394		
3		180	0.0502	0.0511	0.0501		
4		240	0.0583	0.0599	0.0551		
5		300	0.0666	0.0677	0.0629		
6	1.0	60	0,0215	0,0216	0.0220	0.942	0.038
7		120	0,0288	0,0288	0.0300		
8		180	0,0347	0,0340	0.0348		
9		240	0,0396	0.0385	0.0398		
10		300	0,0456	0.0435	0.0456		
11	2.0	60	0,0213	0,0223	0.0240	0.856	0.021
12		120	0,0278	0,0277	0.0285		
13		180	0,0327	0,0337	0.0341		
14		240	0,0379	0,0394	0.0390		
15		300	0,0426	0,0422	0.0436		
16	4.0	60	0.0235	0.0238	0.0228	0.825	0.067
17		120	0.0322	0.0288	0.0269		
18		180	0.0354	0.0329	0.0328		
19		240	0.0405	0.0375	0.0371		
20		300	0.0462	0.0416	0.0430		
21	6.0	60	0.0228	0.0226	0.0212	0.859	0.023
22		120	0.0308	0.0283	0.0293		
23		180	0.0371	0.0327	0.0356		
24		240	0.0387	0.0380	0.0378		
25		300	0.0438	0.0434	0.0436		

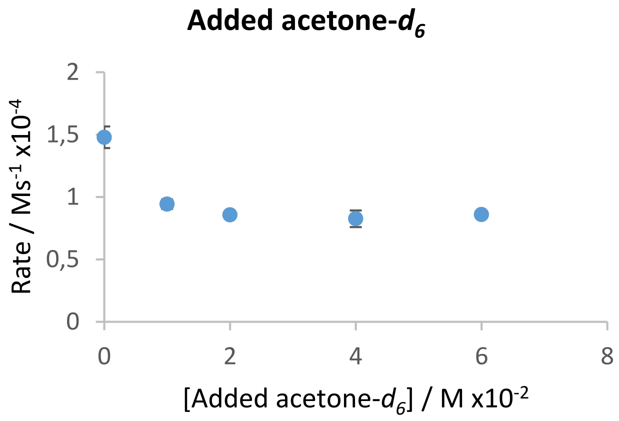

Supplementary Figure 11 Plot of the measured initial rate vs concentration of added acetone-d_6. Error bars represent the standard deviation of three independent experiments.

Primary kinetic isotope effect (KIE)

Inside an Ar-filled glovebox, an oven-dried 16 mL vial was charged with carbonate **3** (0.5 mmol, 1.0 equiv.) *or* carbonate **4** (0.5 mmol, 1.0 equiv.), $Ni(cod)_2$ (10 mol%), and IPr (12 mol%). *n*-Decane (1.0 equiv.) was added as an internal standard followed by THF (2.5 mL). After the addition was completed, the reaction was stirred at room temperature and 0.25 mL aliquots were taken every minute for the first 5 minutes, filtered over a plug of silica and analysed via GC.

Supplementary Table 18 Kinetic data and corresponding primary kinetic isotope effects.

Entry	t / s	Reaction 1		Reaction 2		Reaction 3	
		[2] / M	[2-*d*] / M	[2] / M	[2-*d*] / M	[2] / M	[2-*d*] / M
1	60	0.0240	0.0155	0.0233	0.0196	0.0282	0.0175
2	120	0.0325	0.0199	0.0309	0.0238	0.0363	0.0211
3	180	0.0393	0.0231	0.0377	0.0266	0.0444	0.0251
4	240	0.0458	0.0265	0.0429	0.0304	0.0514	0.0291
5	300	0.0522	0.0304	0.0486	0.0337	0.0572	0.0318
**KIE**		1.917		1.795		1.993	
**Average KIE**		1.902					
**Standard deviation**		0.082					

β-Secondary kinetic isotope effect

Inside an Ar-filled glovebox, an oven-dried 16 mL vial was charged with the corresponding amount of acetone *or* acetone-d_6 and THF (0.5 mL) was added. Thereafter, 1 mL of a stock solution containing $Ni(cod)_2$ (0.05 M) and IPr (0.06 M) in THF was added, followed by 1 mL of a stock solution containing carbonate **1** (0.5 M) and *n*-decane (0.5 M) in THF. After the addition was completed, the reactions were stirred at room temperature and 0.25 mL aliquots were taken every minute for the first 5 minutes, filtered over a plug of silica and analysed via GC.

Supplementary Table 19 Secondary kinetic isotope effects for various concentrations of added acetone/acetone-d_6 and overall average secondary isotope effect.

Entry	[Added acetone or acetone-d_6] / M x 10^{-2}	Reaction 1	Reaction 2	Reaction 3	Average KIE	Standard deviation
		KIE	KIE	KIE		
1	1.0	1.086	1.287	1.070	1.145	0.098
2	2.0	1.132	1.267	1.016	1.138	0.103
3	4.0	1.157	1.521	1.045	1.241	0.203
4	6.0	1.288	1.110	1.089	1.162	0.089
**Overall average KIE**				1.172		
**Standard deviation**				0.123		

External oxidant-free oxidation in the presence of alcohols, thiols and amines

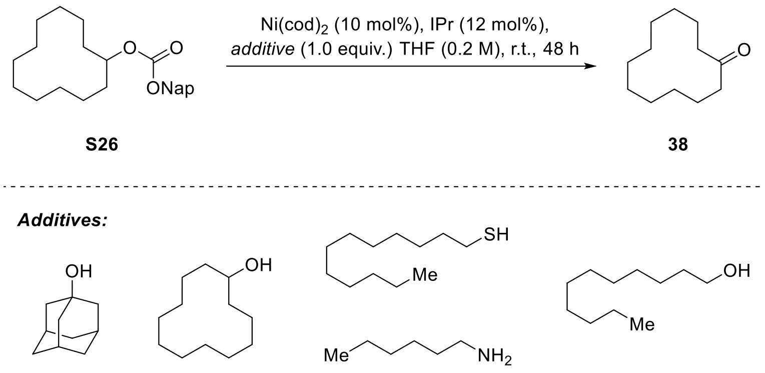

Inside an Ar-filled glovebox, an oven-dried 8 mL vial was charged with carbonate **S26** (0.1 mmol), and the corresponding additive (1.0 equiv.). Thereafter, 0.5 mL of a stock solution containing Ni(cod)$_2$ (0.02 M) and IPr (0.024 M) in THF was added and the vial was sealed. The reaction mixture was stirred at room temperature for 48 h. After that, the vial was decapped, *n*-decane was added as an internal standard, the mixture was diluted with EtOAc and an aliquot was taken for GC analysis.

Supplementary Table 20 External oxidant-free oxidation in the presence of alcohols, thiols and amines.

Entry	Additive	Yield 38 / %
1	adamantan-1-ol	91
2	cyclododecanol	>95
3	undecan-1-ol	13
4	dodecane-1-thiol	27
5	hexan-1-amine	40

Appendix

X-Ray data

Compound 49

Single crystals of compound **49** were obtained by slow evaporation of a THF solution of **49** after 72 h at room temperature. Note on the crystal structure: two symmetrically independent molecules in the asymetric unit.

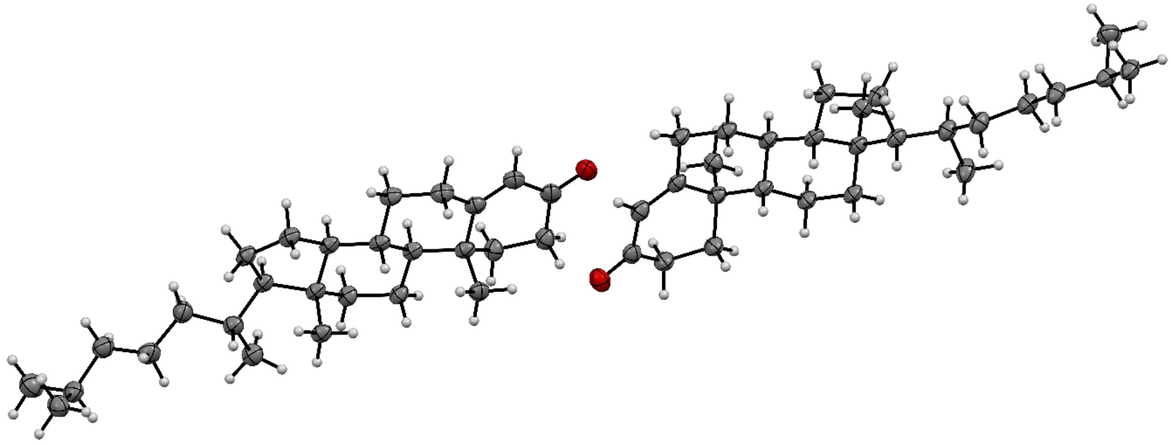

Supplementary Figure 12 Asymetric unit of the crystal structure of compound **49**.

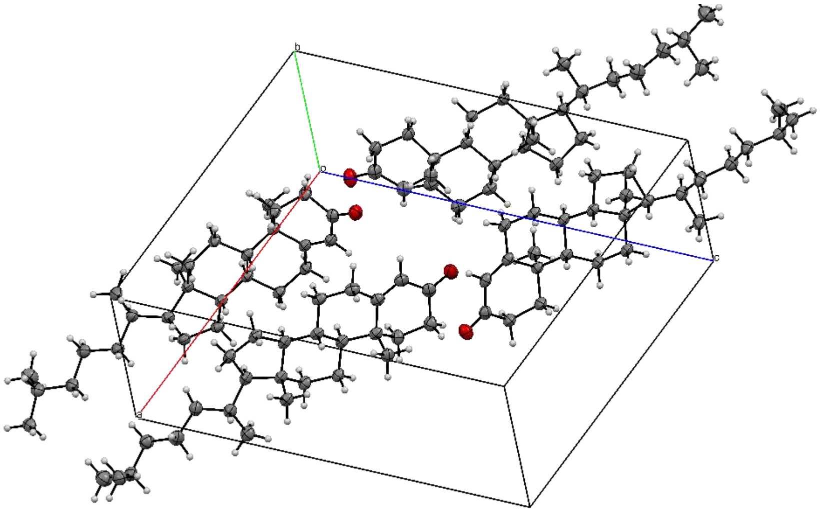

Supplementary Figure 13 Packing of the crystal structure of compound **49**.

Supplementary Table 21 Structure table of compound **49**.

	**bm260322_1_1.cif**
CCDC number	2220801
Empirical formula	$C_{27}H_{44}O$
Formula weight	384.62
Temperature [K]	100.0(1)
Crystal system	monoclinic
Space group (number)	$P2_1$ (4)
a [Å]	14.2181(3)
b [Å]	9.6078(2)
c [Å]	17.8085(3)
α [Å]	90
β [Å]	104.807(2)
γ [Å]	90
Volume [$Å^3$]	2351.94(8)
Z	4
ρ_{calc} [g/cm^3]	1.086
μ [mm^{-1}]	0.469
F(000)	856
Crystal size [mm^3]	0.208×0.092×0.067
Crystal colour	clear colourless
Crystal shape	block
Radiation	Cu K_α (λ=1.54184 Å)
2Θ range [°]	5.13 to 159.89 (0.78 Å)
Index ranges	-16 ≤ h ≤ 17<br>-12 ≤ k ≤ 12<br>-22 ≤ l ≤ 22
Reflections collected	28951

Independent reflections	9853<br>R_{int} = 0.0544<br>R_{sigma} = 0.0470
Completeness	100.0 %
Data / Restraints / Parameters	9853/1/515
Goodness-of-fit on F^2	1.023
Final R indexes [I≥2σ(I)]	R_1 = 0.0617<br>wR_2 = 0.1730
Final R indexes [all data]	R_1 = 0.0653<br>wR_2 = 0.1779
Largest peak/hole [eÅ^3]	0.42/-0.33
Flack X parameter	-0.35(18)

NMR Spectra

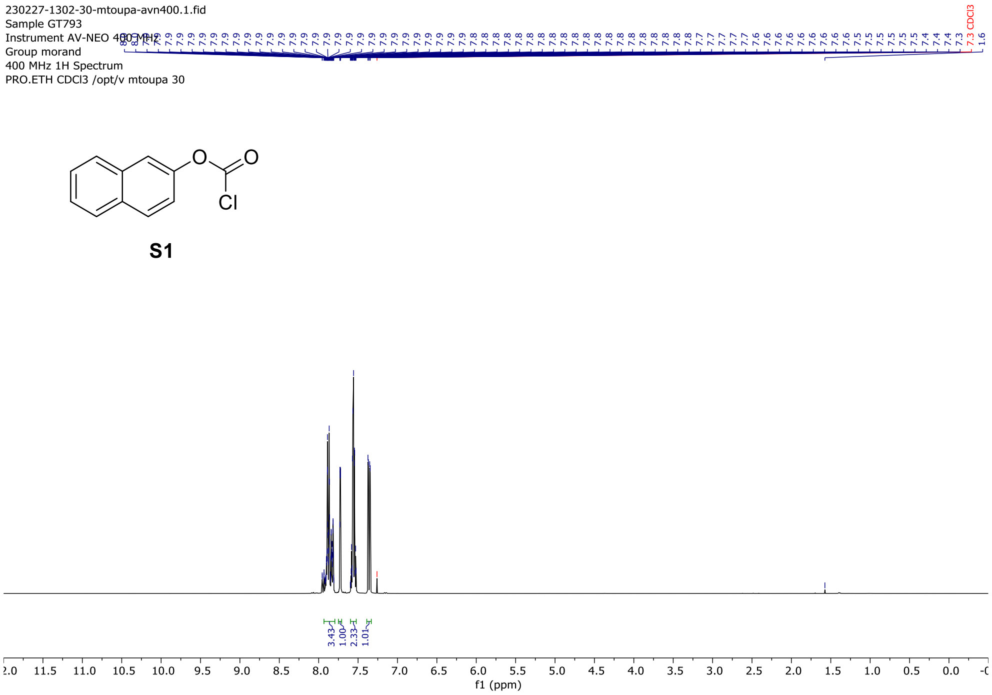

Supplementary Figure 14 ^{1}H NMR (400 MHz, $CDCl_3$, 25 °C) of compound **S1.**

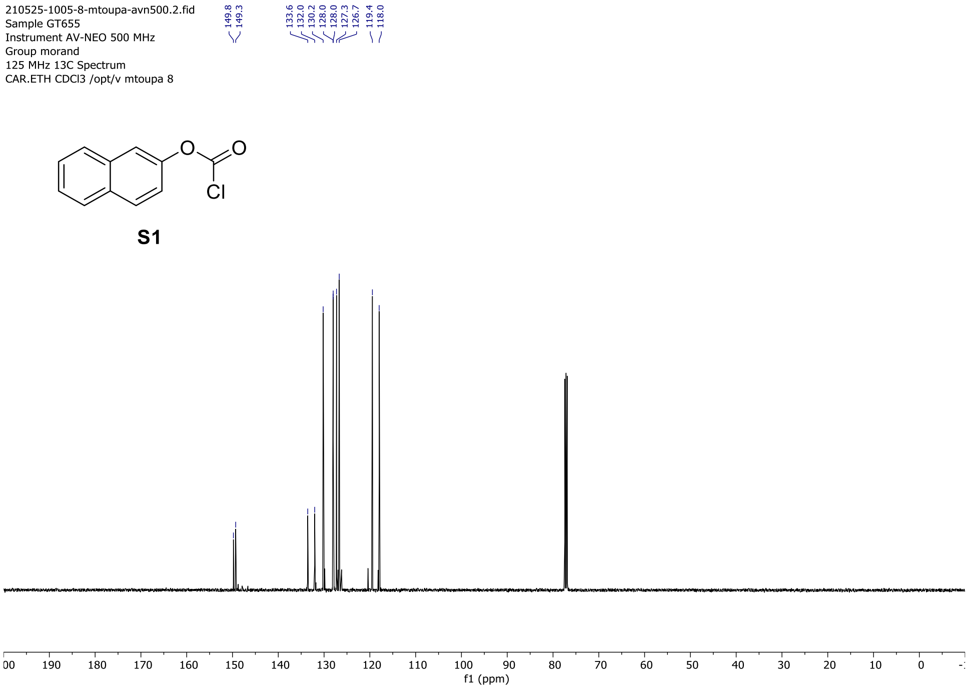

Supplementary Figure 15 ^{13}C NMR (125 MHz, $CDCl_3$, 25 °C) of compound **S1.**

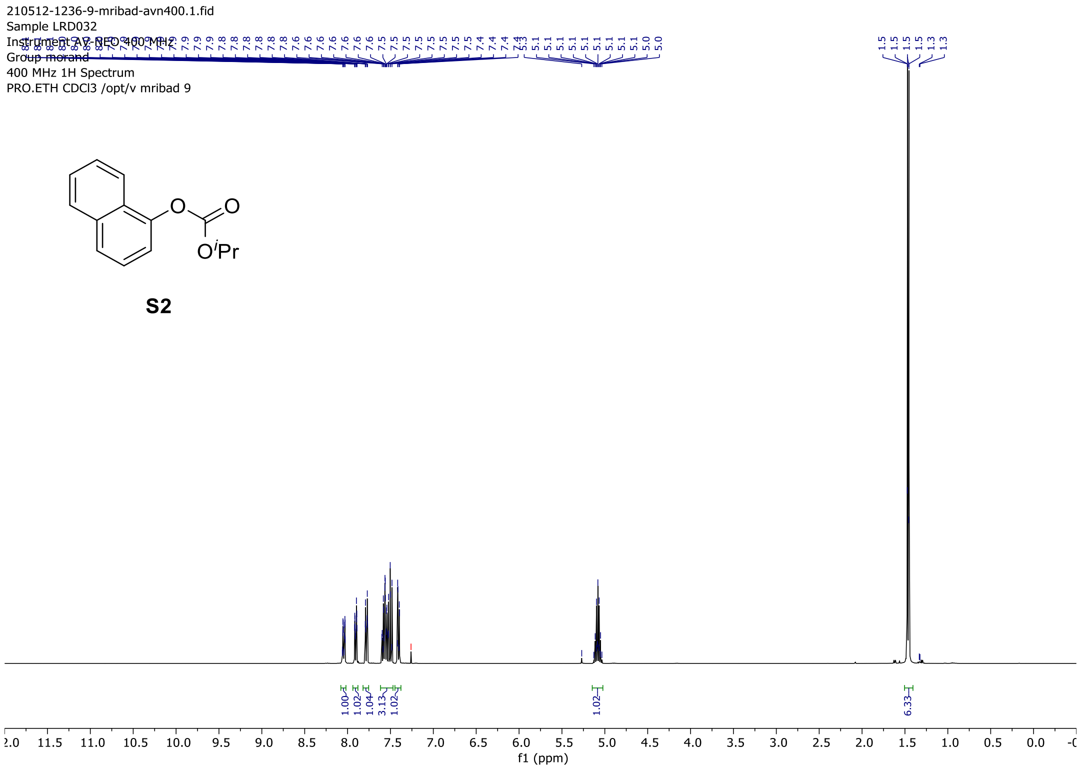

Supplementary Figure 16 ^{1}H NMR (400 MHz, $CDCl_3$, 25 °C) of compound **S2.**

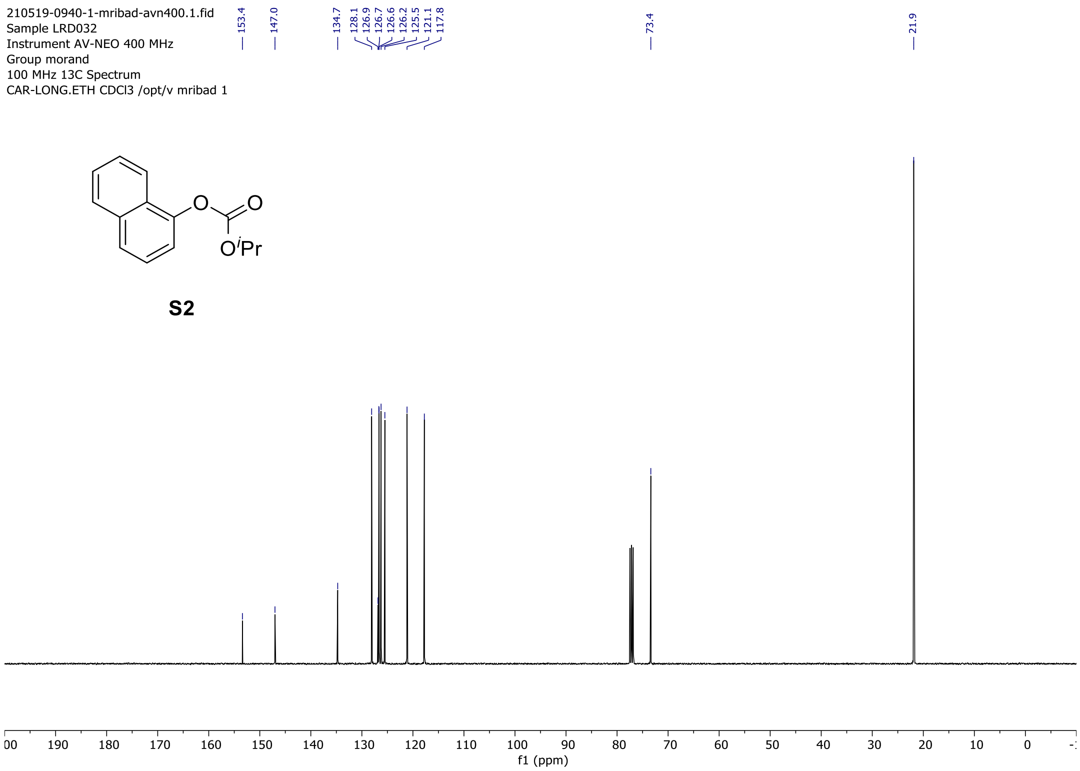

Supplementary Figure 17 ^{13}C NMR (100 MHz, $CDCl_3$, 25 °C) of compound **S2.**

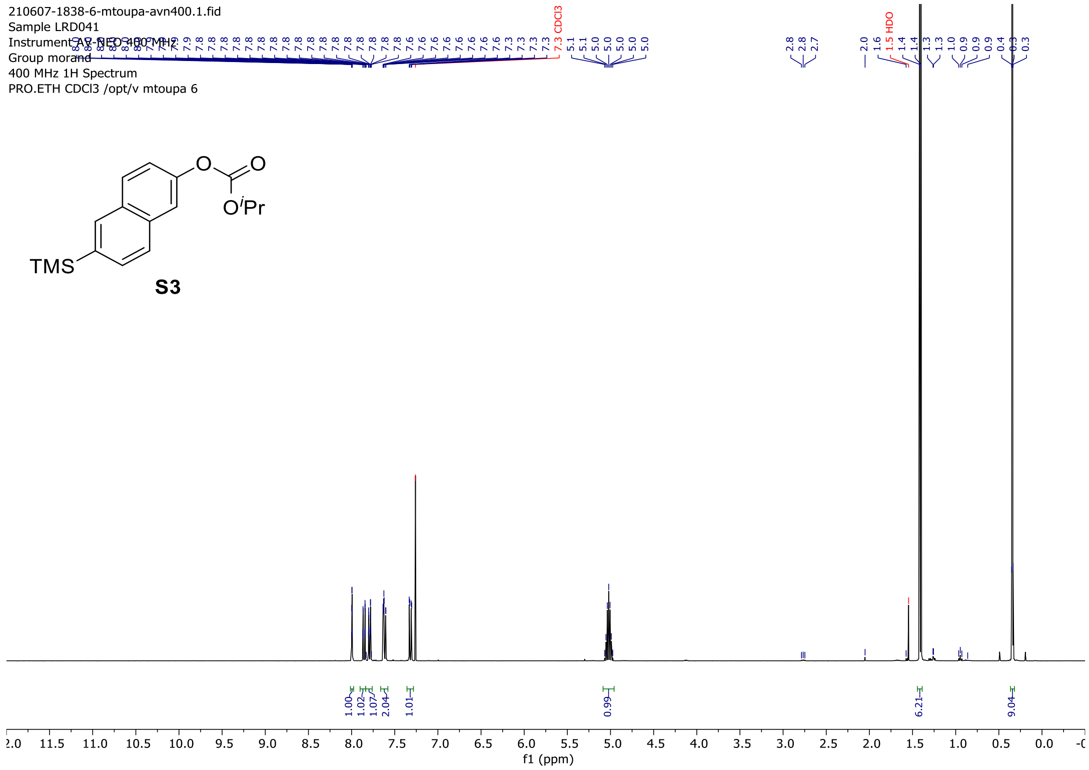

Supplementary Figure 18 ^{13}C NMR (400 MHz, $CDCl_3$, 25 °C) of compound **S3.**

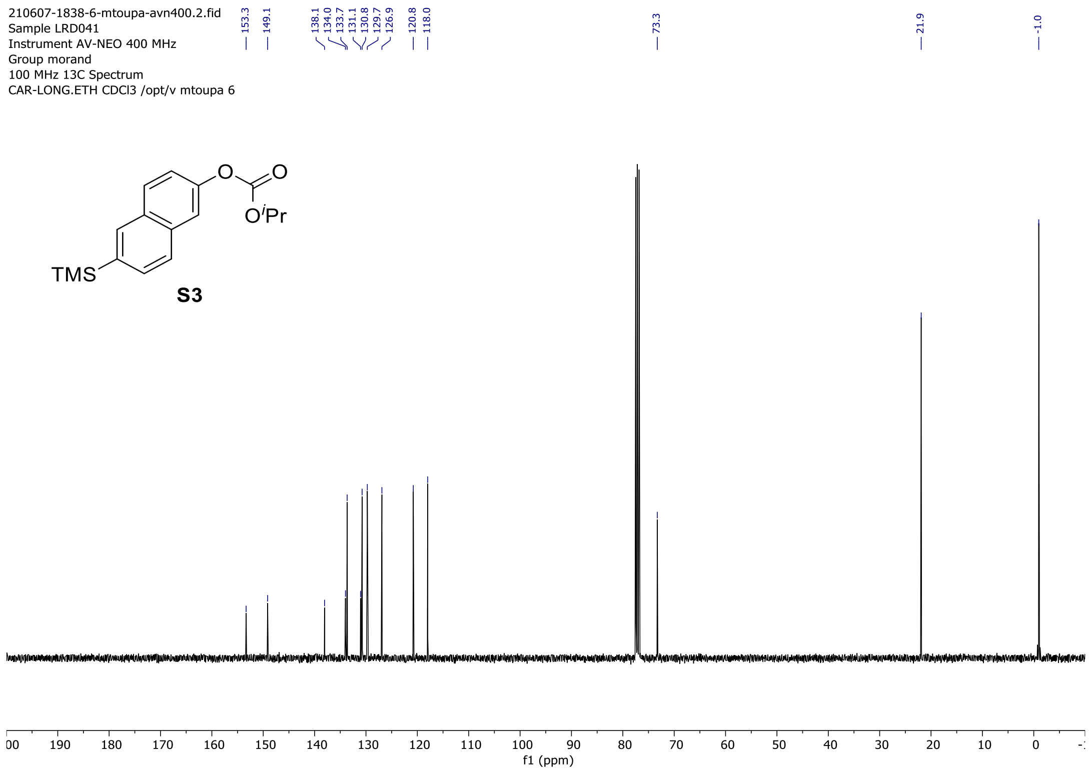

Supplementary Figure 19 ^{13}C NMR (100 MHz, $CDCl_3$, 25 °C) of compound **S3.**

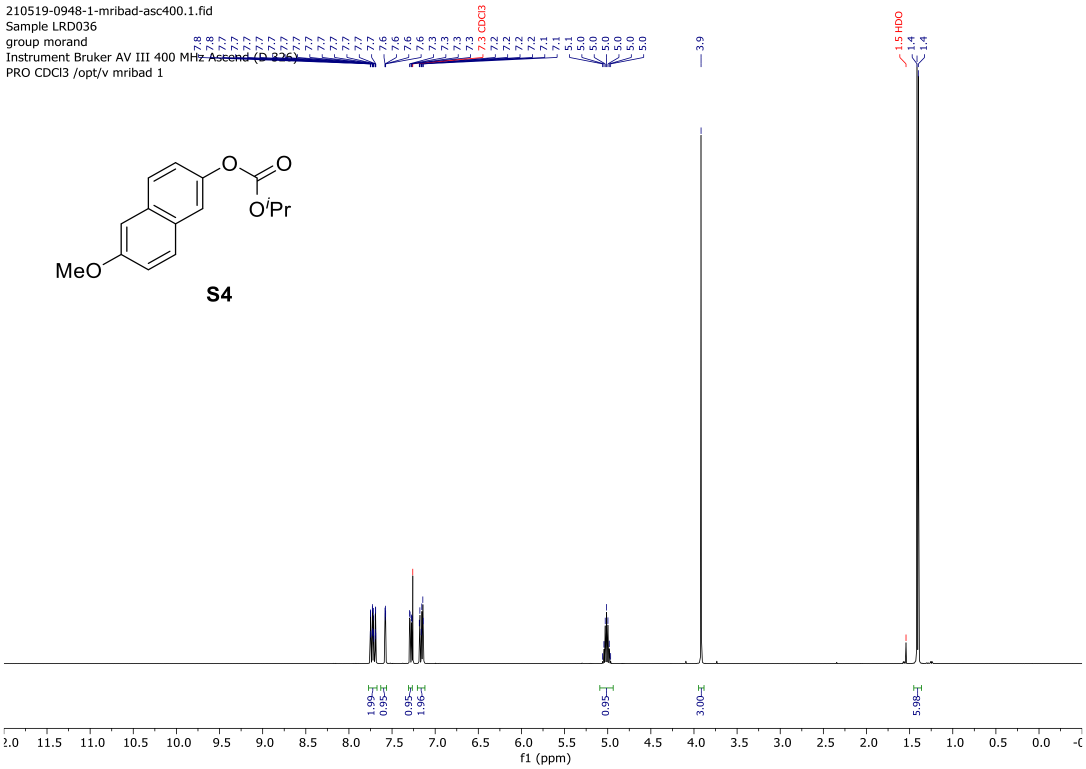

Supplementary Figure 20 ^{1}H NMR (400 MHz, $CDCl_3$, 25 °C) of compound **S4.**

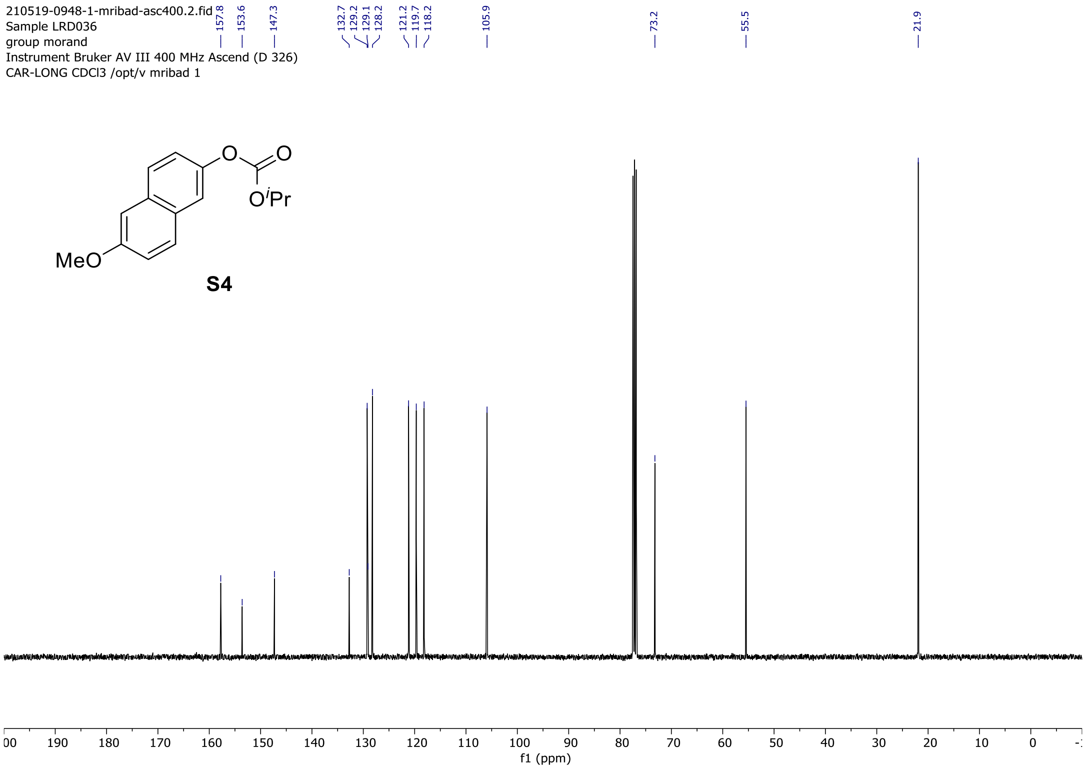

Supplementary Figure 21 ^{13}C NMR (100 MHz, $CDCl_3$, 25 °C) of compound **S4.**

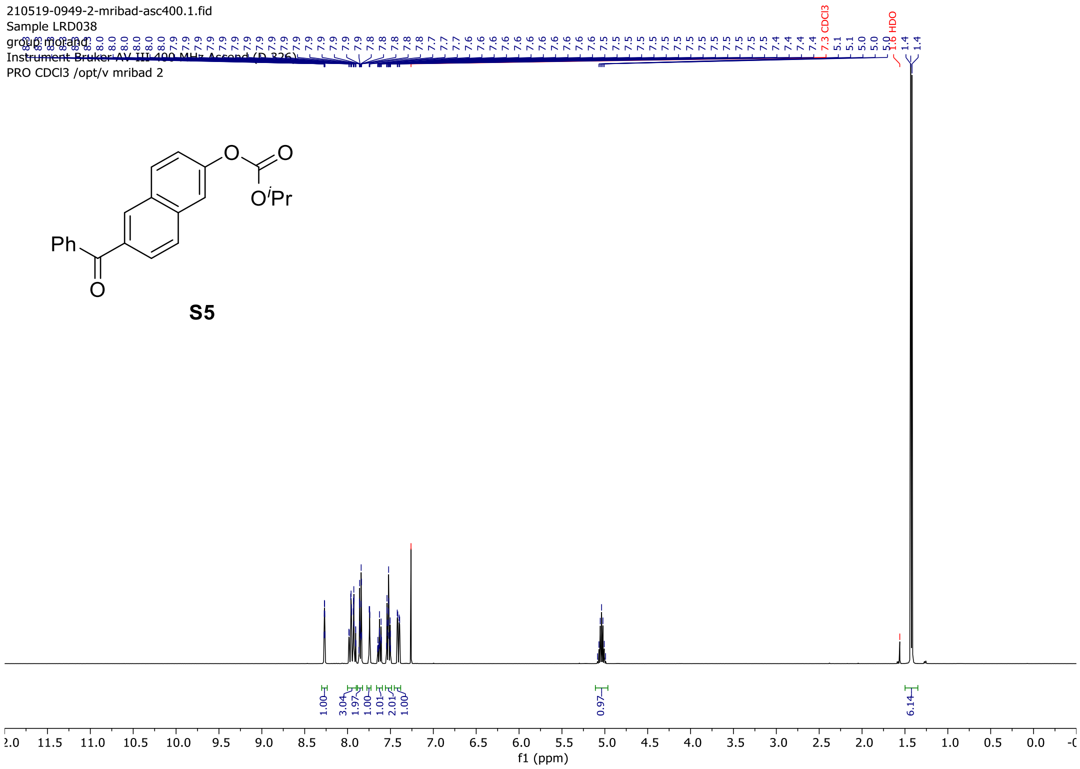

Supplementary Figure 22 ^{1}H NMR (400 MHz, $CDCl_3$, 25 °C) of compound **S5.**

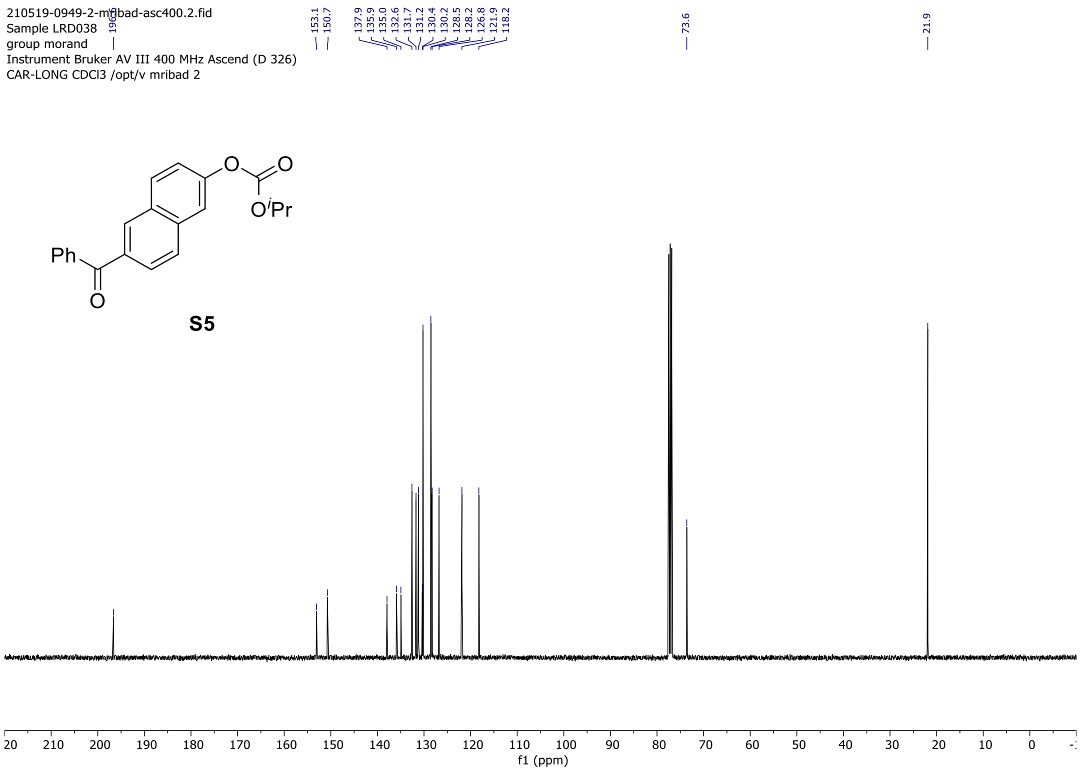

Supplementary Figure 23 ^{13}C NMR (100 MHz, $CDCl_3$, 25 °C) of compound **S5.**

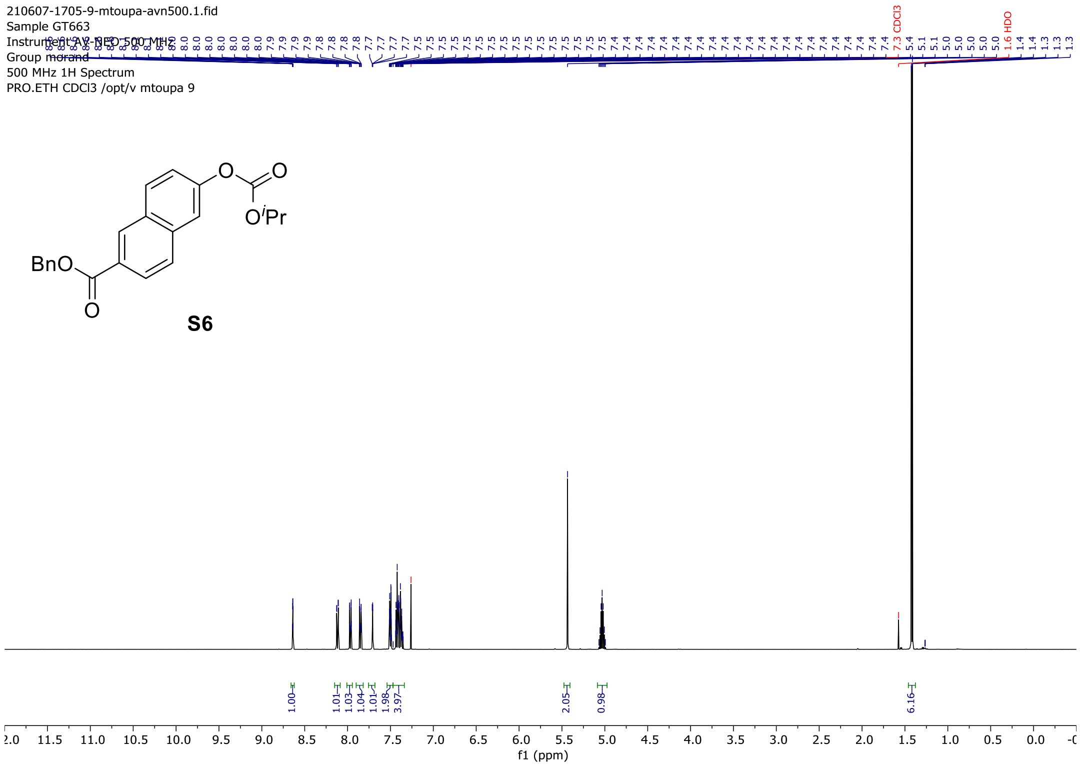

Supplementary Figure 24 ^{1}H NMR (500 MHz, $CDCl_3$, 25 °C) of compound **S6.**

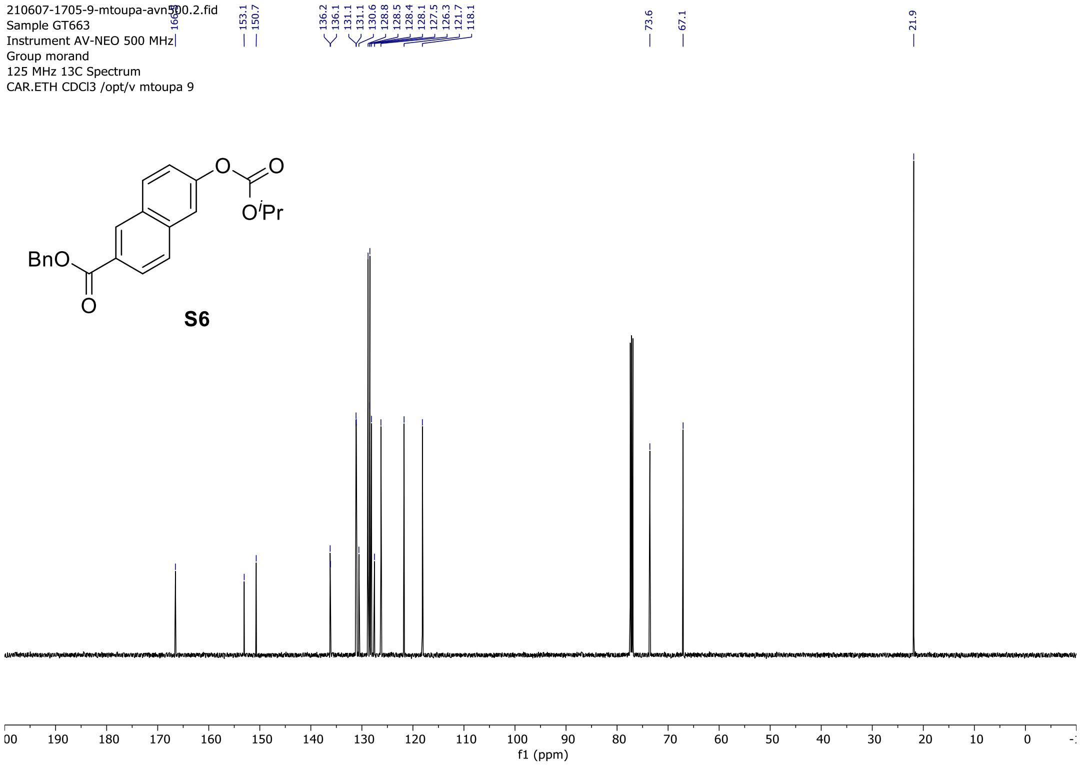

Supplementary Figure 25 ^{13}C NMR (125 MHz, $CDCl_3$, 25 °C) of compound **S6.**

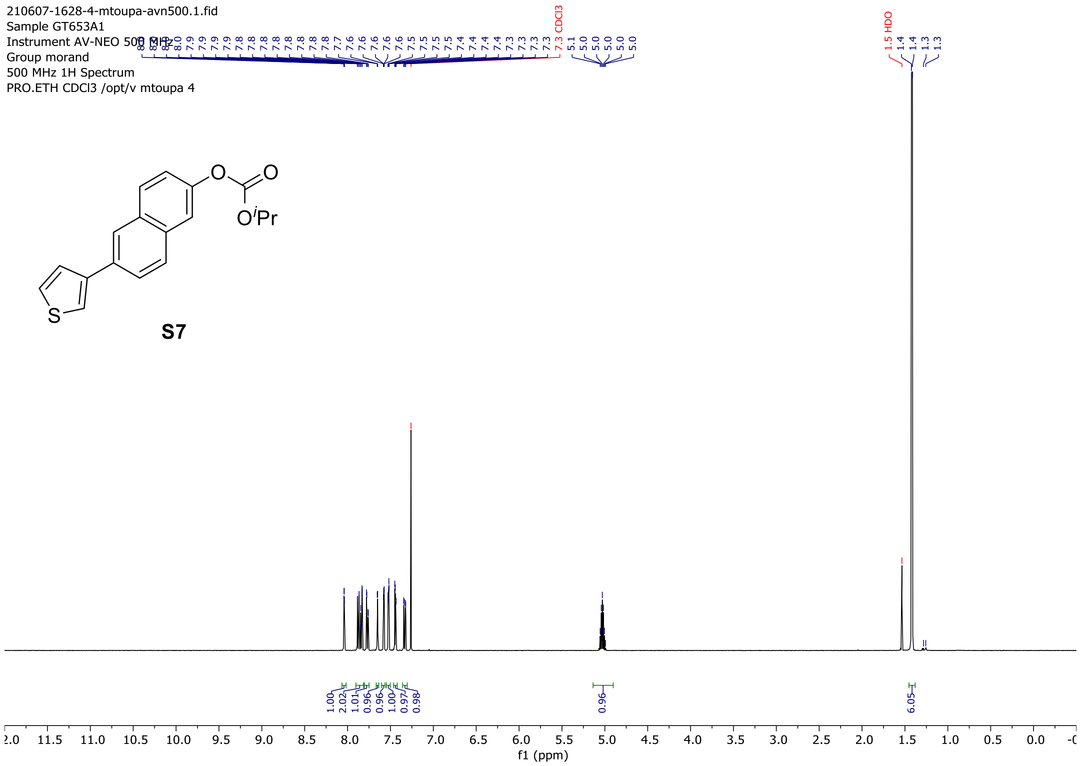

Supplementary Figure 26 ^{1}H NMR (500 MHz, $CDCl_3$, 25 °C) of compound **S7.**

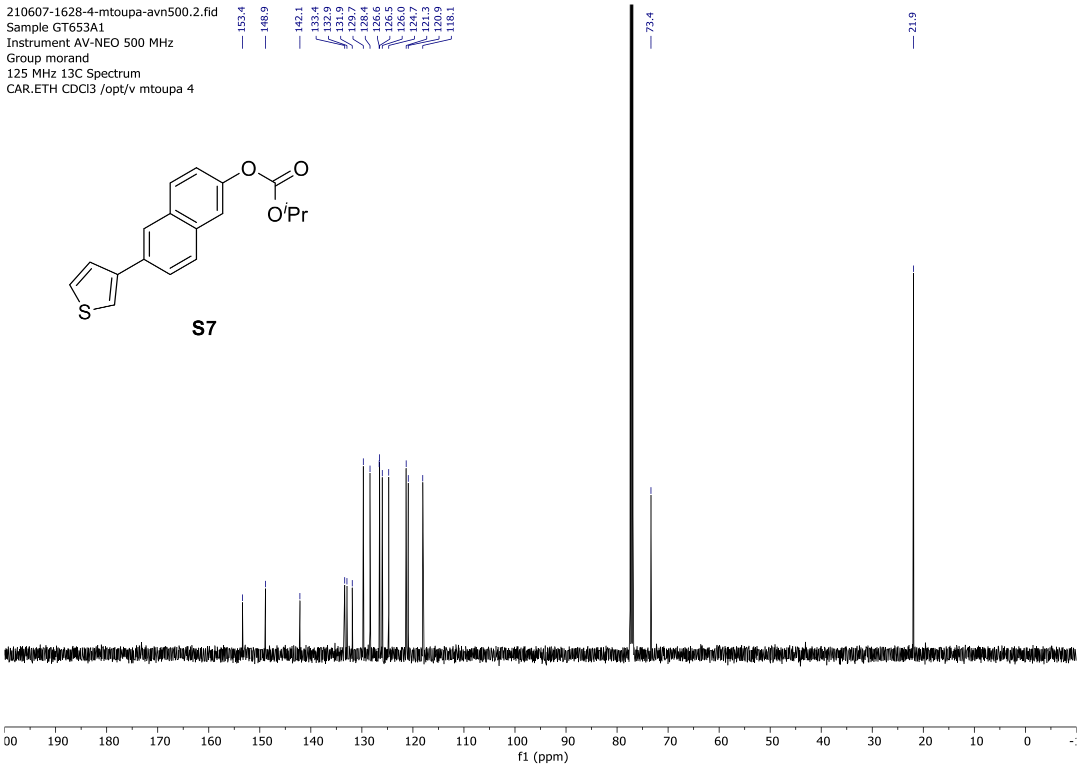

Supplementary Figure 27 ^{13}C NMR (125 MHz, $CDCl_3$, 25 °C) of compound **S7.**

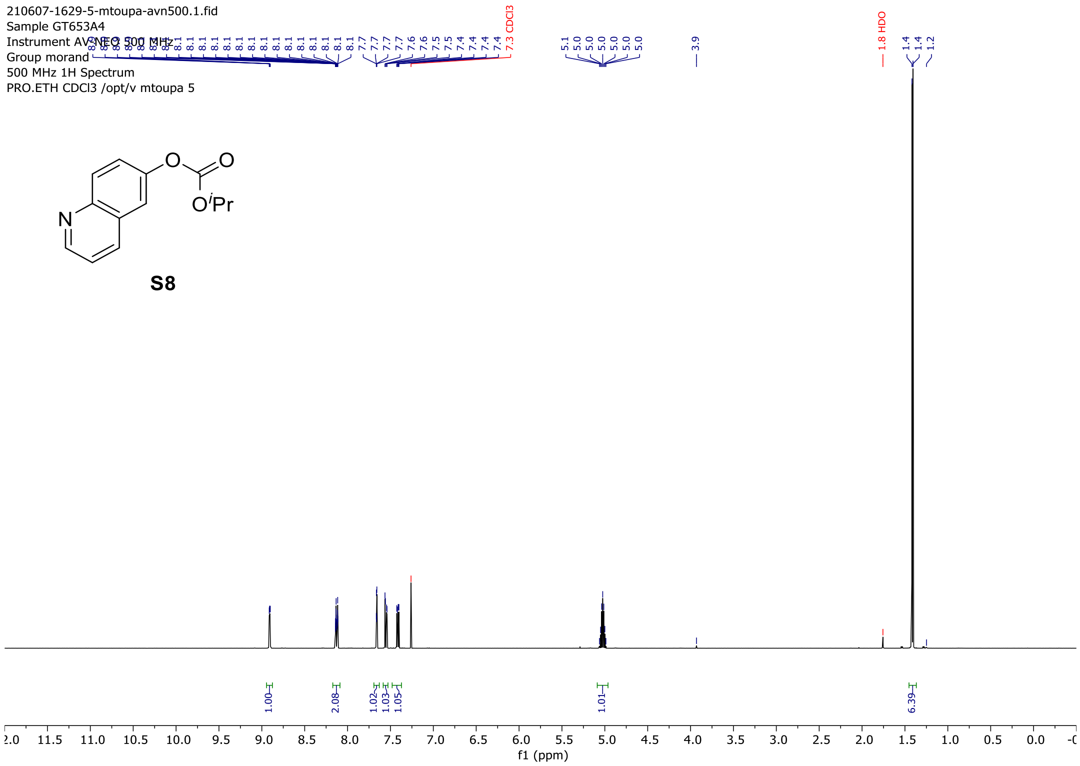

Supplementary Figure 28 ^{1}H NMR (500 MHz, $CDCl_3$, 25 °C) of compound **S8.**

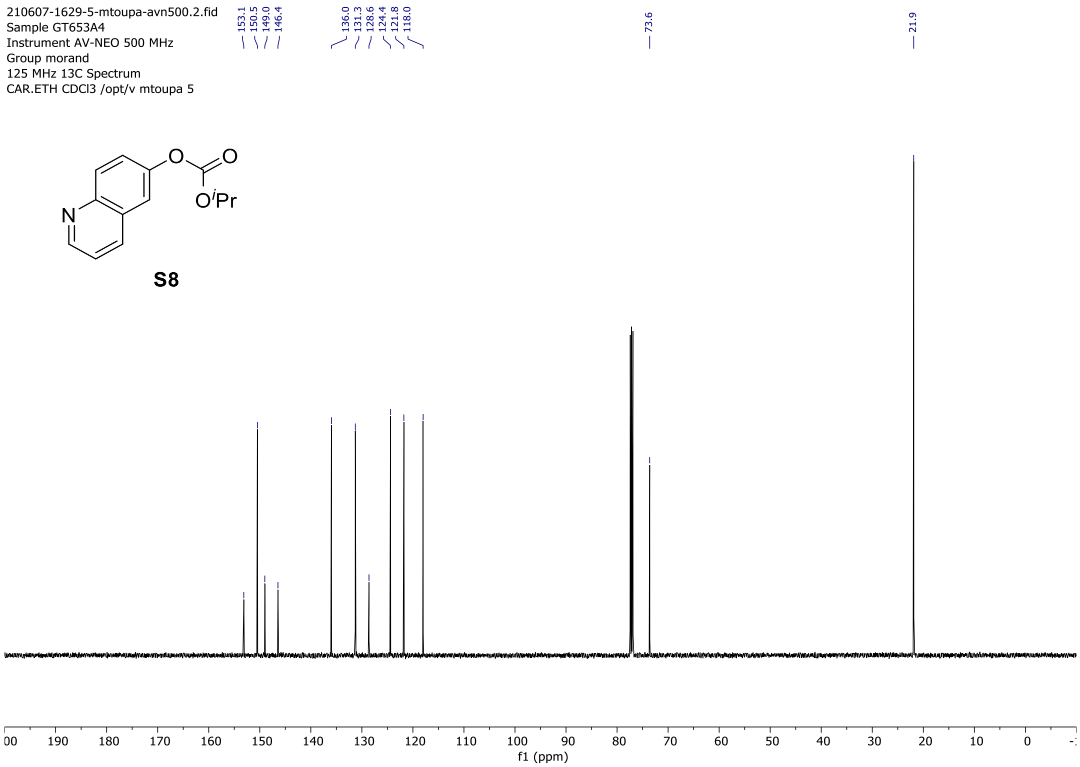

Supplementary Figure 29 ^{13}C NMR (125 MHz, $CDCl_3$, 25 °C) of compound **S8.**

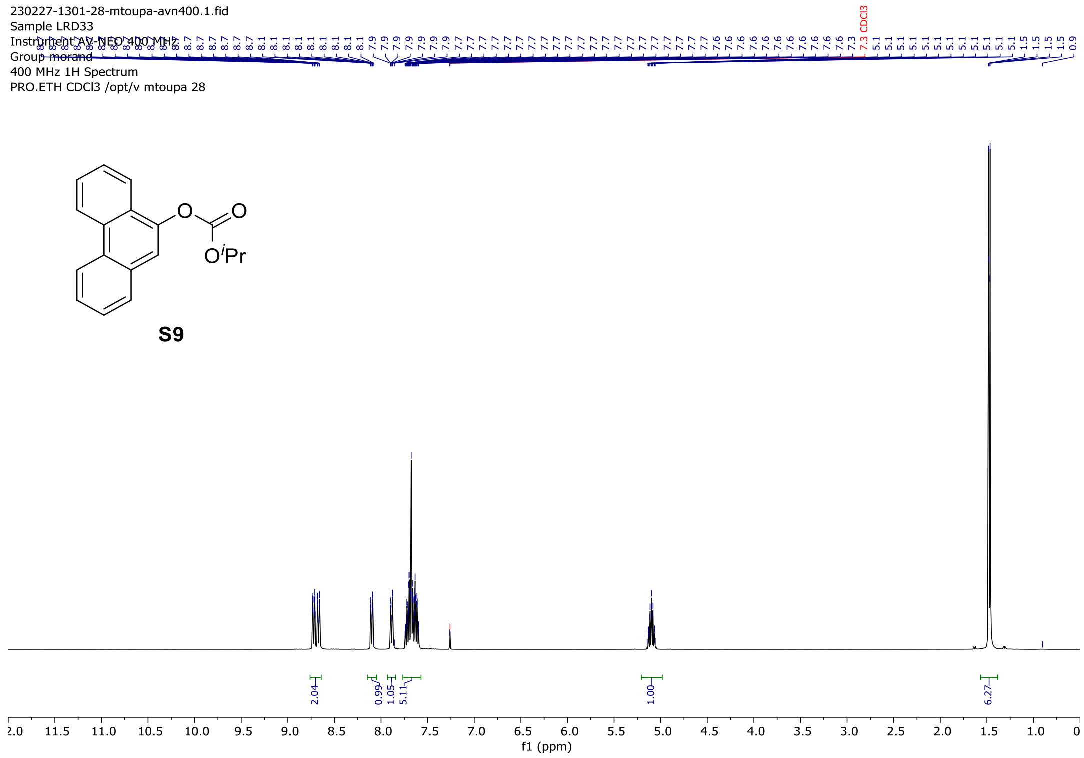

Supplementary Figure 30 ^{1}H NMR (400 MHz, $CDCl_3$, 25 °C) of compound **S9.**

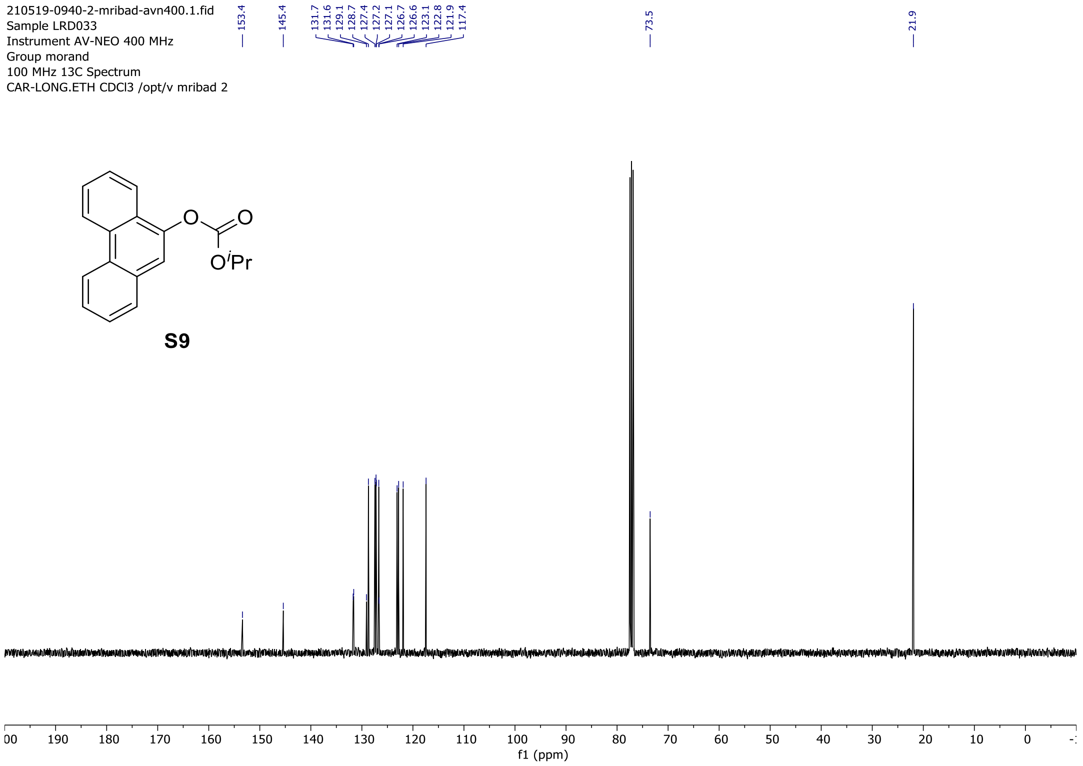

Supplementary Figure 31 ^{13}C NMR (100 MHz, $CDCl_3$, 25 °C) of compound **S9.**

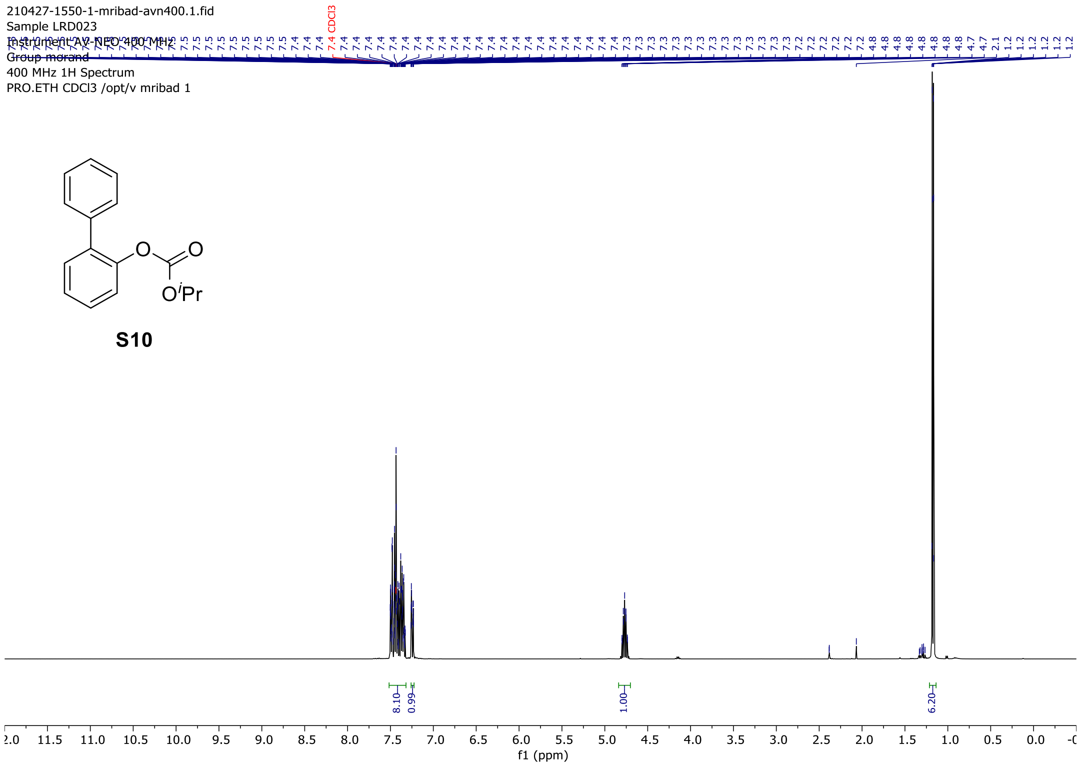

Supplementary Figure 32 ^{1}H NMR (400 MHz, $CDCl_3$, 25 °C) of compound **S10.**

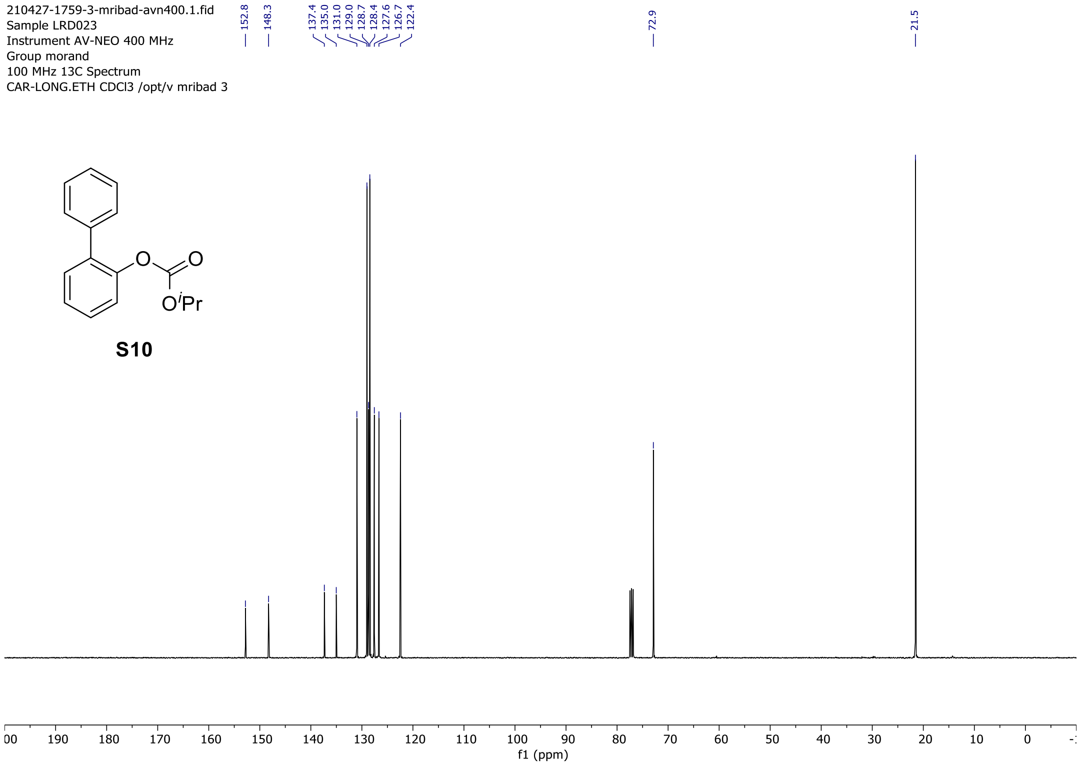

Supplementary Figure 33 ^{13}C NMR (100 MHz, $CDCl_3$, 25 °C) of compound **S10.**

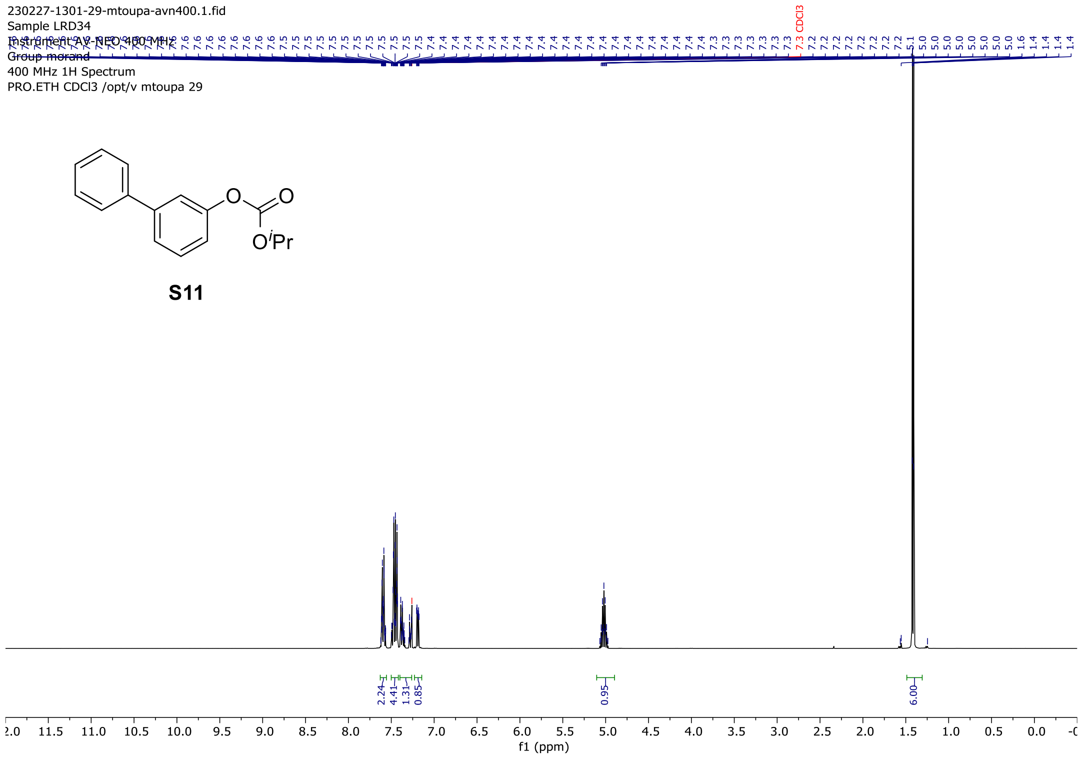

Supplementary Figure 34 ^{1}H NMR (400 MHz, $CDCl_3$, 25 °C) of compound **S11.**

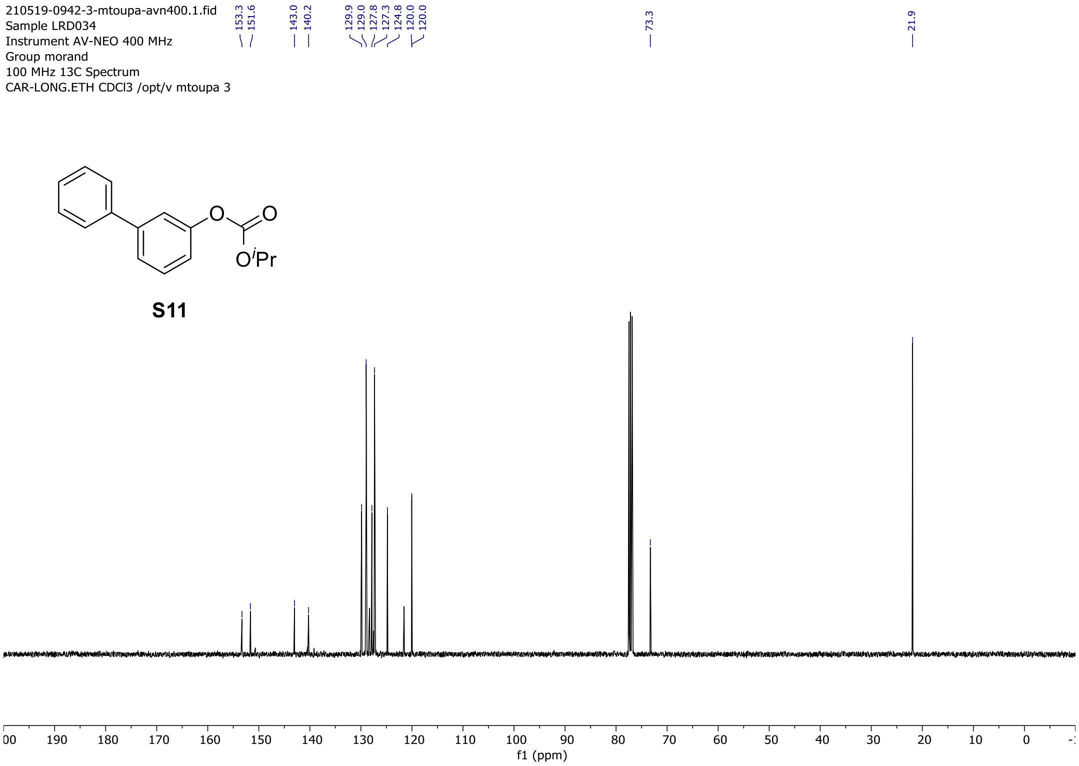

Supplementary Figure 35 ^{13}C NMR (100 MHz, $CDCl_3$, 25 °C) of compound **S11.**

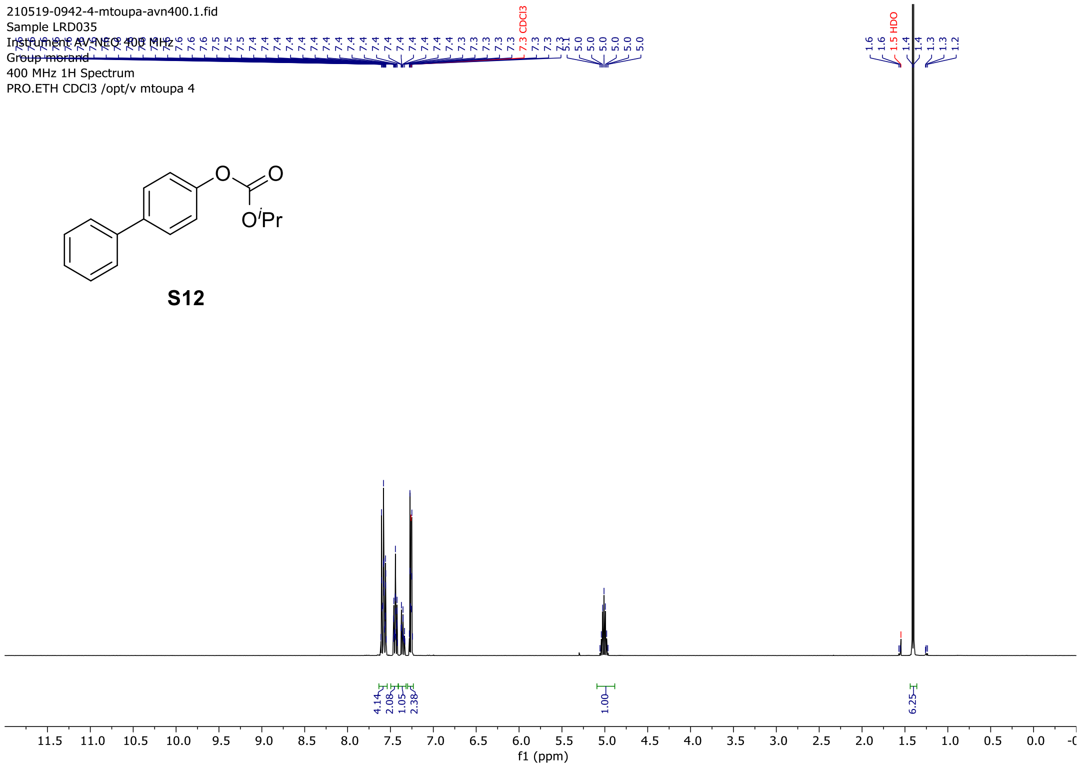

Supplementary Figure 36 ^{1}H NMR (400 MHz, $CDCl_3$, 25 °C) of compound **S12.**

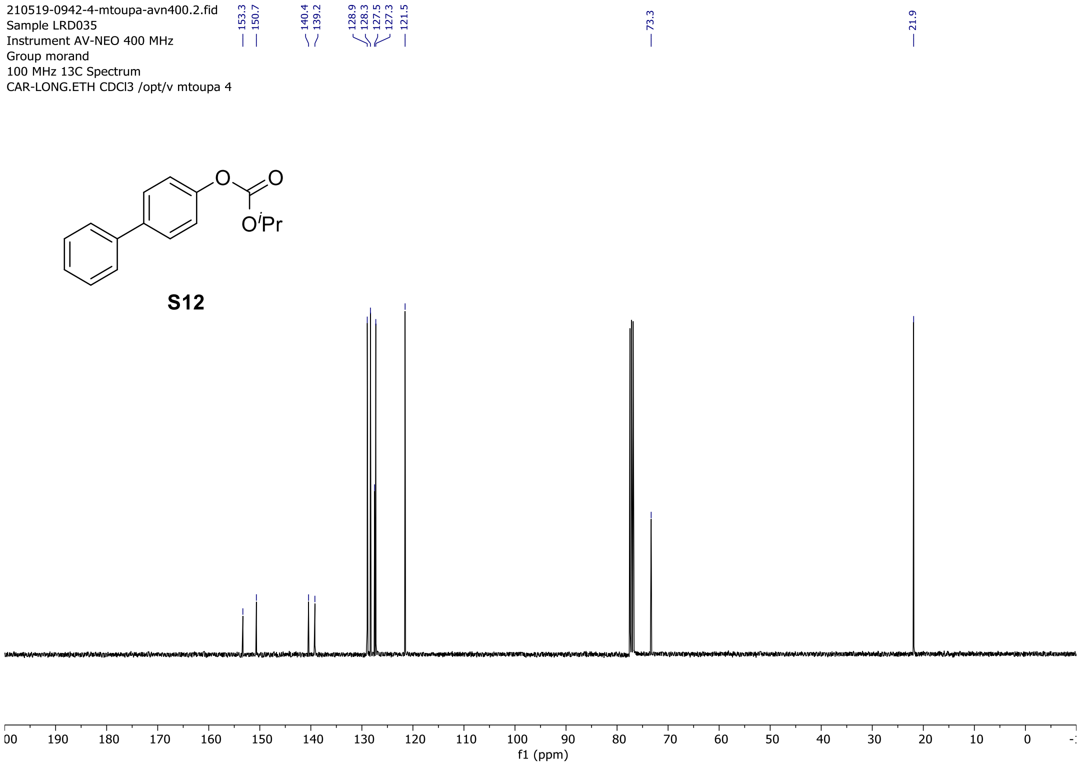

Supplementary Figure 37 ^{13}C NMR (100 MHz, $CDCl_3$, 25 °C) of compound **S12.**

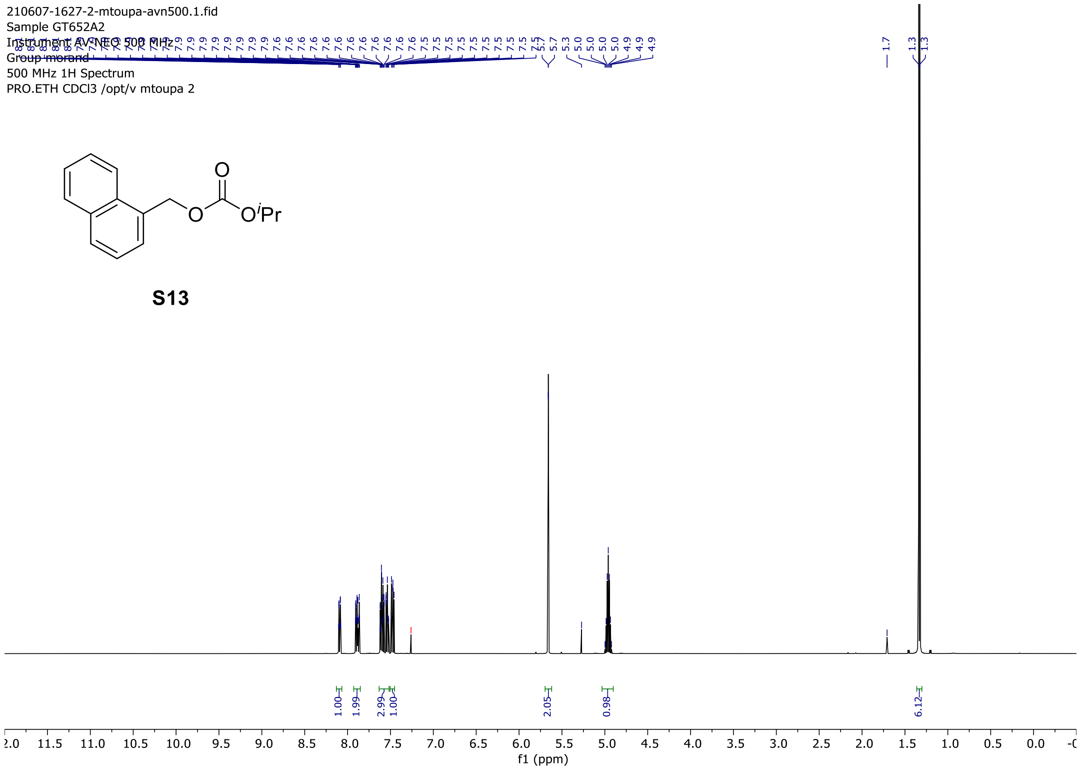

Supplementary Figure 38 ^{1}H NMR (500 MHz, $CDCl_3$, 25 °C) of compound **S13.**

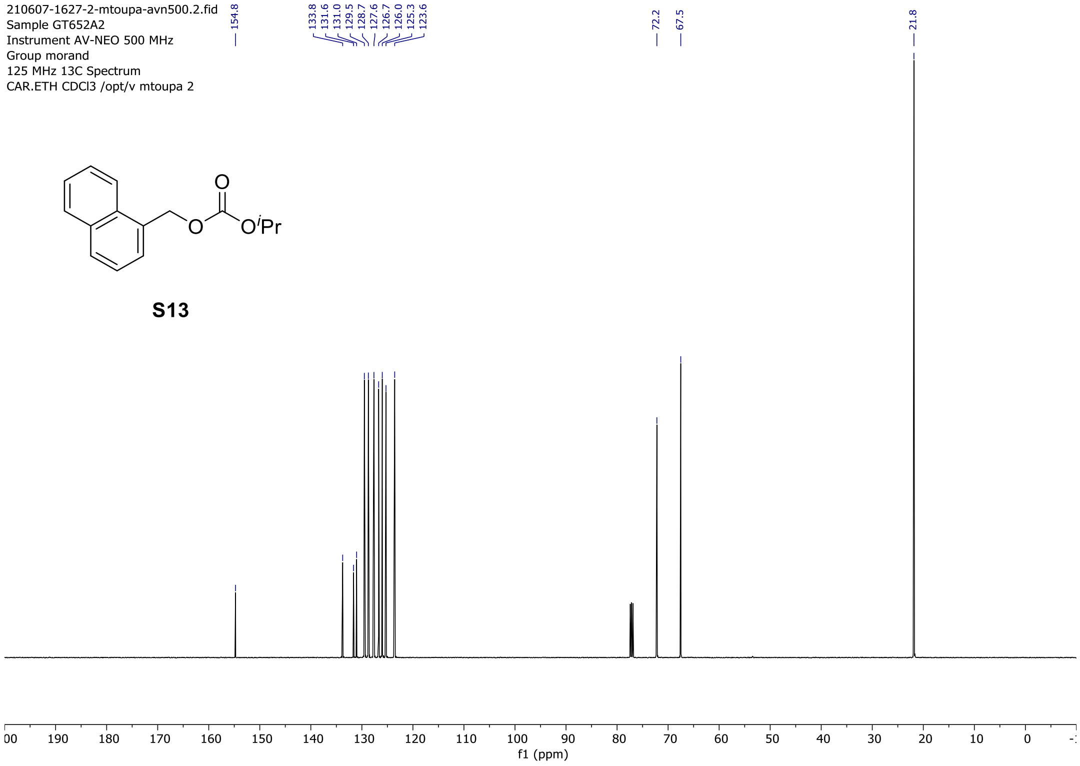

Supplementary Figure 39 ^{13}C NMR (125 MHz, $CDCl_3$, 25 °C) of compound **S13.**

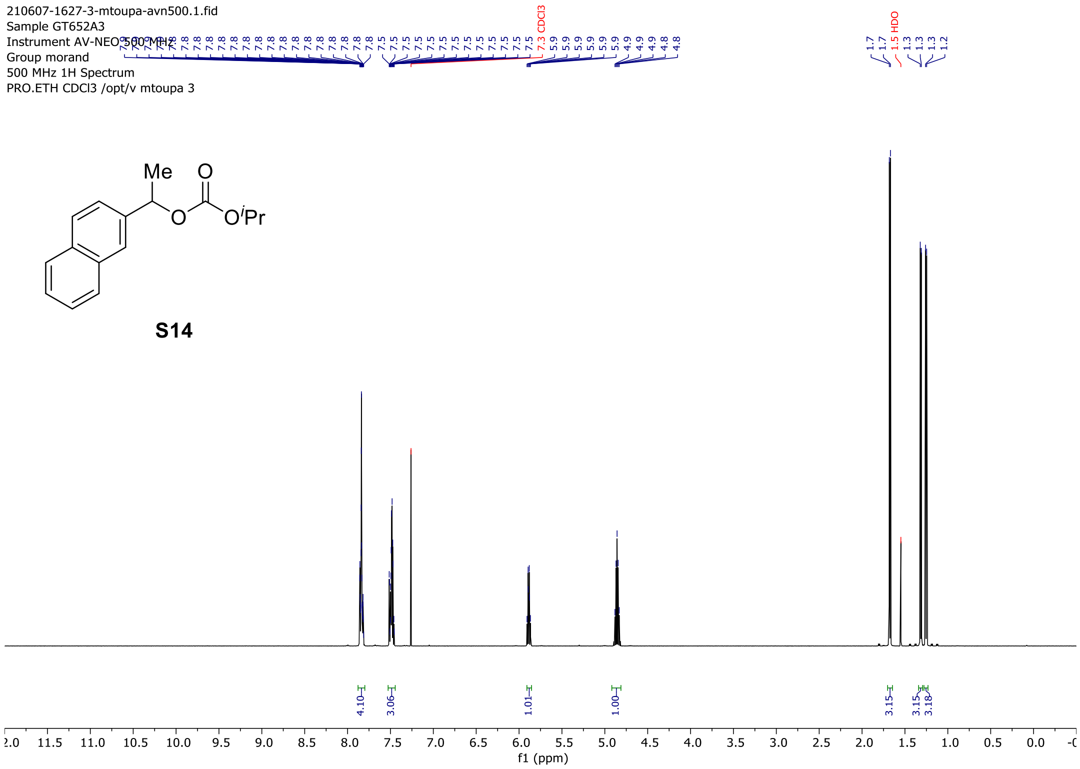

Supplementary Figure 40 ^{1}H NMR (500 MHz, $CDCl_3$, 25 °C) of compound **S14.**

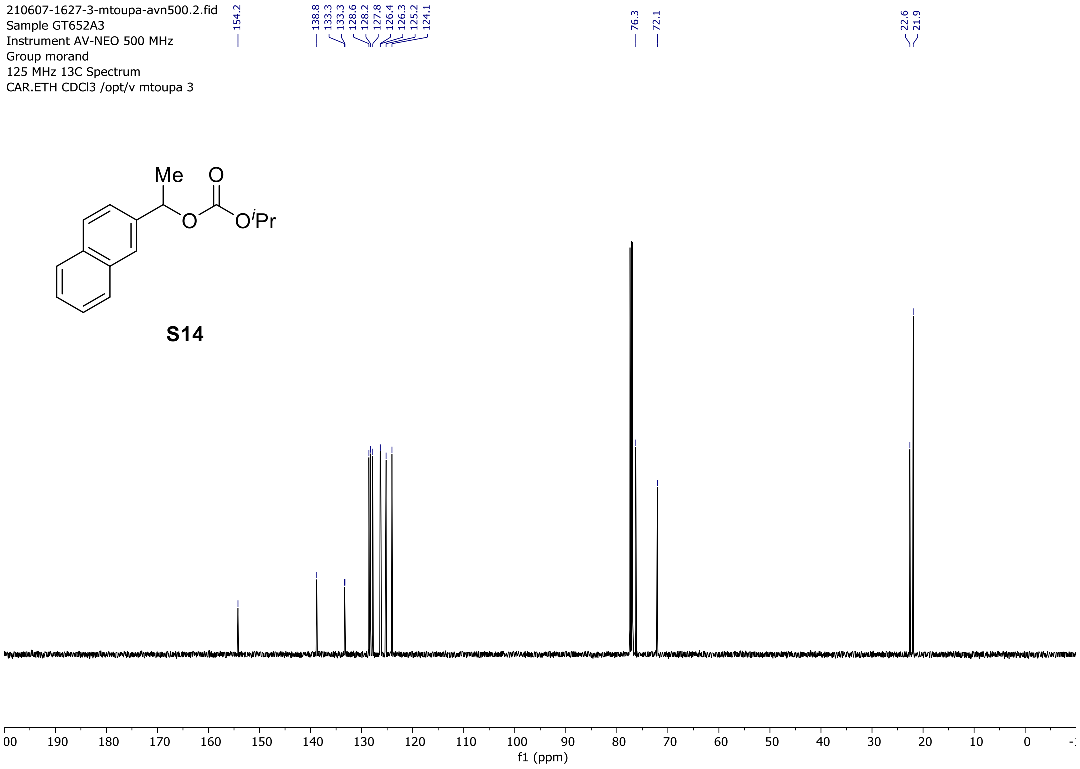

Supplementary Figure 41 ^{13}C NMR (125 MHz, $CDCl_3$, 25 °C) of compound **S14.**

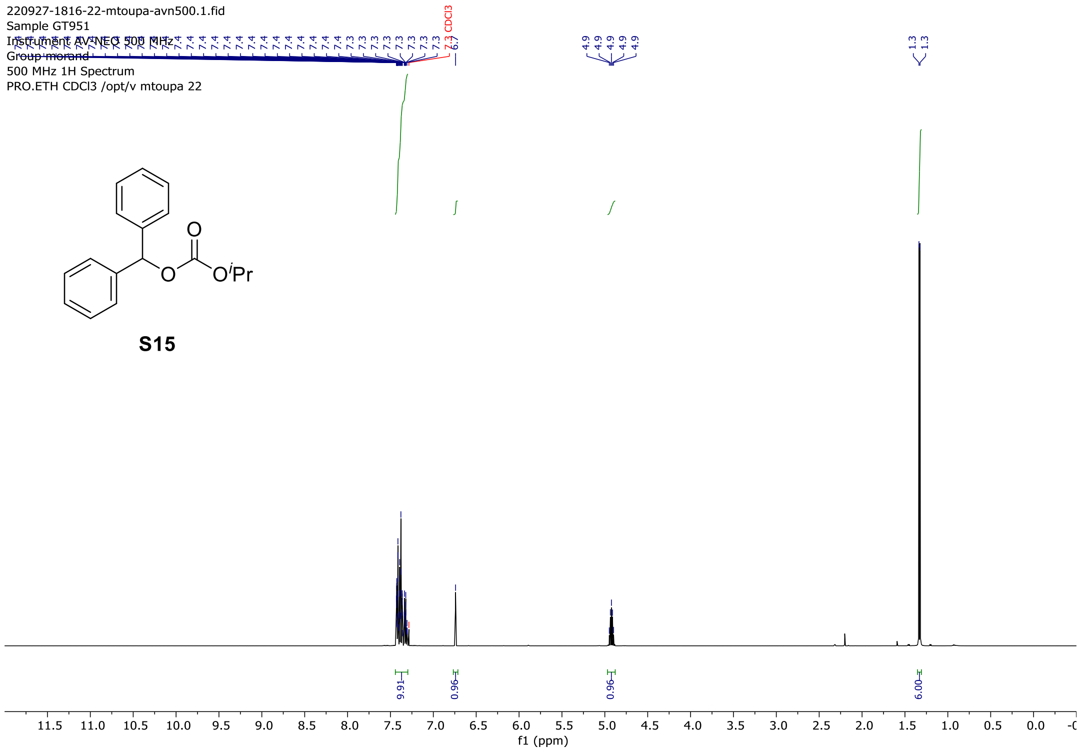

Supplementary Figure 42 ^{1}H NMR (500 MHz, $CDCl_3$, 25 °C) of compound **S15.**

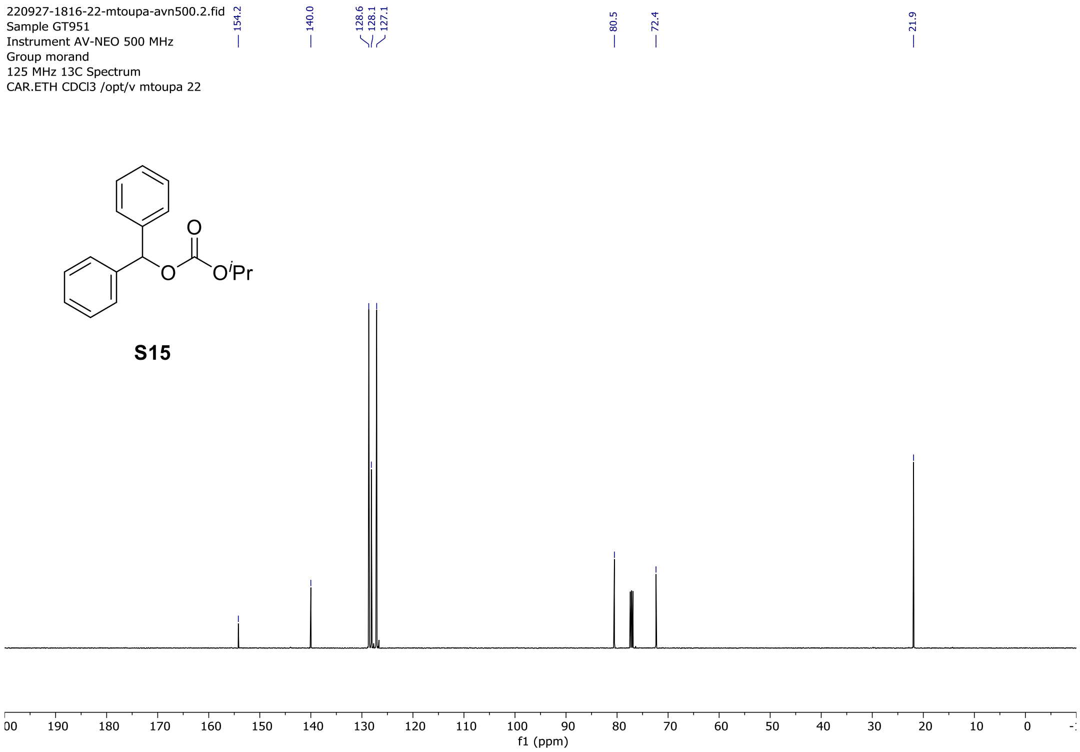

Supplementary Figure 43 ^{13}C NMR (125 MHz, $CDCl_3$, 25 °C) of compound **S15.**

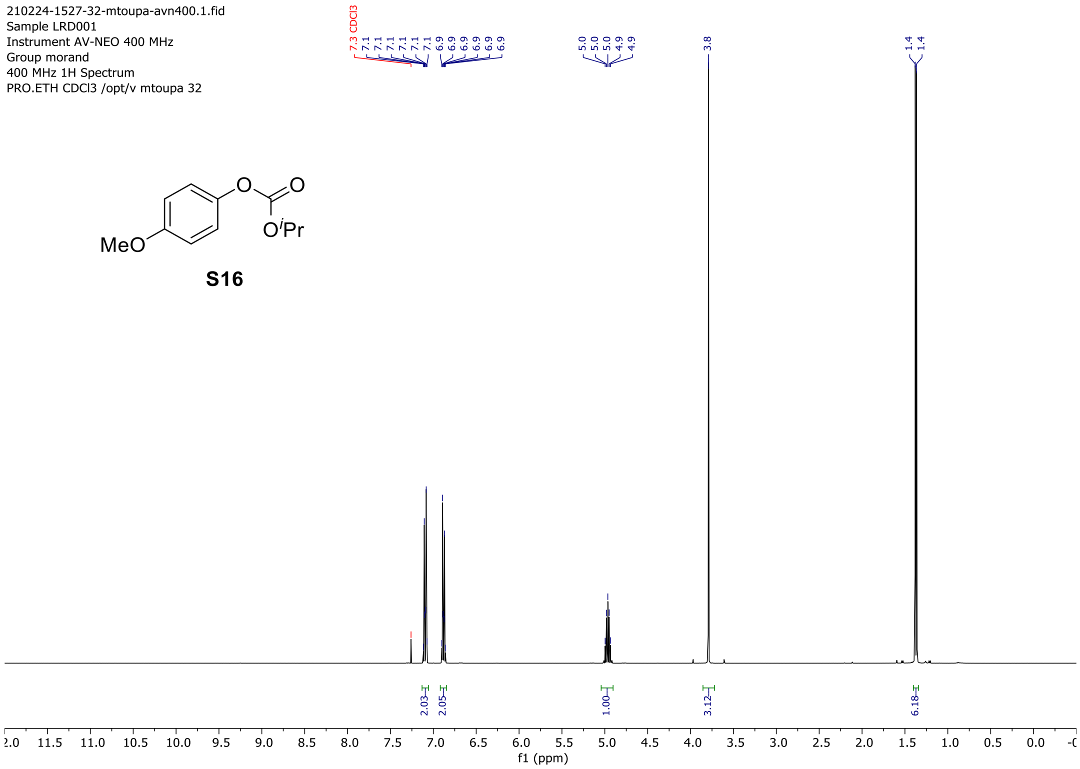

Supplementary Figure 44 ^{1}H NMR (400 MHz, $CDCl_3$, 25 °C) of compound **S16.**

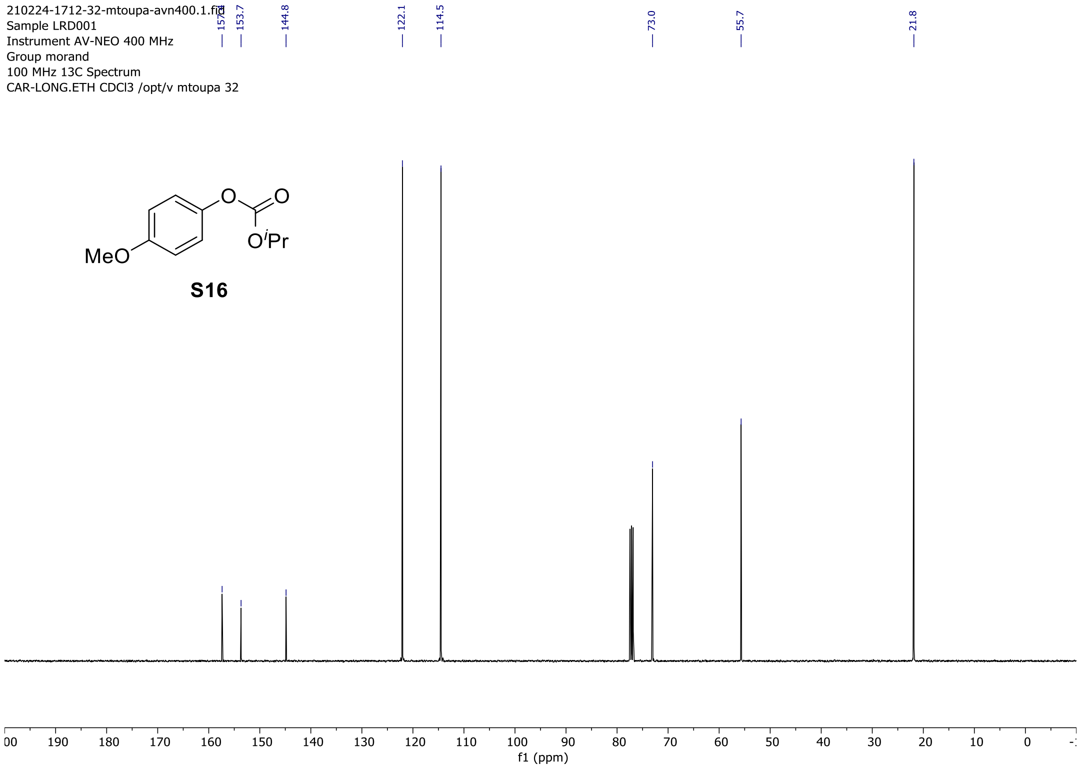

Supplementary Figure 45 ^{13}C NMR (100 MHz, $CDCl_3$, 25 °C) of compound **S16.**

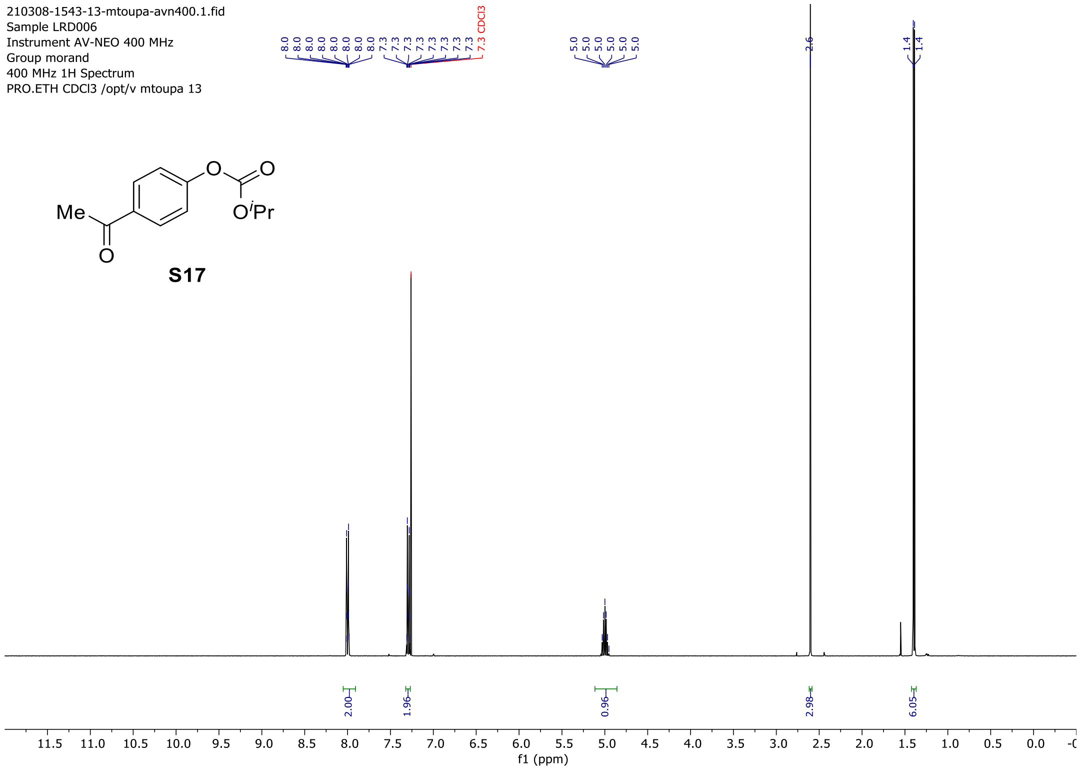

Supplementary Figure 46 ^{1}H NMR (400 MHz, $CDCl_3$, 25 °C) of compound **S17.**

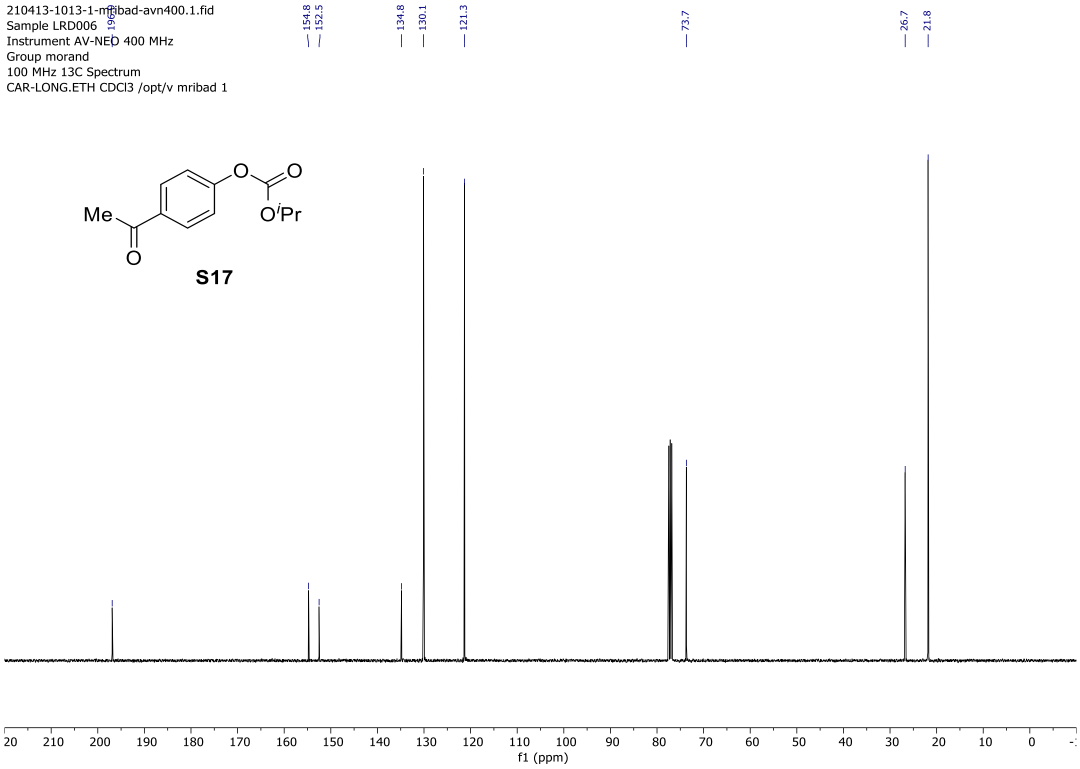

Supplementary Figure 47 ^{13}C NMR (100 MHz, $CDCl_3$, 25 °C) of compound **S17.**

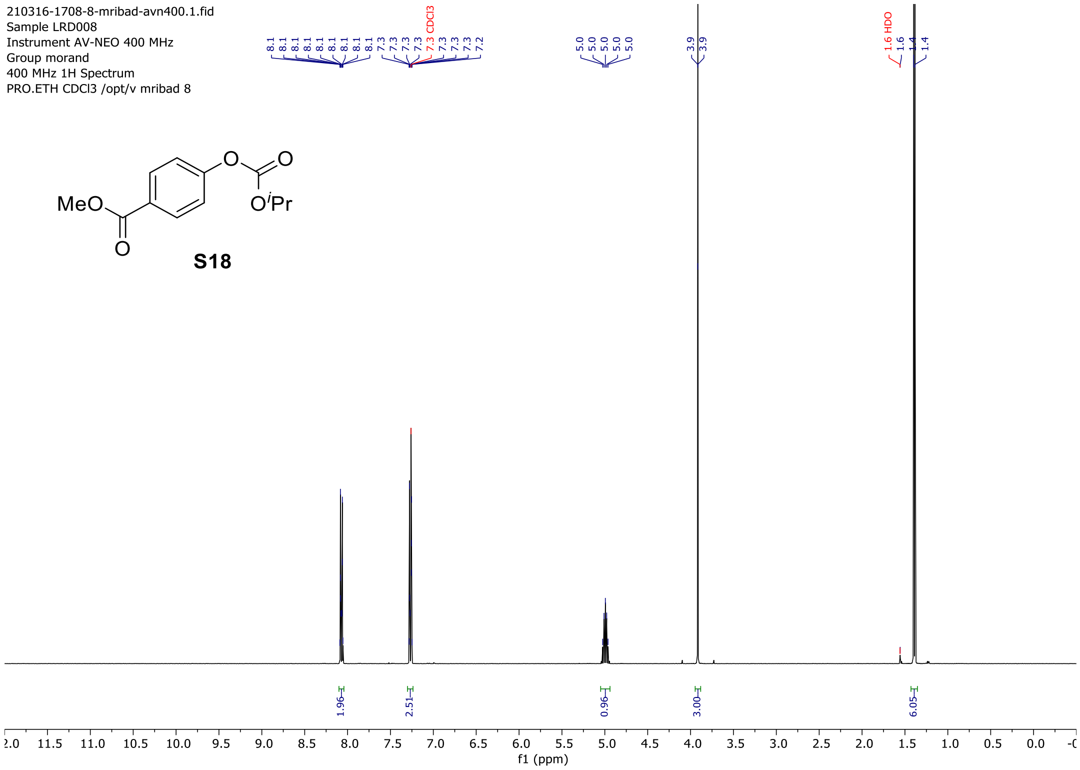

Supplementary Figure 48 ^{1}H NMR (400 MHz, $CDCl_3$, 25 °C) of compound **S18.**

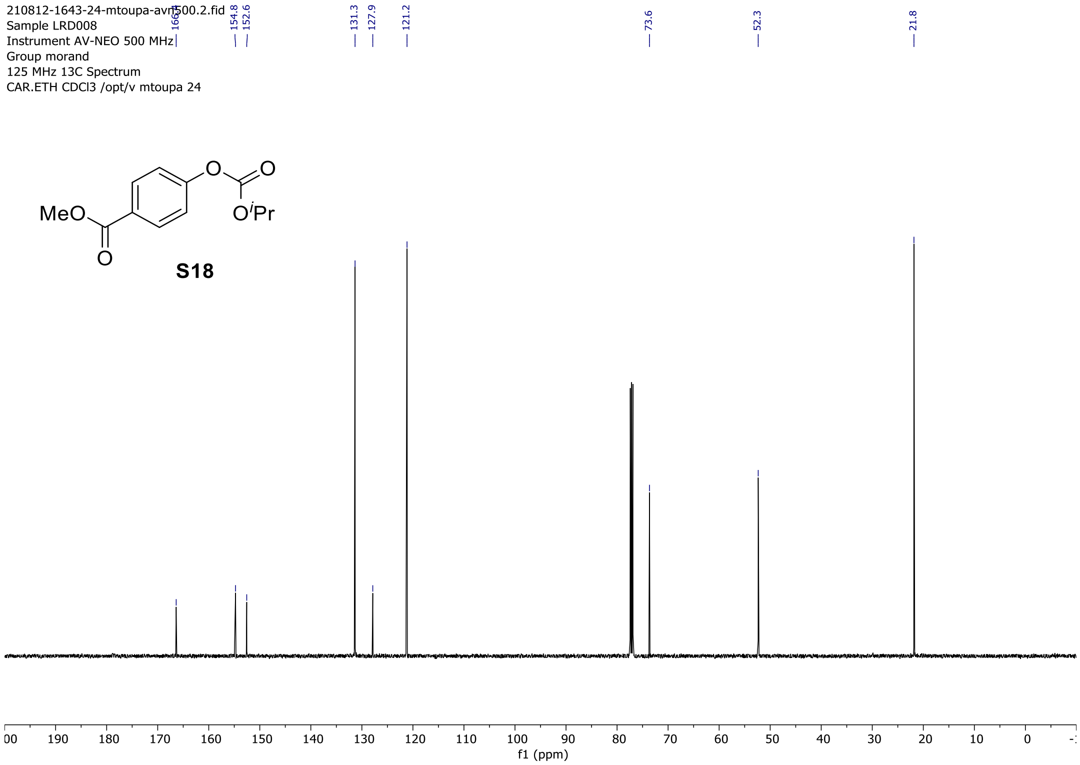

Supplementary Figure 49 ^{13}C NMR (125 MHz, $CDCl_3$, 25 °C) of compound **S18.**

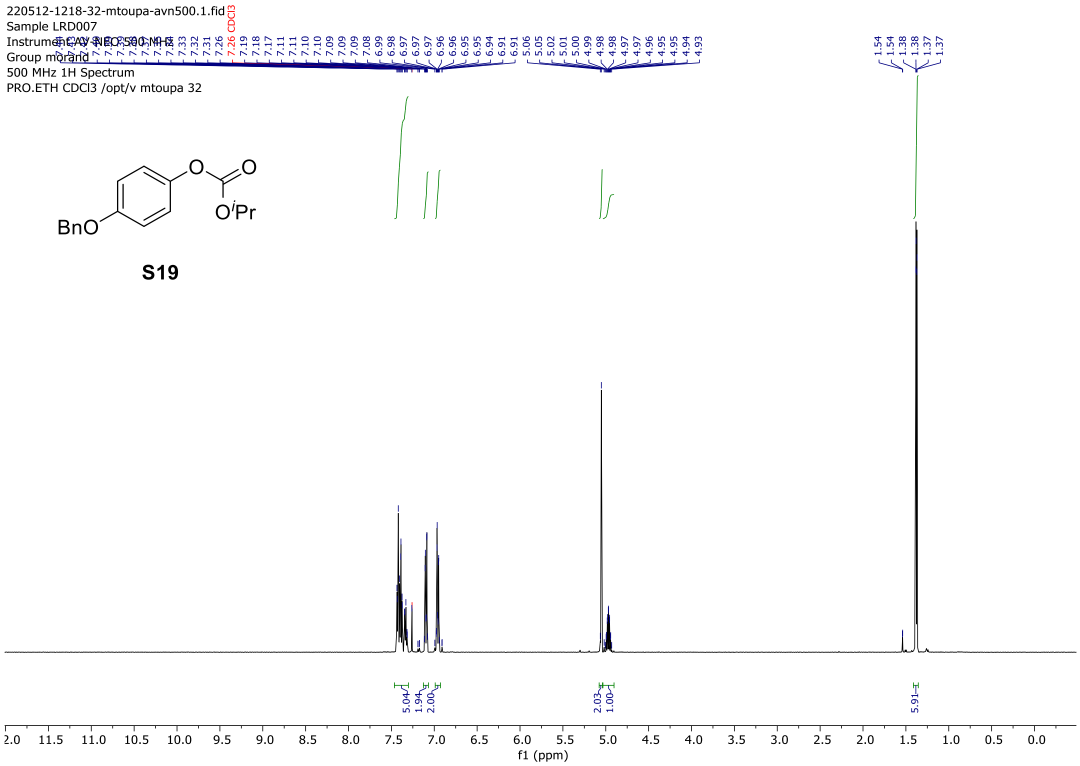

Supplementary Figure 50 ^{1}H NMR (500 MHz, $CDCl_3$, 25 °C) of compound **S19.**

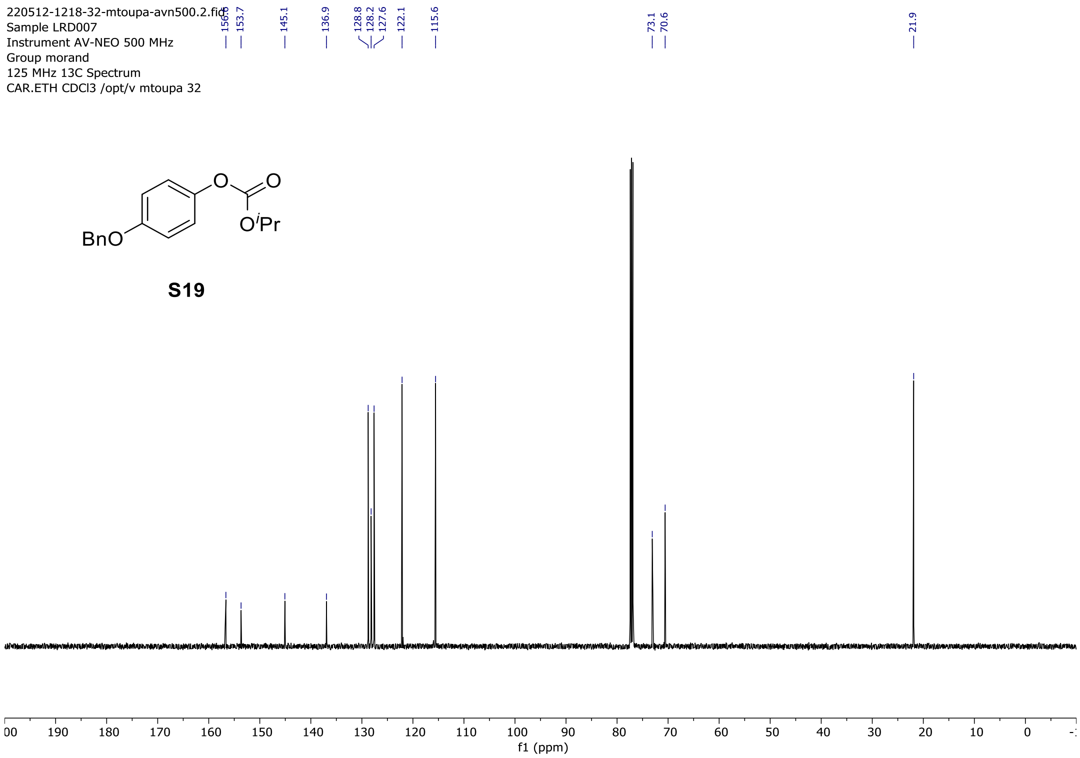

Supplementary Figure 51 ^{13}C NMR (125 MHz, $CDCl_3$, 25 °C) of compound **S19.**

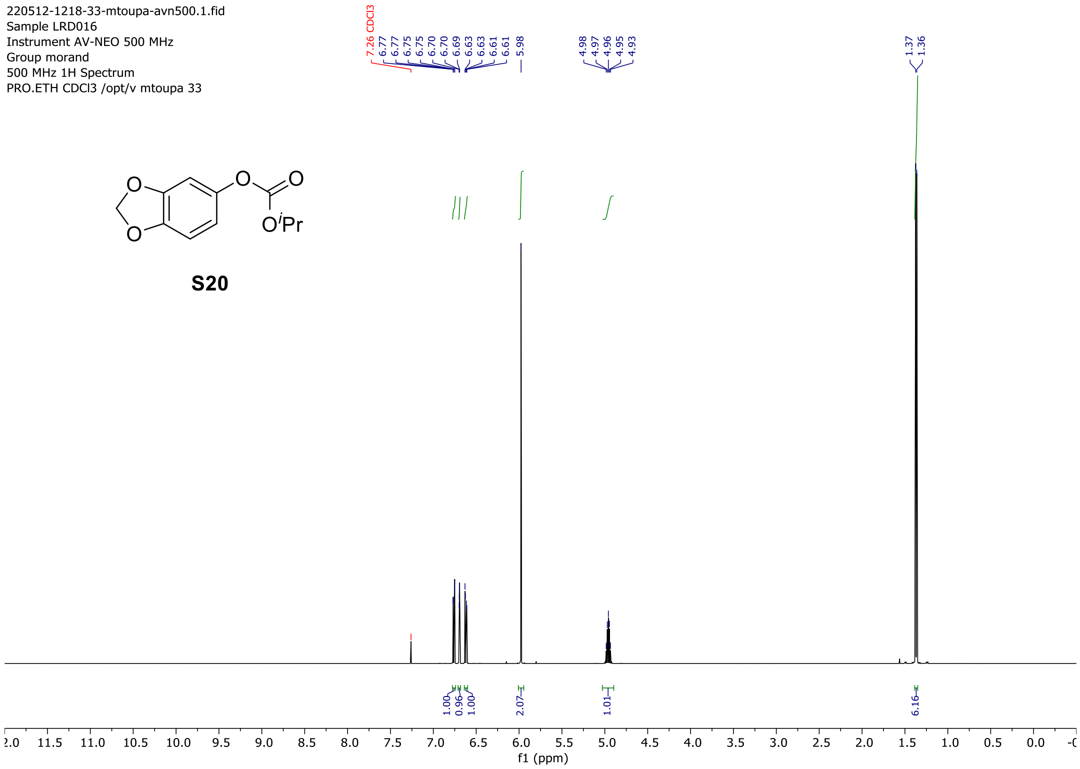

Supplementary Figure 52 ^{1}H NMR (500 MHz, $CDCl_3$, 25 °C) of compound **S20.**

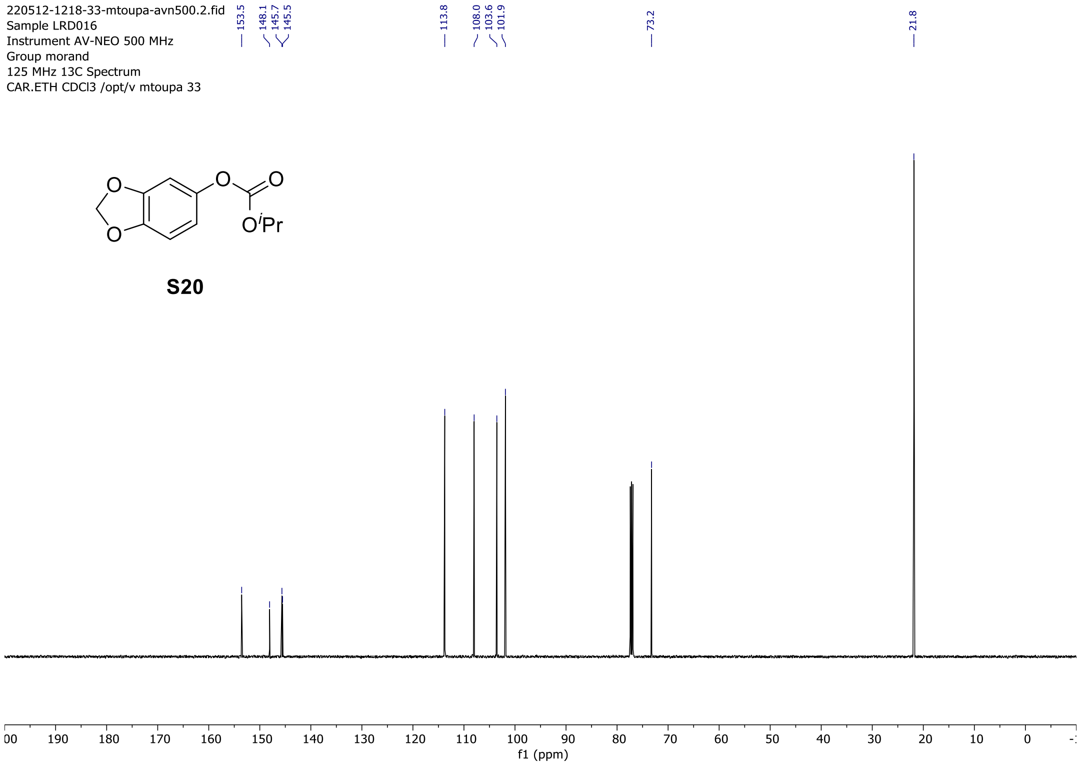

Supplementary Figure 53 ^{13}C NMR (125 MHz, $CDCl_3$, 25 °C) of compound **S20.**

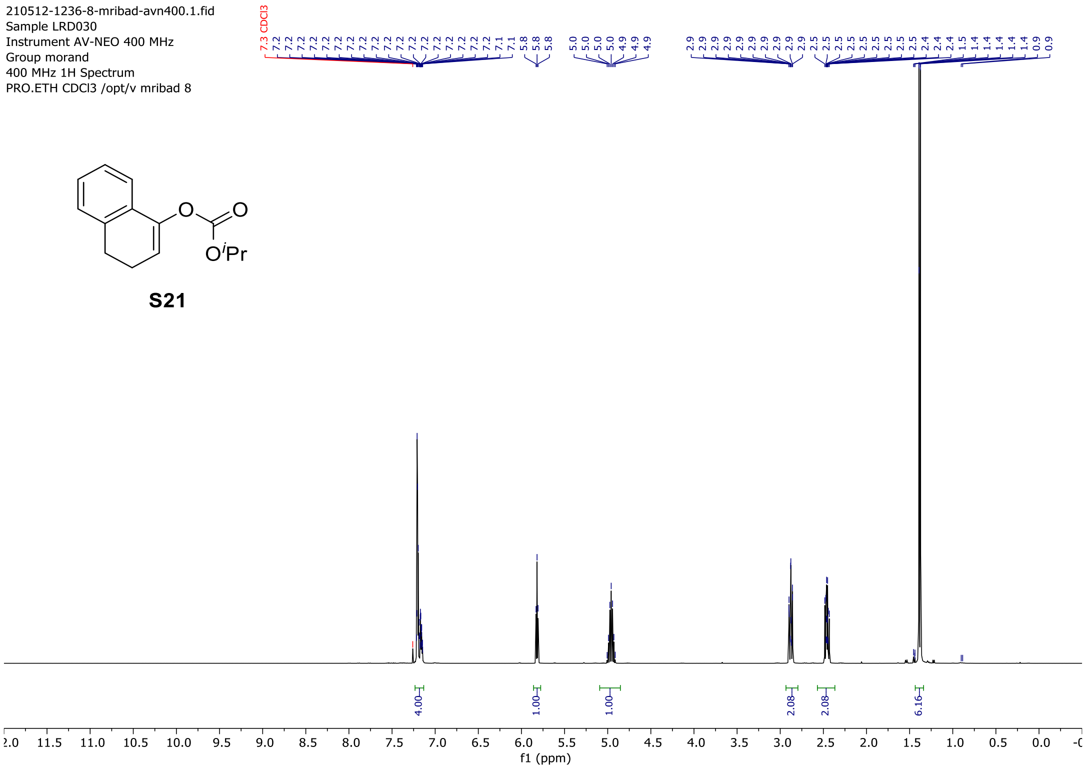

Supplementary Figure 54 ^{1}H NMR (400 MHz, $CDCl_3$, 25 °C) of compound **S21.**

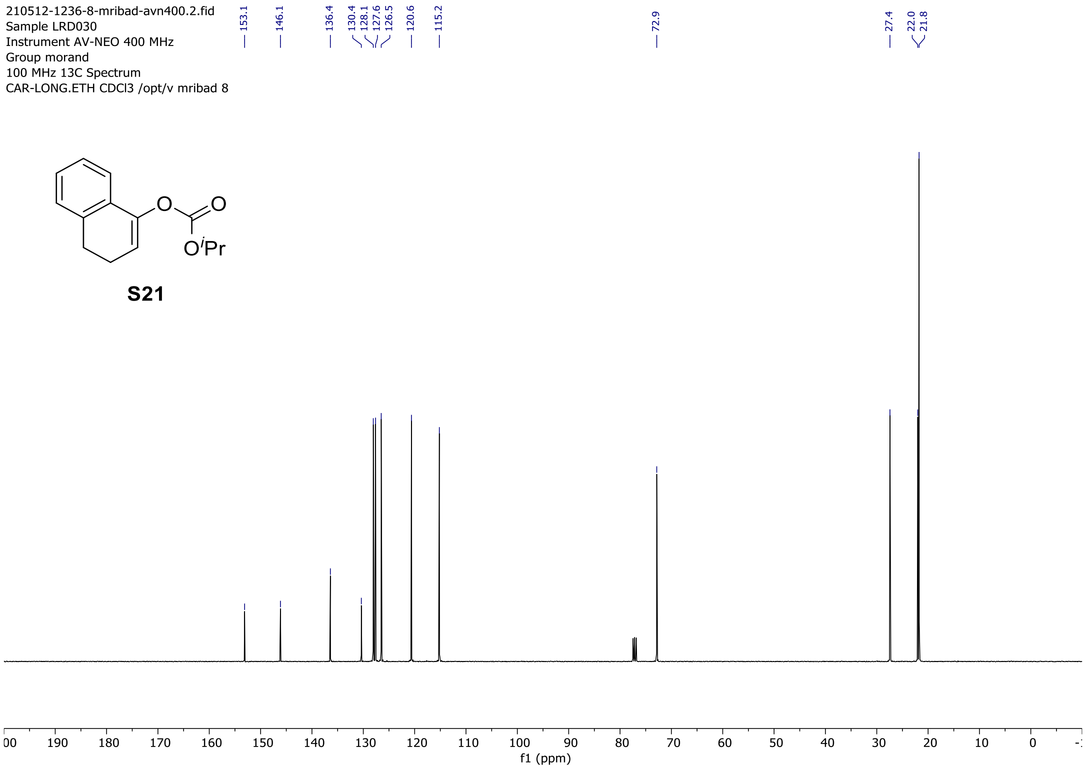

Supplementary Figure 55 ^{13}C NMR (100 MHz, $CDCl_3$, 25 °C) of compound **S21.**

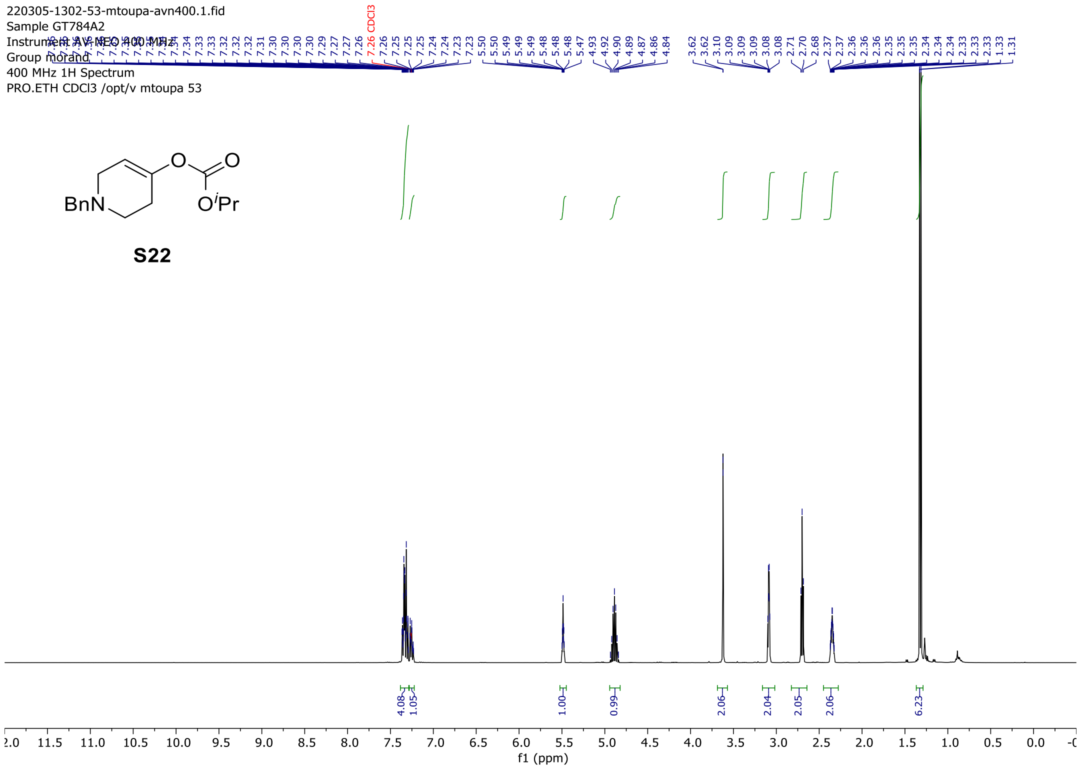

Supplementary Figure 56 ^{1}H NMR (400 MHz, $CDCl_3$, 25 °C) of compound **S22.**

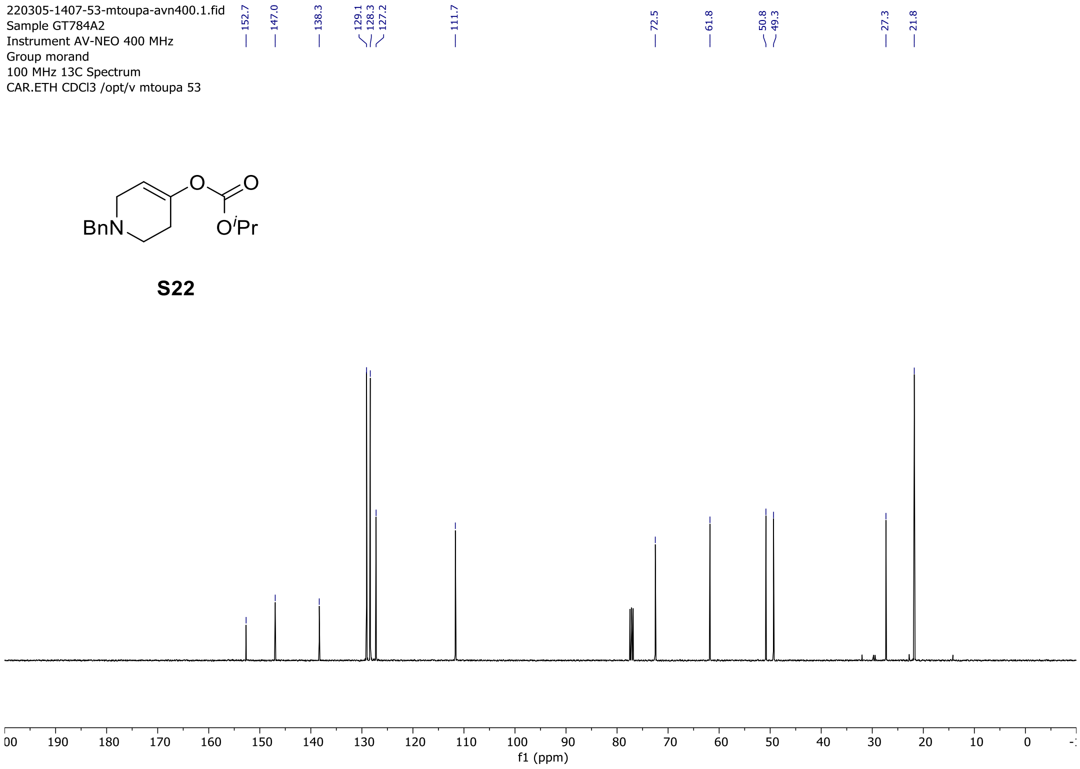

Supplementary Figure 57 ^{13}C NMR (100 MHz, $CDCl_3$, 25 °C) of compound **S22.**

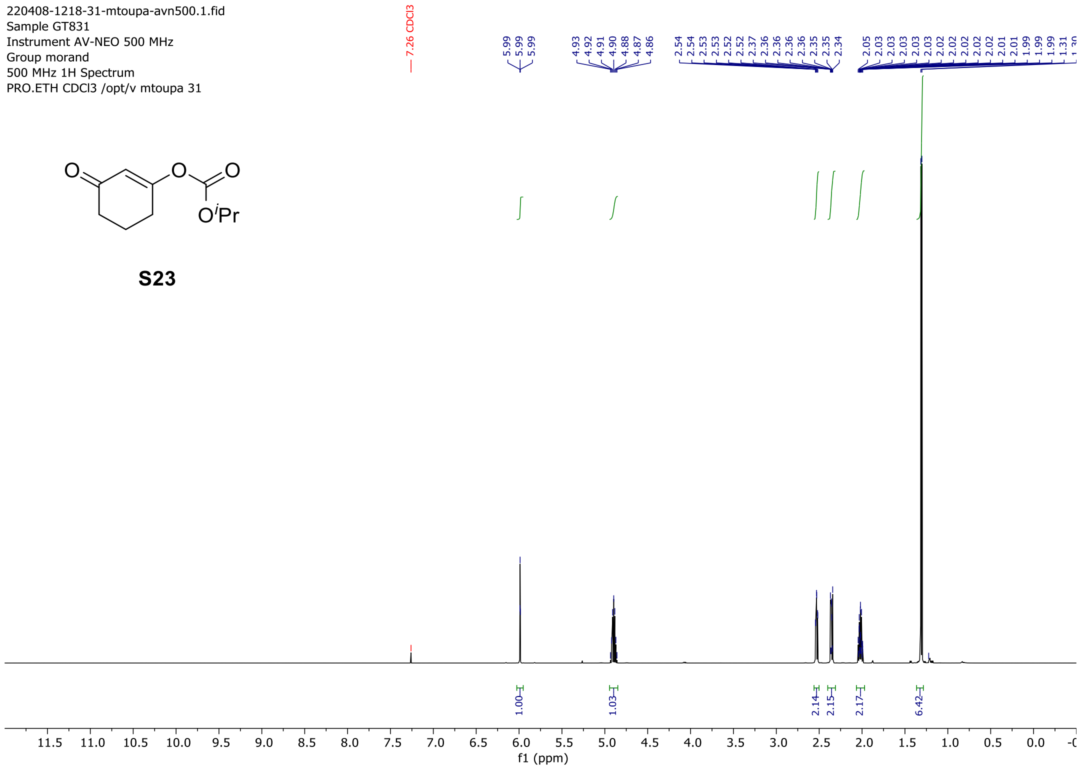

Supplementary Figure 58 ^{1}H NMR (500 MHz, $CDCl_3$, 25 °C) of compound **S23.**

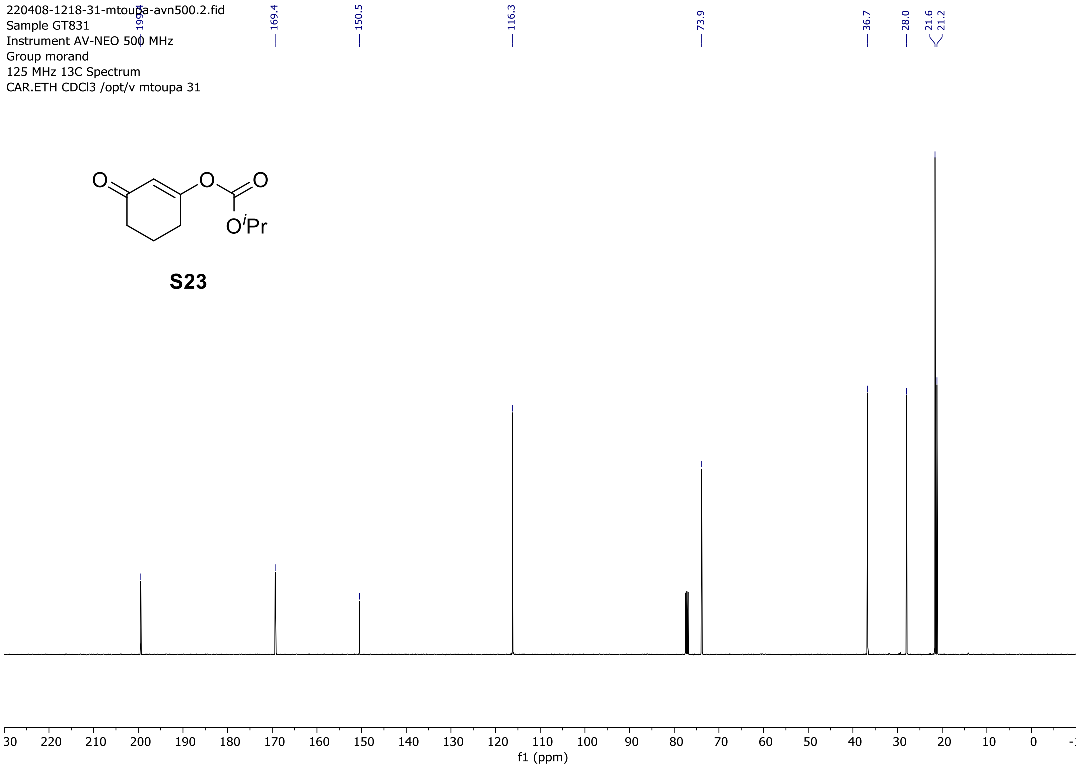

Supplementary Figure 59 ^{13}C NMR (125 MHz, $CDCl_3$, 25 °C) of compound **S23.**

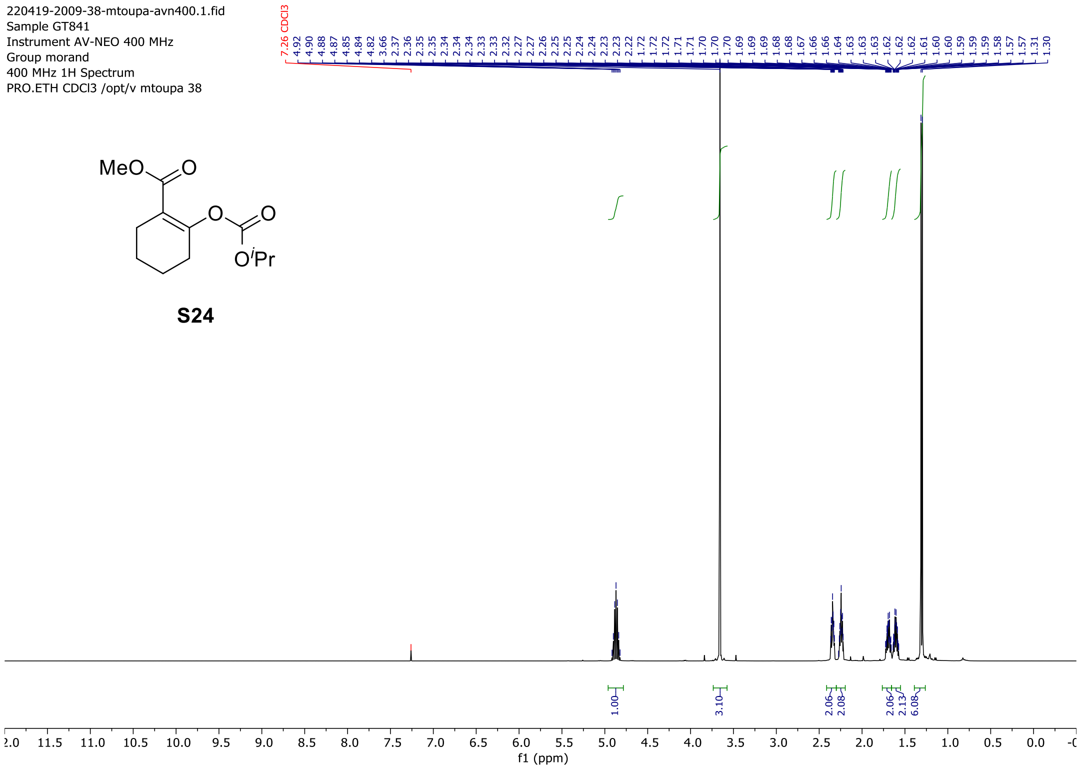

Supplementary Figure 60 ^{1}H NMR (400 MHz, $CDCl_3$, 25 °C) of compound **S24.**

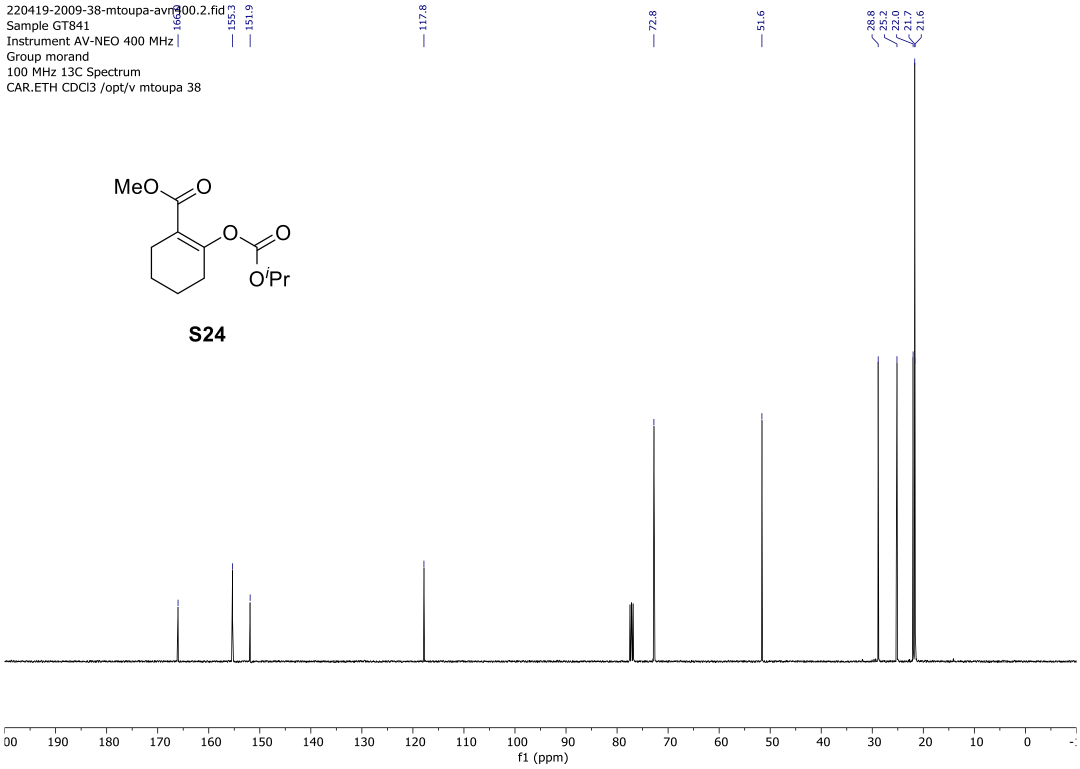

Supplementary Figure 61 ^{13}C NMR (100 MHz, $CDCl_3$, 25 °C) of compound **S24.**

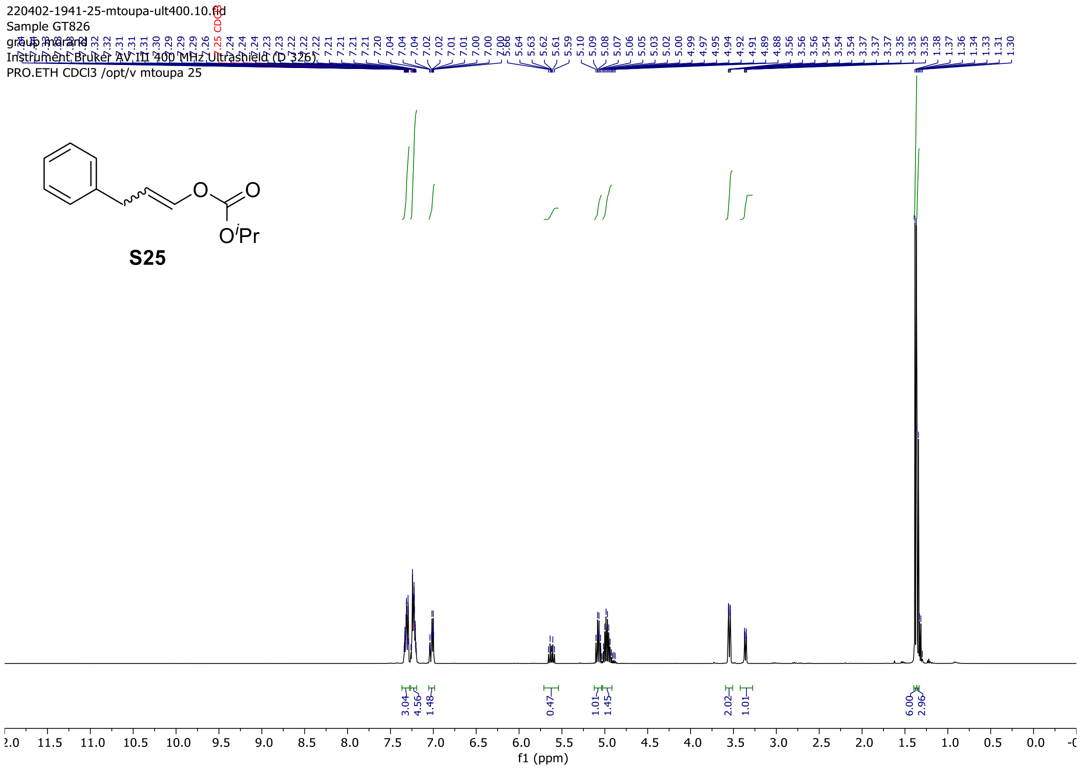

Supplementary Figure 62 ^{1}H NMR (400 MHz, $CDCl_3$, 25 °C) of compound **S25.**

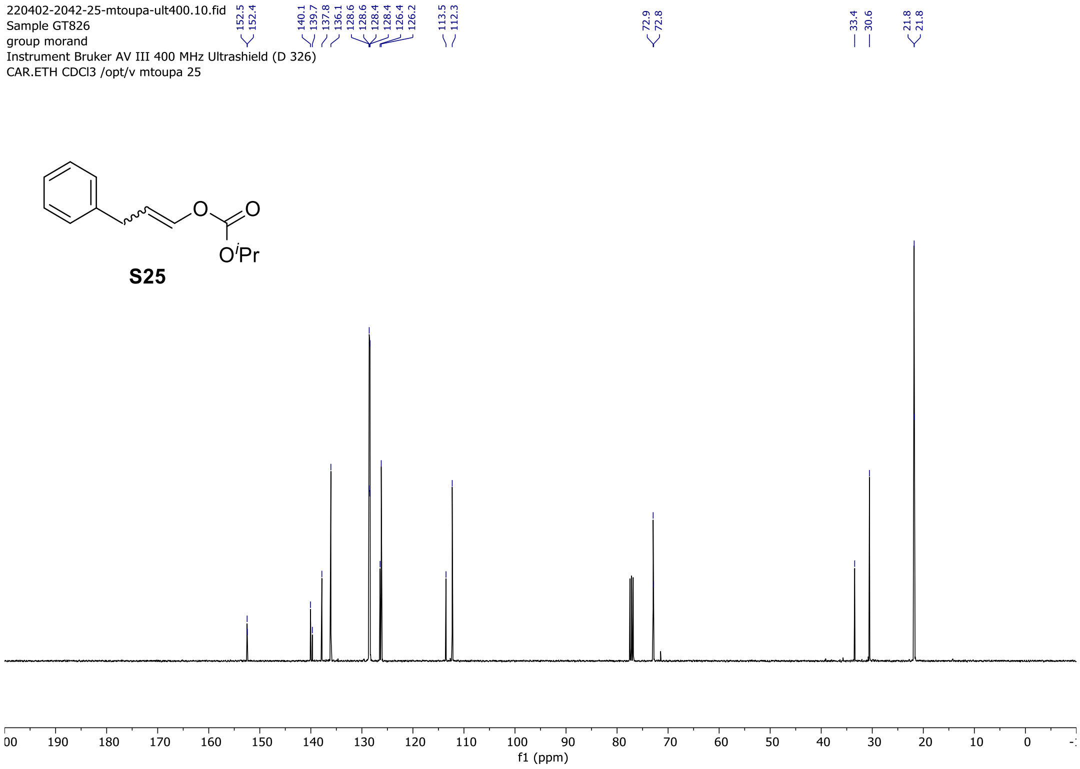

Supplementary Figure 63 ^{13}C NMR (100 MHz, $CDCl_3$, 25 °C) of compound **S25.**

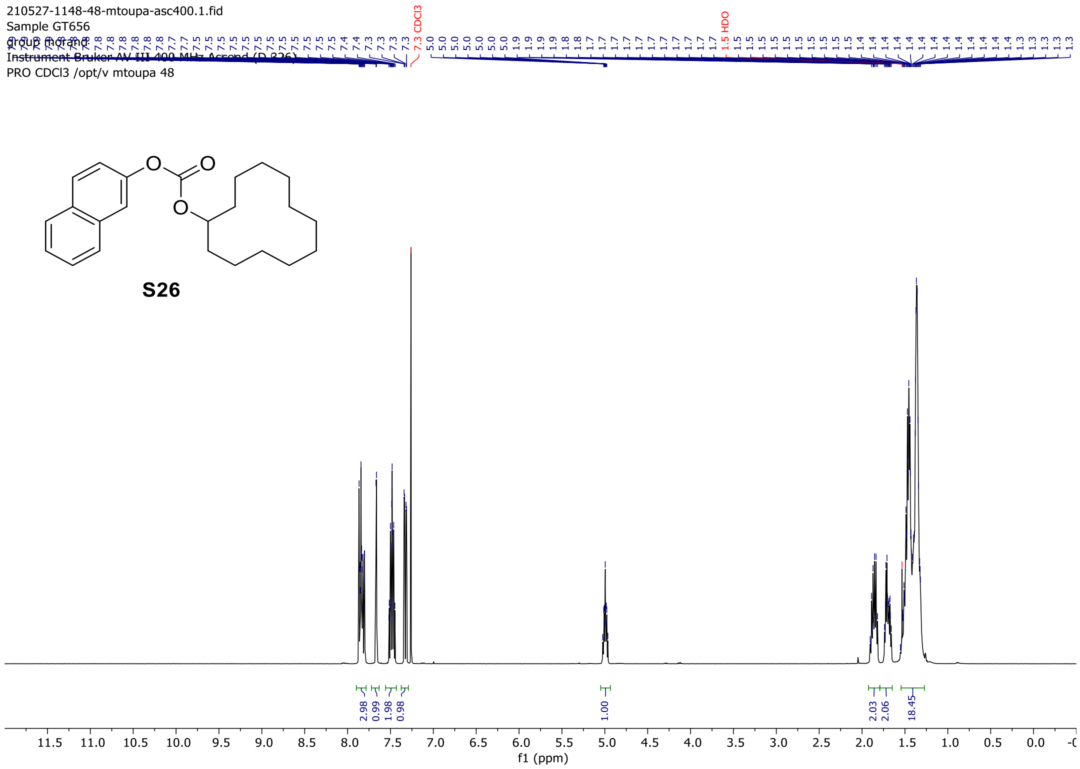

Supplementary Figure 64 ^{1}H NMR (400 MHz, $CDCl_3$, 25 °C) of compound **S26.**

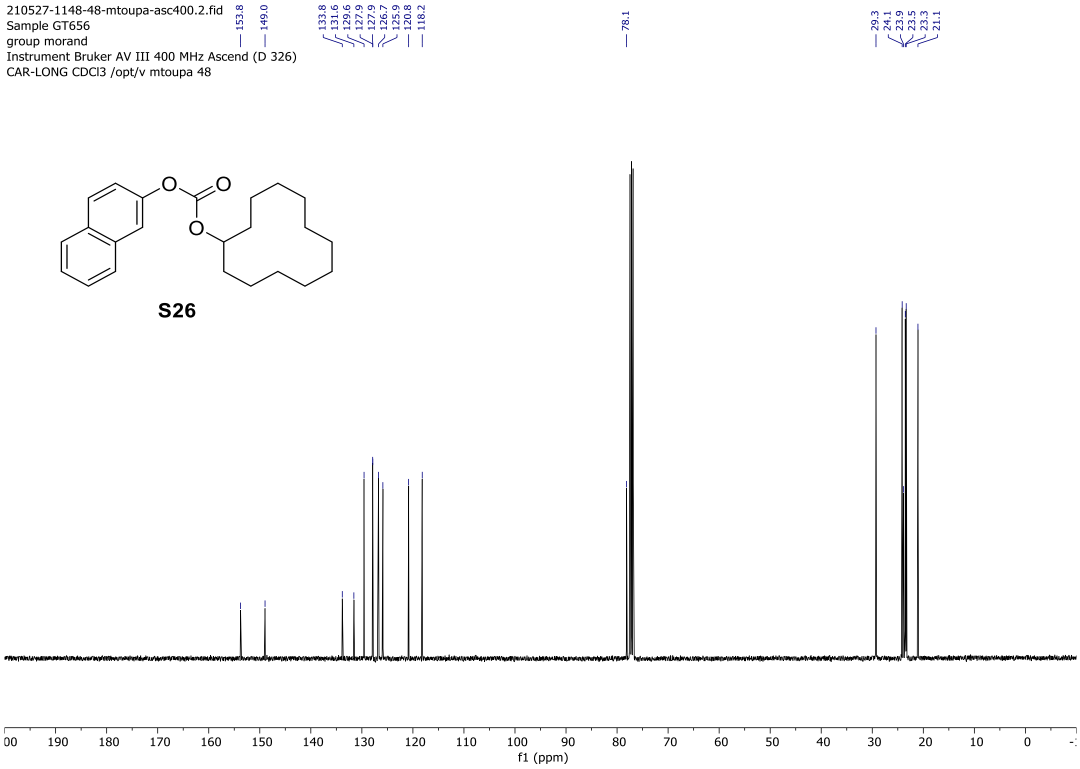

Supplementary Figure 65 ^{13}C NMR (100 MHz, $CDCl_3$, 25 °C) of compound **S26.**

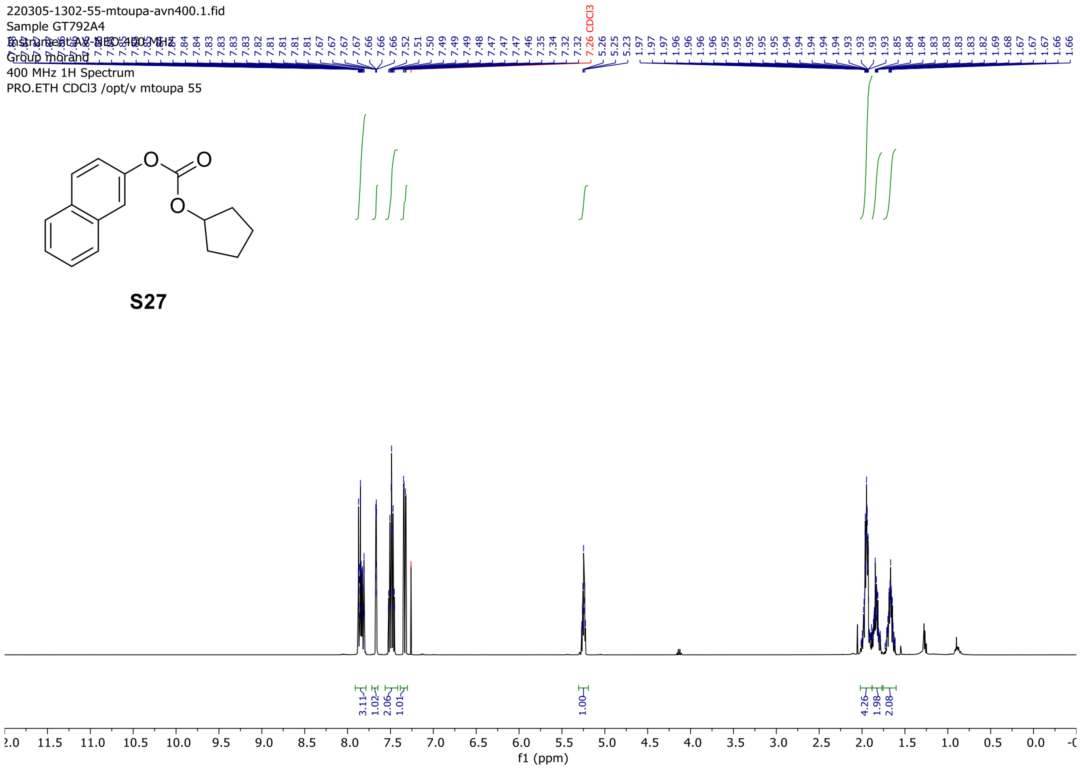

Supplementary Figure 66 ^{1}H NMR (400 MHz, $CDCl_3$, 25 °C) of compound **S27.**

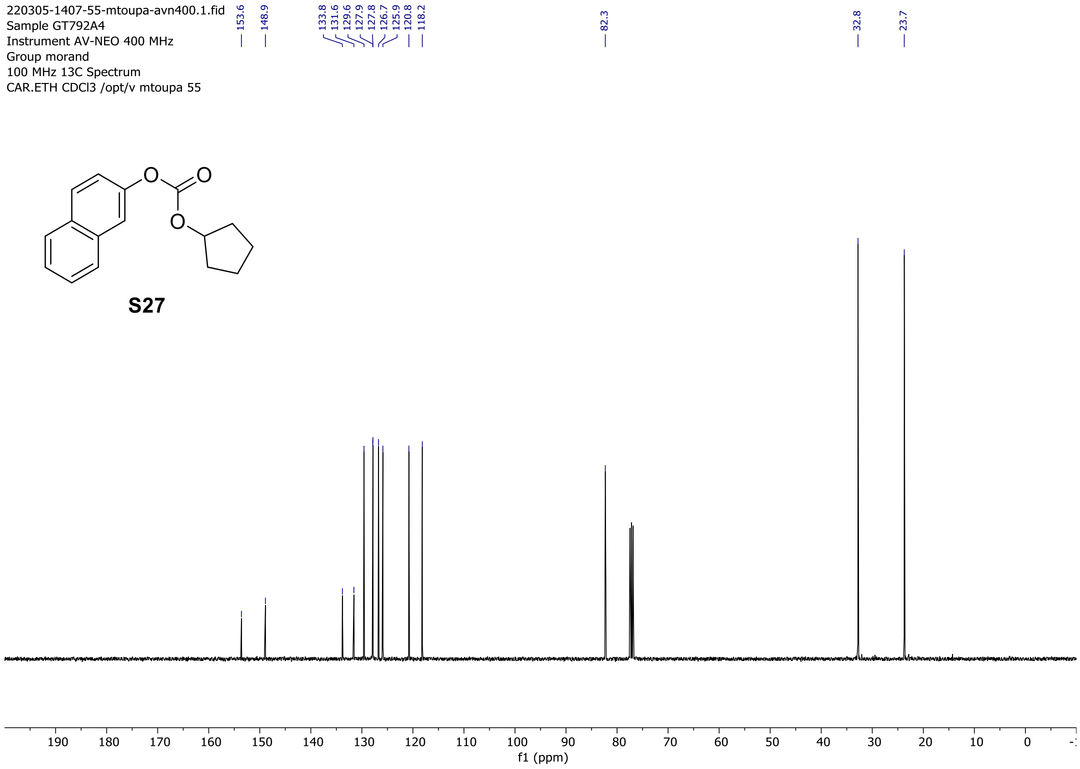

Supplementary Figure 67 ^{13}C NMR (100 MHz, $CDCl_3$, 25 °C) of compound **S27.**

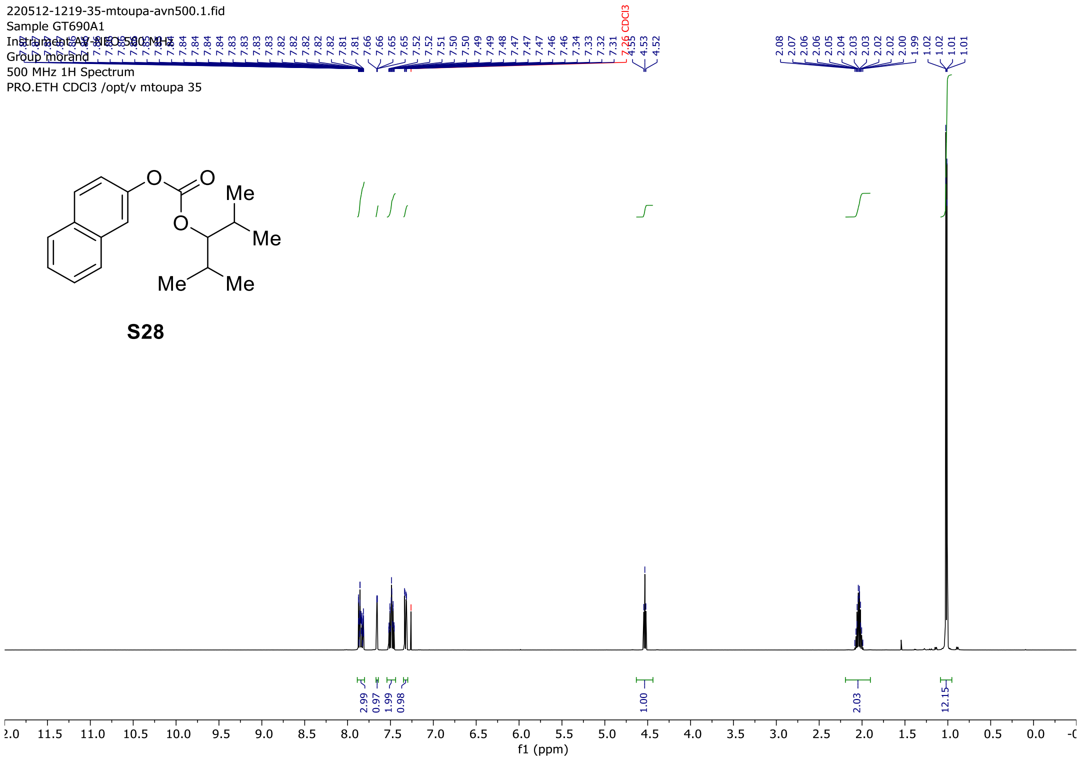

Supplementary Figure 68 ^{1}H NMR (500 MHz, $CDCl_3$, 25 °C) of compound **S28.**

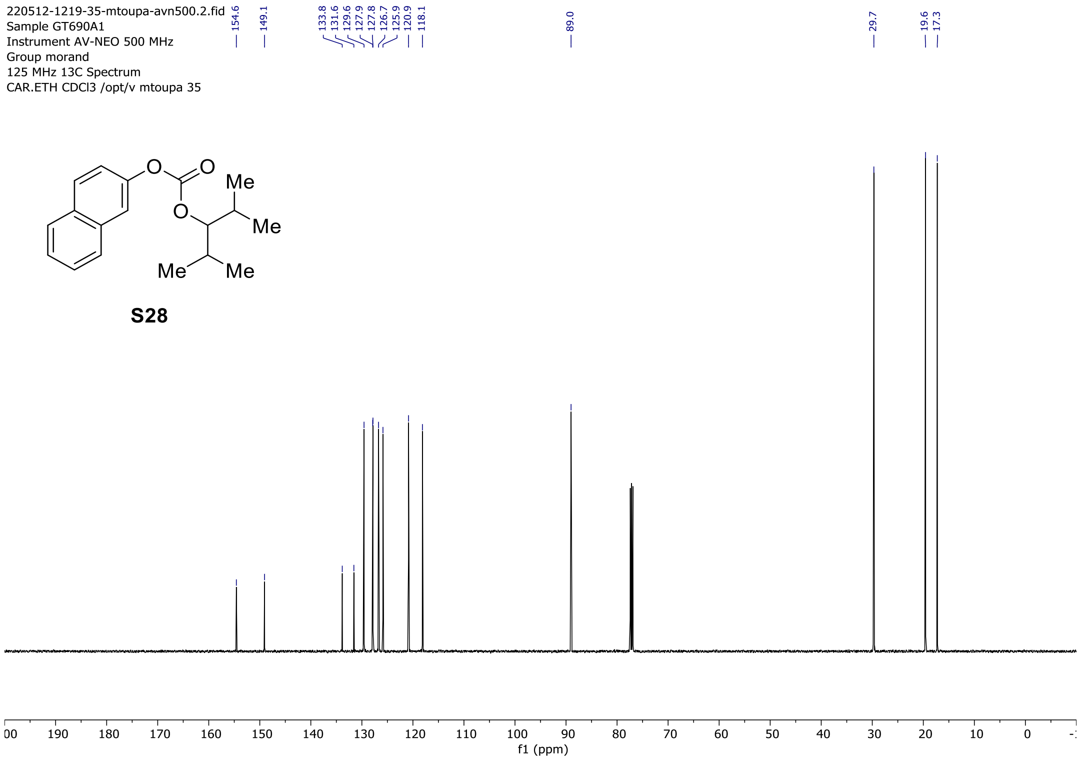

Supplementary Figure 69 ^{13}C NMR (125 MHz, $CDCl_3$, 25 °C) of compound **S28.**

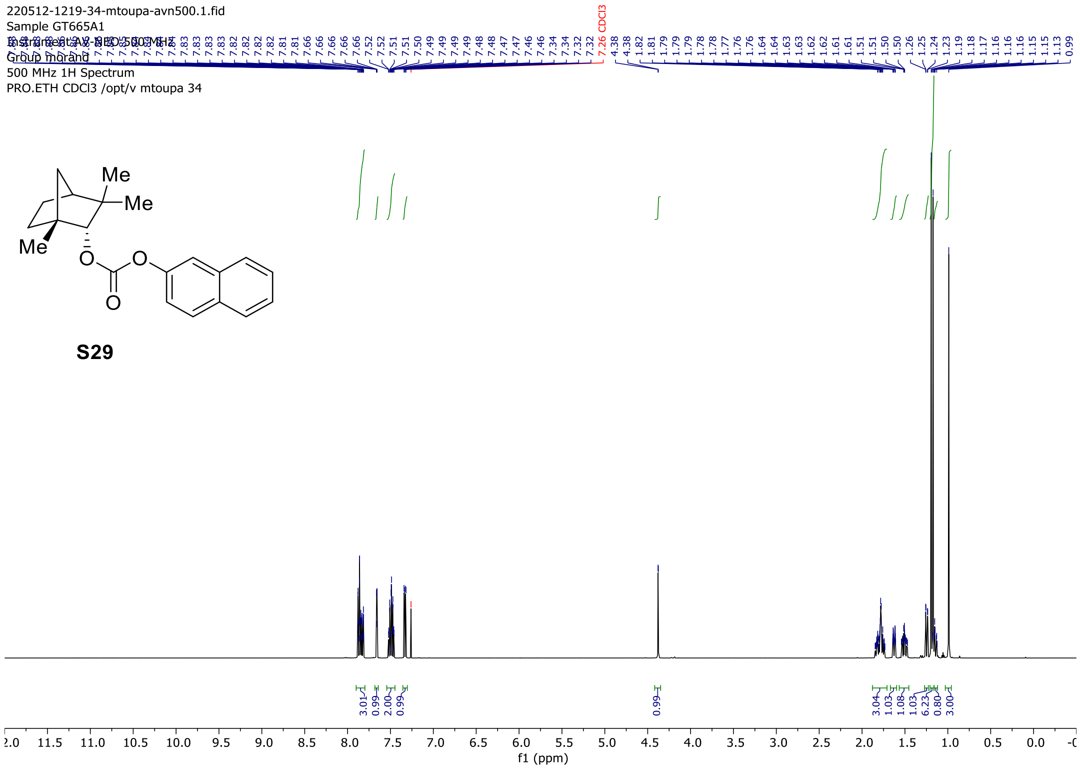

Supplementary Figure 70 ^{1}H NMR (500 MHz, $CDCl_3$, 25 °C) of compound **S29.**

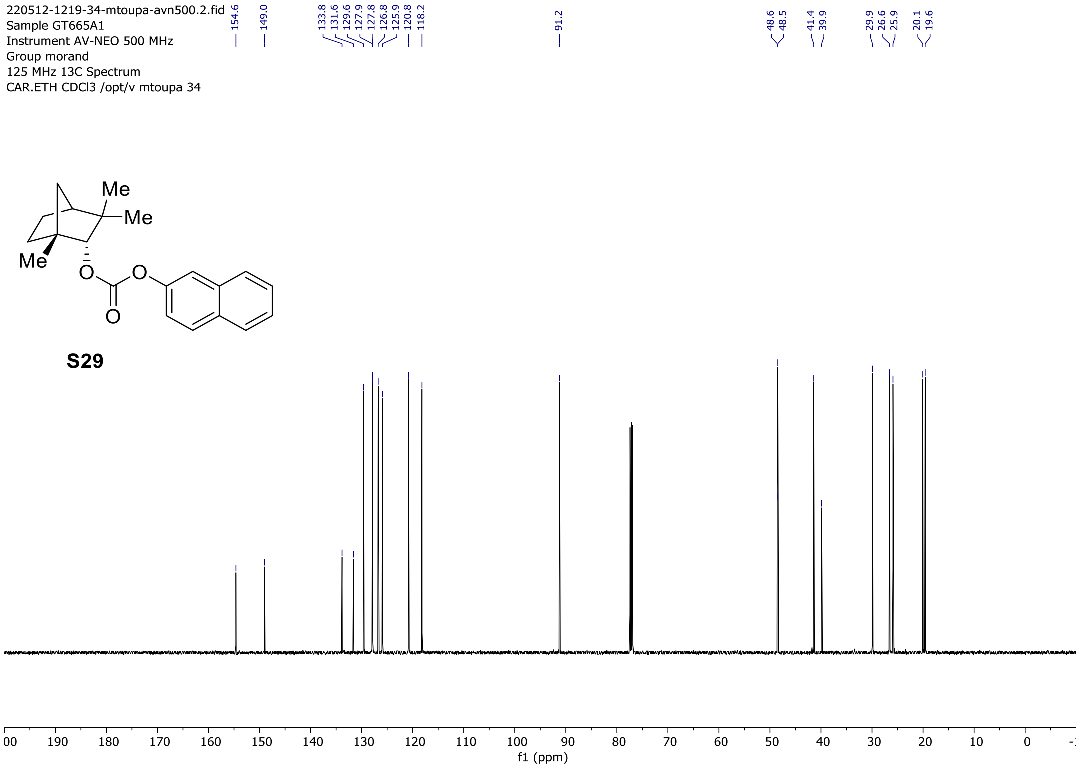

Supplementary Figure 71 ^{13}C NMR (125 MHz, $CDCl_3$, 25 °C) of compound **S29.**

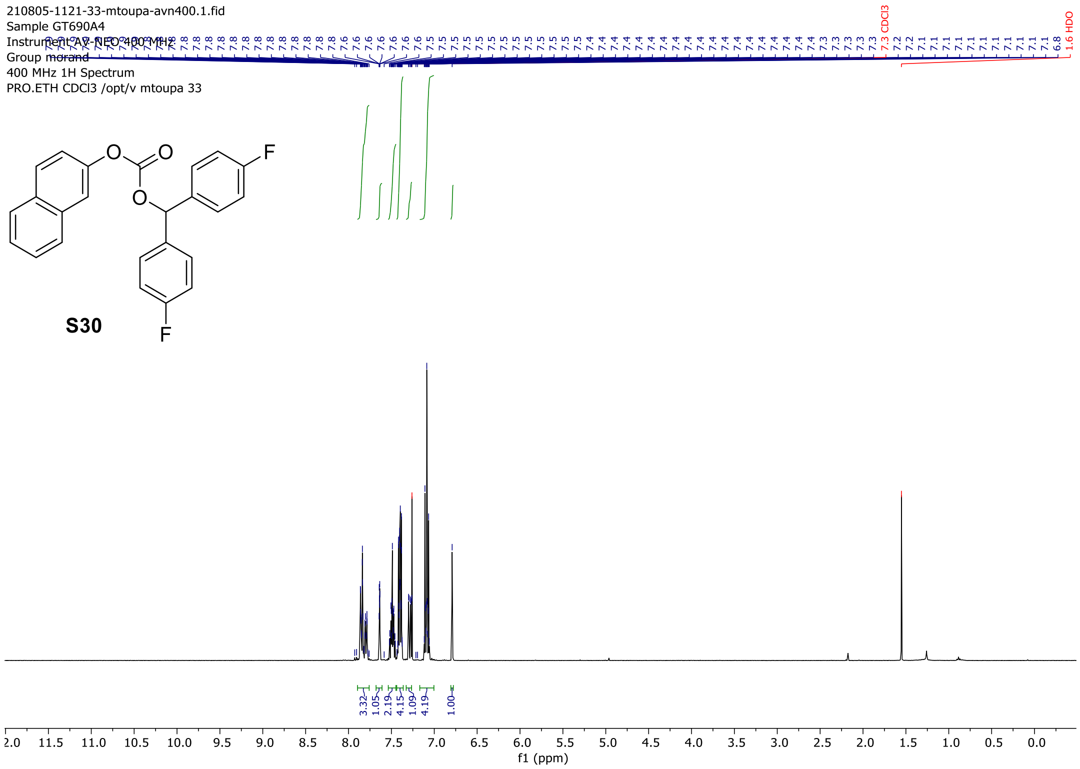

Supplementary Figure 72 ^{1}H NMR (400 MHz, $CDCl_3$, 25 °C) of compound **S30.**

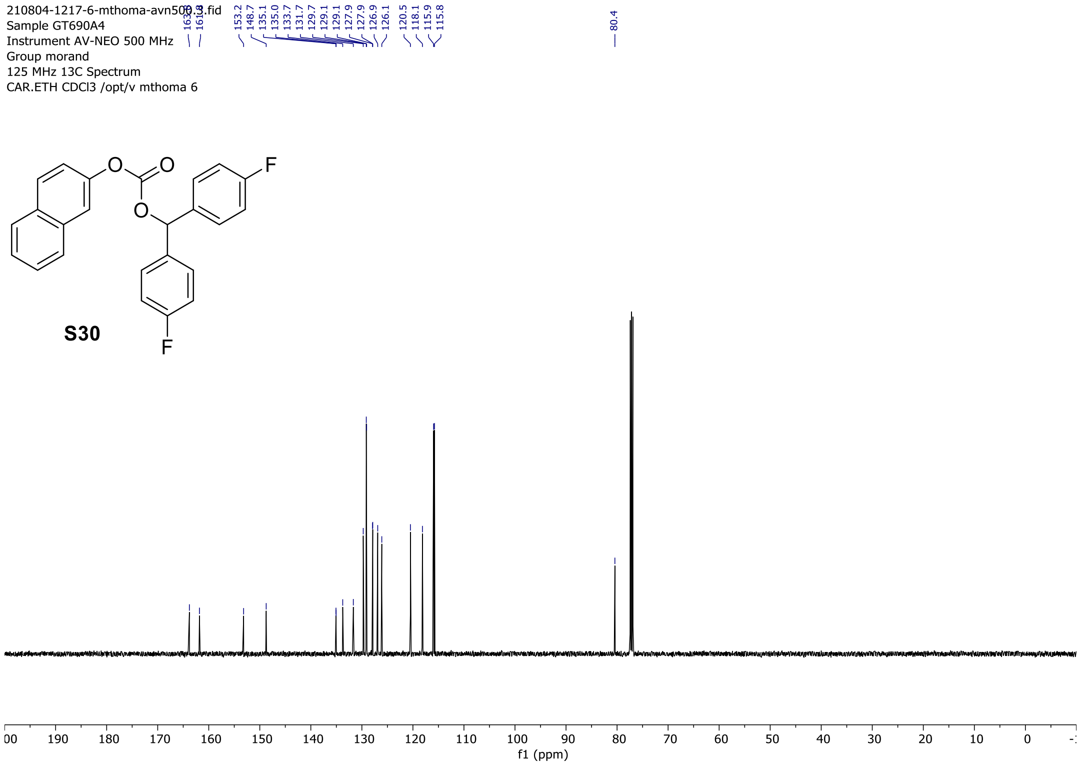

Supplementary Figure 73 ^{13}C NMR (125 MHz, $CDCl_3$, 25 °C) of compound **S30.**

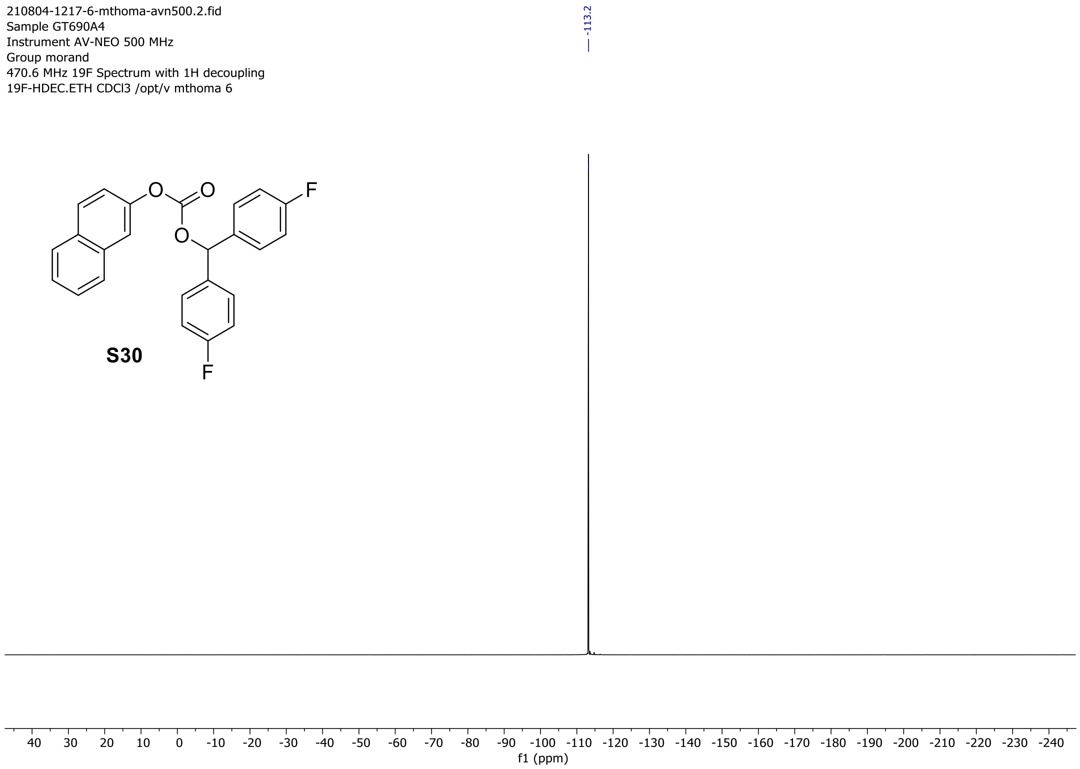

Supplementary Figure 74 ^{19}F NMR (471 MHz, $CDCl_3$, 25 °C) of compound **S30.**

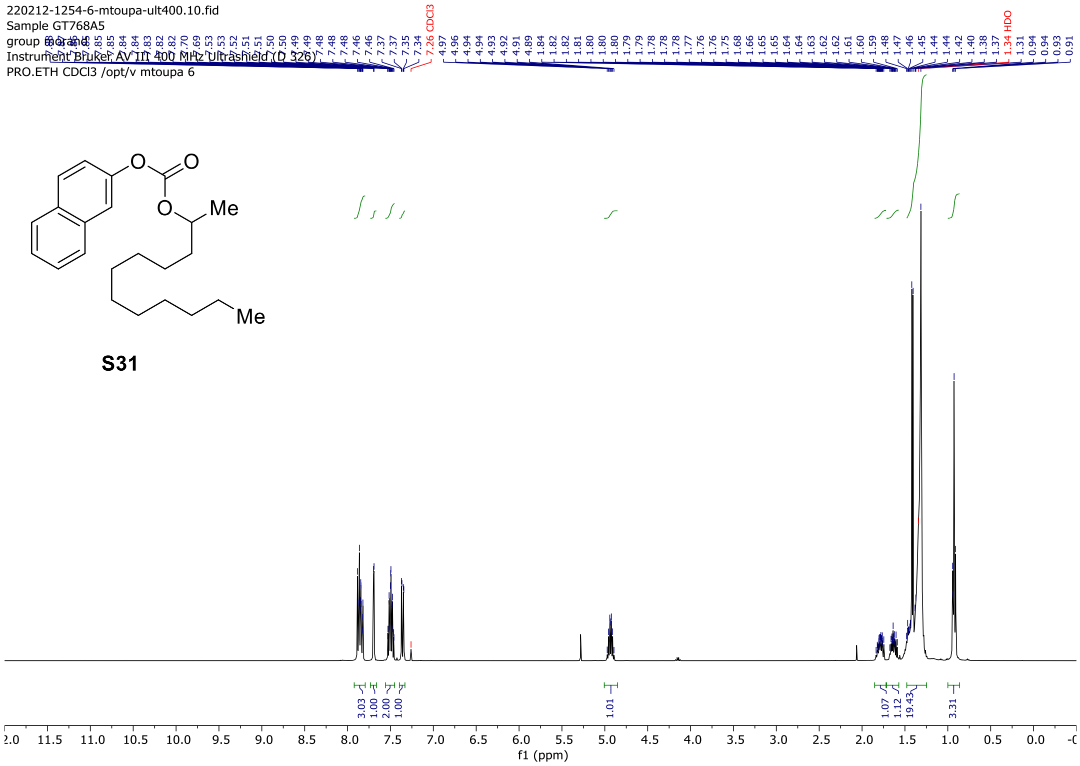

Supplementary Figure 75 ^{1}H NMR (400 MHz, $CDCl_3$, 25 °C) of compound **S31.**

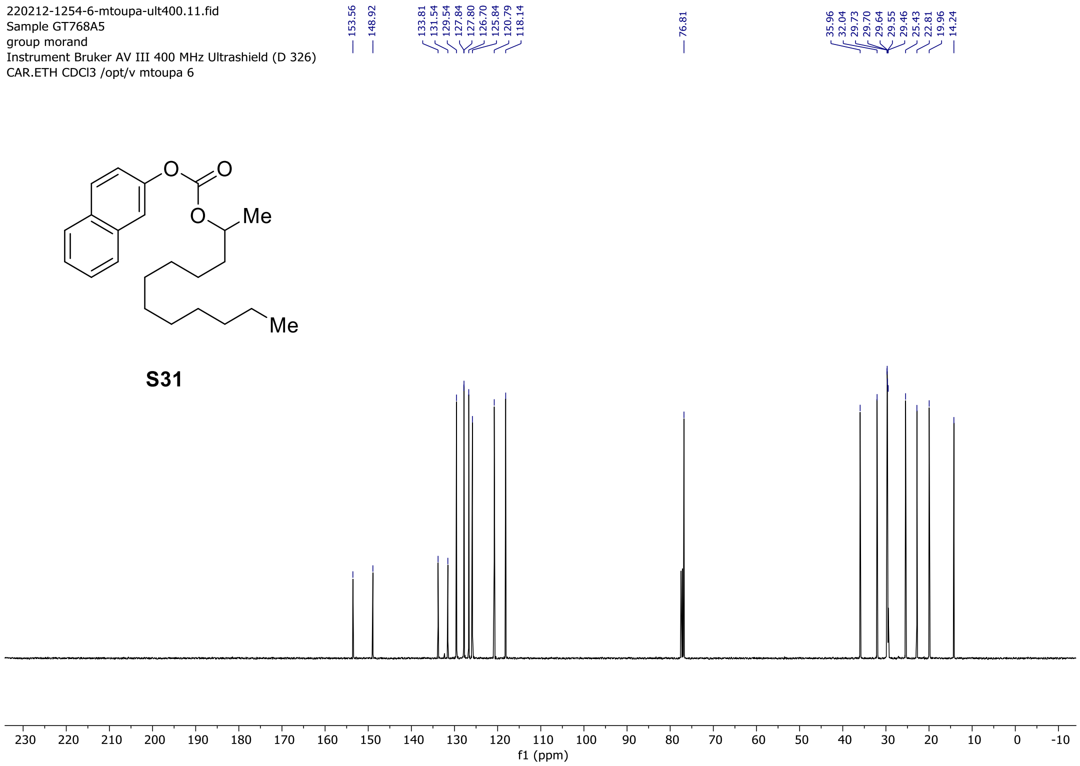

Supplementary Figure 76 ^{13}C NMR (100 MHz, $CDCl_3$, 25 °C) of compound **S31.**

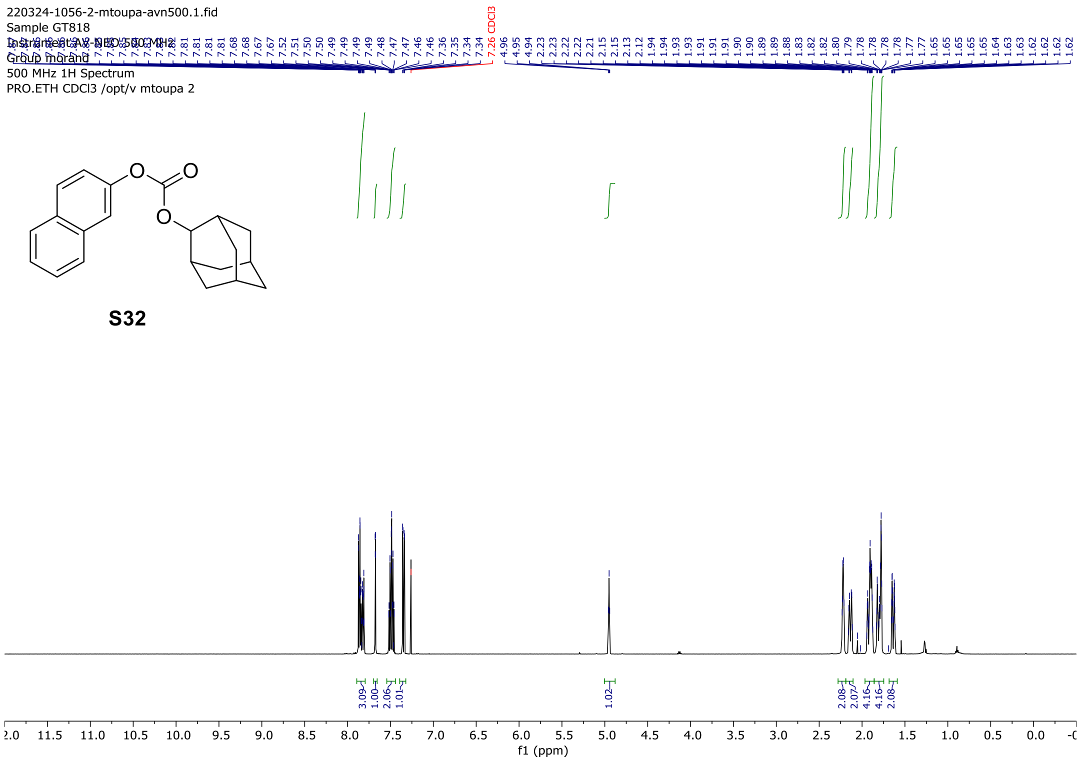

Supplementary Figure 77 ^{1}H NMR (500 MHz, $CDCl_3$, 25 °C) of compound **S32.**

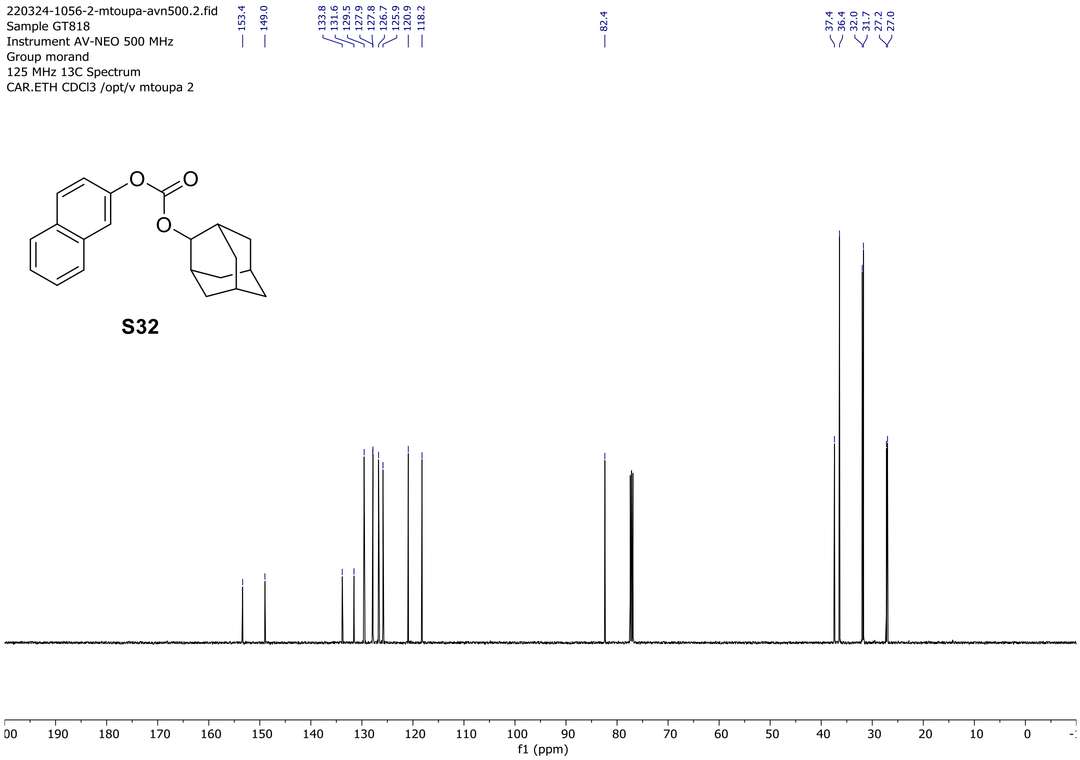

Supplementary Figure 78 ^{13}C NMR (125 MHz, $CDCl_3$, 25 °C) of compound **S32.**

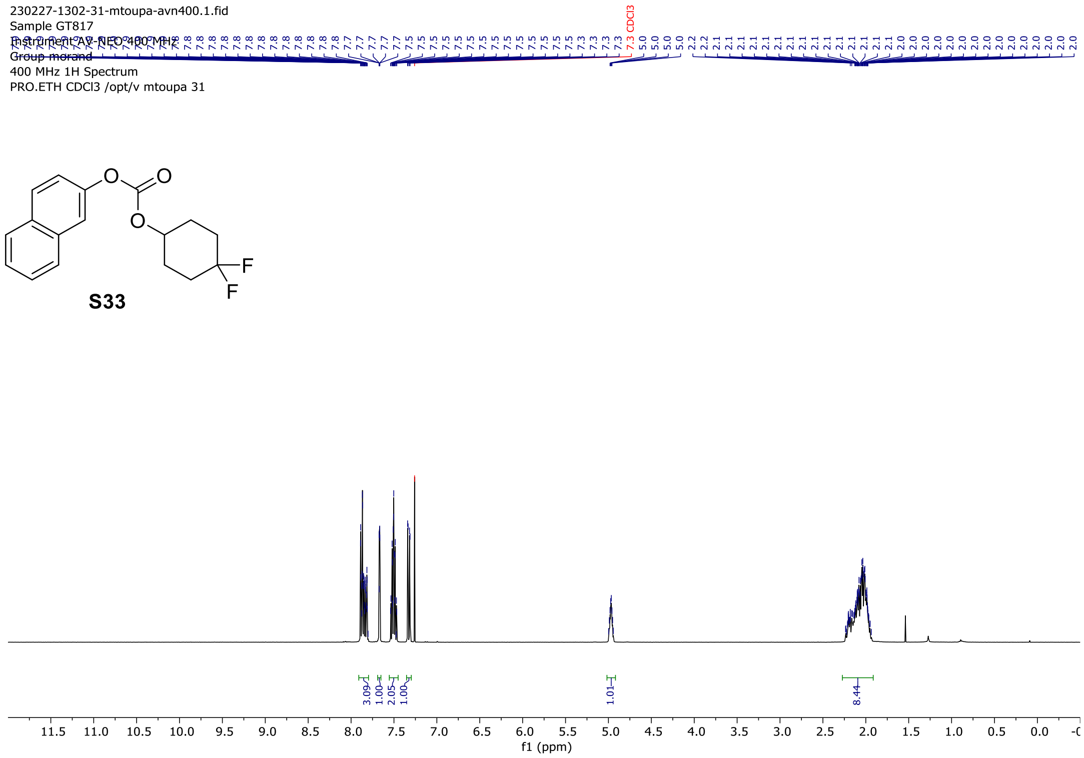

Supplementary Figure 79 ^{1}H NMR (400 MHz, $CDCl_3$, 25 °C) of compound **S33.**

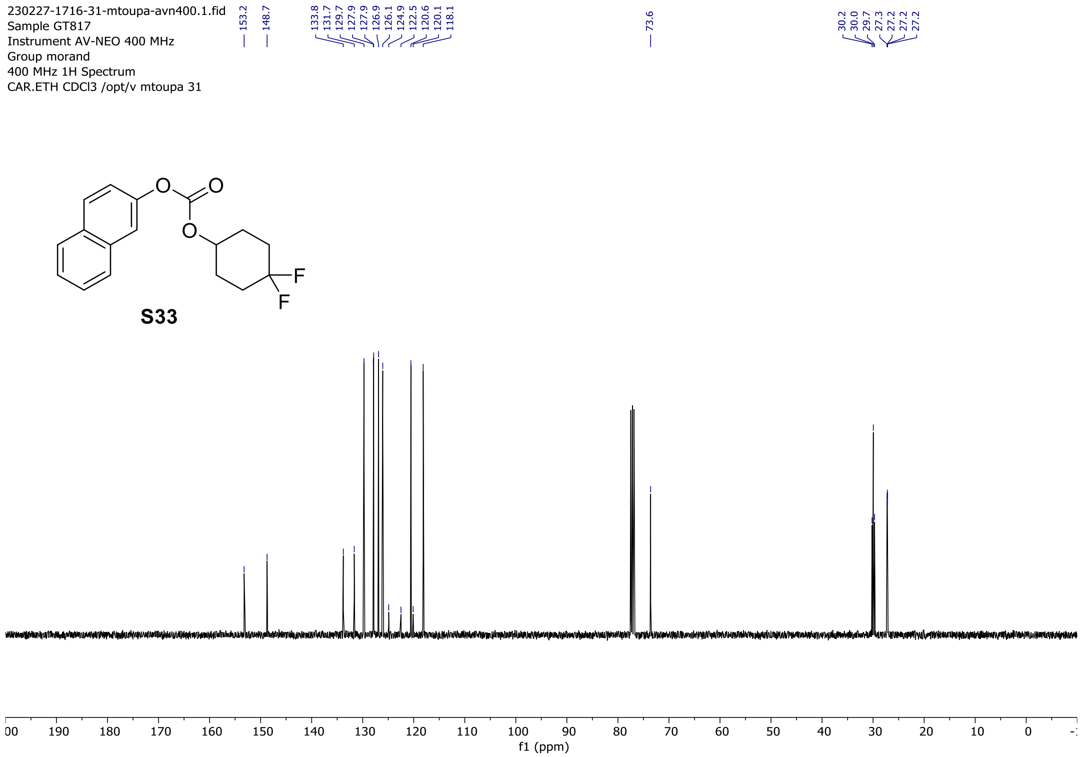

Supplementary Figure 80 ^{13}C NMR (100 MHz, $CDCl_3$, 25 °C) of compound **S33.**

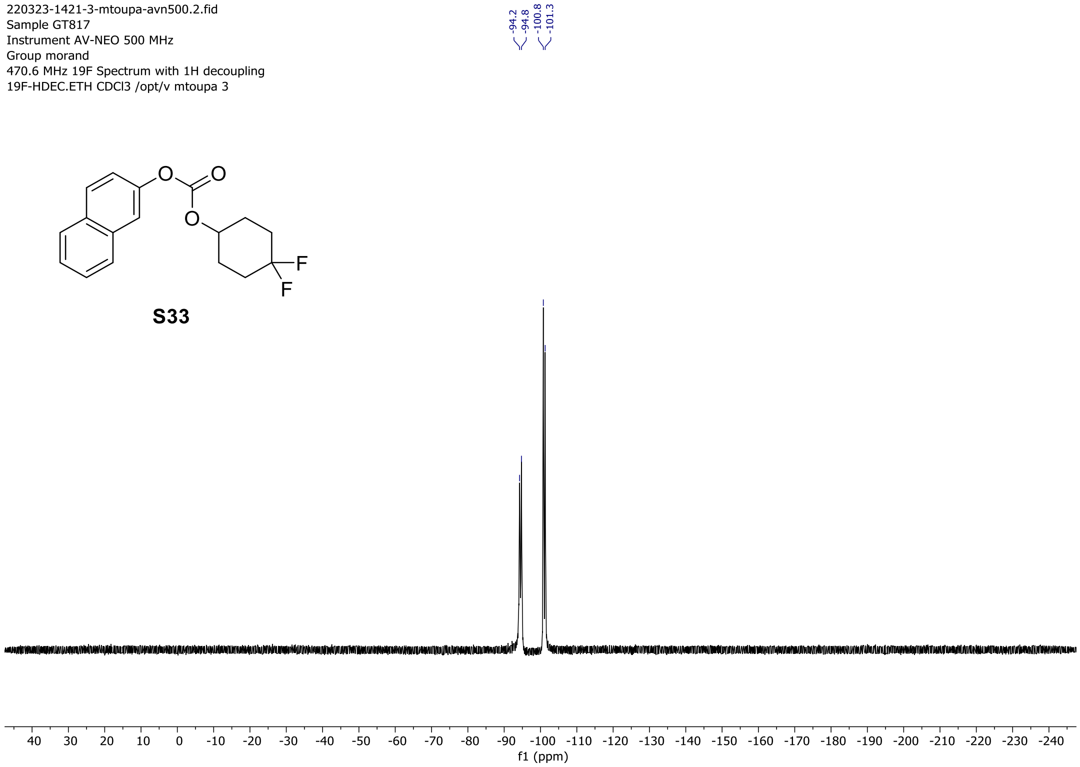

Supplementary Figure 81 ^{19}F NMR (471 MHz, $CDCl_3$, 25 °C) of compound **S33.**

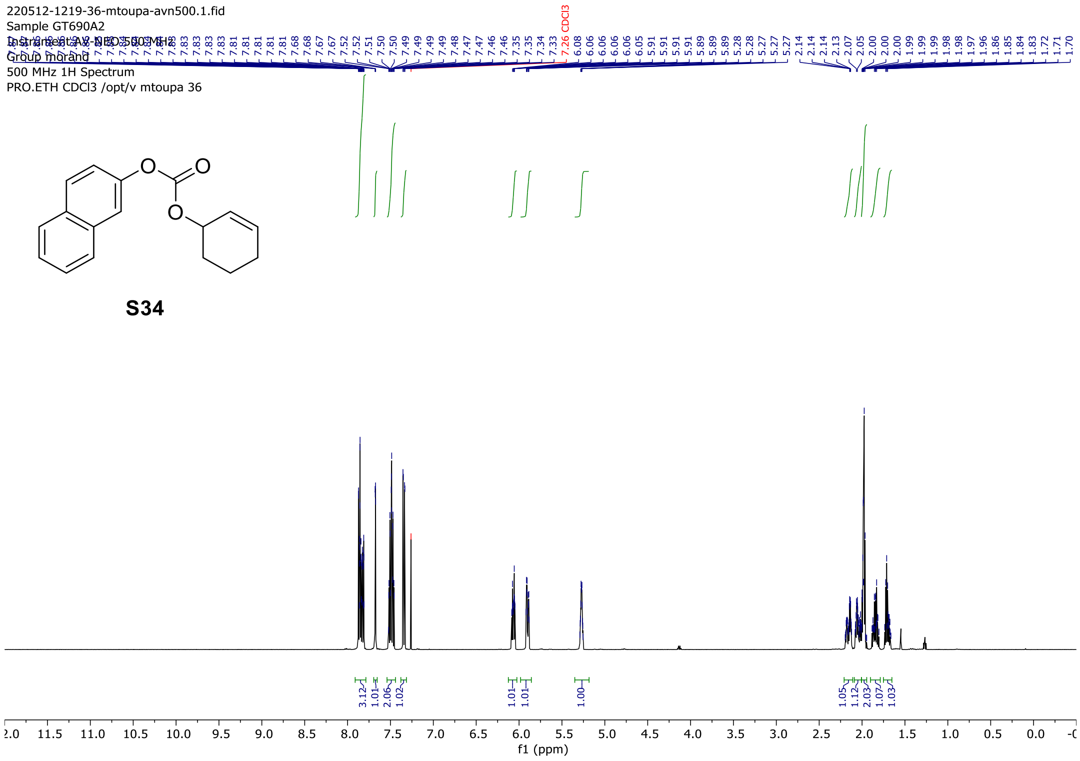

Supplementary Figure 82 ^{1}H NMR (500 MHz, $CDCl_3$, 25 °C) of compound **S34.**

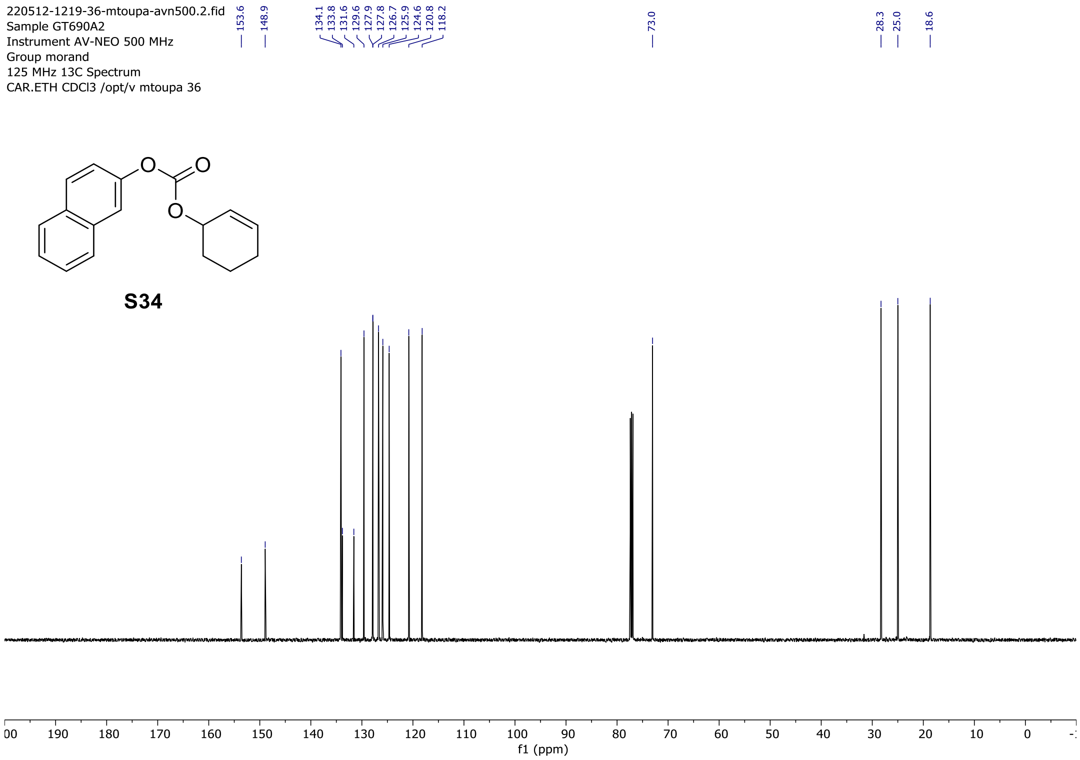

Supplementary Figure 83 ^{13}C NMR (125 MHz, $CDCl_3$, 25 °C) of compound **S34.**

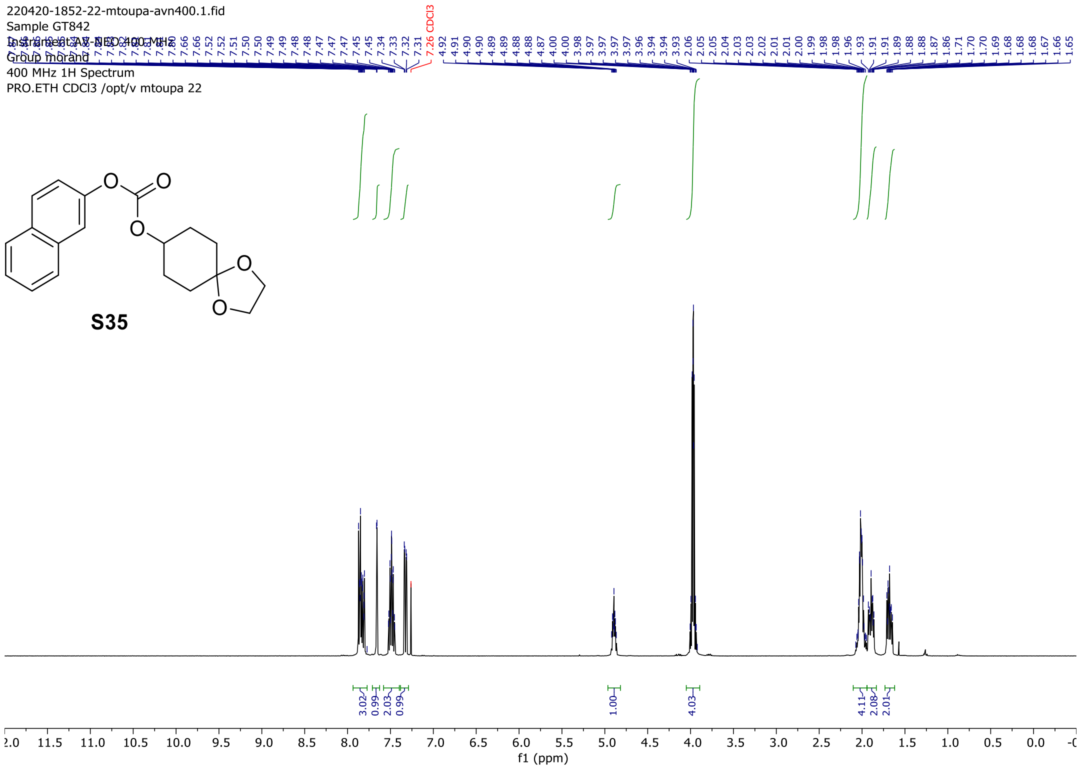

Supplementary Figure 84 ^{1}H NMR (400 MHz, $CDCl_3$, 25 °C) of compound **S35.**

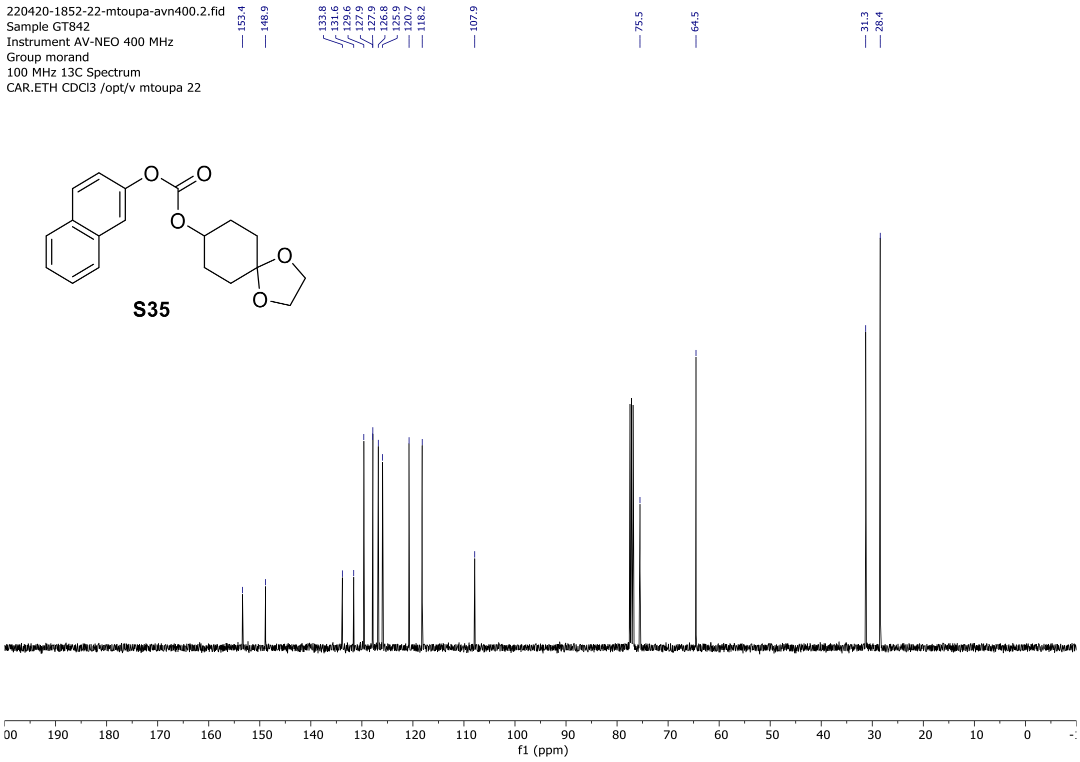

Supplementary Figure 85 ^{13}C NMR (100 MHz, $CDCl_3$, 25 °C) of compound **S35.**

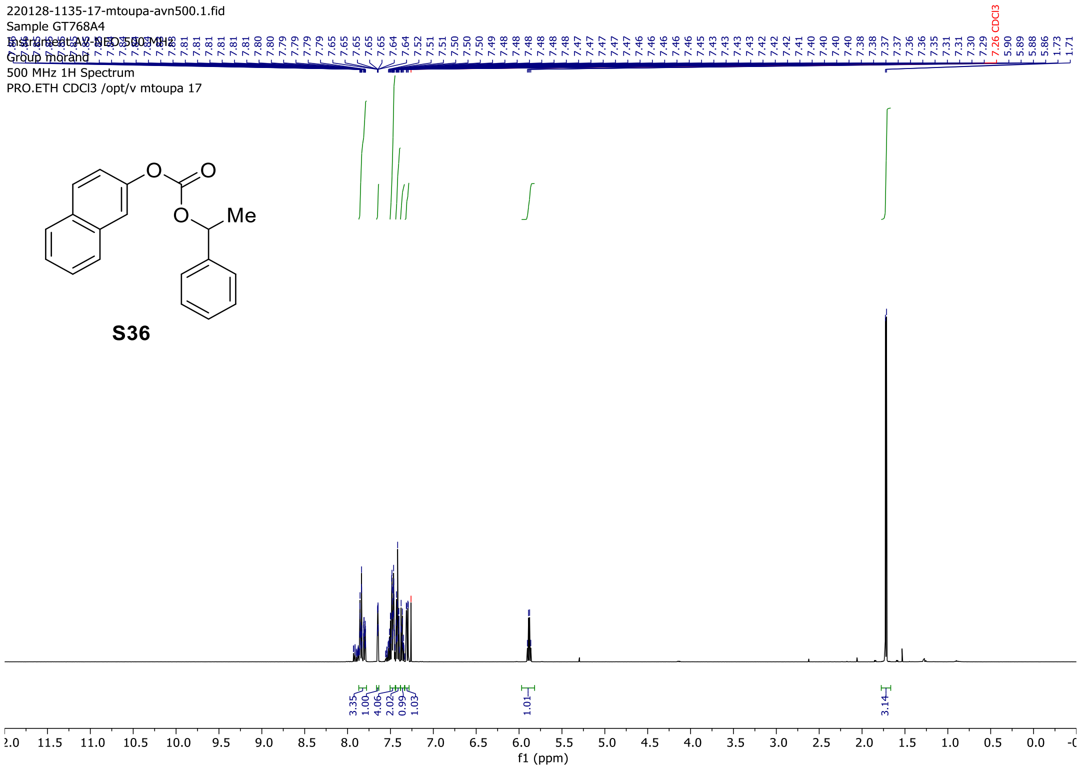

Supplementary Figure 86 ^{1}H NMR (500 MHz, $CDCl_3$, 25 °C) of compound **S36.**

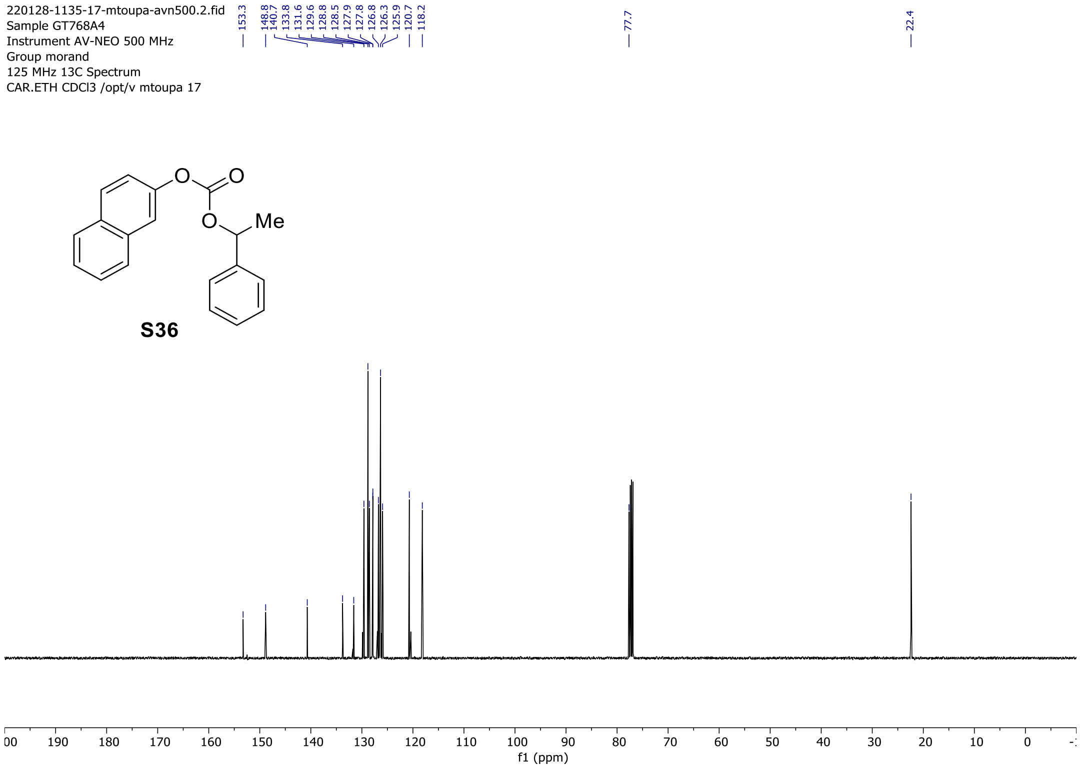

Supplementary Figure 87 ^{13}C NMR (125 MHz, $CDCl_3$, 25 °C) of compound **S36.**

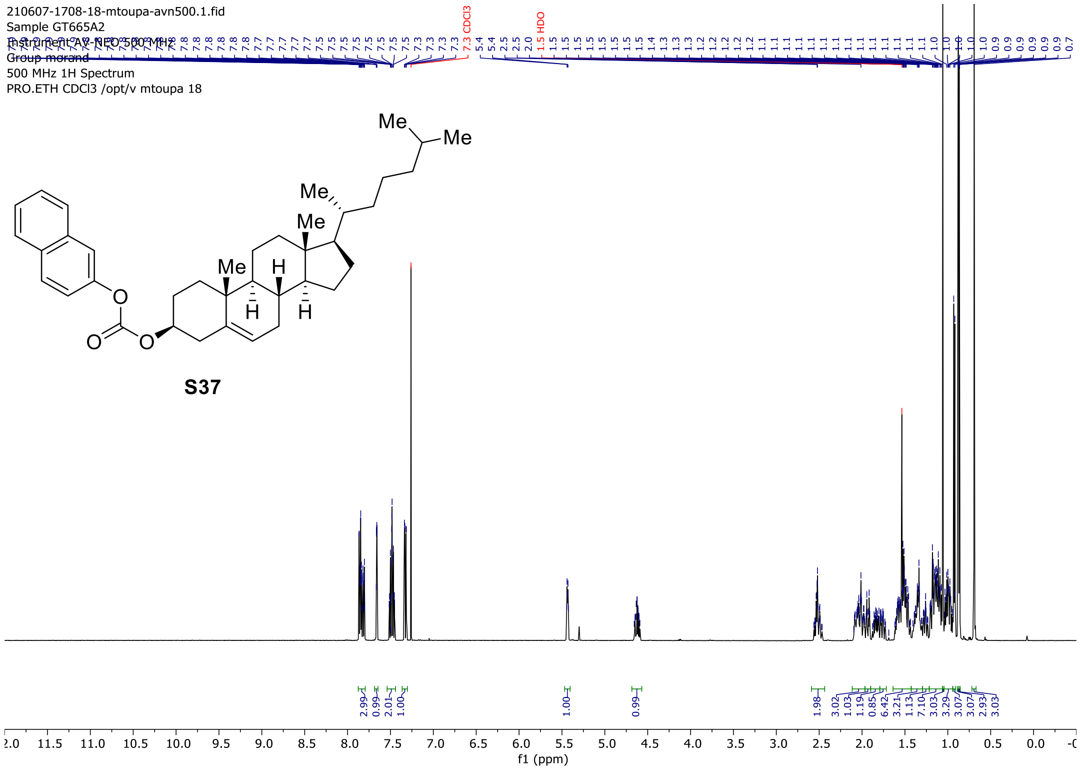

Supplementary Figure 88 ^{1}H NMR (500 MHz, $CDCl_3$, 25 °C) of compound **S37.**

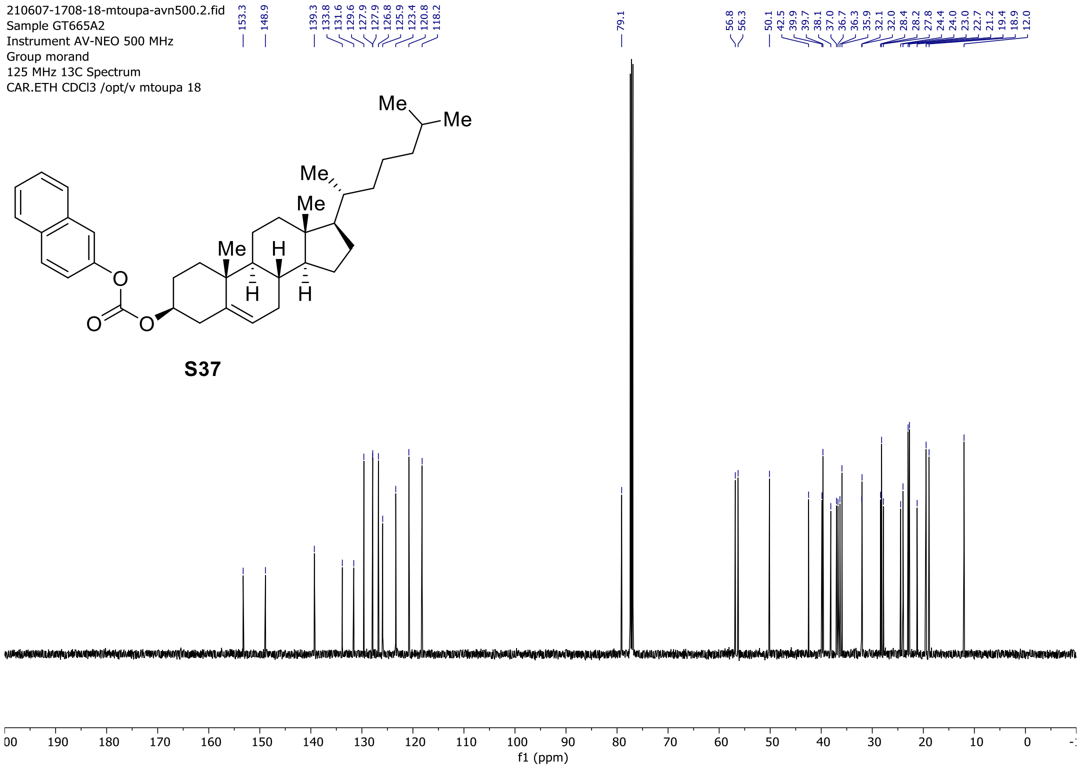

Supplementary Figure 89 ^{13}C NMR (125 MHz, $CDCl_3$, 25 °C) of compound **S37.**

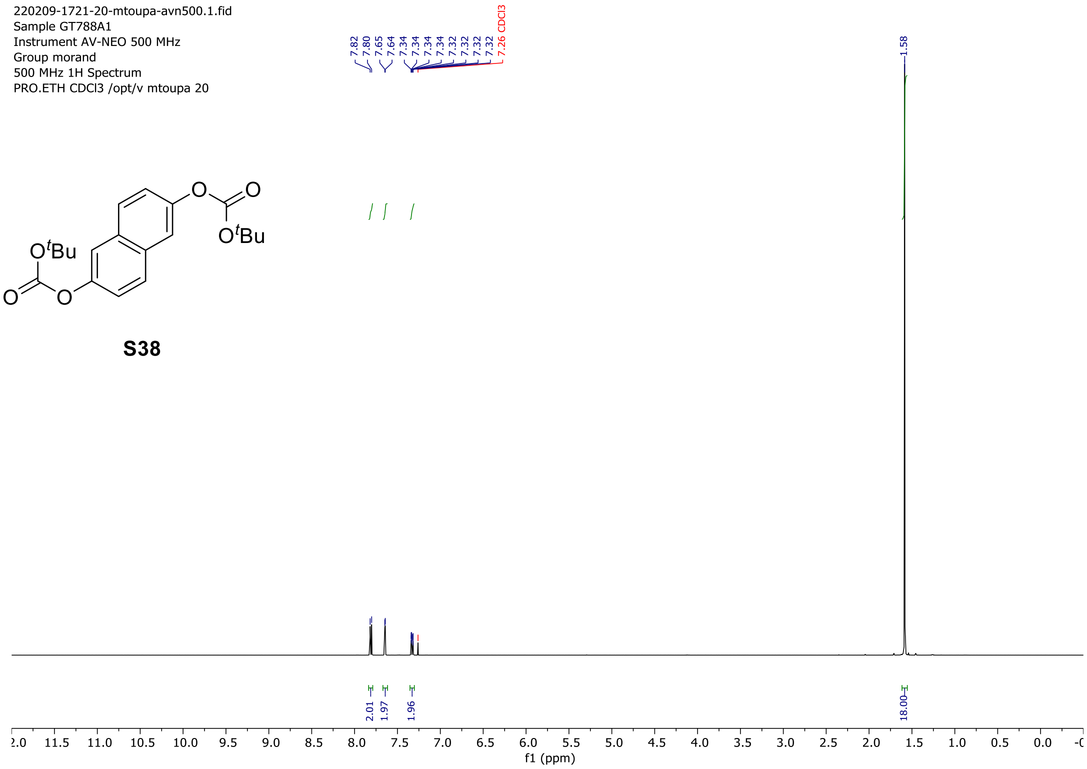

Supplementary Figure 90 ^{1}H NMR (500 MHz, $CDCl_3$, 25 °C) of compound **S38.**

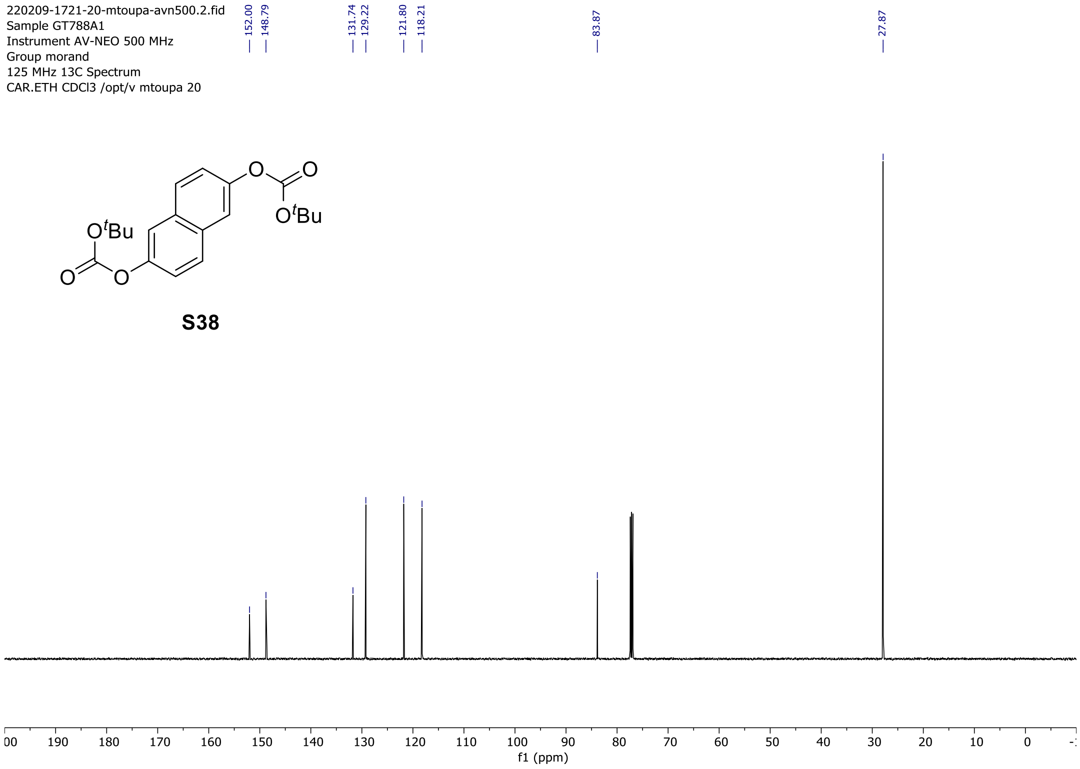

Supplementary Figure 91 ^{13}C NMR (125 MHz, $CDCl_3$, 25 °C) of compound **S38.**

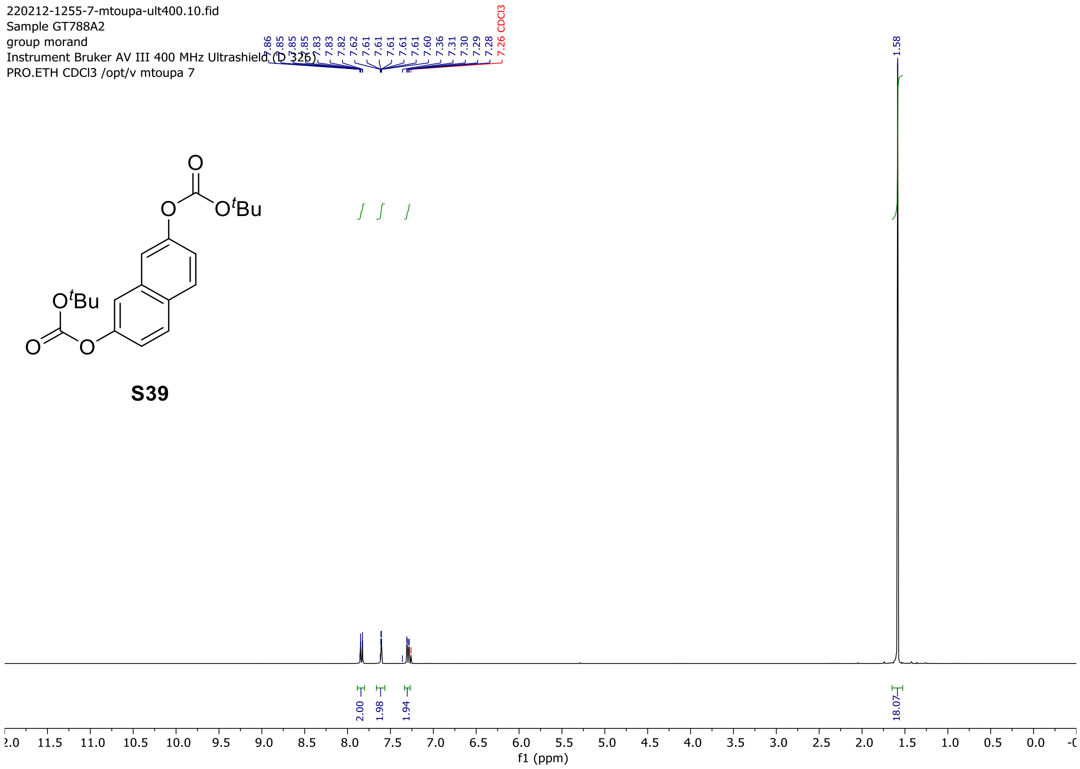

Supplementary Figure 92 ^{1}H NMR (400 MHz, $CDCl_3$, 25 °C) of compound **S39.**

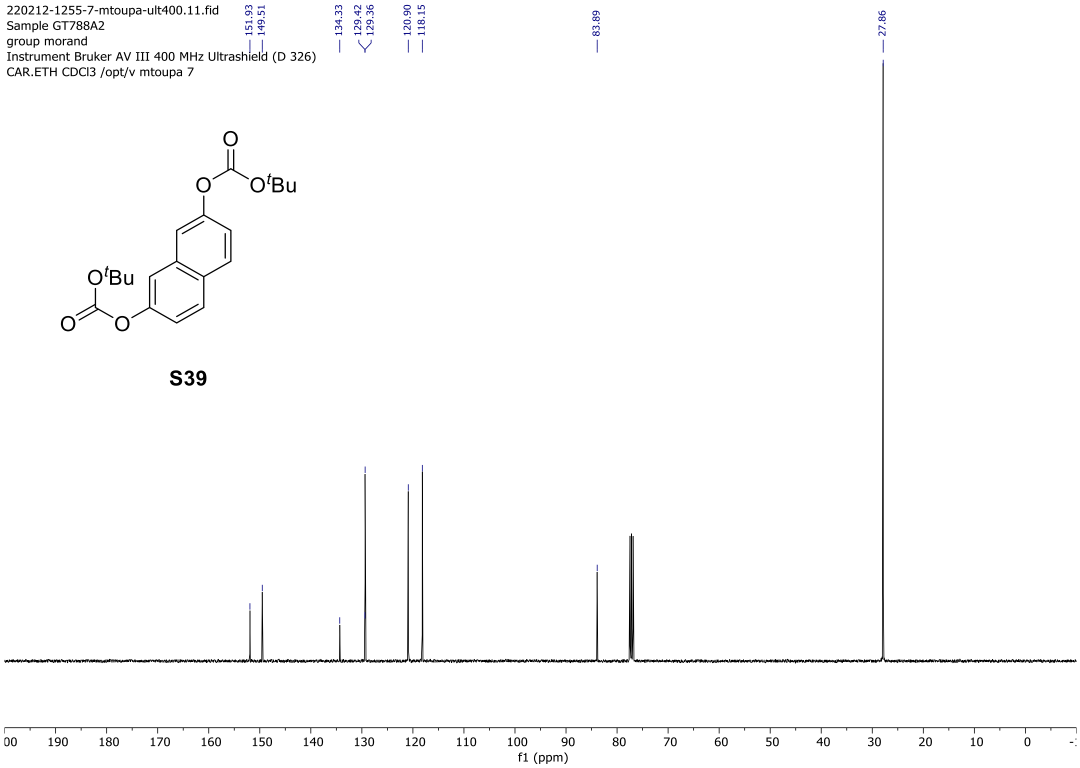

Supplementary Figure 93 ^{13}C NMR (100 MHz, $CDCl_3$, 25 °C) of compound **S39.**

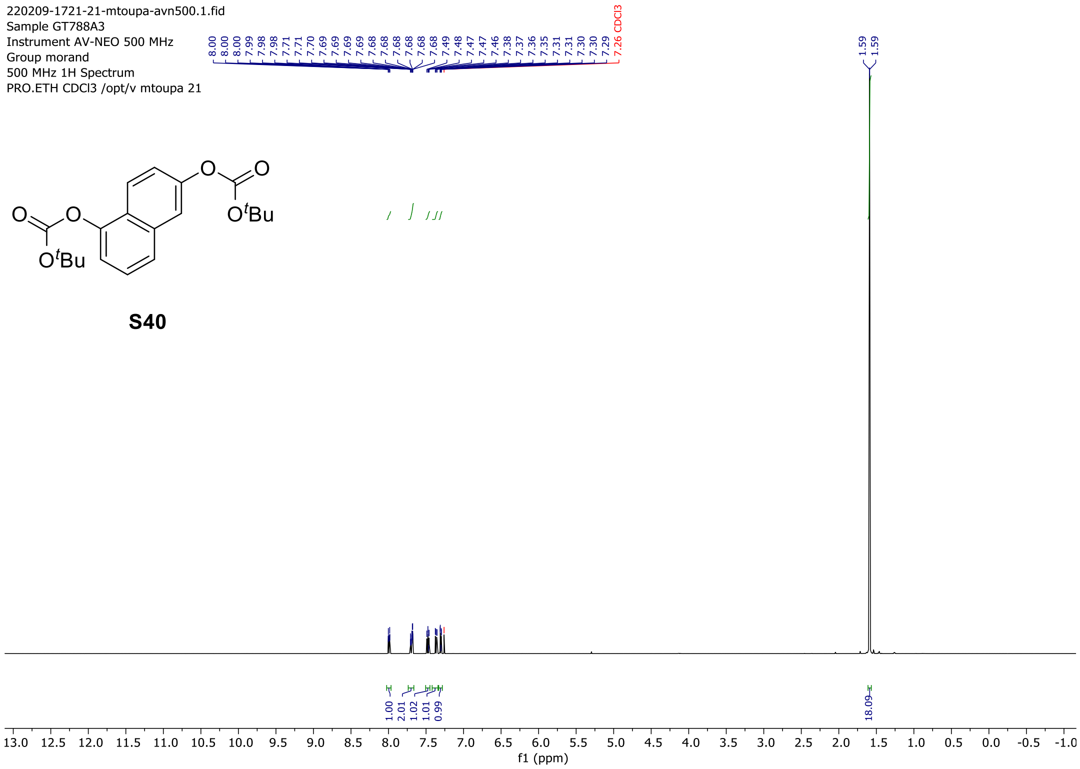

Supplementary Figure 94 ^{1}H NMR (500 MHz, $CDCl_3$, 25 °C) of compound **S40.**

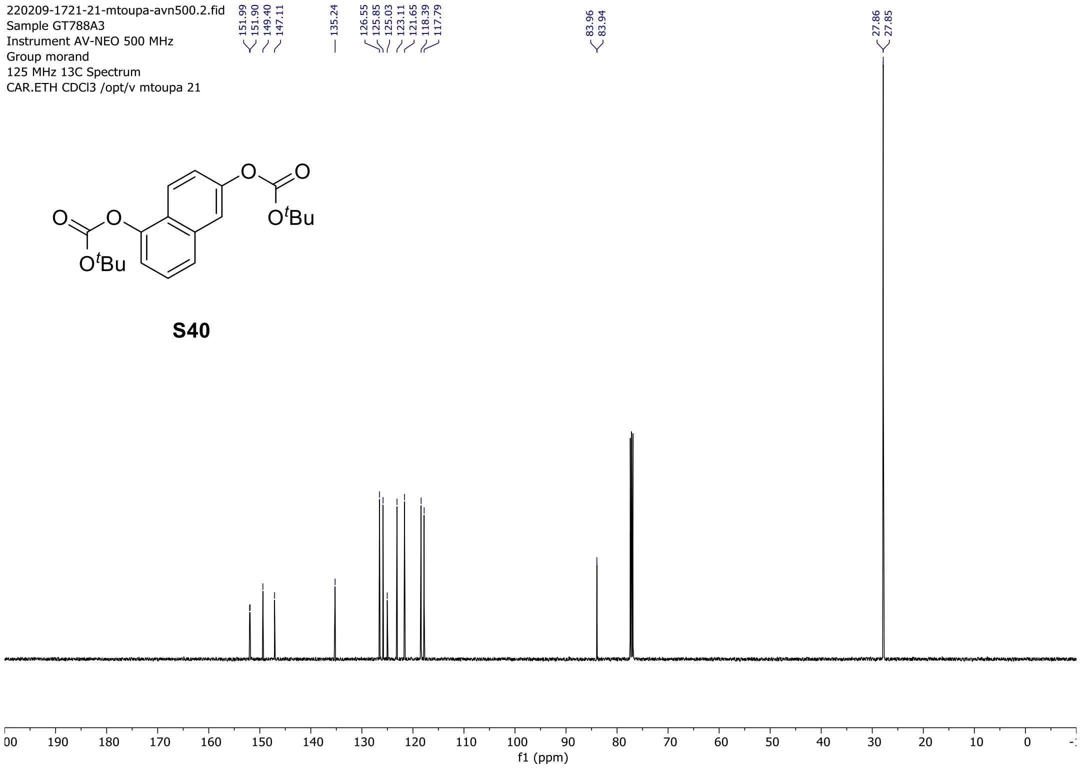

Supplementary Figure 95 ^{13}C NMR (125 MHz, $CDCl_3$, 25 °C) of compound **S40.**

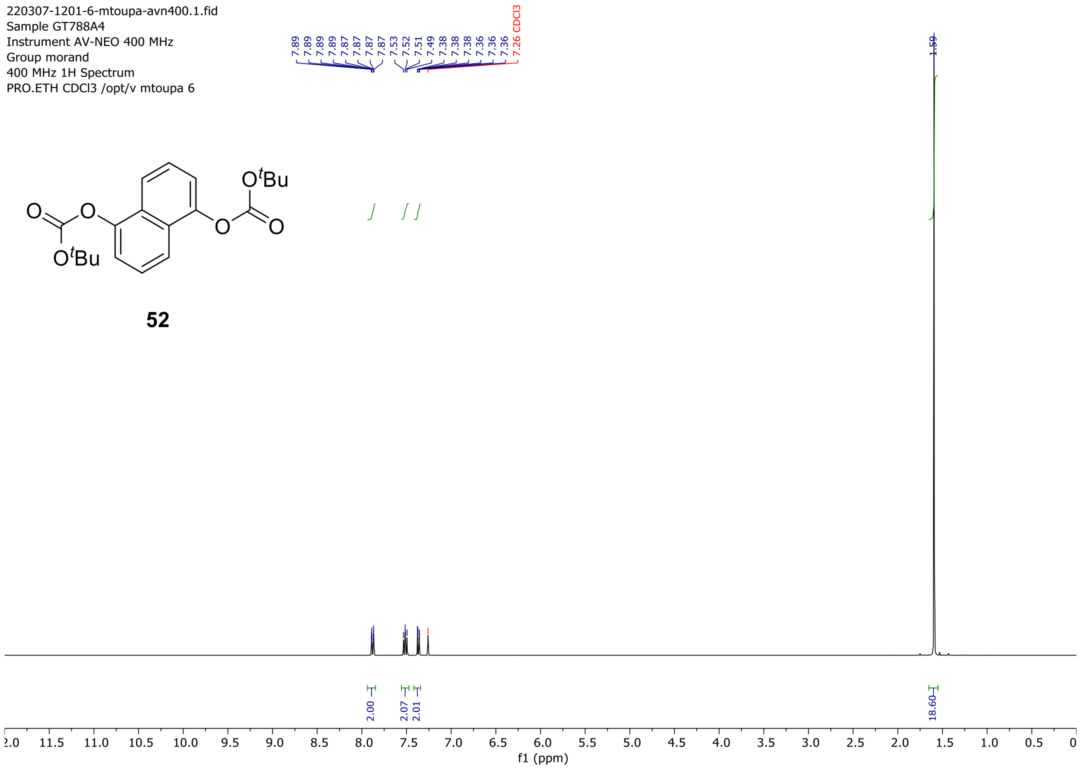

Supplementary Figure 96 ^{1}H NMR (400 MHz, $CDCl_3$, 25 °C) of compound **52.**

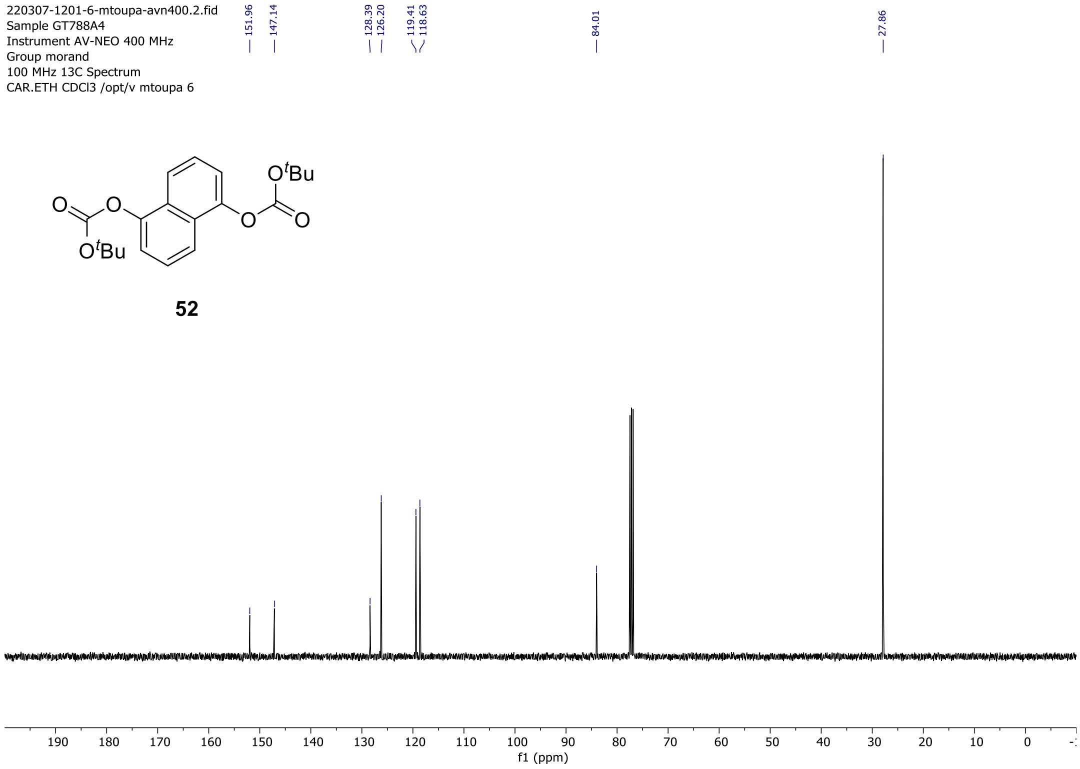

Supplementary Figure 97 ^{13}C NMR (100 MHz, $CDCl_3$, 25 °C) of compound **52.**

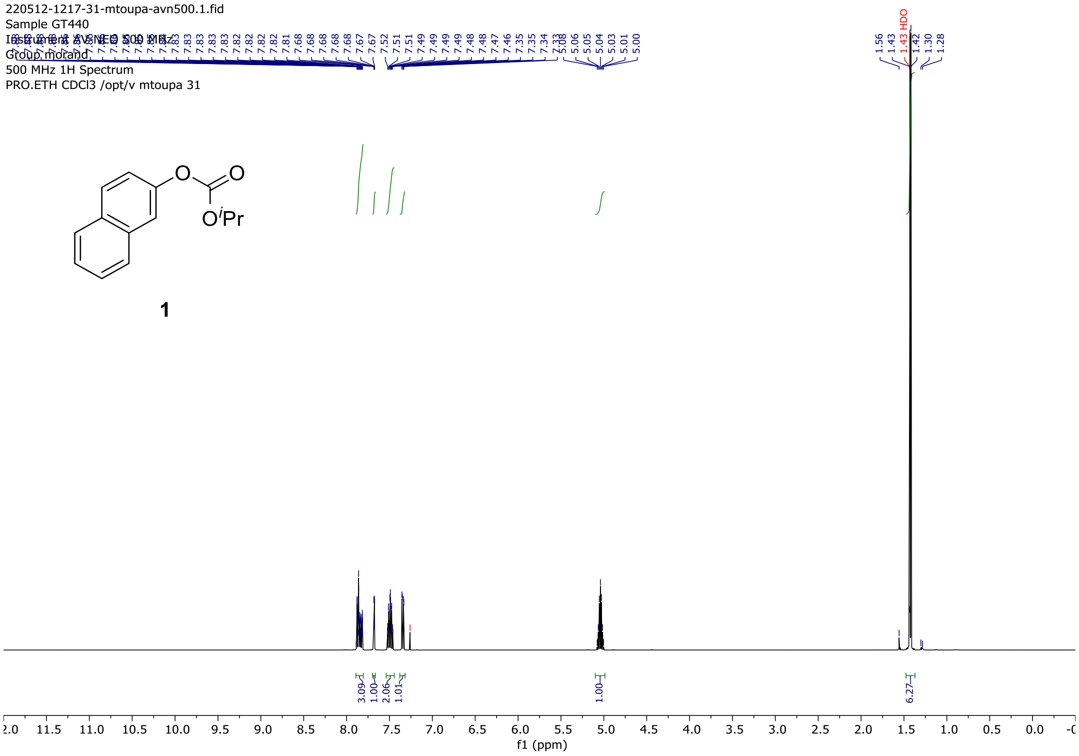

Supplementary Figure 98 ^{1}H NMR (500 MHz, $CDCl_3$, 25 °C) of compound **1.**

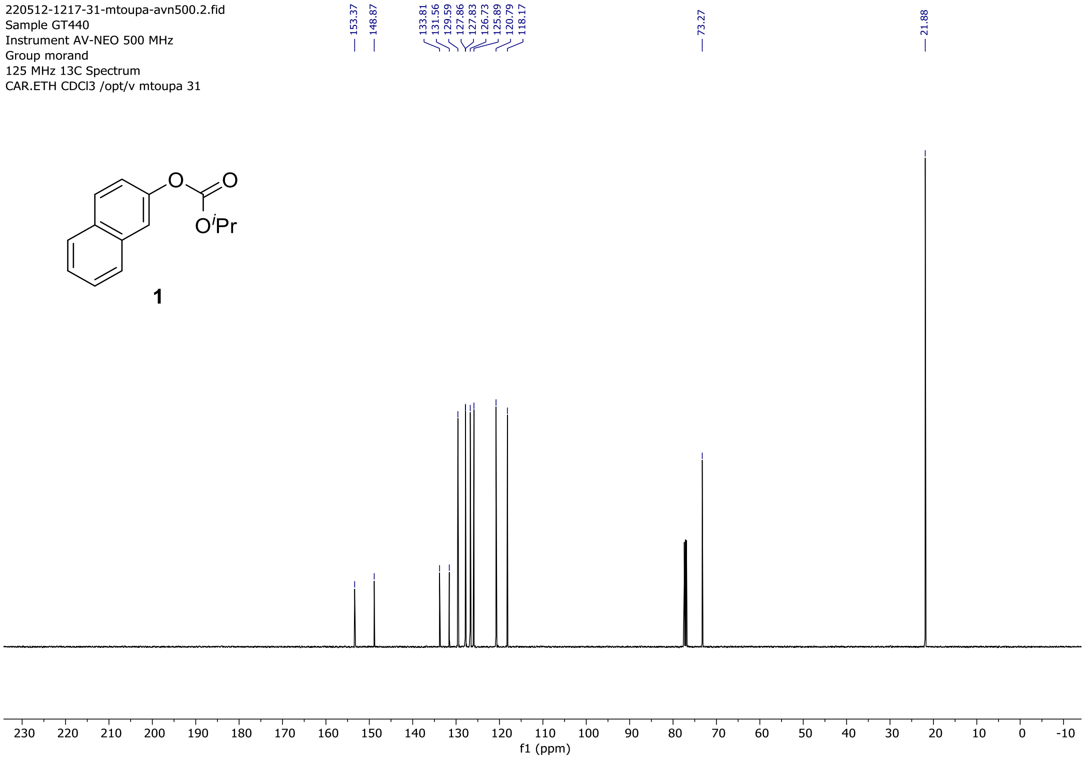

Supplementary Figure 99 ^{13}C NMR (125 MHz, $CDCl_3$, 25 °C) of compound **1.**

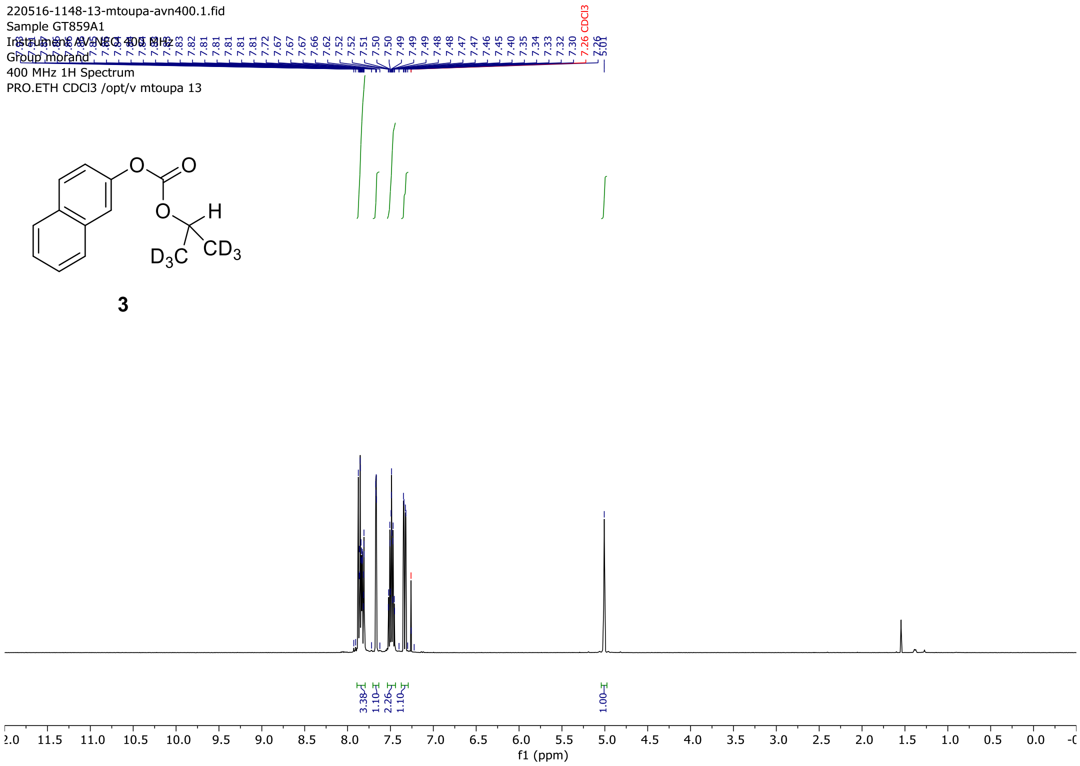

Supplementary Figure 100 ^{1}H NMR (400 MHz, $CDCl_3$, 25 °C) of compound **3.**

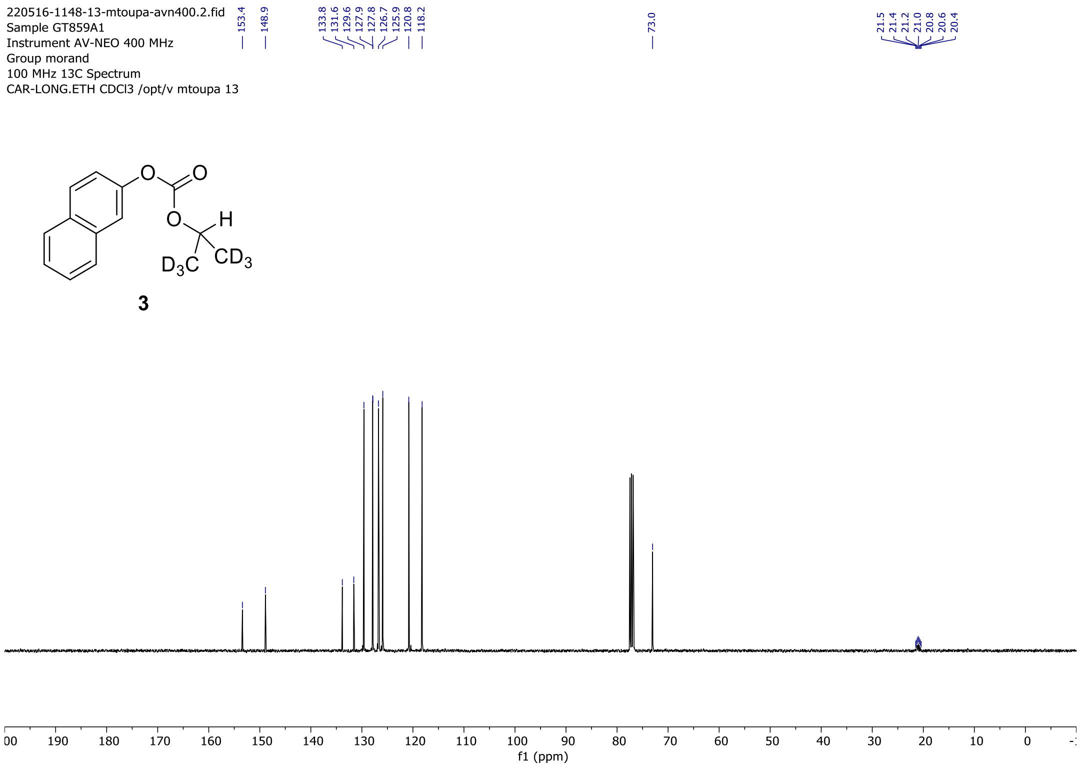

Supplementary Figure 101 ^{13}C NMR (100 MHz, $CDCl_3$, 25 °C) of compound **3.**

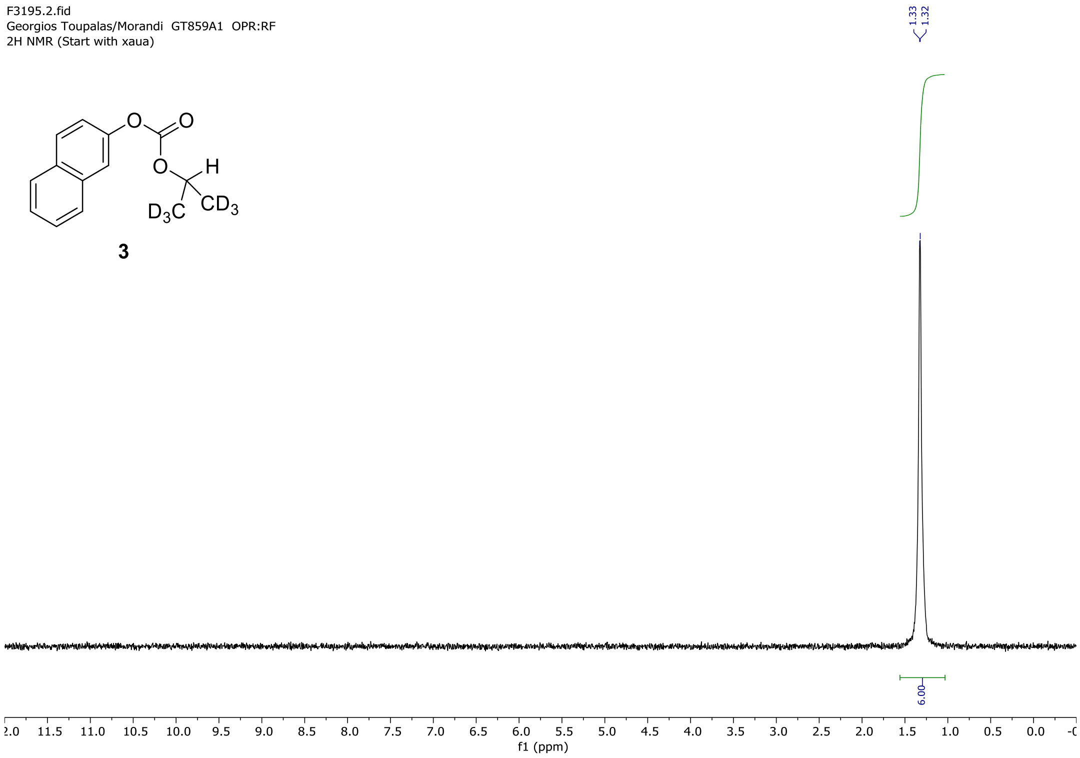

Supplementary Figure 102 ^{2}H NMR (77 MHz, $CHCl_3$, 25 °C) of compound **3.**

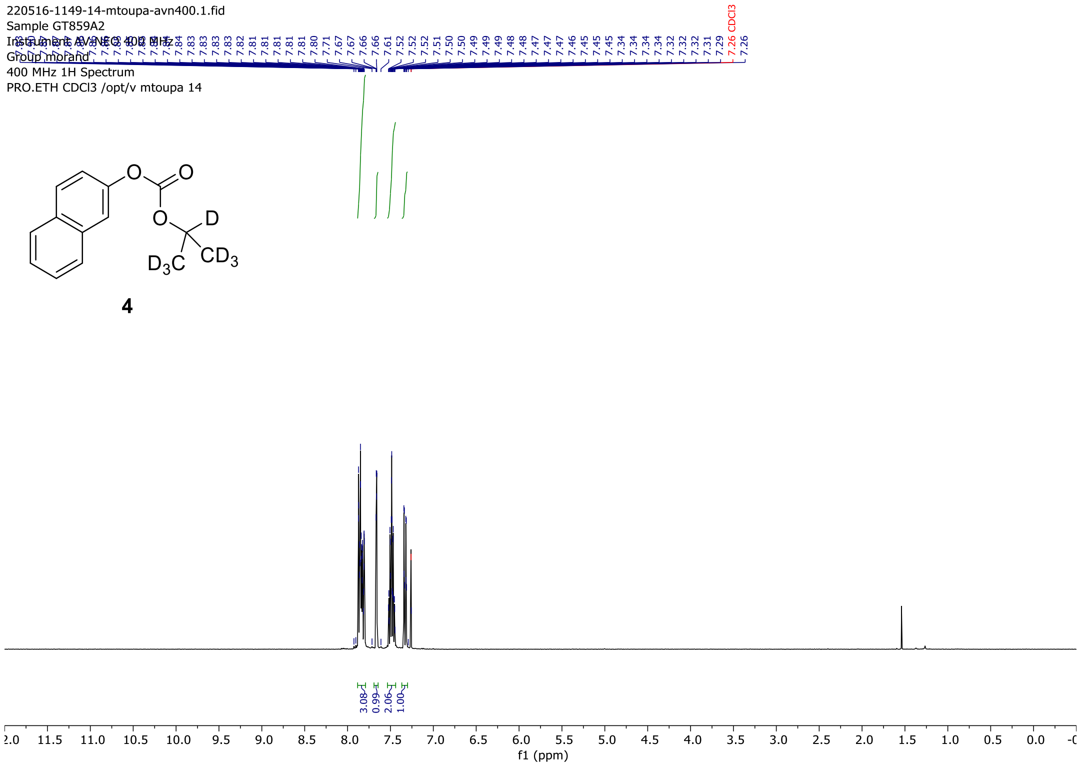

Supplementary Figure 103 ^{1}H NMR (400 MHz, $CDCl_3$, 25 °C) of compound **4.**

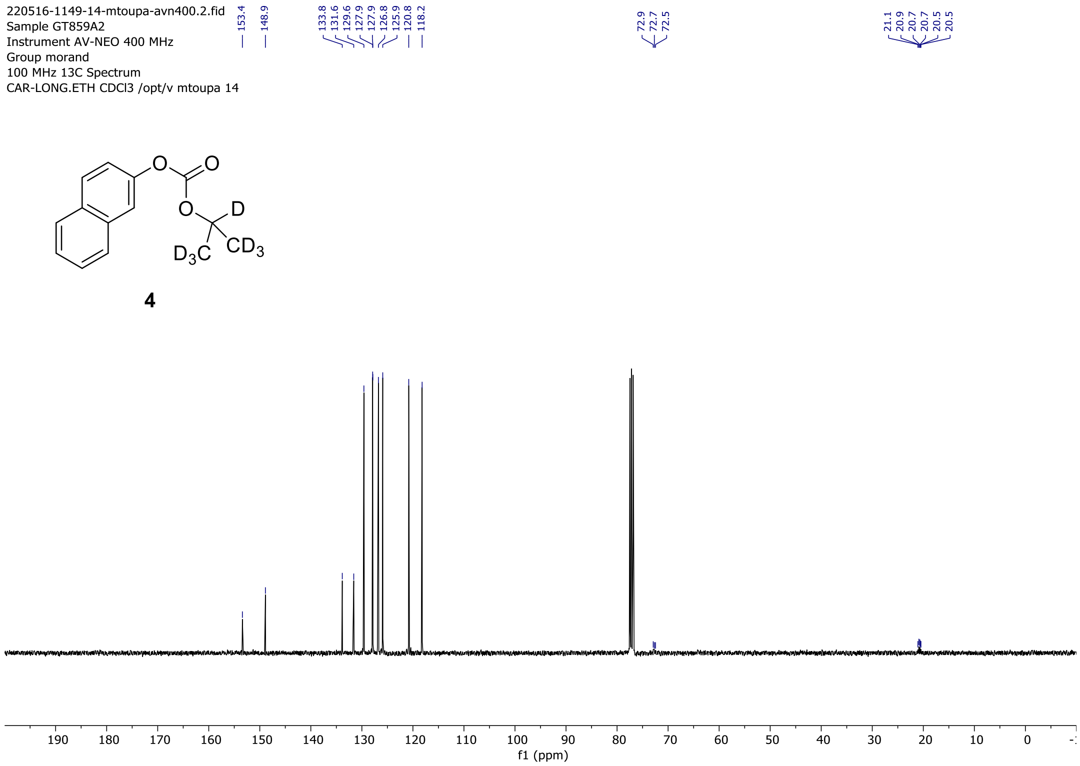

Supplementary Figure 104 ^{13}C NMR (100 MHz, $CDCl_3$, 25 °C) of compound **4.**

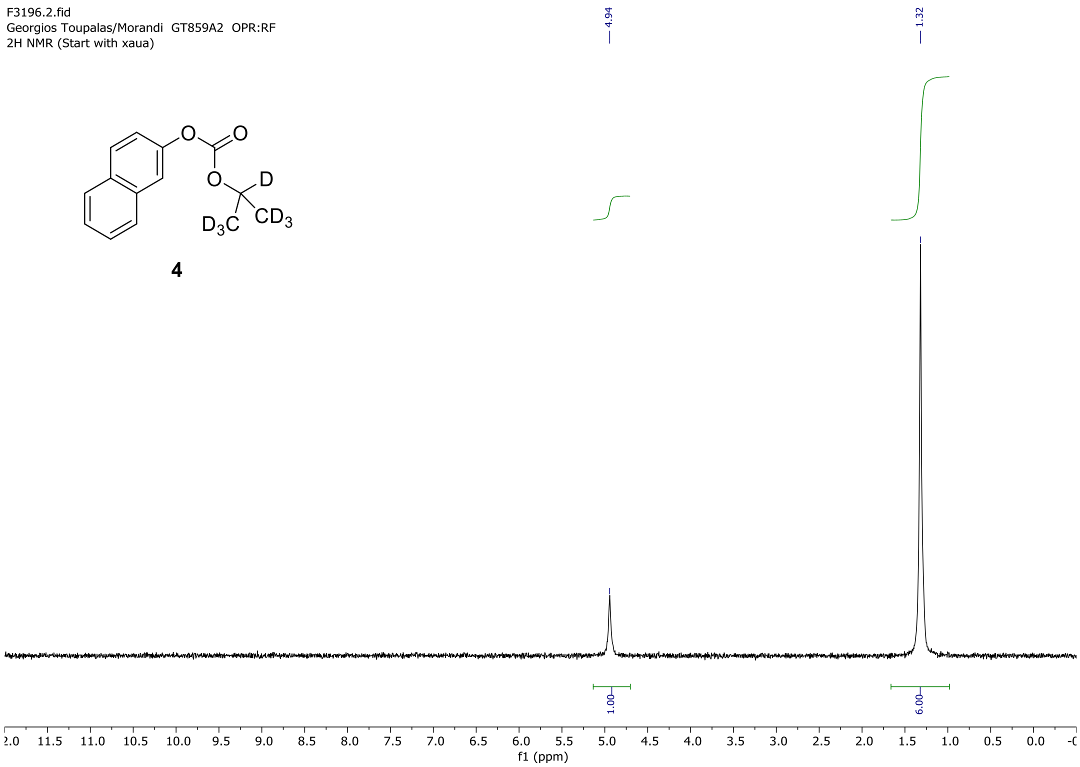

Supplementary Figure 105 ^{2}H NMR (77 MHz, $CHCl_3$, 25 °C) of compound **4.**

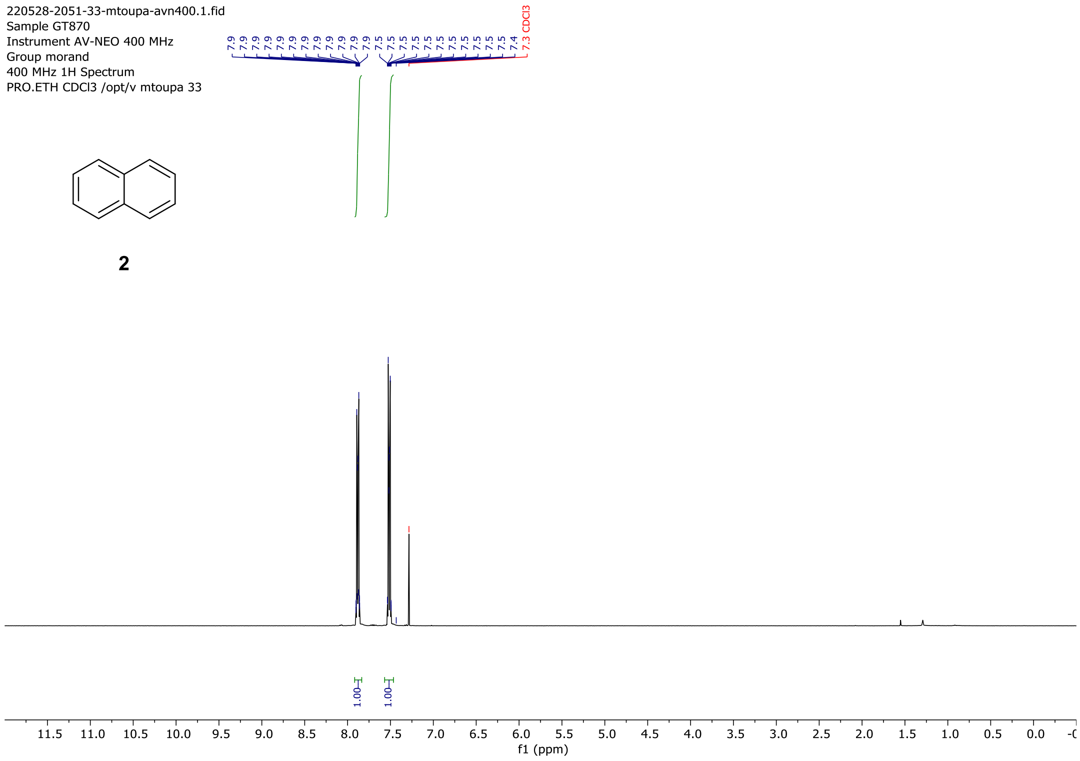

Supplementary Figure 106 ^{1}H NMR (400 MHz, $CDCl_3$, 25 °C) of compound **2.**

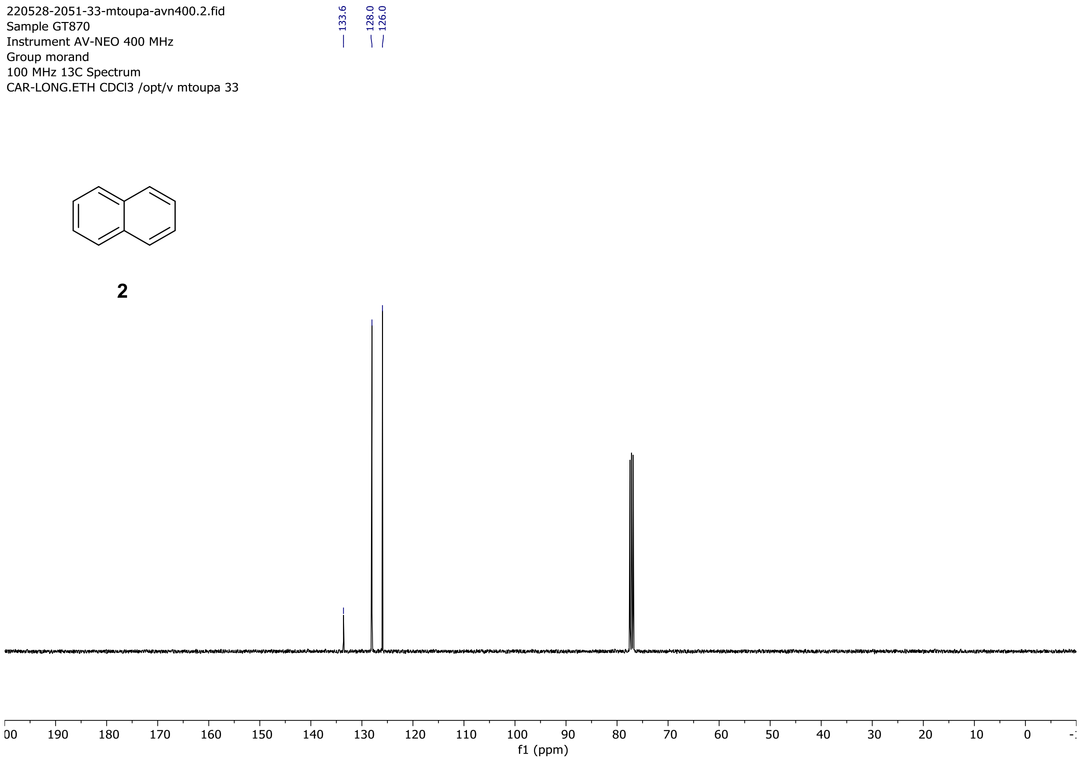

Supplementary Figure 107 ^{13}C NMR (100 MHz, $CDCl_3$, 25 °C) of compound **2.**

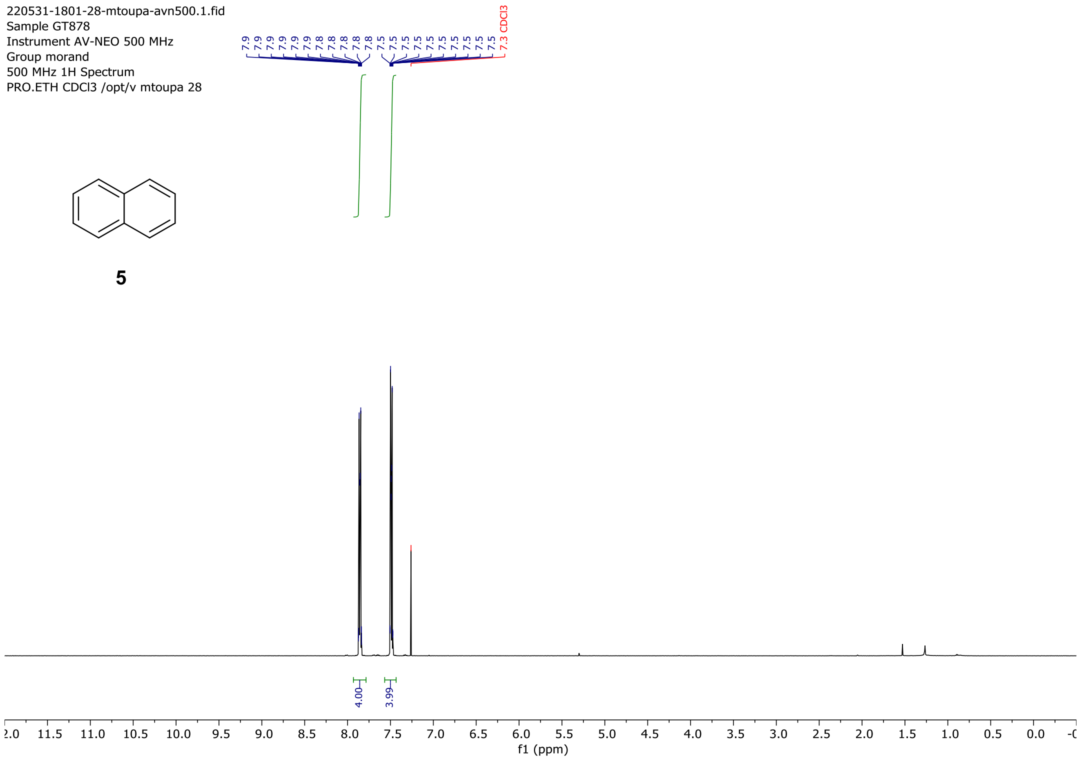

Supplementary Figure 108 ^{1}H NMR (500 MHz, $CDCl_3$, 25 °C) of compound **5.**

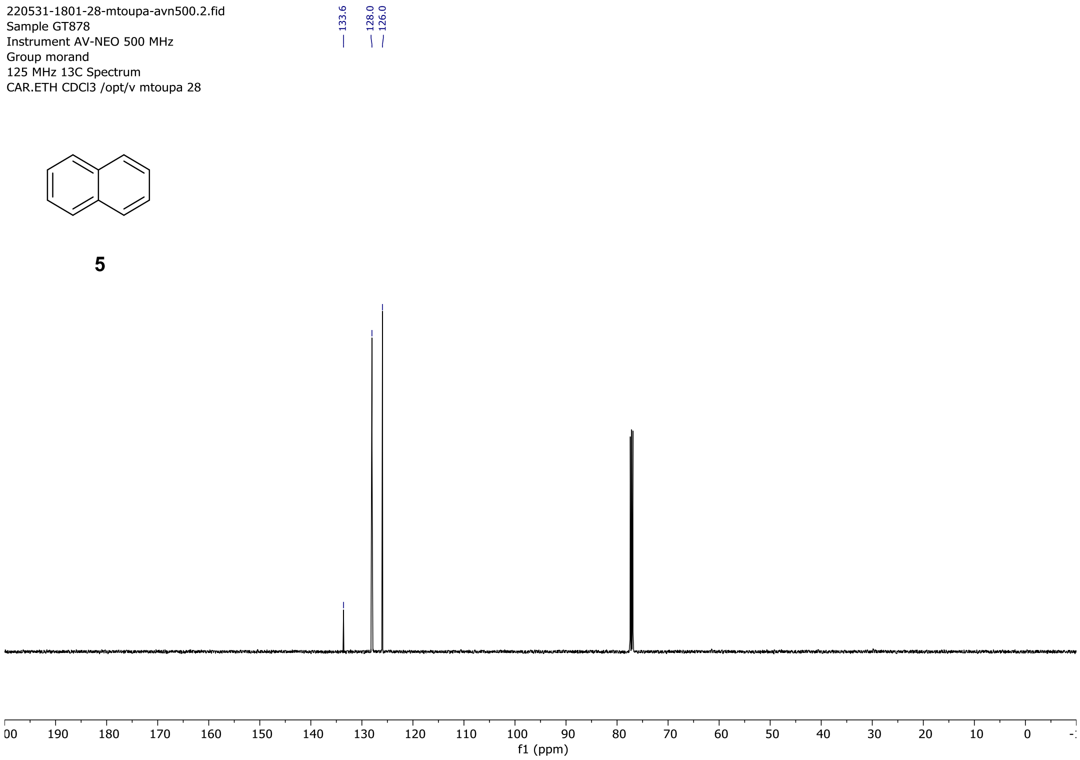

Supplementary Figure 109 ^{13}C NMR (125 MHz, $CDCl_3$, 25 °C) of compound **5.**

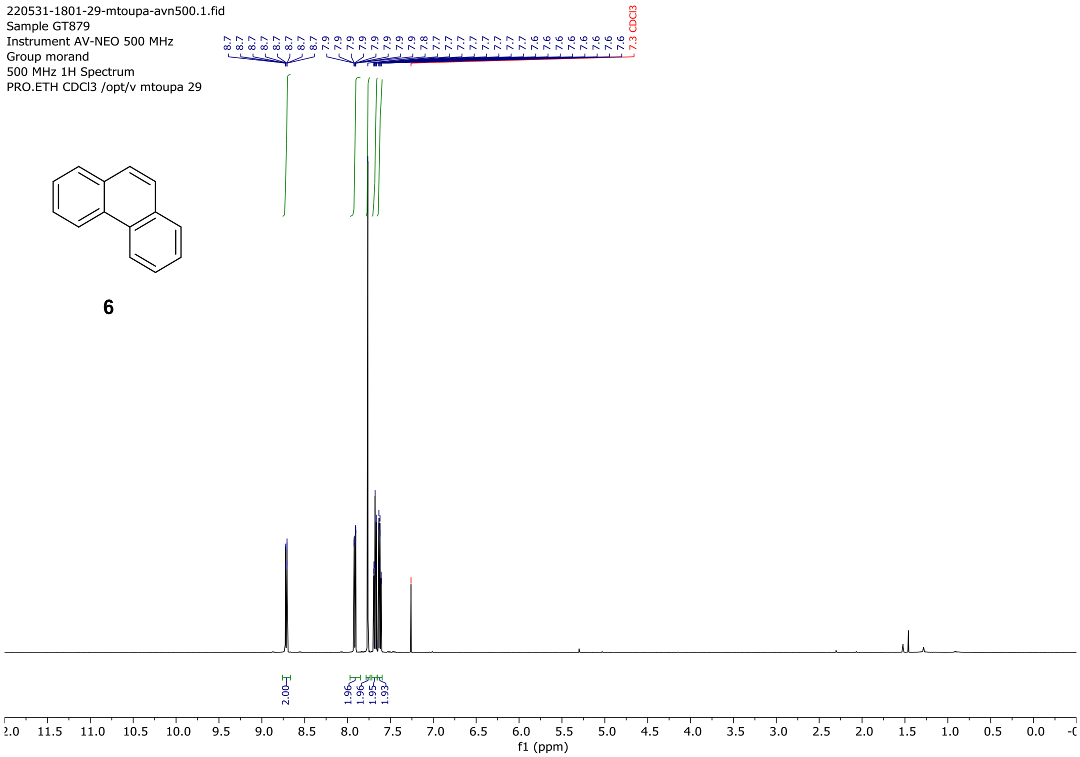

Supplementary Figure 110 ^{1}H NMR (400 MHz, $CDCl_3$, 25 °C) of compound **6.**

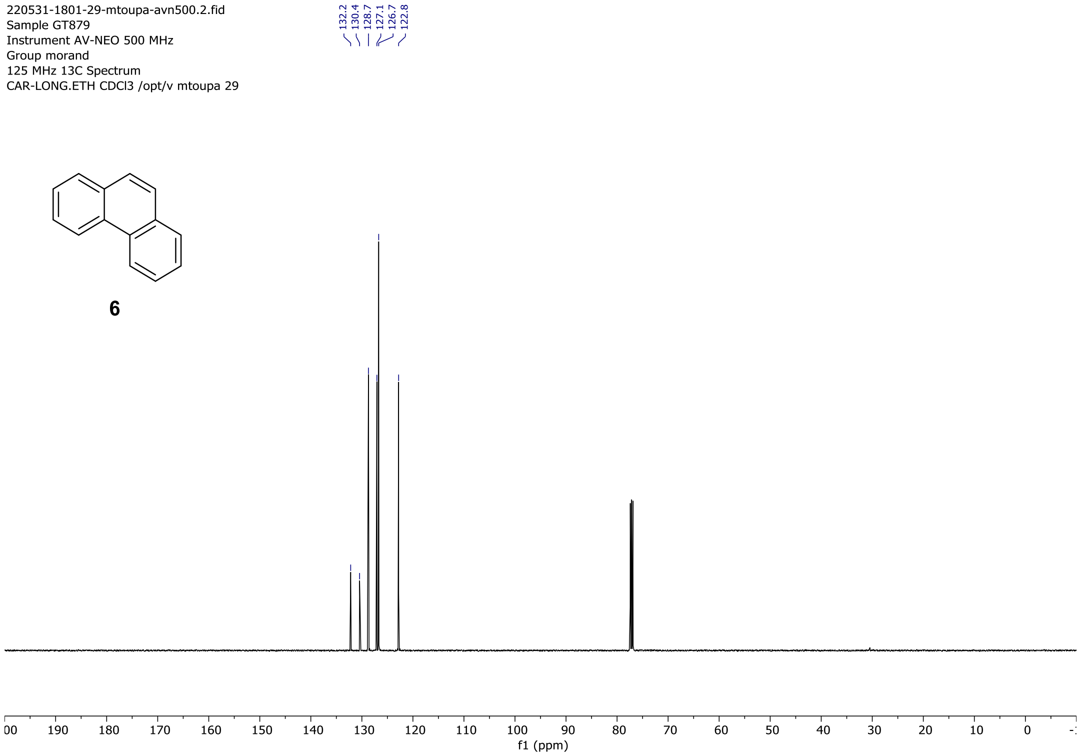

Supplementary Figure 111 ^{13}C NMR (100 MHz, $CDCl_3$, 25 °C) of compound **6.**

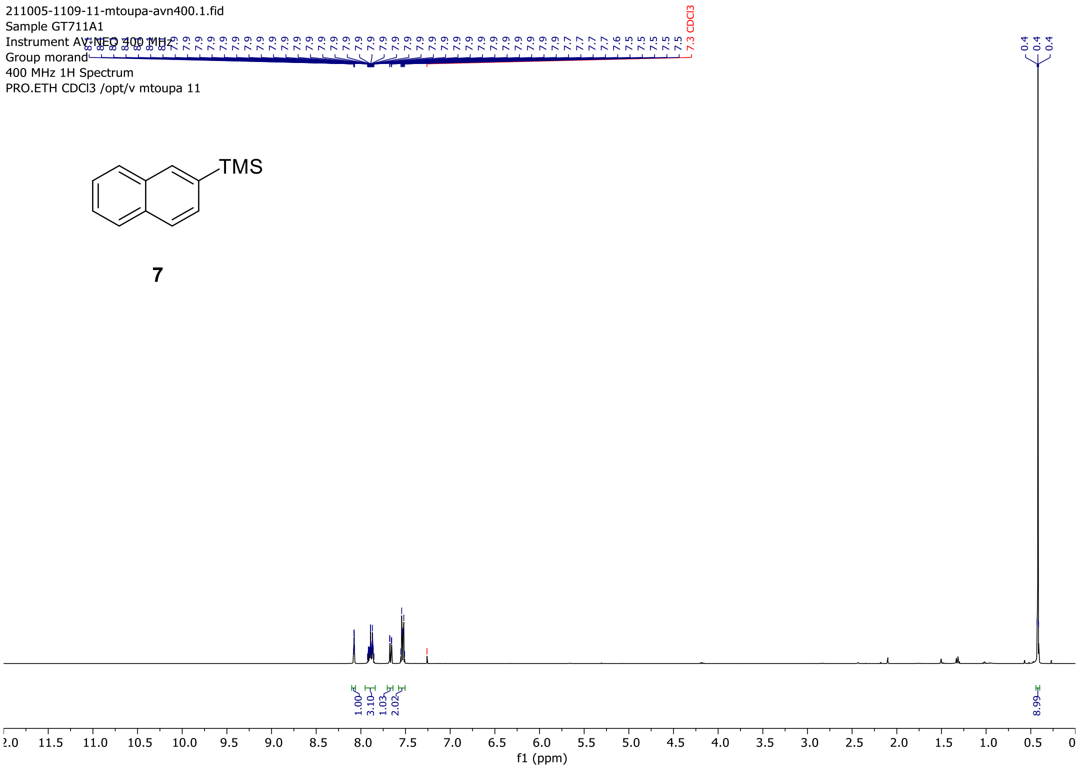

Supplementary Figure 112 ^{1}H NMR (400 MHz, $CDCl_3$, 25 °C) of compound **7.**

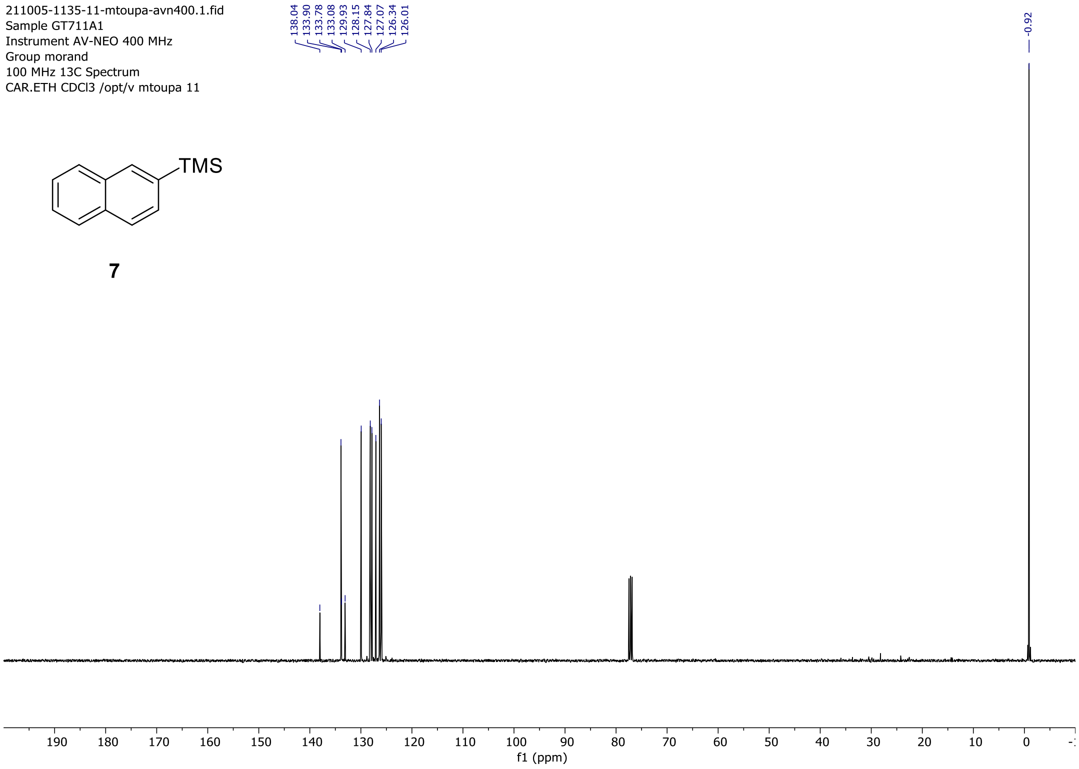

Supplementary Figure 113 ^{13}C NMR (100 MHz, $CDCl_3$, 25 °C) of compound **7.**

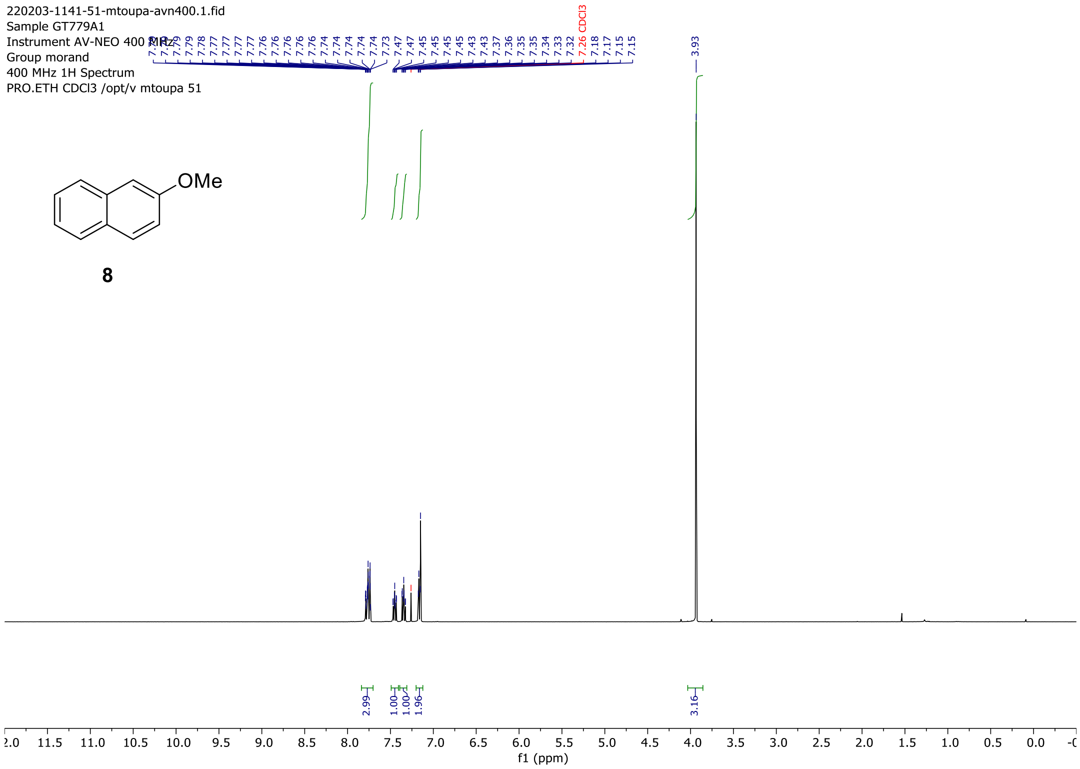

Supplementary Figure 114 ^{1}H NMR (400 MHz, $CDCl_3$, 25 °C) of compound **8.**

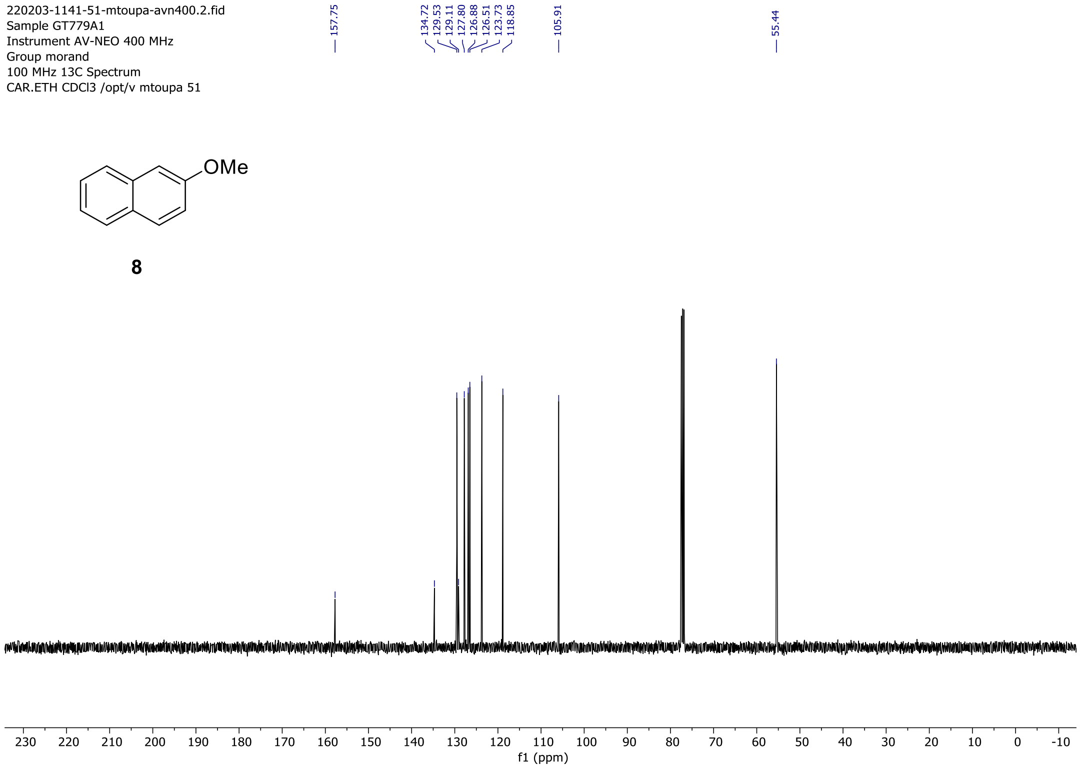

Supplementary Figure 115 ^{13}C NMR (100 MHz, $CDCl_3$, 25 °C) of compound **8.**

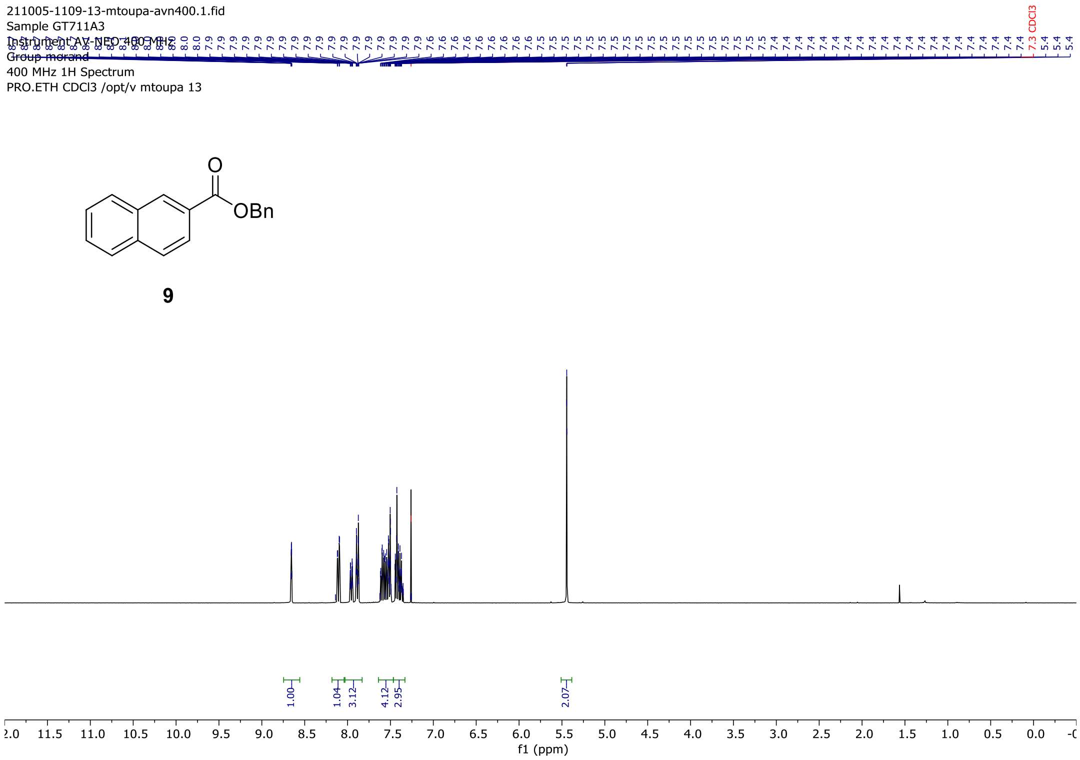

Supplementary Figure 116 ^{1}H NMR (400 MHz, $CDCl_3$, 25 °C) of compound **9.**

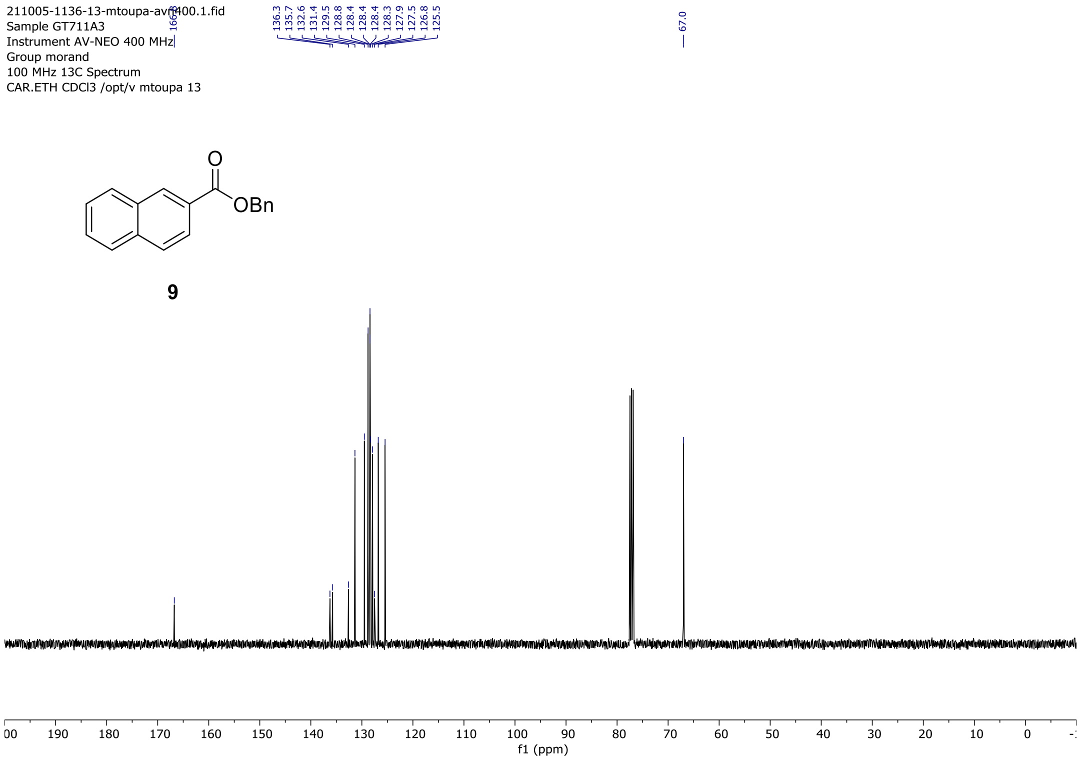

Supplementary Figure 117 ^{13}C NMR (100 MHz, $CDCl_3$, 25 °C) of compound **9.**

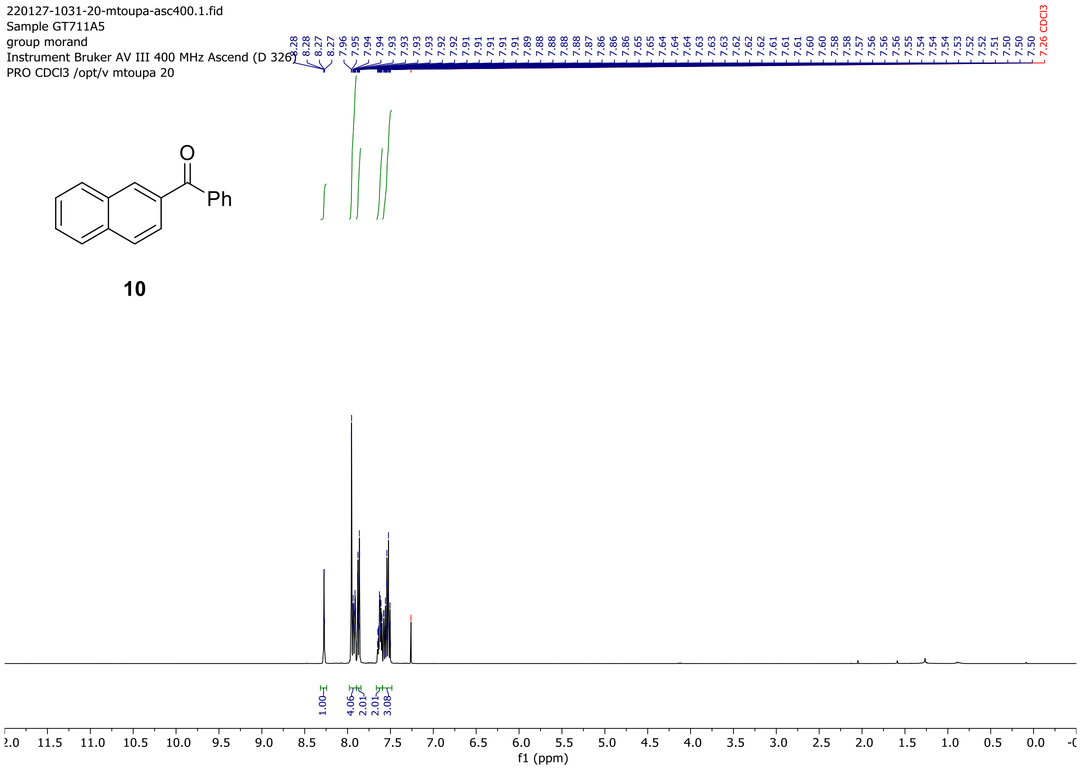

Supplementary Figure 118 ^{1}H NMR (400 MHz, $CDCl_3$, 25 °C) of compound **10.**

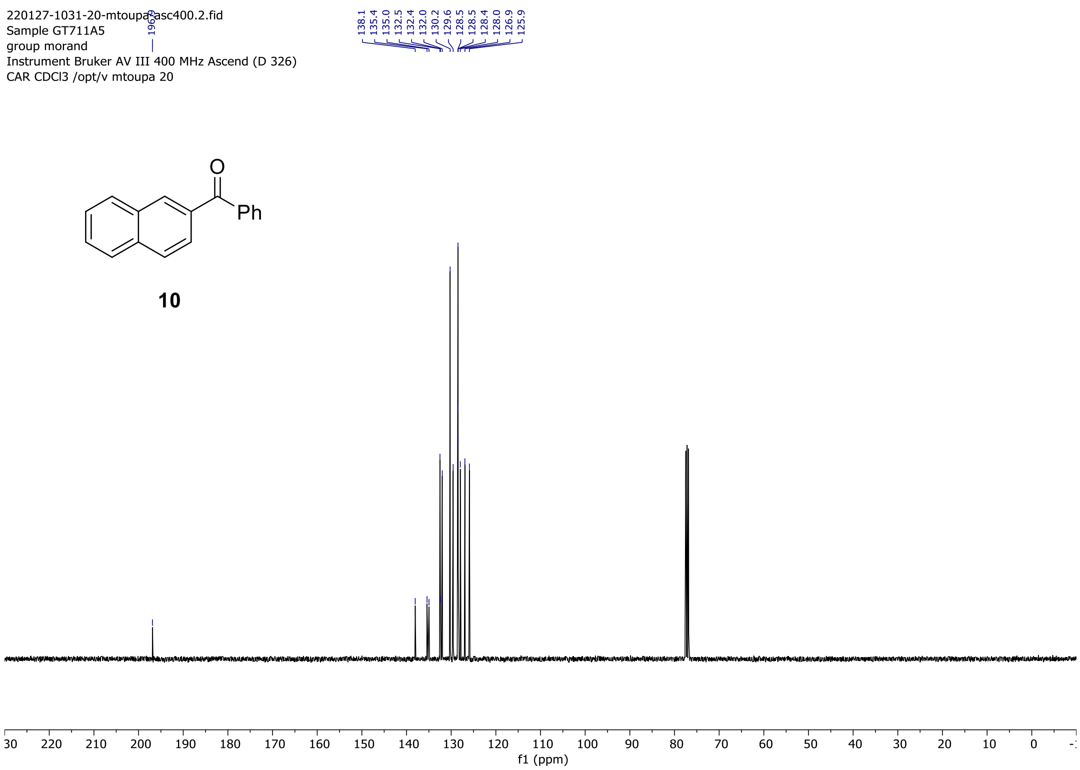

Supplementary Figure 119 ^{13}C NMR (100 MHz, $CDCl_3$, 25 °C) of compound **10.**

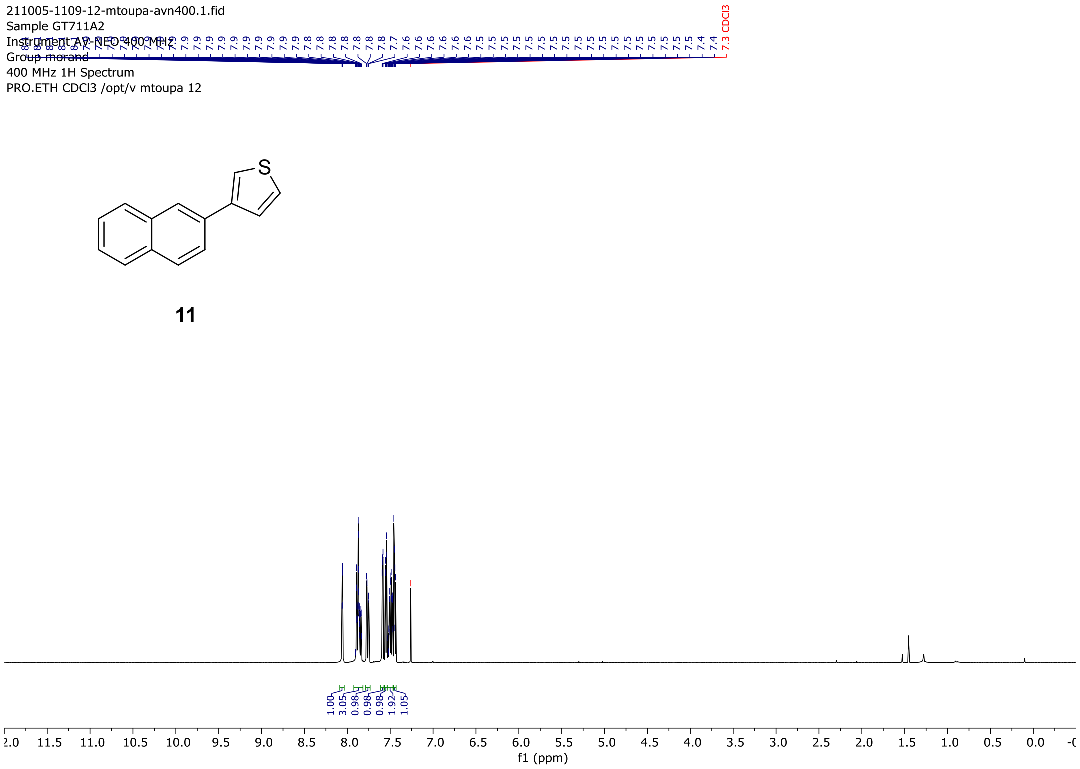

Supplementary Figure 120 ^{1}H NMR (400 MHz, $CDCl_3$, 25 °C) of compound **11.**

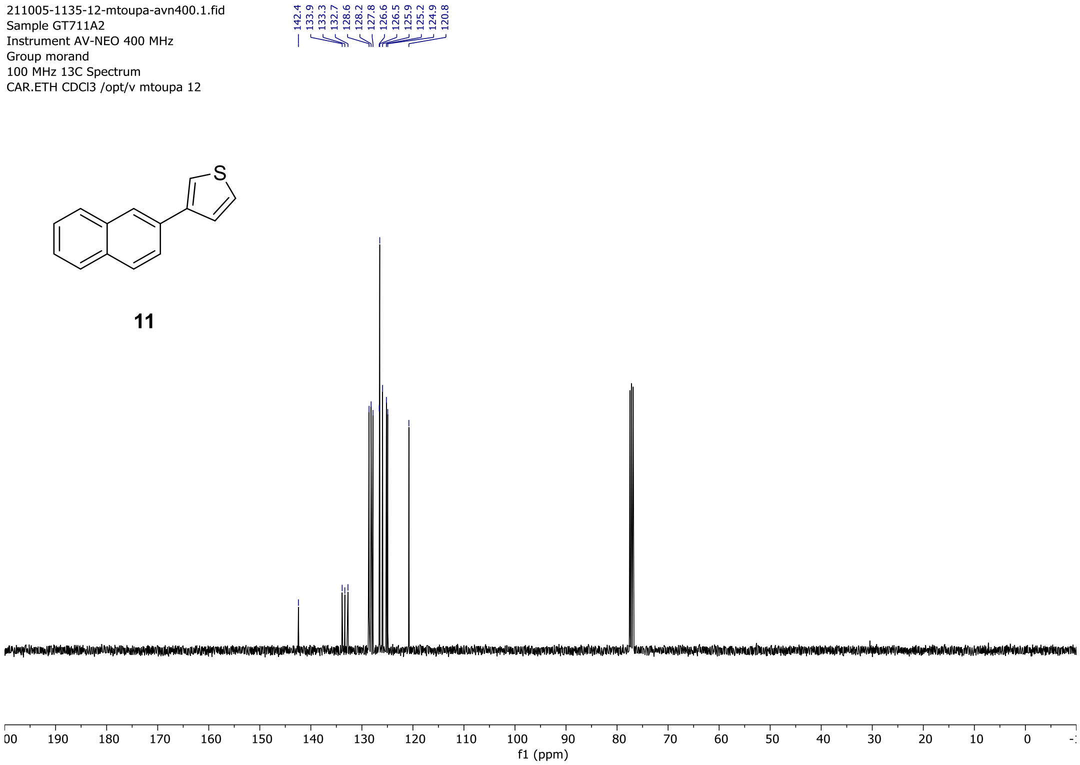

Supplementary Figure 121 ^{13}C NMR (100 MHz, $CDCl_3$, 25 °C) of compound **11.**

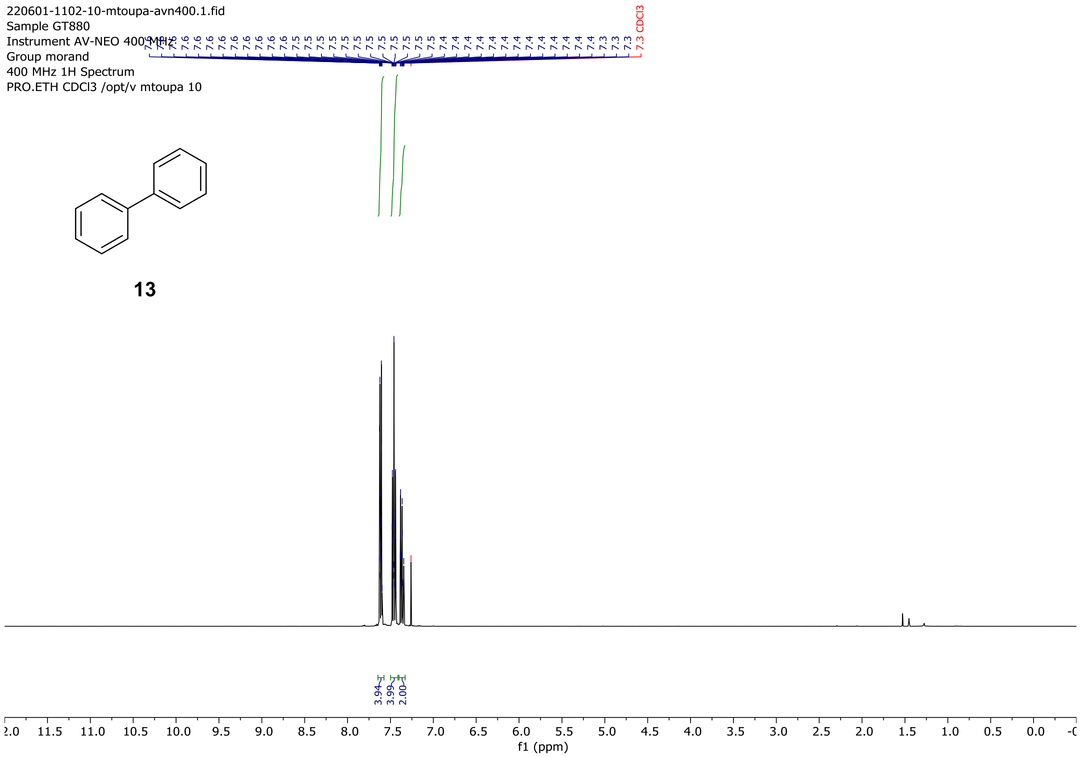

Supplementary Figure 122 ^{1}H NMR (400 MHz, $CDCl_3$, 25 °C) of compound **13.**

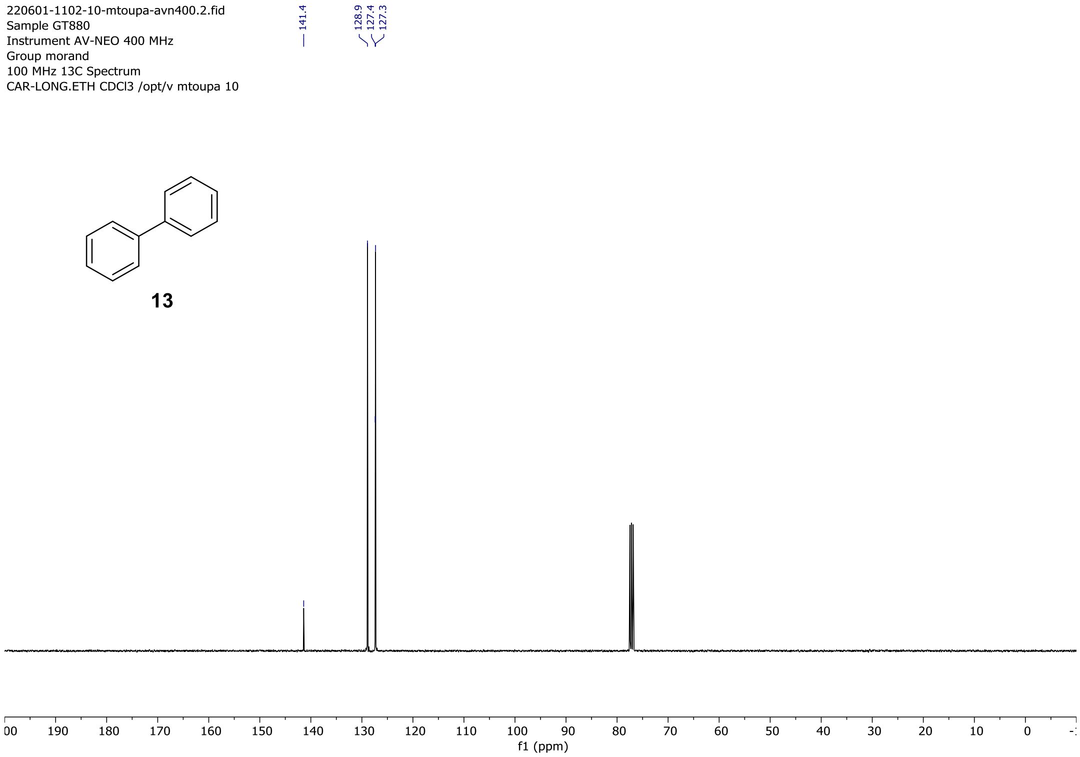

Supplementary Figure 123 ^{13}C NMR (100 MHz, $CDCl_3$, 25 °C) of compound **13.**

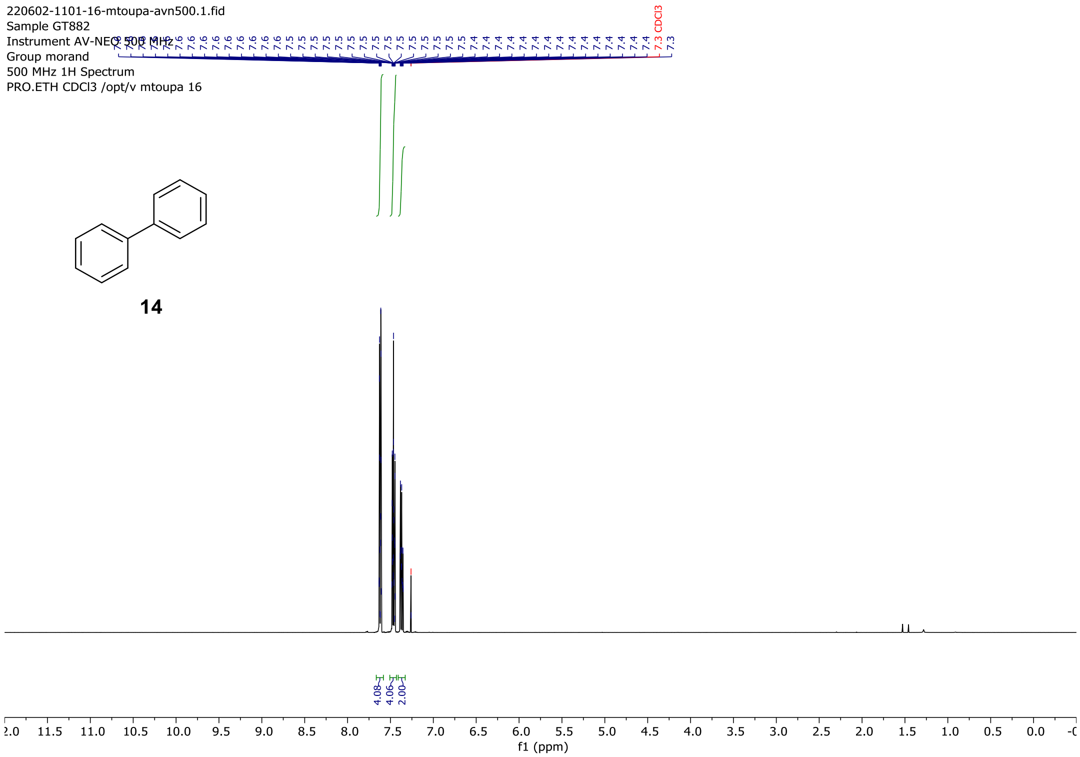

Supplementary Figure 124 ^{1}H NMR (500 MHz, $CDCl_3$, 25 °C) of compound **14.**

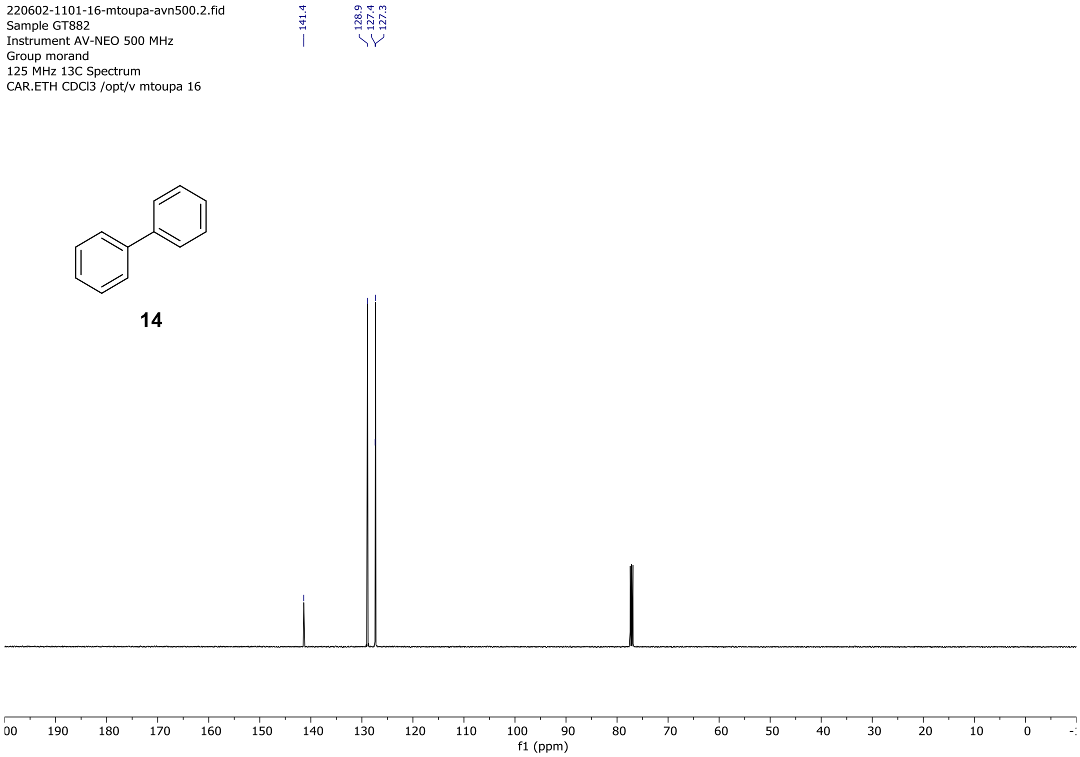

Supplementary Figure 125 ^{13}C NMR (125 MHz, $CDCl_3$, 25 °C) of compound **14.**

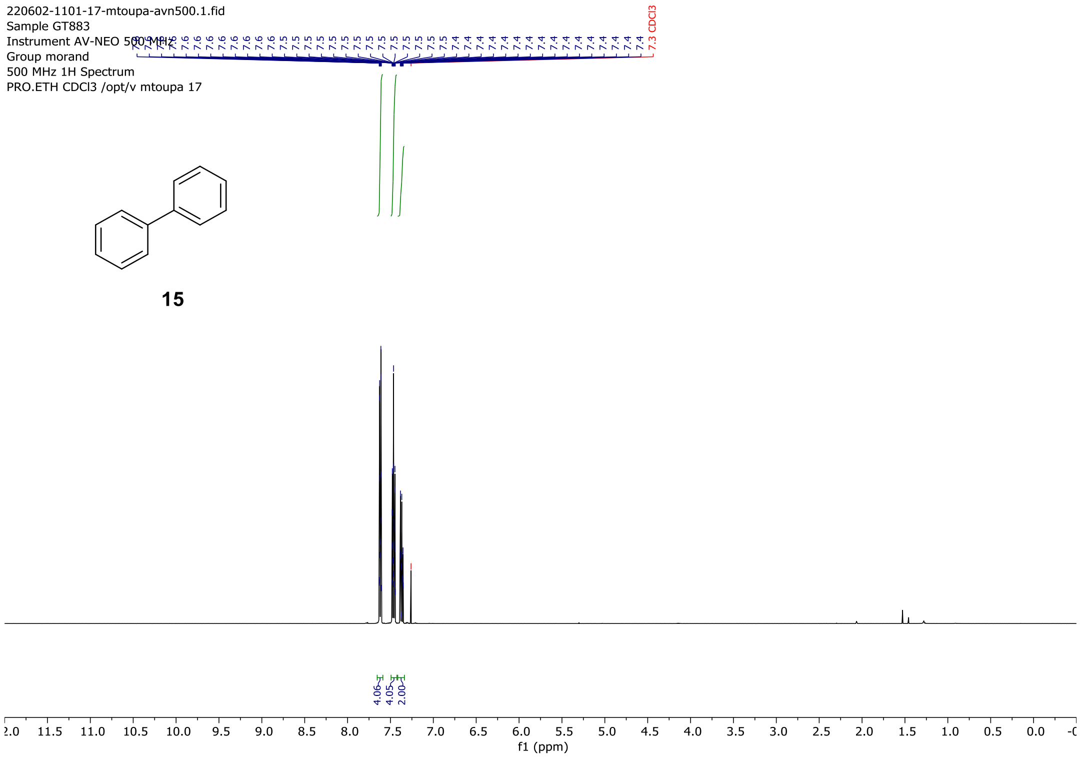

Supplementary Figure 126 ^{1}H NMR (500 MHz, $CDCl_3$, 25 °C) of compound **15.**

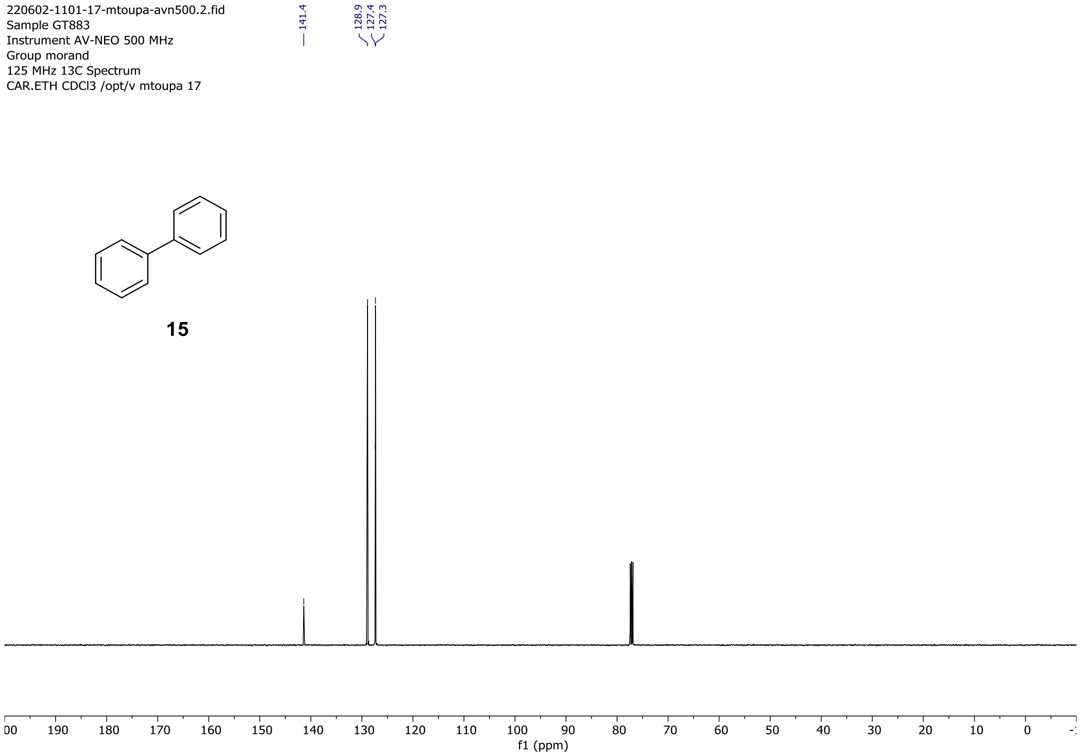

Supplementary Figure 127 ^{13}C NMR (125 MHz, $CDCl_3$, 25 °C) of compound **15.**

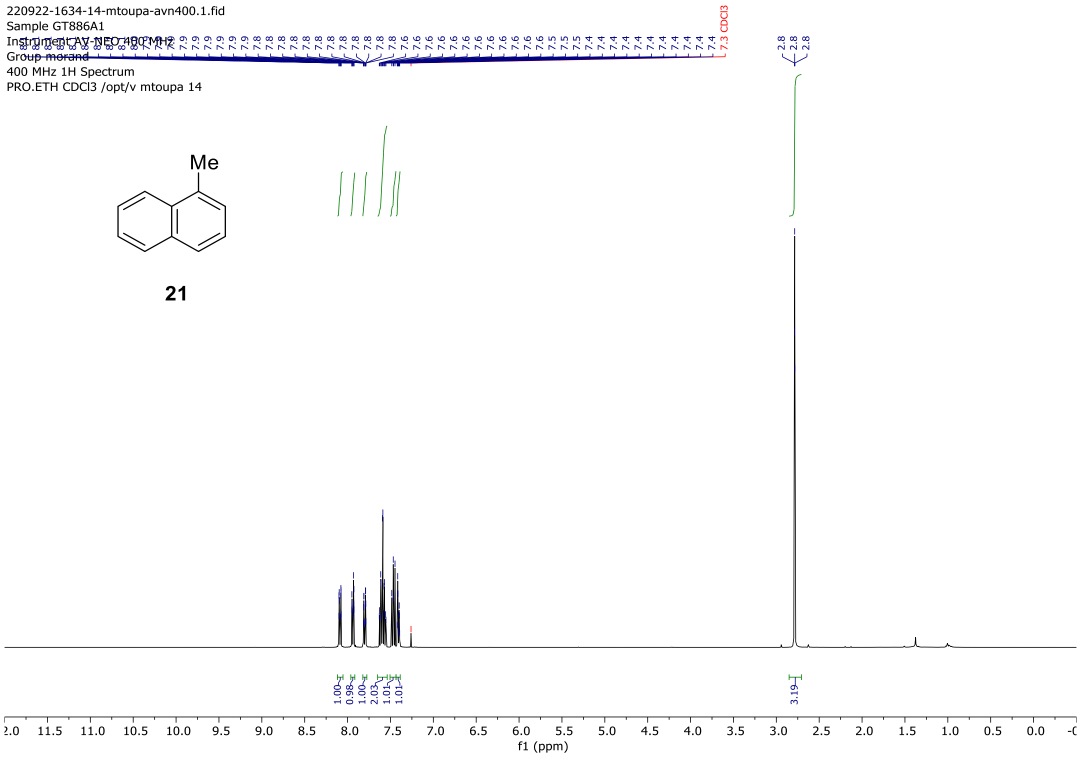

Supplementary Figure 128 ^{1}H NMR (400 MHz, $CDCl_3$, 25 °C) of compound **21.**

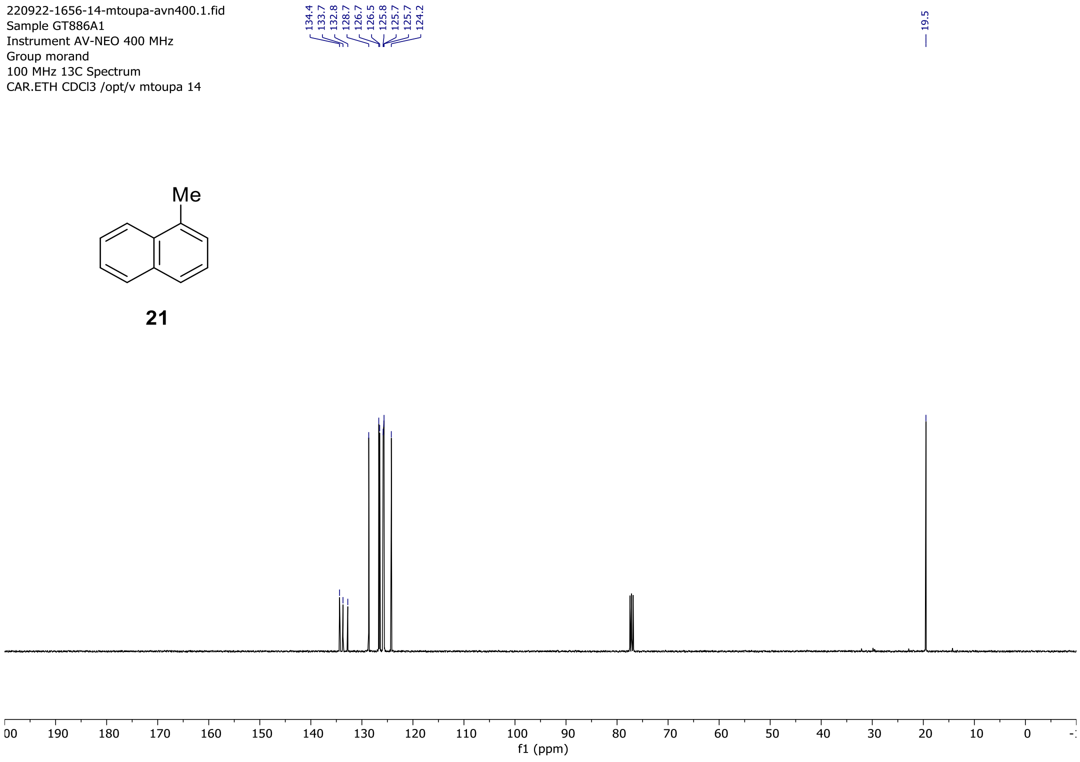

Supplementary Figure 129 ^{13}C NMR (100 MHz, $CDCl_3$, 25 °C) of compound **21.**

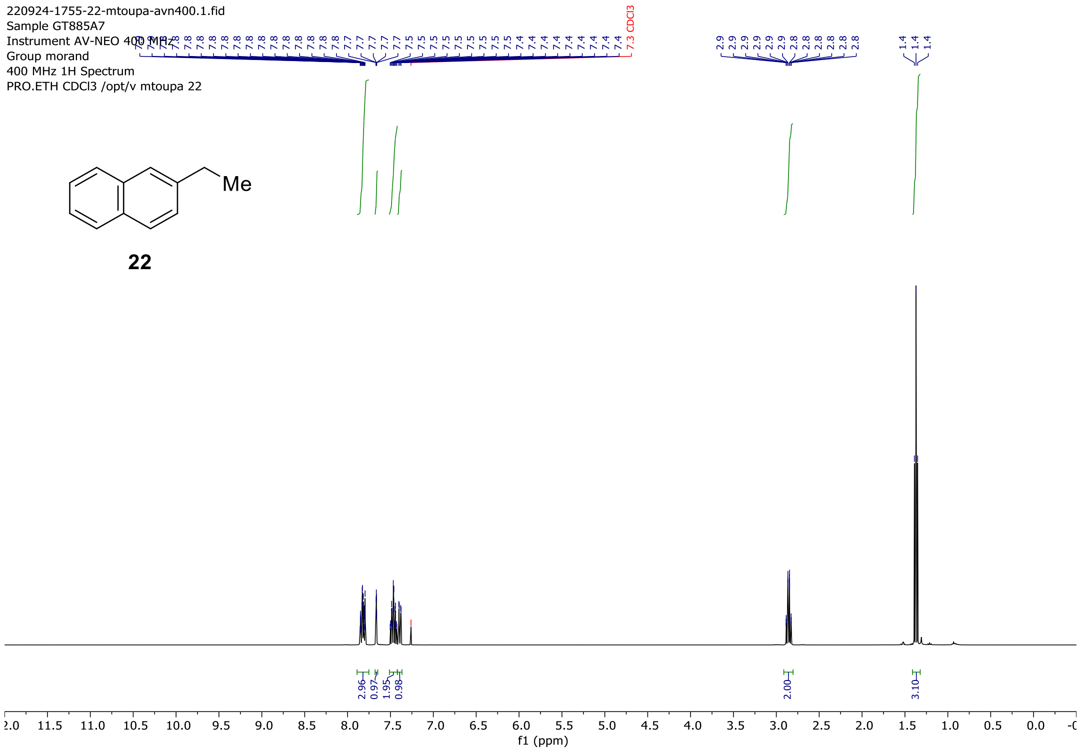

Supplementary Figure 130 ^{1}H NMR (400 MHz, $CDCl_3$, 25 °C) of compound **22.**

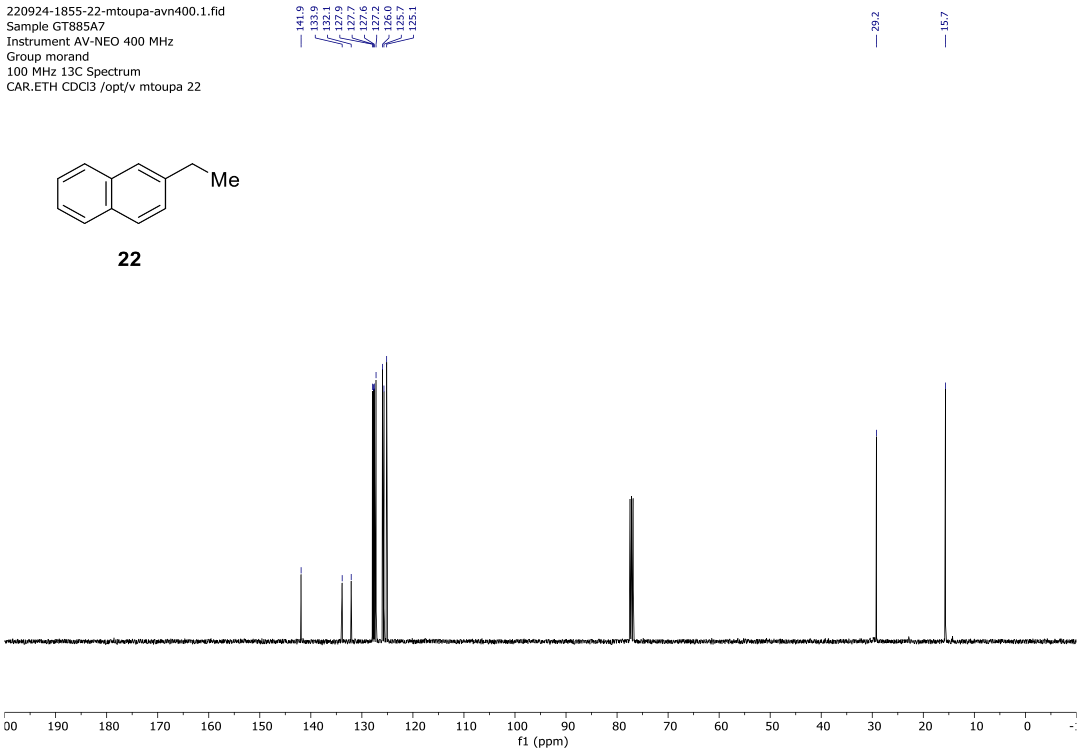

Supplementary Figure 131 ^{13}C NMR (100 MHz, $CDCl_3$, 25 °C) of compound **22.**

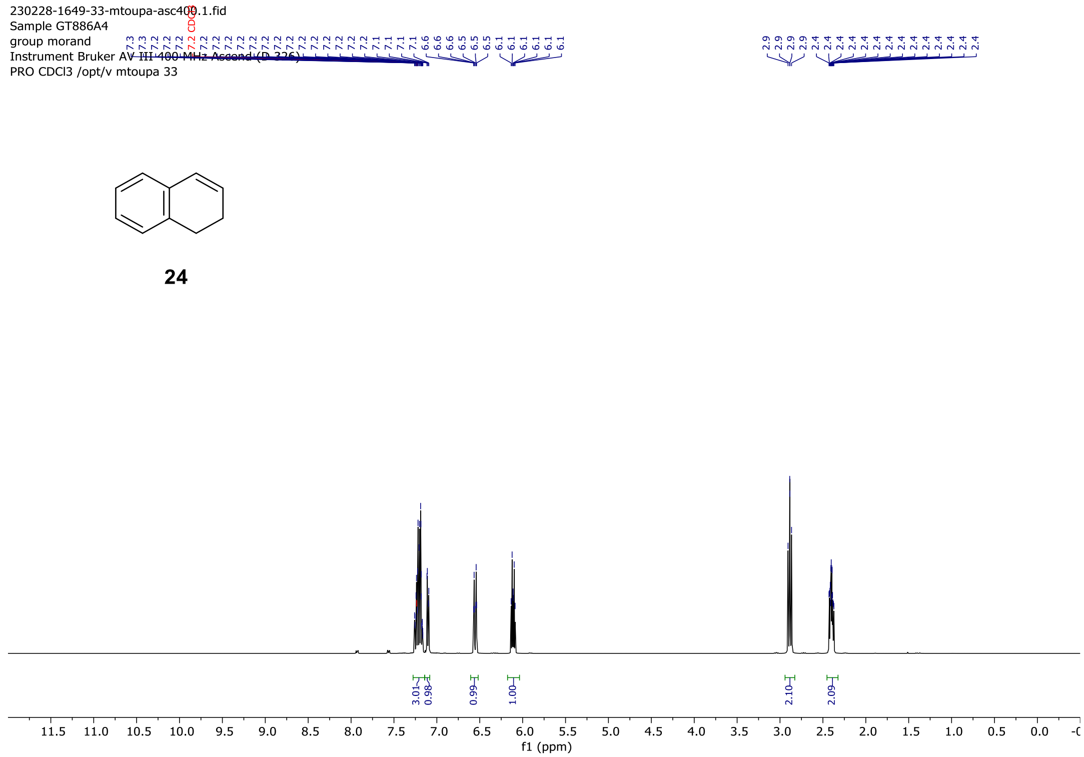

Supplementary Figure 132 ^{1}H NMR (400 MHz, $CDCl_3$, 25 °C) of compound **24.**

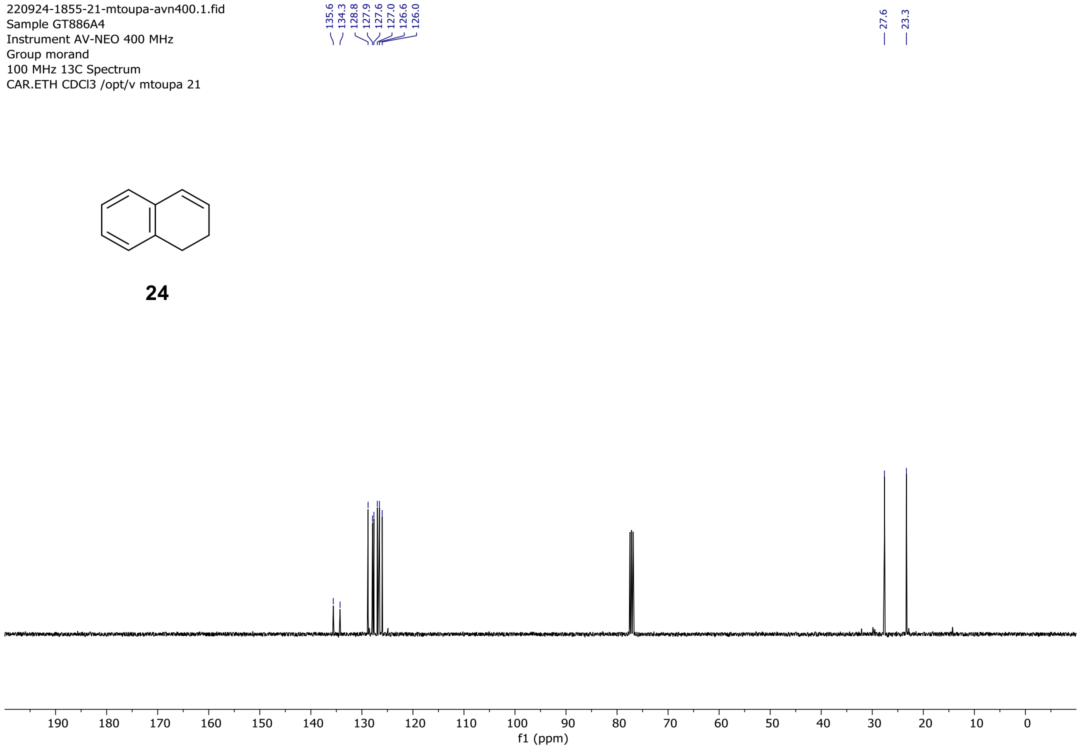

Supplementary Figure 133 ^{13}C NMR (100 MHz, $CDCl_3$, 25 °C) of compound **24.**

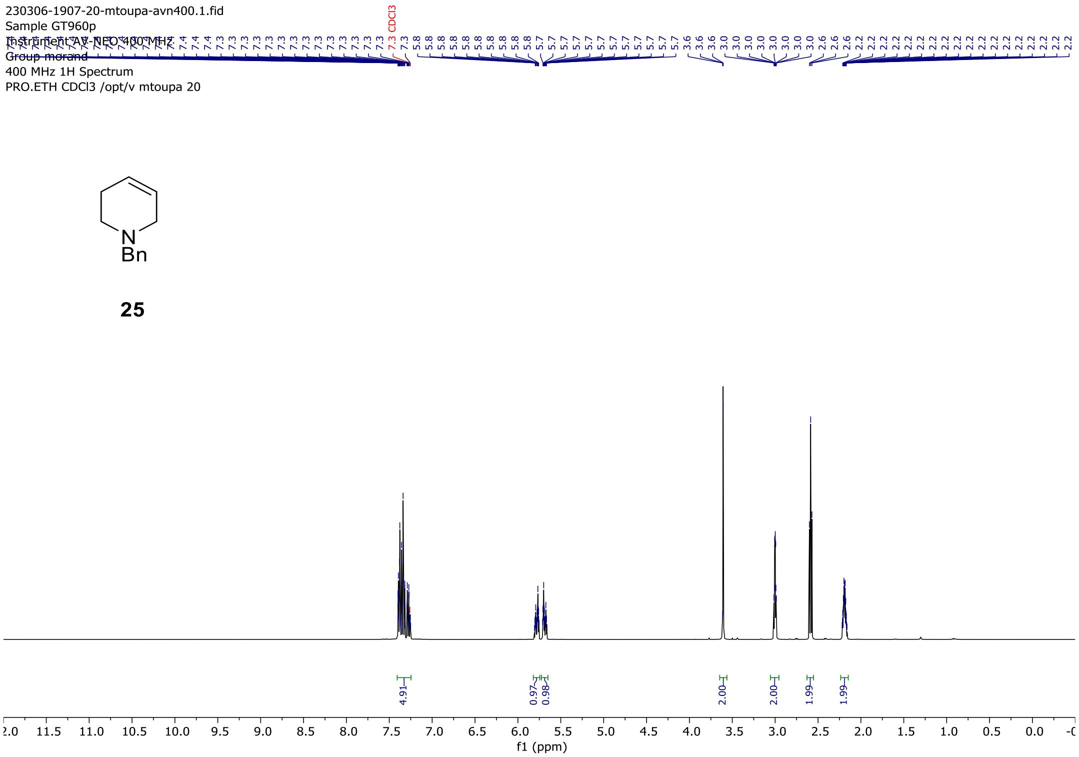

Supplementary Figure 134 ^{1}H NMR (400 MHz, $CDCl_3$, 25 °C) of compound **25.**

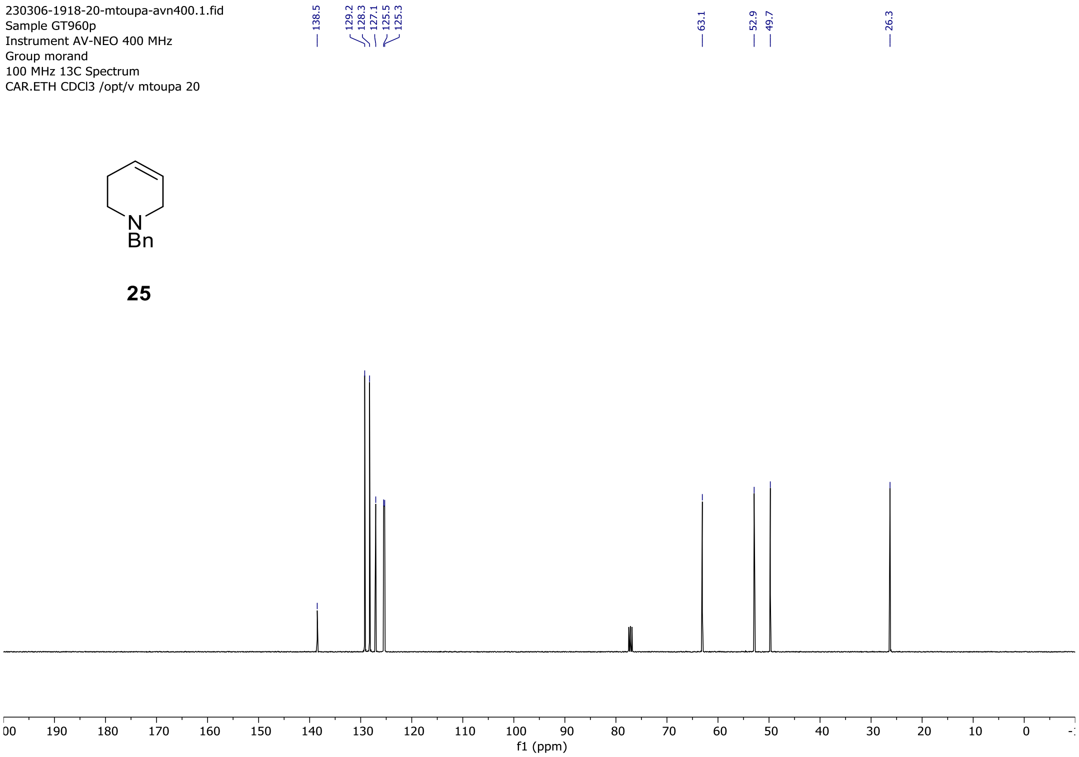

Supplementary Figure 135 ^{13}C NMR (100 MHz, $CDCl_3$, 25 °C) of compound **25.**

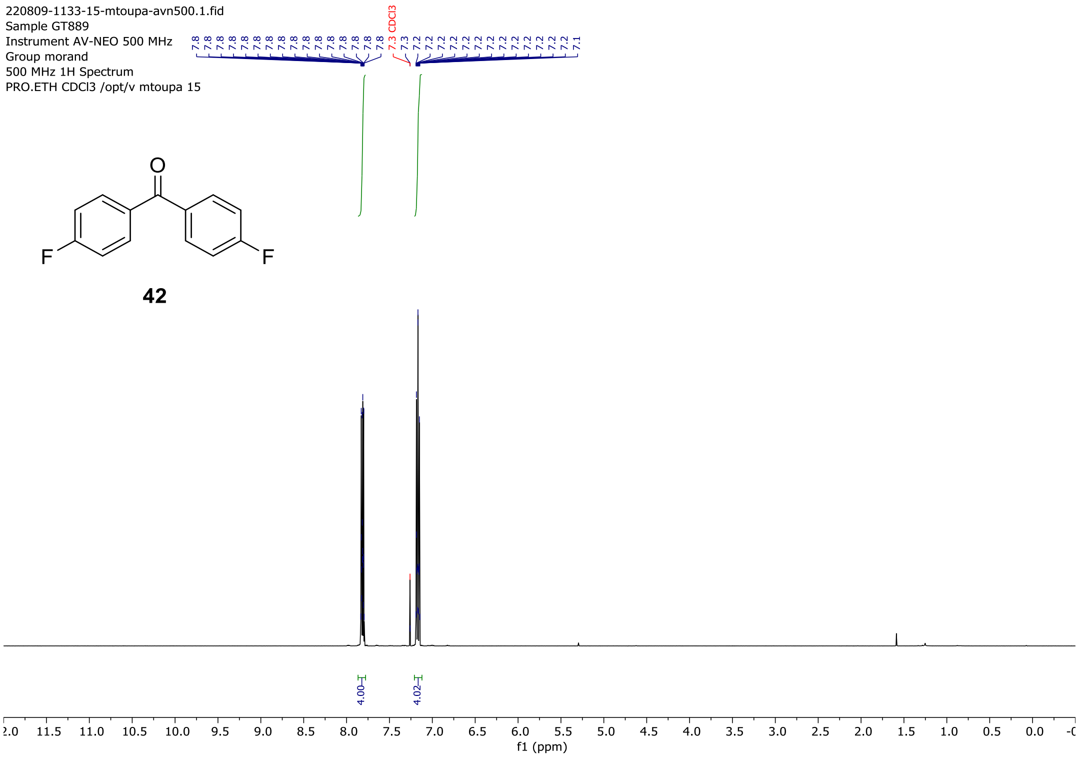

Supplementary Figure 136 ^{1}H NMR (500 MHz, $CDCl_3$, 25 °C) of compound **42.**

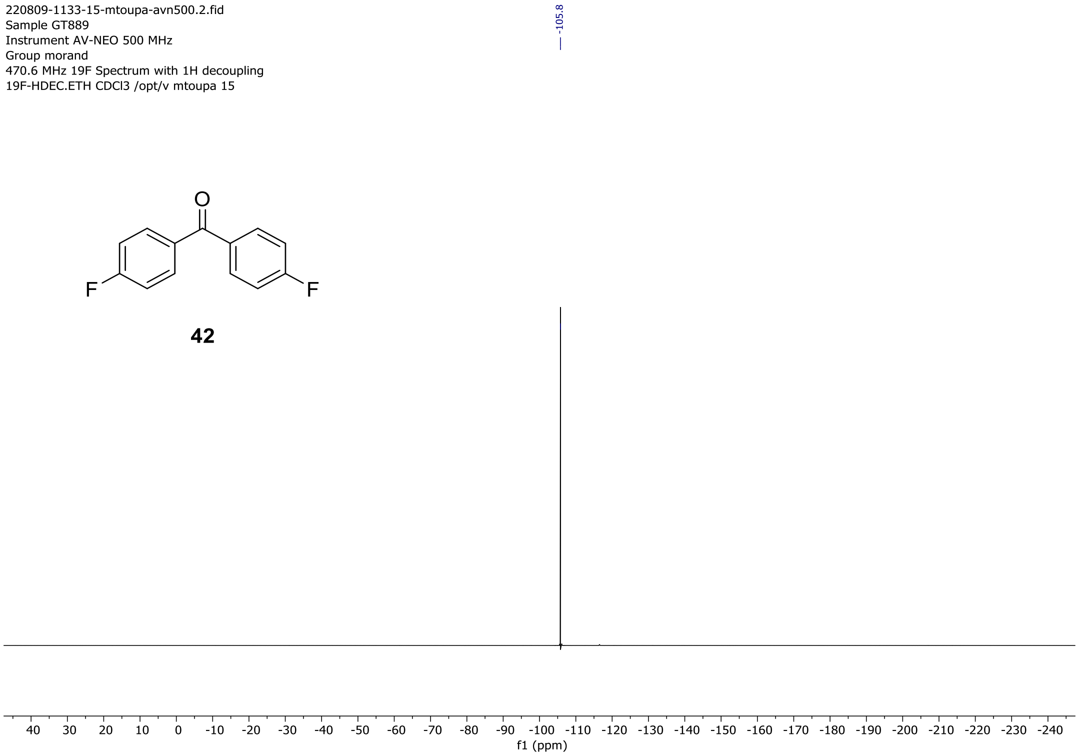

Supplementary Figure 137 ^{19}F NMR (471 MHz, $CDCl_3$, 25 °C) of compound **42.**

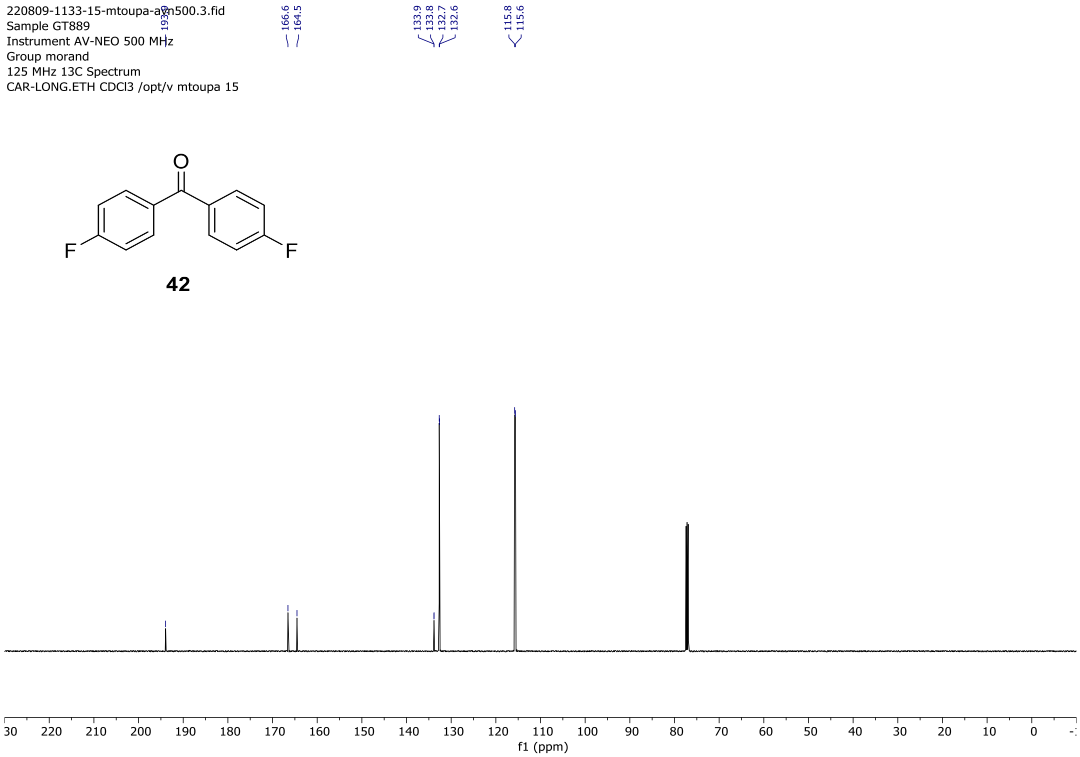

Supplementary Figure 138 ^{13}C NMR (125 MHz, $CDCl_3$, 25 °C) of compound **42.**

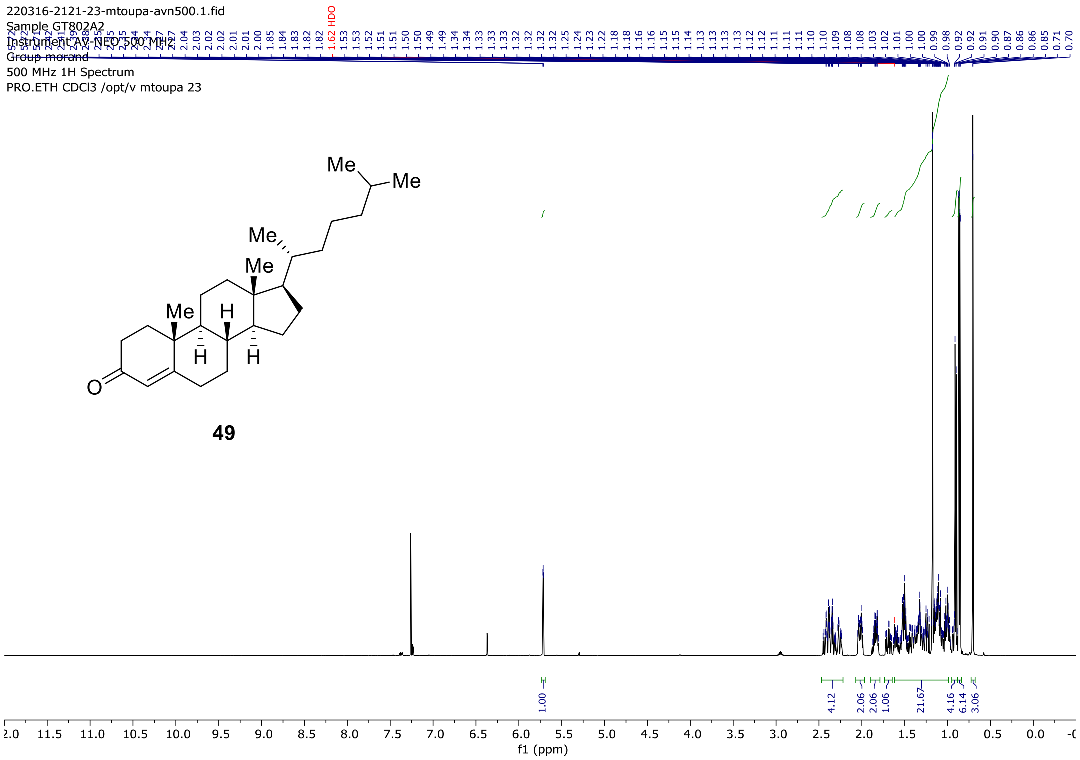

Supplementary Figure 139 ^{1}H NMR (500 MHz, $CDCl_3$, 25 °C) of compound **49.**

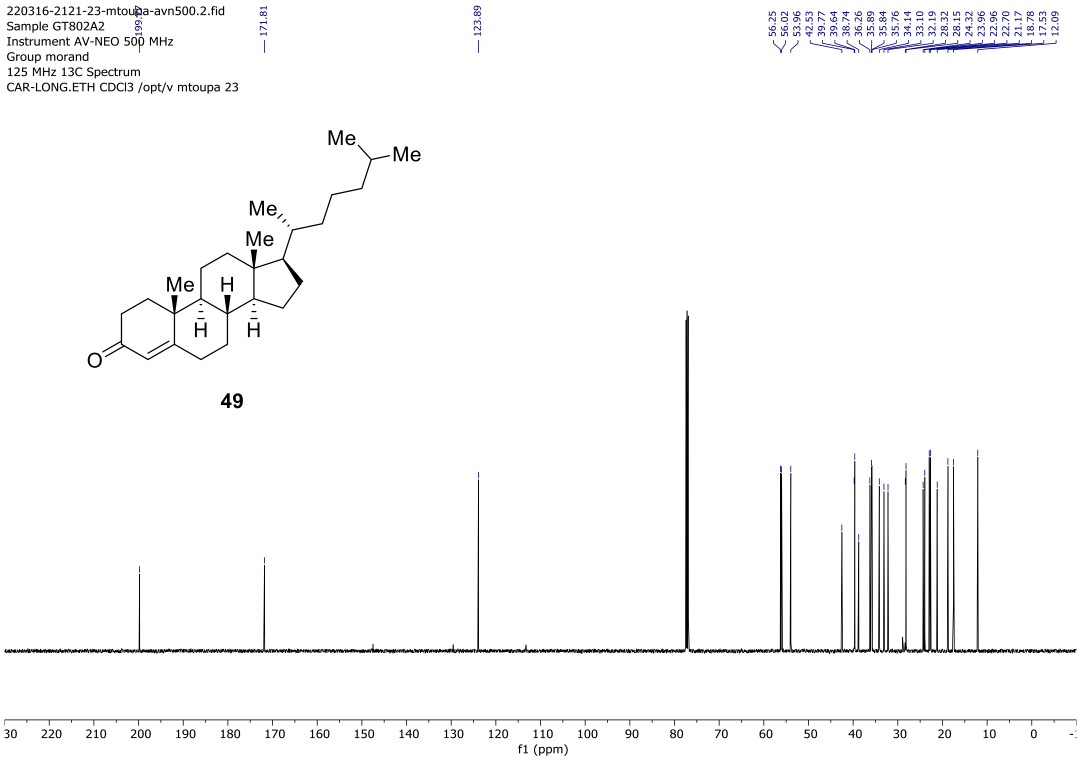

Supplementary Figure 140 ^{13}C NMR (125 MHz, $CDCl_3$, 25 °C) of compound **49.**

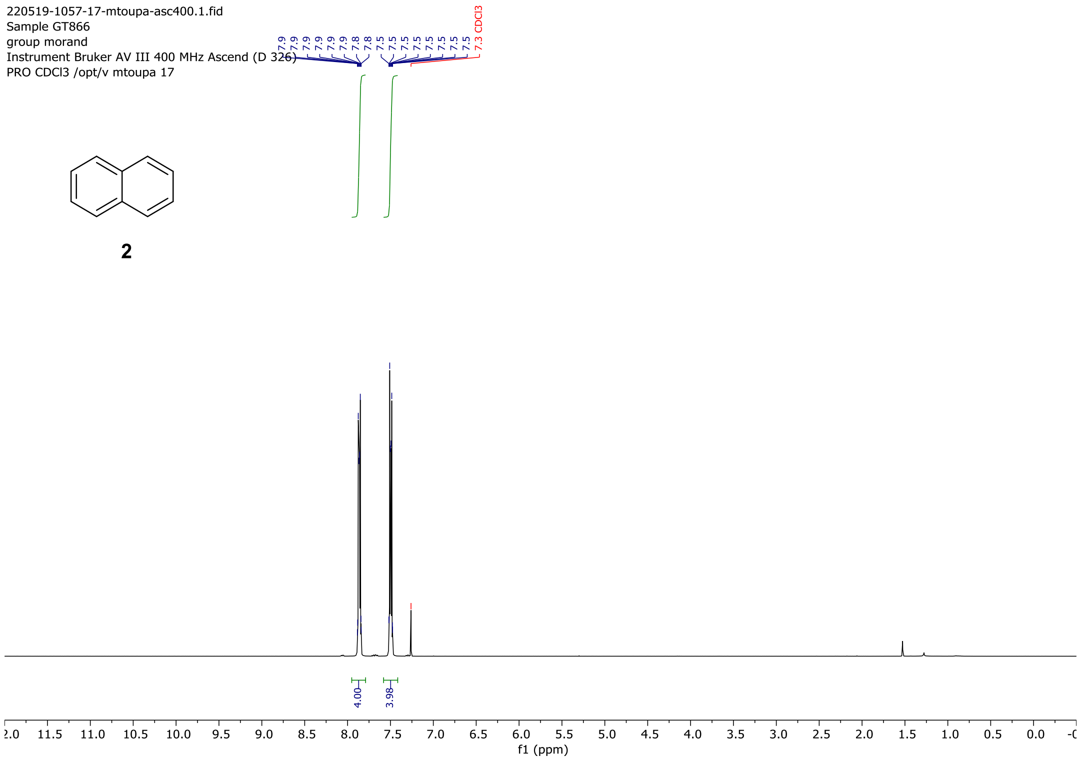

Supplementary Figure 141 ^{1}H NMR (500 MHz, $CDCl_3$, 25 °C) of compound **2** from the reaction with THF-d_8**.**

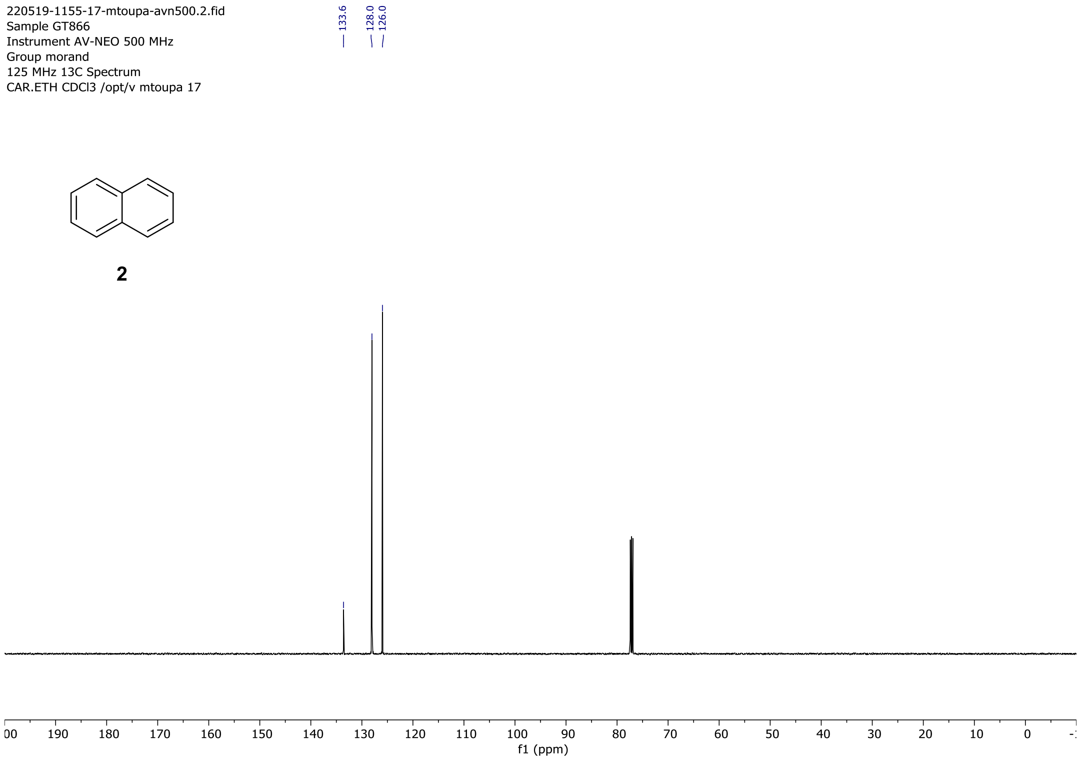

Supplementary Figure 142 ^{13}C NMR (125 MHz, $CDCl_3$, 25 °C) of compound **2** from the reaction with THF-d_8**.**

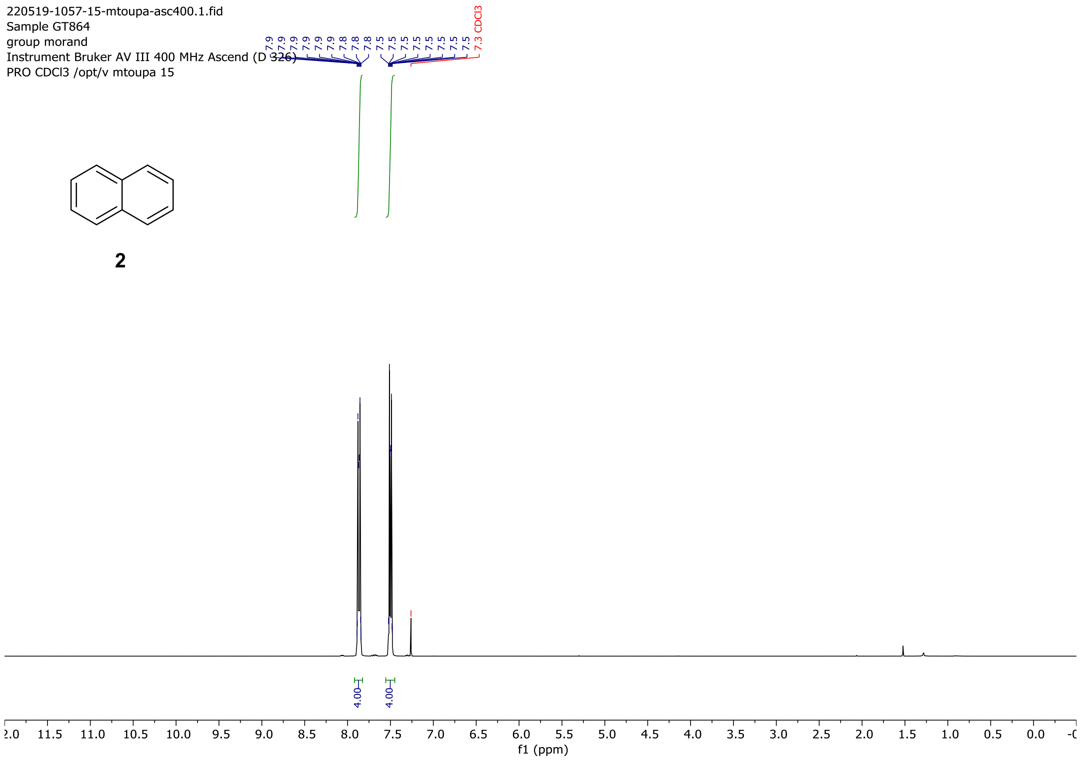

Supplementary Figure 143 ^{1}H NMR (500 MHz, $CDCl_3$, 25 °C) of compound **2** from the reaction with carbonate **3.**

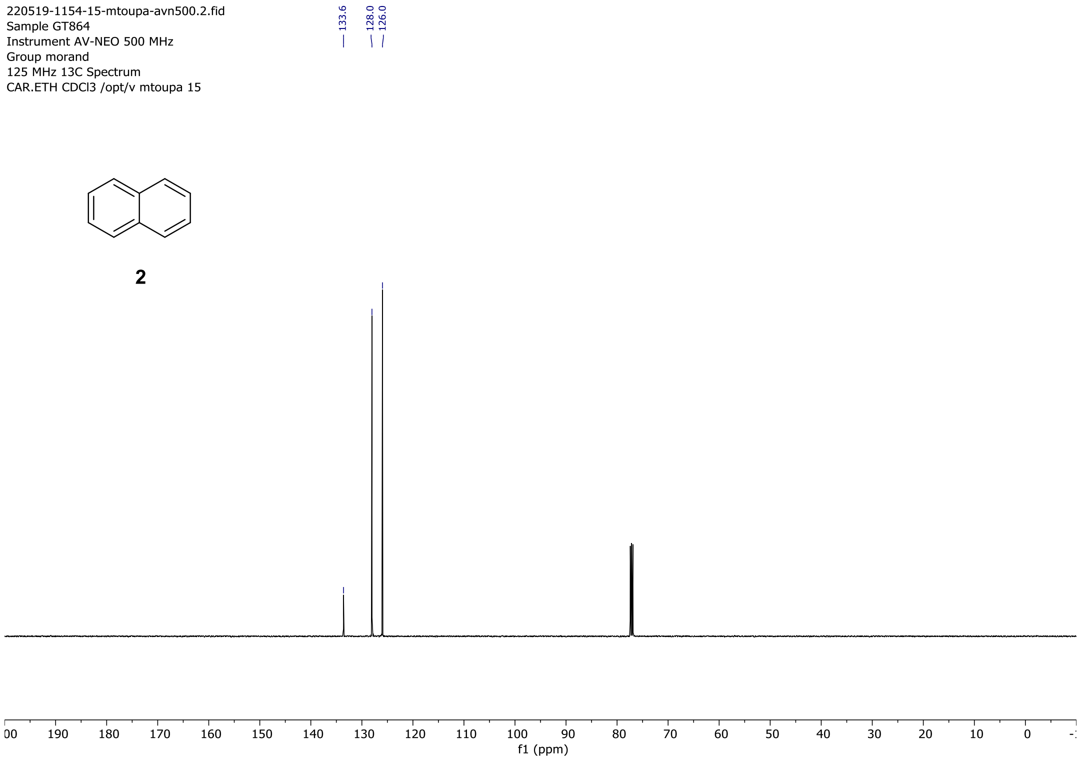

Supplementary Figure 144 ^{13}C NMR (125 MHz, $CDCl_3$, 25 °C) of compound **2** from the reaction with carbonate **3.**

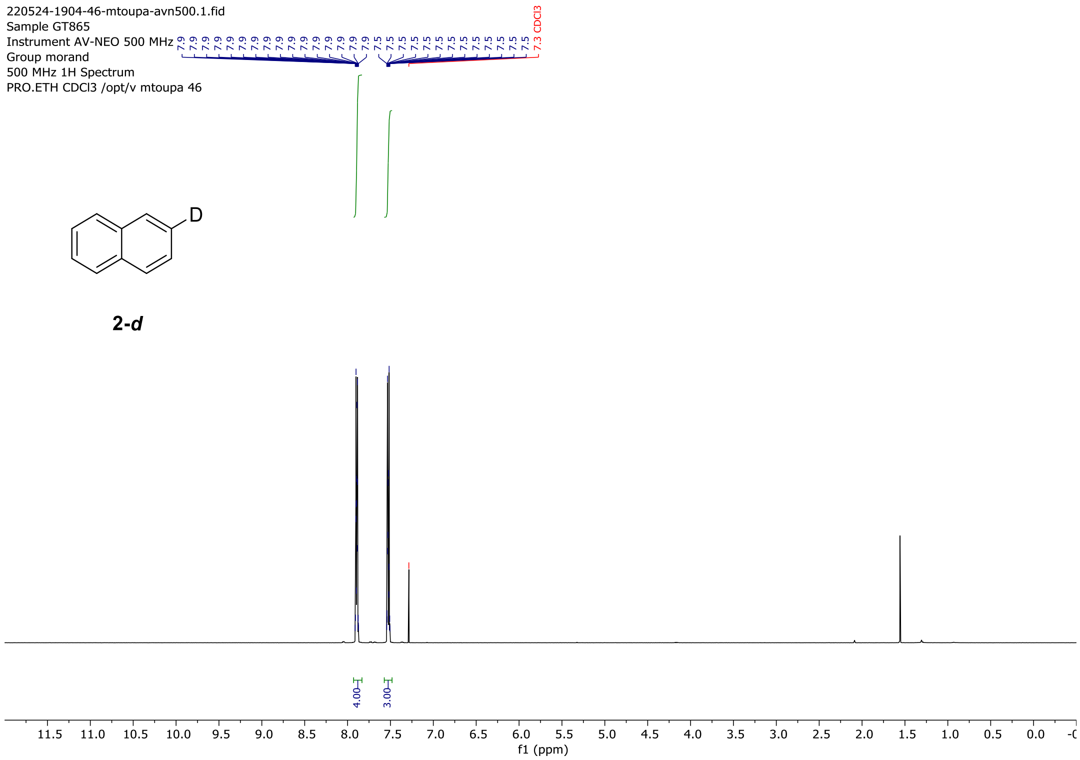

Supplementary Figure 145 ^{1}H NMR (500 MHz, $CDCl_3$, 25 °C) of compound **2-d** from the reaction with carbonate **4.**

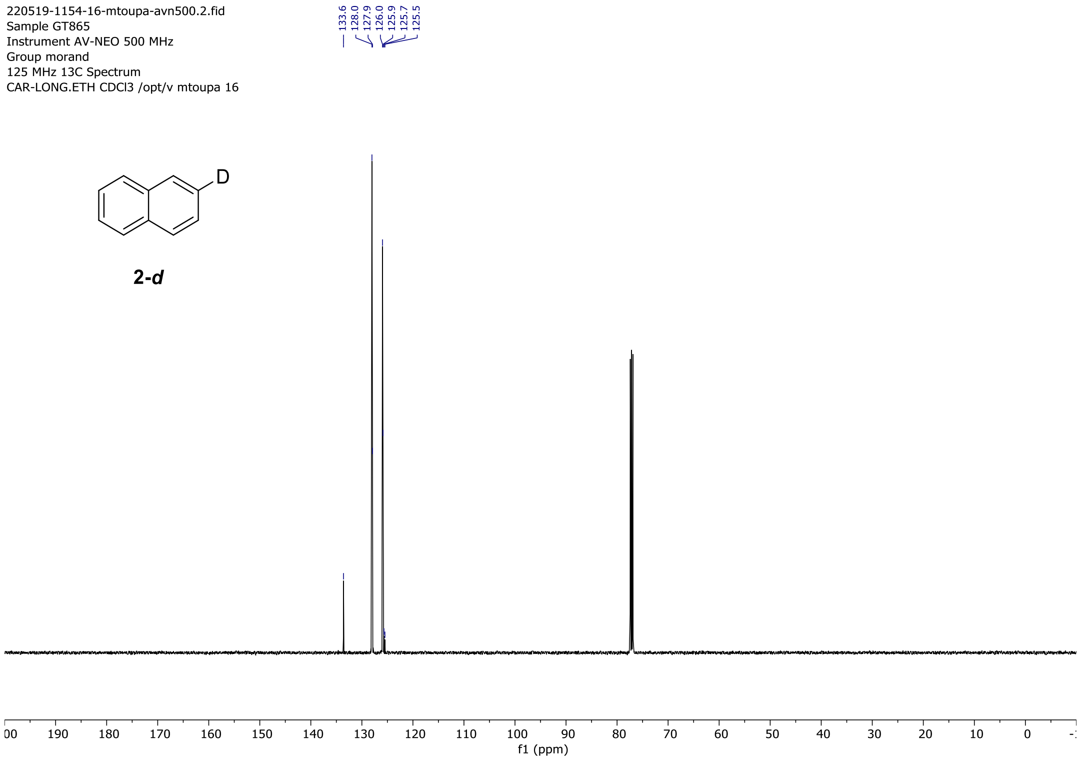

Supplementary Figure 146 ^{13}C NMR (125 MHz, $CDCl_3$, 25 °C) of compound **2-d** from the reaction with carbonate **4.**

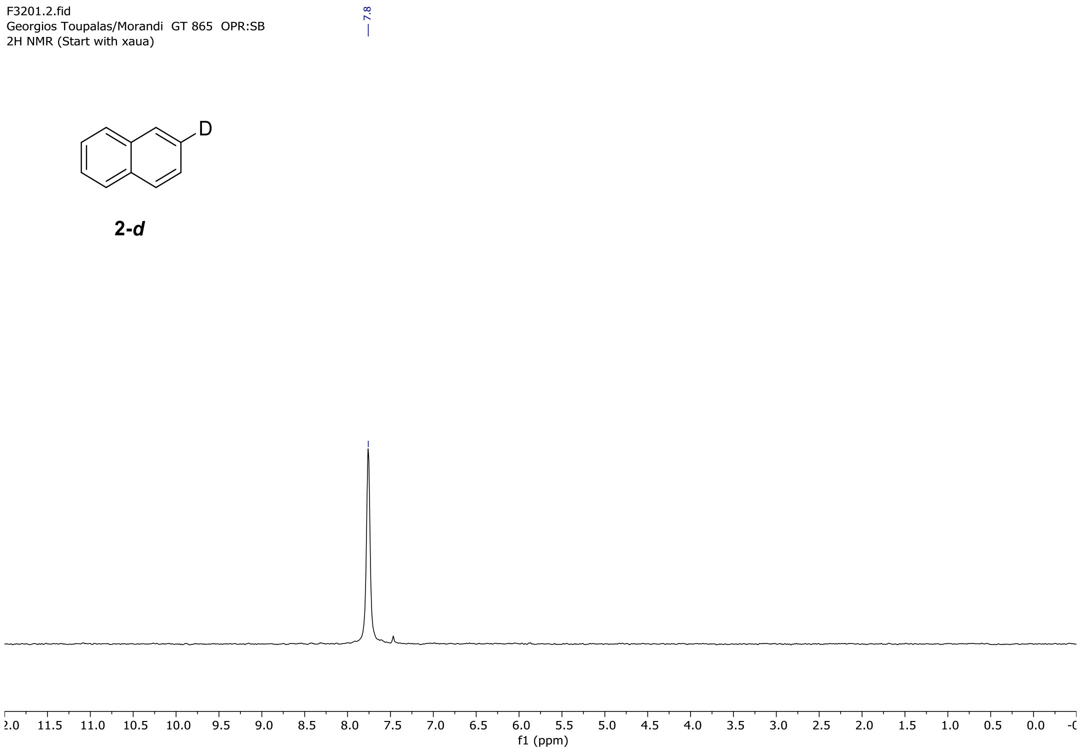

Supplementary Figure 147 ^{2}H NMR (77 MHz, $CDCl_3$, 25 °C) of compound **2-d** from the reaction with carbonate **4.**

Supplementary References

1. Elumalai, N., Berg, A., Natarajan, K., Scharow, A. & Berg, T. Nanomolar Inhibitors of the Transcription Factor STAT5b with High Selectivity over STAT5a. *Angew. Chem. Int. Ed.* **54**, 4758–4763 (2015).

2. De, C. K., Pesciaioli, F. & List, B. Catalytic Asymmetric Benzidine Rearrangement. *Angew. Chem. Int. Ed.* **52**, 9293–9295 (2013).

3. He, Z., Pulis, A. P. & Procter, D. J. The Interrupted Pummerer Reaction in a Sulfoxide-Catalyzed Oxidative Coupling of 2-Naphthols. *Angew. Chem. Int. Ed.* **58**, 7813–7817 (2019).

4. Quasdorf, K. W., Riener, M., Petrova, K. V & Garg, N. K. Suzuki−Miyaura Coupling of Aryl Carbamates, Carbonates, and Sulfamates. *J. Am. Chem. Soc.* **131**, 17748–17749 (2009).

5. Xu, B. & Tambar, U. K. Remote Allylation of Unactivated C(sp^3)–H Bonds Triggered by Photogenerated Amidyl Radicals. *ACS Catal.* **9**, 4627–4631 (2019).

6. Kita, Y., Numajiri, Y., Okamoto, N. & Stoltz, B. M. Construction of tertiary chiral centers by Pd-catalyzed asymmetric allylic alkylation of prochiral enolate equivalents. *Tetrahedron* **71**, 6349–6353 (2015).

7. Schröder, S. P., Taylor, N. J., Jackson, P. & Franckevičius, V. Catalytic Decarboxylative Alkenylation of Enolates. *Org. Lett.* **15**, 3778–3781 (2013).

8. Olofson, R. A., Anh, D. V., Morrison, D. S. & De Cusati, P. F. Simple one-step preparations of vinylic carbonates from aldehydes. *J. Org. Chem.* **55**, 1–3 (1990).

9. Tobisu, M., Morioka, T., Ohtsuki, A. & Chatani, N. Nickel-catalyzed reductive cleavage of aryl alkyl ethers to arenes in absence of external reductant. *Chem. Sci.* **6**, 3410–3414 (2015).
